# Procalcitonin levels in candidemia versus bacteremia: a systematic review

**DOI:** 10.1186/s13054-019-2481-y

**Published:** 2019-05-28

**Authors:** Andrea Cortegiani, Giovanni Misseri, Mariachiara Ippolito, Matteo Bassetti, Antonino Giarratano, Ignacio Martin-Loeches, Sharon Einav

**Affiliations:** 1 Department of Surgical, Oncological and Oral Science (Di.Chir.On.S.). Section of Anesthesia, Analgesia, Intensive Care and Emergency, Policlinico Paolo Giaccone, University of Palermo, via del vespro 129, 90127 Palermo, Italy; 2 Infectious Diseases Division, Department of Medicine, University of Udine and Santa Maria della Misericordia University Hospital, Piazzale Santa Maria della Misericordia 15, Udine, Italy; 3 Multidisciplinary Intensive Care Research Organization (MICRO), St. James’s Hospital, Dublin, Ireland; 4 Hospital Clinic, Universidad de Barcelona, CIBERes, Barcelona, Spain; 5 Intensive Care Unit of the Shaare Zedek Medical Medical Centre and Hebrew University Faculty of Medicine, Jerusalem, Israel

**Keywords:** Procalcitonin, PCT, Sepsis, *Candida*, Fungi, Candidemia, Biomarker, Fungal

## Abstract

**Background:**

Procalcitonin (PCT) is a biomarker used to assess systemic inflammation, infection, and sepsis and to optimize antimicrobial therapies. Its role in the in the differential diagnosis between candidemia and bacteremia is unclear. The aim of this systematic review was to summarize the current evidence about PCT values for differentiating candidemia from bacteremia.

**Methods:**

PubMed and EMBASE were searched for studies reporting data on the diagnostic performance of serum PCT levels in intensive care unit (ICU) or non-ICU adult patients with candidemia, in comparison to patients with bacteremia.

**Results:**

We included 16 studies for a total of 45.079 patients and 785 cases of candidemia. Most studies claimed to report data relating to the use of PCT values for differentiating between candidemia and bacteremia in septic patients in the intensive care unit. However, the studies identified were all retrospective, except for one secondary analysis of a prospective dataset, and clinically very heterogeneous and involved different assessment methods. Most studies did show lower PCT values in patients with candidemia compared to bacteremia. However, the evidence supporting this observation is of low quality and the difference seems insufficiently discriminative to guide therapeutic decisions. None of the studies retrieved actually studied guidance of antifungal treatment by PCT. PCT may improve diagnostic performance regarding candidemia when combined with other biomarkers of infection (e.g., beta-d-glucan) but more data is needed.

**Conclusions:**

PCT should not be used as a standalone tool for the differential diagnosis between candidemia and bacteremia due to limited supporting evidence.

**Electronic supplementary material:**

The online version of this article (10.1186/s13054-019-2481-y) contains supplementary material, which is available to authorized users.

## Background

Early diagnosis of candidemia is challenging [1–3]. The absence of sensitive and specific clinical signs and symptoms and radiological findings as well as the prolonged time of blood culture growth hamper early identification of candidemia [2, 4]. Adding to this is the need to differentiate between bacterial and fungal infections, which often have similar clinical manifestations. For these reasons, risk factor clinical characteristics, scoring systems, and microbiological techniques (culture- and non-culture-based) are all being used to optimize early treatment and reduce unnecessary antifungal therapy [4–13].

Procalcitonin (PCT) has been proposed as a useful tool to characterize systemic inflammation, infection, and sepsis [14–16]. Findings from several randomized controlled trials indicate that the use of a PCT-guided antibiotic treatment algorithm (i.e., PCT guidance) is likely to reduce antibiotic exposure in septic patients, without an adverse effect on health outcomes [17]. PCT production is promoted by lipopolysaccharides and cytokines, which are expressed in pro-inflammatory conditions [18]. Although some non-bacterial inflammatory conditions increase PCT levels, bacterial infections typically show higher PCT serum concentration [14, 18, 19]. Some studies reported lower PCT serum levels in patients with candidemia compared to bacteremia [20, 21]. Although the mechanism for this finding is unclear, patients with invasive candidiasis showed signs of impaired inflammatory response, immune cell exhaustion, and reduced production of positive co-stimulatory molecules [22–24]. Thus, the serum levels of PCT may differ in patients with bacterial and *Candida* infections [1, 20, 21]. The aim of this systematic review was to summarize the current evidence about PCT values for differentiating candidemia from bacteremia.

## Methods

### Search strategy and selection process

For the purpose of this review, a search was conducted in PubMed and EMBASE (see Additional files 1 and 2). The terms used included “Candida” OR “fungi” AND “Procalcitonin” (see full search strategy in Additional file 1). We considered only articles published in peer-review journals in the English language. We excluded conference proceedings and case reports.

We selected studies reporting data on the values and diagnostic performance of PCT in intensive care unit (ICU) or non-ICU nonimmunosuppressed adult patients with microbiologically confirmed candidemia in comparison to patients with bacteremia. We also included studies in which data about PCT where reported separately for patients with candidemia from those with other fungal infections. If several samples of PCT were taken, we selected the value of the first available PCT sampled during the diagnostic process.

**Fig. 1:**
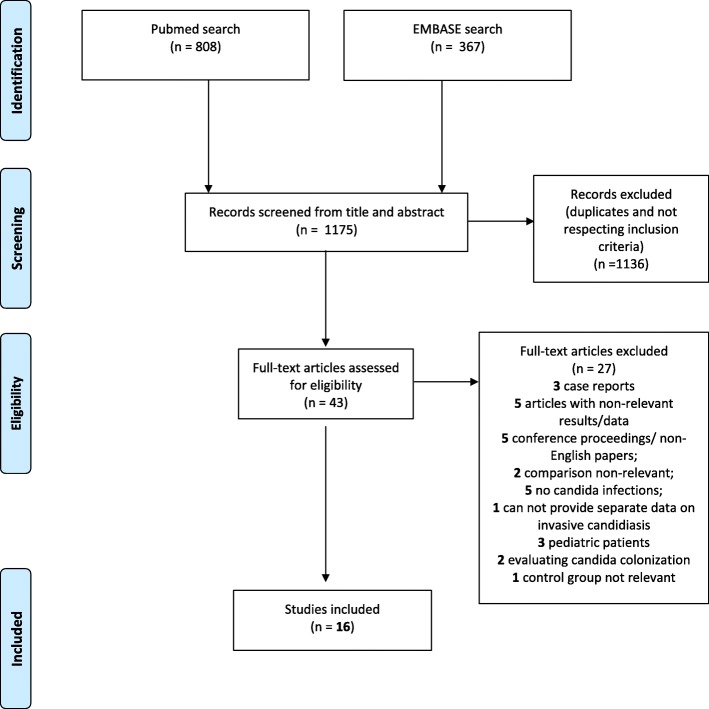
PRISMA flow chart of the systematic search

Two searches were run: the first in 5 October 2018 and the last in 20 February 2019. Two authors (AC, GM) independently screened all titles and abstracts to select potentially relevant papers. Papers selected for full review also underwent screening of their list of references by the same authors to identify additional potential studies of interest. Discrepancies between the two reviewers on relevance at any stage were adjudicated by two other authors (ES, AG). Papers selected for full review underwent data extraction if both reviewers (AC, GM) agreed on their relevance. In case of doubt at any stage, we contacted the corresponding authors of the manuscripts. Figure 1 describes paper inclusion/exclusion process.

## Results

### Characteristics of the included studies

The searches yielded overall 1175 articles (see Additional files 1 and 2). Among these, 43 were selected for full review but only 16 were ultimately selected for inclusion. These 16 studies included overall 45.079 adults and yielded of 785 cases of candidemia. Of these studies, 10 specifically referred to ICU patients. Twelve of the 16 included studies had at least sepsis as inclusion criteria; three studies did not report this information; in one study, the majority of patients were at least septic, but sepsis was not an inclusion criterion (Table 1). All studies were retrospective, except for one secondary analysis of a prospectively collected dataset.

**Table 1:** Study and clinical characteristics, microbiological findings, and PCT values in included studies

Author (year) [Ref]	Number of centers and setting	Design	Candidemia group	Causative microorganisms	Bacteremia group	Severity of infection *N* (%)	PCT level (ng/ml) in the candidemia group	PCT level (ng/ml) in the bacteremia group	PCT testing assay
Charles et al. (2006) [25]	Single center (ICU)	Retrospective study	11 patients with candidemia	*C*. *albicans* (13)	33 patients with bacteremia	Sepsis as inclusion criteria. Septic shock: 19 (54.3%) with bacteremia, 8 (53.3%) with candidemia Severe sepsis: 12 (34.3%) with bacteremia, 7 (46.7%) with candidemia	0.65 [0.08–5.46]	9.75 [1–259.5]	Kryptor
Martini et al. (2010) [26]	Single center (ICU)	Observational prospective study	17 patients with candidemia	*C. albicans* (6); *C. parapsilosis* (6); *C. glabrata* (4); *C. tropicalis* (3); *Candida* spp. and bacteria (2)	16 patients with bacteremia	Sepsis as inclusion criteria	0.71 [0.5–1.1]	12.9 [2.6–81.2]	LUMItest
Fu et al. (2012) [27]	Single centre (ICU)	Prospective observational study	23 patients with candidemia	*Candida* spp.	39 patients with bacteremia	Sepsis as inclusion criteria	1.0 [0.5–7.3]	G− 20.9 [12.4–40.7]; G + 10.0 [2.9–19.7]	E170
Brodskà et al. (2013) [28]	Single center (ICU)	Retrospective study	5 patients with candidemia	*Candida* spp.	161 patients with bacteremia	Sepsis as inclusion criteria	0.58 [0.35–0.73]	G− 8.90 [1.88–32.60]; G+ 0.73 [0.22–3.40]	ECLIA
Held et al. (2013) [29]	Single center (hospitalized patients)	Retrospective case–control study	56 patients with candidemia	*C. albicans* (32); *C. glabrata* (11); *C. tropicalis* (6); *C. parapsilosis* (4); *C. guilliermondii* (3)	100 patients with bacteremia	NA	0.80 (NA)	2.36 (NA)	NA
Cortegiani et al. (2014) [30]	Single center (ICU)	Retrospective study	18 patients with candidemia; 31 mixed BSI	*C. albicans* (13); *C. parapsilosis* (9); *C. glabrata* (2); *C. krusei* (1); *Candida* spp. *+*bacteria (31)	151 cases of bacteremia	Severe sepsis or septic shock as inclusion criteria. Septic shock: 52 (34.4%) with bacteremia; 9 (29%) with mixed; 7 (31.8%) with *Candida*; 83 (31.9%) overall	0.99 [0.86–1.34]; Mixed BSI: 4.76 [2.98–6.08]	Bacteremia 16.75 [7.65–50.5]	Kryptor
Leli et al. (2015) [31]	Single center (medical ward)	Prospective observational study	20 patients with candidemia	*C. albicans* (12); *C. lusitaniae* (5); *C. parapsilosis* (3)	562 patients with bacteremia	Sepsis as inclusion criteria	0.5 [0.4–1]	G + 2.1 [0.6–7.6]; G− 13.8 [3.4–44.1]	VIDAS
Miglietta et al. (2015) [32]	Single center (ICU)	Retrospective study	33 patients with candidemia	*C. albicans* (17); other *Candida* spp. (16)	70 patients with bacteremia	Sepsis as inclusion criteria	0.55 [0.36–0.9]	10.2 [1.28–25.3]	NA
Oussalah et al. (2015) [33]	Multi-center (67 healthcare departments)	Retrospective cross-sectional study	256 patients with candidemia	NA	2443 patients with bacteremia	NA	1 [0.3–2.7]	G− 2.2 [0.6–12.2]; G+ 1.3 [0.3–6.9]	Kryptor
Li et al. (2016) [34]	Single center (hospitalized patients)	Retrospective study	16 cases of candidemia	*C. albicans* (8); *C. parapsilosis* (8)	328 cases of bacteremia	Sepsis as inclusion criteria	*C. albicans* 1.00 [0.30–2.65]; *C. parapsilosis* 0.73 [0.23–1.60]	G− 7.47 [1.09–41.26]; G+ 0.48 [0.15–2.16]	VIDAS
Giacobbe et al. (2017) [35]	Multi-center (3 ICUs)	Retrospective study	73 critically ill adult patients with candidemia	*C. albicans* (37); *C. parapsilosis* (23); *C. tropicalis* (7); *C. glabrata* (4); *C. guilliermondii* (1); *C. lusitaniae* (1)	93 patients with bacteremia	NA	0.76 [NA]	4.32 [NA]	CLIA
Pieralli et al. (2017) [36]	Single center (internal medicine wards)	Retrospective case–control study	64 patients with candidemia	*C. albicans* (42); *C. parapsilosis* (16); *C. glabrata* (8); *C. tropicalis* (2); *C. krusei* (1); *C. albicans + C. glabrata* (3); *C. albicans + C. parapsilosis* (1); *C. parapsilosis + C. krusei* (1)	128 patients with bacteremia	Sepsis as inclusion criteria	0.73 [0.26–1.85]	4.48 [1.10–18.26]	VIDAS
Yan et al. (2017) [37]	Single center (ICU and EM department)	Retrospective study	26 cases of candidemia	*C. albicans* (19); *C. parapsilosis* (5); *C. tropicalis* (2)	456 cases of bacteremia	Sepsis as inclusion criteria	*C. albicans* 1.11 [0.41–2.24]; *C. parapsilosi*s 0.79 [0.40–1.70]; C. tropicalis 5.37 [0.29–10.45]	G− 2.42 [0.38–15.52]; G+ 0.49 [0.13–5.89]	VIDAS
Bassetti et al. (2018) [38]	Single center (ICU)	Retrospective case–control study	11 patients with candidemia	*Candida* spp.	247 patients with positive BC (other than *Candida*)	Sepsis or septic shock: 46 (43.4%) with G−; 70 (49.6%) with G+; 5 (45.4%) with *Candida*	2.1 ± 1.8	G− 25.1 ± 19.9; G+ 29.9 ± 13.2	NA
Murri et al. (2018) [39]	Single centre (hospitalized patients)	Retrospective cohort study	83 patients with candidemia	*Candida* spp. (59); mixed (G + and *Candida* spp.) (24)	263 patients with bacteremia	Sepsis as inclusion criteria	1.07 (5.9) alone; mixed with G− 0.1 (± 0.1); mixed with G+ 3.1 (±12.2)	G− 12.2 (±28.6); G+ 3.4 (±16.6)	ADVIA Centaur
Thomas-Ruddel et al. (2018) [40]	Multi-center (ICUs)	Secondary analysis of a cluster randomized trial	65 patients with candidemia	*C. albicans* (57); Candida other spp. (37)	815 patients with G− bacteremia; 948 with G+ bacteremia	Sepsis with organ dysfunction as inclusion criteria; Septic shock: 1137 (57.3%) with positive BCs; 2714 (55.9%) overall	4.7 [2–14]	G− 26 ng/ml [7.7–63.1]; G+ 7.1 ng/ml [2.0–23.3]	NA

Procalcitonin (PCT) values are reported in nanograms per milliliter unless otherwise indicated. The reported PCT values refer to the first timepoint of diagnostic assessment. Values are reported as median [IQR] or as mean (± SD)

*IQR* interquartile range, *SD* standard deviation, *BC* blood cultures, *BSI* blood stream infections, *EM* emergency medicine, *G* Gram, *ICU* intensive care unit, *NA* not available, *PCT* procalcitonin, *SIRS* systemic inflammatory response syndrome

Table 1 presents data from the included studies, including study design, patient characteristics, microbiological findings, assays used for dosing, and the information given on the diagnostic performance of PCT. Following qualitative synthesis of the data, a decision was made to not to proceed to meta-analysis because of the heterogeneity found in patient populations (study and control groups) and the assays used, as well as the amount of missing data (i.e., large risk of bias). Instead, we hereby summarize the evidence from included studies.

### PCT levels for differentiating candidemia from bacteremia

#### Studies in the ICU

In a retrospective cohort study, Charles et al. evaluated 50 non-surgical septic ICU patients with bloodstream infection (BSI). They found significantly lower PCT levels in patients with candidemia (median 0.65 ng/ml [range 0.08–1.56], *n* = 15) compared to those with bacteremia (median 9.75 ng/ml [range 1.00–259.5]). PCT levels < 5.5 ng/ml had a negative predictive value (NPV) of 100% and a positive predictive value (PPV) of 65% for *Candida* spp. sepsis [25].

Martini et al. prospectively studied 48 post-surgery septic ICU patients. PCT levels were lower in candidemia (0.71 [IQR 0.5–1.1], *n* = 17) than in bacterial BSI (12.9 [IQR 2.6–81.2]) [26].

Brodska et al. retrospectively studied 166 ICU septic patients with BSI. Significantly higher PCT levels were observed with Gram-negative pathogens (8.90 ng/ml [IQR 1.88–32.60]) than with Gram-positive pathogens (0.73 ng/ml [IQR 0.22–3.40]) or *Candida* spp. (0.58 [IQR 0.35–0.73], *n* = 5) [28].

Cortegiani et al. retrospectively studied PCT levels and blood cultures in 182 ICU patients with sepsis (60% post-surgical). Significantly lower levels of PCT were found in cases with candidemia (0.99 ng/ml [IQR 0.86–1.34], *n* = 22) than in cases with bacterial BSI (16.7 ng/ml [IQR 7.52–50.2]) or mixed BSI (4.76 ng/ml [IQR 2.98–6.08]). A PCT cut-off value ≤ 6.08 ng/ml demonstrated a PPV of 63.9% and a NPV of 96.3% for identifying *Candida* spp. [30].

Miglietta et al. retrospectively studied 145 septic ICU patients (mostly medical). Significantly lower PCT levels were found in patients with candidemia (0.55 [IQR 0.36–0.91], *n* = 33) than in patients with bacteremia (10.2 [IQR 1.28–25.3]). However, PCT was unable to differentiate between candidemia and a systemic inflammatory response without infection [32].

Yan et al. retrospectively evaluated 414 septic patients in the ICU and emergency department with positive blood culture [37]. They found a median PCT level of 1.11 [0.41–2.24] in 19 candidemias caused by *C. albicans*, 0.79 [IQR 0.4–1.7] in 5 candidemias by *C. parapsilosis* and 5.37 [0.29–10.45] in 2 candidemias by *C. tropicalis*.

Bassetti et al. retrospectively compared 258 ICU patients with positive blood culture (cases) to 213 controls. In cases with candidemia (*n* = 11), the serum PCT concentration was 2.1 ng/ml (SD 1.8), significantly lower than in Gram-positive or Gram-negative BSI [38].

Thomas-Rüddel et al. performed a secondary analysis of a prospectively collected dataset involving 4858 septic patients with at least one related organ dysfunction from the ICUs of 40 hospitals [40]. PCT values at sepsis onset were analyzed in patients with bacteremia or candidemia but mixed infections were excluded. PCT values were significantly higher in patients with Gram-negative (26 ng/ml [IQR 7.7–63.1]) than Gram-positive bacteremia (7.1 ng/ml [IQR 2.0–23.3]) or candidemia (4.7 ng/ml [IQR 1.9–13.7], *n* = 63).

#### Studies in wards or including hospitalized patients

Pieralli et al. retrospectively compared 64 cases with sepsis due to *Candida* spp. and 128 cases with sepsis due to bacteria in 3 internal medicine wards [36]. PCT levels were significantly lower in candidemia than in bacteremia (0.73 ng/ml [IQR 0.26–1.85] and 4.48 ng/ml [IQR 1.10–18.26], respectively). The best cut-off was 2.5 ng/ml, with a NPV of 98.3% and a PPV of 15.1%.

Oussalah et al. performed a cross-sectional, single-center study of 35.343 patients with suspected BSI [33]. Significantly lower PCT levels were found in patients with candidemia (1.0 ng/ml [IQR 0.3–2.7], *n* = 256) compared to patients with Gram-positive (1.3 ng/ml [IQR 0.3–6.9]) and Gram-negative BSI (2.2 ng/ml [IQR 0.6–12.2]). However, these levels were also higher than those in patients with negative blood culture (0.3 ng/ml [IQR 0.1–1.1]).

Li et al. retrospectively evaluated PCT levels in 292 septic patients in a single center. PCT levels were lower in patients with sepsis caused by *C. parapsilosis* (0.60 [IQR 0.14–2.06], *n* = 8) or by *C. albicans* (1.00 [IQR 0.30–2.65], *n* = 8) than in patients with Gram-negative sepsis (7.47 [IQR 1.09–41.26]). No difference was found between patients with sepsis caused by *Candida* spp. versus Gram-positive bacteria (0.48 [IQR 0.15–2.16]) [34].

Leli et al. prospectively observed 1.949 patients (89% from medical ward) and found that a cut-off value of 1.6 ng/ml differentiates Gram-negative BSI from candidemia and a cut-off value of 1.3 ng/ml differentiates Gram-positive BSI from candidemia (*n* = 24). Patients with candidemia presented with a median PCT value of 0.5 ng/ml [IQR 0.4–1] [31].

Murri et al. retrospectively studied 401 patients hospitalized with sepsis and BSI. Those with candidemia (*n* = 55) had significantly lower PCT levels (0.8 ng/ml, SD 4.9) than those with Gram-positive (2.8 ng/ml, SD 16.6) or Gram-negative BSI (10.4 ng/ml, SD 26.9) [39]. In mixed infections, PCT levels were 2.1 ng/ml (SD 10.0) and 0.1 ng/ml (SD 0.1) for *Candida* spp. with Gram-positive and Gram-negative bacteria, respectively.

### PCT use in association with other biomarkers

PCT has been also evaluated in combination with other biomarkers for improving performance in diagnosis of IC [29, 35].

Giacobbe et al. retrospectively assessed the combination of PCT and beta-d-glucan (BDG) in 166 critically ill ICU patients for early differentiation between bacteremia and candidemia [35]. Compared to patients with bacteremia, the levels of PCT were lower (median 0.76 vs. 4.32 ng/ml, *p* < 0.001) and those of BDG were higher (median > 500 vs. < 80 pg/ml, *p* < 0.001) in patients affected by candidemia. Combining the standard BDG cut-off level (≥ 80 pg/ml) with the rounded optimal PCT cut-off level (< 2 ng/ml) yielded a higher PPV for identifying the presence of candidemia than the PPV of either test alone. Held et al. similarly reported that the combination of BDG and PCT increased specificity (from 89.4 to 96.2%), but this was accompanied by loss of sensitivity (from 86.7 to 51.7%) for candidemia in 56 hospitalized patients [29].

Fu et al. found that the combination of PCT (cut-off 8.06 ng/ml), CRP (cut-off value 116 mg/l), and IL-6 (cut-off 186.5 pg/ml) increased the sensitivity and specificity for early diagnosis of candidemia (*n* = 23) and its distinction from Gram-positive/negative bacteremia (AUC to 0.912) in 85 ICU septic patients [27]. However, PCT showed the best diagnostic performance, when compared to CRP or IL-6.

## Discussion

In this systematic review of the value of PCT for differentiating between candidemia and bacteremia, we found that PCT has been studied in only 785 cases of candidemia. We limited our analysis to adult nonimmunosuppressed patients with bloodstream infections related to *Candida* spp. to reduce clinical heterogeneity.

Most of the studies identified evaluated the use of PCT for differentiating between candidemia and bacteremia in septic patients in the ICU. We found no study specifically evaluating PCT levels as a tool for monitoring the effect of antifungal treatment.

Although most of these studies showed lower PCT values in patients with candidemia compared to bacteremia, the evidence supporting this observation is of low quality. Moreover, this difference seems to be insufficiently discriminative to guide therapeutic decisions.

PCT may improve diagnostic performance when combined with other biomarkers of infection. Of note, the association with BDG may be of interest due its widespread use and specific role in this setting [2, 41]. However, this finding requires additional confirmation.

Our systematic review has several limitations. We could not proceed with meta-analysis because the studies identified were clinically very heterogeneous, involving different assessment methods and comparators. This may limit the impact of our findings but should be mostly seen as a limitation of the available evidence rather than of the review*.* Another limitation is the inability to separate the results and conclusions according to septic state (e.g., sepsis, septic shock). However, most studies did use sepsis as inclusion criteria or included mostly septic patients (13 out of 16 studies). We were unable to select studies where a surrogate of fungal infection (e.g., beta-d-glucan) was sampled alongside PCT since only one study included such data. The timing of blood sampling for PCT levels varied among the included studies. However, for all studies, we considered the value of the first available PCT sampled during the diagnostic process.

## Conclusions

PCT should not be used as a standalone tool for the differential diagnosis between candidemia and bacteremia due to limited supporting evidence. In this setting, PCT values seem to be insufficiently discriminative to guide therapeutic decisions. PCT should be further investigated in antifungal stewardship programs, in association with other biomarkers or non-culture diagnostic tests.

## Additional files

Additional file 1: Search output from PubMed. Full search output from PubMed. (DOCX 299 kb)

Additional file 2: Search output from EMBASE. Full search output from EMBASE. (DOCX 88 kb)

## Data Availability

All related data are reported in the text or in additional files.

## Abbreviations

AUC: Area under the curve
BDG: Beta-d-glucan
BSI: Blood stream infection
CRP: C-reactive protein
IC: Invasive candidiasis
ICU: Intensive care unit
NPV: Negative predictive value
PCT: Procalcitonin
PPV: Positive predictive value
SD: Standard deviation
