# Correction to: Procalcitonin levels in candidemia versus bacteremia: a systematic review

**DOI:** 10.1186/s13054-019-2598-z

**Published:** 2019-10-11

**Authors:** Andrea Cortegiani, Giovanni Misseri, Mariachiara Ippolito, Matteo Bassetti, Antonino Giarratano, Ignacio Martin-Loeches, Sharon Einav

**Affiliations:** 1 Department of Surgical, Oncological and Oral Science (Di.Chir.On.S.). Section of Anesthesia, Analgesia, Intensive Care and Emergency, Policlinico Paolo Giaccone. University of Palermo, via del vespro 129, 90127 Palermo, Italy; 2 Infectious Diseases Division, Department of Medicine, University of Udine and Santa Maria della Misericordia University Hospital. Piazzale Santa Maria della Misericordia 15, Udine, Italy; 3 Multidisciplinary Intensive Care Research Organization (MICRO), St. James’s Hospital, Dublin, Ireland; 4 Hospital Clinic, Universidad de Barcelona, CIBERes, Barcelona, Spain; 5 Intensive Care Unit of the Shaare Zedek Medical Medical Centre and Hebrew, University Faculty of Medicine, Jerusalem, Israel

**Correction to: Crit Care (2019)23:190**

**https://doi.org/10.1186/s13054-019-2481-y**

The author wish to note there are three imprecisions in the article [1]. Part of the sentence “All the studies identified were retrospective, except for three prospective and one secondary analysis of a prospectively collected dataset” was omitted, leading to the declaration that “…the studies identified were all retrospective, except for one secondary analysis of a prospective dataset” in the “Results” of the abstract and “All studies were retrospective, except for one secondary analysis of a prospectively collected dataset” under the subheading “Characteristics of the included studies”. Table 1 reports the correct number of prospective analyses. Also, the number of excluded records in Fig. 1 should be 1132 and not 1136. Finally, the procalcitonin (PCT) value in the Gram + group in the paper by Basetti et al. [2] should be 8.9 ± 7.5 in Table 1. This has now been included in this correction article.

## Data Availability

All related data are reported in the text or in additional filies.

## Abbreviation

PCT: Procalcitonin
