# Supplementary material for: Procalcitonin levels in candidemia versus bacteremia: a systematic review
Source: Crit Care. 2019 May 28;23:190. doi: 10.1186/s13054-019-2481-y (PMC6537202; doi:10.1186/s13054-019-2481-y)
Supplement: Supplementary file 1 — Search output from PubMed. Full search output from PubMed. (DOCX 299 kb) [file 13054_2019_2481_MOESM1_ESM.docx]

**PUBMED**

**Search Strategy:** ("procalcitonin"[MeSH Terms] OR "procalcitonin"[All Fields]) AND (("candida"[MeSH Terms] OR "candida"[All Fields]) OR ("microbiology"[Subheading] OR "microbiology"[All Fields] OR "fungi"[All Fields] OR "fungi"[MeSH Terms]))

1: Tascini C, Carannante N, Sodano G, Tiberio C, Atripaldi L, Di Caprio G, Sozio

E, Sbrana F, Ripoli A, Menchini C, Galli L, Di Sarno R, Chiappini E, Sarno M,

Siani P. Neonatal pertussis diagnosis: low procalcitonin level and high

lymphocyte count are able to discriminate pertussis from bacterial and viral

infections. New Microbiol. 2019 Feb 20;42(1). [Epub ahead of print] PubMed PMID:

30785208.

2: Ozer Balin S, Sagmak Tartar A, Uğur K, Kilinç F, Telo S, Bal A, Balin M,

Akbulut A. Pentraxin-3: A new parameter in predicting the severity of diabetic

foot infection? Int Wound J. 2019 Feb 15. doi: 10.1111/iwj.13075. [Epub ahead of

print] PubMed PMID: 30767386.

3: Chambliss AB, Hayden J, Colby JM. Evaluation of procalcitonin immunoassay

concordance near clinical decision points. Clin Chem Lab Med. 2019 Feb 14. pii:

/j/cclm.ahead-of-print/cclm-2018-1362/cclm-2018-1362.xml. doi:

10.1515/cclm-2018-1362. [Epub ahead of print] PubMed PMID: 30763263.

4: Schuetz P, Beishuizen A, Broyles M, Ferrer R, Gavazzi G, Gluck EH, González

Del Castillo J, Jensen JU, Kanizsai PL, Kwa ALH, Krueger S, Luyt CE, Oppert M,

Plebani M, Shlyapnikov SA, Toccafondi G, Townsend J, Welte T, Saeed K.

Procalcitonin (PCT)-guided antibiotic stewardship: an international experts

consensus on optimized clinical use. Clin Chem Lab Med. 2019 Feb 6. pii:

/j/cclm.ahead-of-print/cclm-2018-1181/cclm-2018-1181.xml. doi:

10.1515/cclm-2018-1181. [Epub ahead of print] PubMed PMID: 30721141.

5: Takahashi K, Ozawa E, Nakao K, Aoki S, Takase Y. Hepatobiliary and Pancreatic:

A procalcitonin-secreting and calcitonin-secreting pancreatic neuroendocrine

carcinoma. J Gastroenterol Hepatol. 2019 Jan 21. doi: 10.1111/jgh.14568. [Epub

ahead of print] PubMed PMID: 30663800.

6: Daef EA, Elsherbiny NM, Agban MN, Riad KF, Mohammed LF. Bloodstream Infections

in Febrile Neutropenic Pediatric Cancer Patients: Microbiological and Sepsis

Biomarkers Insight. Egypt J Immunol. 2018 Jun;25(2):21-34. PubMed PMID: 30600945.

7: Esposito S, Rinaldi VE, Argentiero A, Farinelli E, Cofini M, D'Alonzo R,

Mencacci A, Principi N. Approach to Neonates and Young Infants with Fever without

a Source Who Are at Risk for Severe Bacterial Infection. Mediators Inflamm. 2018

Nov 26;2018:4869329. doi: 10.1155/2018/4869329. eCollection 2018. Review. PubMed

PMID: 30581369; PubMed Central PMCID: PMC6287153.

8: Sen P, Demirdal T, Nemli SA, Vardar I, Kizilkaya M, Sencan A, Yilmaz HE.

Infection markers as predictors of Bacteremia in an Intensive Care Unit: A

prospective study. Pak J Med Sci. 2018 Nov-Dec;34(6):1517-1524. doi:

10.12669/pjms.346.15665. PubMed PMID: 30559815; PubMed Central PMCID: PMC6290214.

9: Gupta S, Jaswani P, Sharma RK, Agrawal S, Prasad N, Sahu C, Gupta A, Prasad

KN. Procalcitonin as a diagnostic biomarker of sepsis: A tertiary care centre

experience. J Infect Public Health. 2018 Nov 26. pii: S1876-0341(18)30312-5. doi:

10.1016/j.jiph.2018.11.004. [Epub ahead of print] PubMed PMID: 30497960.

10: Liu WC, Zhan YP, Wang XH, Hou BC, Huang J, Chen SB. Comprehensive

preoperative regime of selective gut decontamination in combination with

probiotics, and smectite for reducing endotoxemia and cytokine activation during

cardiopulmonary bypass: A pilot randomized, controlled trial. Medicine

(Baltimore). 2018 Nov;97(46):e12685. doi: 10.1097/MD.0000000000012685. PubMed

PMID: 30431563; PubMed Central PMCID: PMC6257461.

11: Shafiq N, Gautam V, Pandey AK, Kaur N, Garg S, Negi H, Kaur S, Ray P,

Malhotra S. Authors' Response. Indian J Med Res. 2018 Sep;148(3):349-350. doi:

10.4103/0971-5916.245302. PubMed PMID: 30425230; PubMed Central PMCID:

PMC6251257.

12: Chen C, Yan M, Hu C, Lv X, Zhang H, Chen S. Diagnostic efficacy of serum

procalcitonin, C-reactive protein concentration and clinical pulmonary infection

score in Ventilator-Associated Pneumonia. Med Sci (Paris). 2018 Oct;34 Focus

issue F1:26-32. doi: 10.1051/medsci/201834f105. Epub 2018 Nov 7. PubMed PMID:

30403171.

13: Zheng W, Liang X, Shui L, Ye B, Lou G, Liu Y, Zheng M. Serum Procalcitonin

Correlates with Renal Function in Hepatitis B Virus-Related Acute-on-Chronic

Liver Failure. Cell Physiol Biochem. 2018;50(5):1794-1803. doi:

10.1159/000494820. Epub 2018 Nov 5. PubMed PMID: 30396164.

14: Bamba Y, Moro H, Aoki N, Koizumi T, Ohshima Y, Watanabe S, Sakagami T, Koya

T, Takada T, Kikuchi T. Increased presepsin levels are associated with the

severity of fungal bloodstream infections. PLoS One. 2018 Oct 31;13(10):e0206089.

doi: 10.1371/journal.pone.0206089. eCollection 2018. PubMed PMID: 30379880;

PubMed Central PMCID: PMC6209217.

15: Tagarro A, Martín MD, Del-Amo N, Sanz-Rosa D, Rodríguez Md PhD M, Galán Md

PhD JC, Otheo E. Hyponatremia in children with pneumonia rarely means SIADH.

Paediatr Child Health. 2018 Nov;23(7):e126-e133. doi: 10.1093/pch/pxy003. Epub

2018 Mar 21. PubMed PMID: 30374221; PubMed Central PMCID: PMC6199641.

16: Dat VQ, Long NT, Hieu VN, Phuc NDH, Kinh NV, Trung NV, van Doorn HR, Bonell

A, Nadjm B. Clinical characteristics, organ failure, inflammatory markers and

prediction of mortality in patients with community acquired bloodstream

infection. BMC Infect Dis. 2018 Oct 26;18(1):535. doi: 10.1186/s12879-018-3448-3.

PubMed PMID: 30367601; PubMed Central PMCID: PMC6204014.

17: Sa-Ngasoongsong P, Wongsak S, Jarungvittayakon C, Limsamutpetch K, Channoom

T, Kawinwonggowit V. Comparison of Synovial Fluid and Serum Procalcitonin for

Diagnosis of Periprosthetic Joint Infection: A Pilot Study in 32 Patients. Biomed

Res Int. 2018 Oct 1;2018:8351308. doi: 10.1155/2018/8351308. eCollection 2018.

PubMed PMID: 30364023; PubMed Central PMCID: PMC6188588.

18: Mikuła T, Sapuła M, Jabłońska J, Kozłowska J, Stańczak W, Krankowska D,

Wiercińska-Drapało A. Significance of Heparin-Binding Protein and D-dimers in the

Early Diagnosis of Spontaneous Bacterial Peritonitis. Mediators Inflamm. 2018 Sep

30;2018:1969108. doi: 10.1155/2018/1969108. eCollection 2018. PubMed PMID:

30363905; PubMed Central PMCID: PMC6186319.

19: Pitarch A, Nombela C, Gil C. Diagnosis of Invasive Candidiasis: From Gold

Standard Methods to Promising Leading-edge Technologies. Curr Top Med Chem.

2018;18(16):1375-1392. doi: 10.2174/1568026618666181025093146. Review. PubMed

PMID: 30360714.

20: Taymaz T, Ergönül Ö, Kebapcı A, Okyay R. Significance of the detection of

influenza and other respiratory viruses for antibiotic stewardship: Lessons from

the post-pandemic period. Int J Infect Dis. 2018 Dec;77:53-56. doi:

10.1016/j.ijid.2018.10.003. Epub 2018 Oct 10. PubMed PMID: 30315991.

21: Pieróg J, Bielewicz M, Wójcik J, Kubisa B, Kaseja K, Safranow K, Dołęgowska

B, Waloszczyk P, Grodzki T. Evaluation of the metabolic response to open and

minimally invasive resection of the oesophagus due to oesophageal cancer.

Kardiochir Torakochirurgia Pol. 2018 Sep;15(3):151-156. doi:

10.5114/kitp.2018.78438. Epub 2018 Sep 24. PubMed PMID: 30310392; PubMed Central

PMCID: PMC6180019.

22: van der Does Y, Rood PPM, Ramakers C, Schuit SCE, Patka P, van Gorp ECM,

Limper M. Identifying patients with bacterial infections using a combination of

C-reactive protein, procalcitonin, TRAIL, and IP-10 in the emergency department:

a prospective observational cohort study. Clin Microbiol Infect. 2018

Dec;24(12):1297-1304. doi: 10.1016/j.cmi.2018.09.007. Epub 2018 Sep 28. PubMed

PMID: 30268671.

23: Legriel S, Grigoresco B, Martel P, Henry-Lagarrigue M, Lvovschi V, Troché G,

Amara M, Jacq G, Bruneel F, Bernard M, Marinier A, Bedos JP; PCT INHAL study

group. Diagnostic Accuracy of Procalcitonin for Early Aspiration Pneumonia in

Critically Ill Patients with Coma: A Prospective Study. Neurocrit Care. 2018 Sep

28. doi: 10.1007/s12028-018-0623-8. [Epub ahead of print] PubMed PMID: 30267280.

24: Quah J, Jiang B, Tan PC, Siau C, Tan TY. Impact of microbial Aetiology on

mortality in severe community-acquired pneumonia. BMC Infect Dis. 2018 Sep

4;18(1):451. doi: 10.1186/s12879-018-3366-4. PubMed PMID: 30180811; PubMed

Central PMCID: PMC6122562.

25: Corbacho Re MF, Rocchetti NS, Settecase CJ, Bagilet DH. Diagnostic value of

procalcitonin in ventilator-associated pneumonia. Med Clin (Barc). 2018 Aug 25.

pii: S0025-7753(18)30441-X. doi: 10.1016/j.medcli.2018.06.019. [Epub ahead of

print] English, Spanish. PubMed PMID: 30154009.

26: Zhao Z, Li X, Zhao Y, Wang D, Li Y, Liu L, Sun T, Chen G. Role of C-reactive

protein and procalcitonin in discriminating between infectious fever and tumor

fever in non-neutropenic lung cancer patients. Medicine (Baltimore). 2018

Aug;97(33):e11930. doi: 10.1097/MD.0000000000011930. PubMed PMID: 30113495;

PubMed Central PMCID: PMC6112972.

27: Gupta P, Kale P, Khillan V. Resurgence of Global Opportunistic

Multidrug-resistant Stenotrophomonas maltophilia. Indian J Crit Care Med. 2018

Jul;22(7):503-508. doi: 10.4103/ijccm.IJCCM_106_18. PubMed PMID: 30111925; PubMed

Central PMCID: PMC6069309.

28: Ratzinger F, Haslacher H, Perkmann T, Pinzan M, Anner P, Makristathis A,

Burgmann H, Heinze G, Dorffner G. Machine learning for fast identification of

bacteraemia in SIRS patients treated on standard care wards: a cohort study. Sci

Rep. 2018 Aug 15;8(1):12233. doi: 10.1038/s41598-018-30236-9. PubMed PMID:

30111827; PubMed Central PMCID: PMC6093921.

29: Giamarellos-Bourboulis EJ, Kotsaki A, Routsi C, Graziano E, Righi E, Bassetti

M. A prognostic score for the resolution of bacteremia by Gram-negative bacteria

resistant to carbapenems. Eur J Clin Microbiol Infect Dis. 2018

Nov;37(11):2083-2089. doi: 10.1007/s10096-018-3342-3. Epub 2018 Aug 6. PubMed

PMID: 30083886.

30: Aydemir C, Aydemir H, Kokturk F, Kulah C, Mungan AG. The cut-off levels of

procalcitonin and C-reactive protein and the kinetics of mean platelet volume in

preterm neonates with sepsis. BMC Pediatr. 2018 Aug 1;18(1):253. doi:

10.1186/s12887-018-1236-2. PubMed PMID: 30068303; PubMed Central PMCID:

PMC6090766.

31: Mustafić S, Brkić S, Prnjavorac B, Sinanović A, Porobić Jahić H, Salkić S.

Diagnostic and prognostic value of procalcitonin in patients with sepsis. Med

Glas (Zenica). 2018 Aug 1;15(2):93-100. doi: 10.17392/963-18. PubMed PMID:

30047536.

32: Vitkon-Barkay I, Lazarovitch T, Marchaim D, Zaidenstein R, Temkin E, Martin

ET, Segaloff HE, Litovchik I, Rum V, Richter C, Tzuman O, Vered Z, Minha S.

Usefulness of Serum Procalcitonin as a Markerfor Coexisting Infection in Patients

With Acute Myocardial Infarction. Am J Cardiol. 2018 Sep 1;122(5):729-734. doi:

10.1016/j.amjcard.2018.05.004. Epub 2018 Jun 2. PubMed PMID: 30037423.

33: Chernyshov S, Alexeev M, Rybakov E, Tarasov M, Shelygin Y, Zarodniuk I,

Sukhina M. Risk factors and inflammatory predictors for Anastomotic Leakage

following Total Mesorectal Excision with defunctioning stoma. Pol Przegl Chir.

2018 May 16;90(3):31-36. doi: 10.5604/01.3001.0011.8169. PubMed PMID: 30015322.

34: Schuetz P, Bolliger R, Merker M, Christ-Crain M, Stolz D, Tamm M, Luyt CE,

Wolff M, Schroeder S, Nobre V, Reinhart K, Branche A, Damas P, Nijsten M,

Deliberato RO, Verduri A, Beghé B, Cao B, Shehabi Y, Jensen JS, Beishuizen A, de

Jong E, Briel M, Welte T, Mueller B. Procalcitonin-guided antibiotic therapy

algorithms for different types of acute respiratory infections based on previous

trials. Expert Rev Anti Infect Ther. 2018 Jul;16(7):555-564. doi:

10.1080/14787210.2018.1496331. Epub 2018 Jul 13. Review. PubMed PMID: 29969320.

35: Candel FJ, Borges Sá M, Belda S, Bou G, Del Pozo JL, Estrada O, Ferrer R,

González Del Castillo J, Julián-Jiménez A, Martín-Loeches I, Maseda E, Matesanz

M, Ramírez P, Ramos JT, Rello J, Suberviola B, Suárez de la Rica A, Vidal P.

Current aspects in sepsis approach. Turning things around. Rev Esp Quimioter.

2018 Aug;31(4):298-315. Epub 2018 Jun 25. Review. PubMed PMID: 29938972; PubMed

Central PMCID: PMC6172679.

36: Iacovelli A, Spaziante M, Al Moghazi S, Giordano A, Ceccarelli G, Venditti M.

A challenging case of carbapenemase-producing Klebsiella pneumoniae septic

thrombophlebitis and right mural endocarditis successfully treated with

ceftazidime/avibactam. Infection. 2018 Oct;46(5):721-724. doi:

10.1007/s15010-018-1166-9. Epub 2018 Jun 20. Erratum in: Infection. 2018 Jul

10;:. PubMed PMID: 29926399.

37: Kanashvili B, Saganelidze K, Ratiani L. RECENT PRINCIPLES OF ANTIMICROBIAL

TREATMENT IN POLYTRAUMA INDUCED SEPSIS AND SEPTIC SHOCK (REVIEW). Georgian Med

News. 2018 May;(278):72-80. Review. PubMed PMID: 29905549.

38: Wang Q, Tian XB, Liu W, Zhang LX. Procalcitonin as a diagnostic indicator for

systemic bacterial infections in patients with Stevens-Johnson syndrome/toxic

epidermal necrolysis. J Dermatol. 2018 Aug;45(8):989-993. doi:

10.1111/1346-8138.14488. Epub 2018 Jun 13. PubMed PMID: 29897140.

39: Sun J, Xiao Y, Zhang M, Ao T, Lang S, Wang J. Serum Inflammatory Markers in

Patients with Adenovirus Respiratory Infection. Med Sci Monit. 2018 Jun

7;24:3848-3855. doi: 10.12659/MSM.910692. PubMed PMID: 29877315; PubMed Central

PMCID: PMC6020746.

40: Bartoletti M, Antonelli M, Bruno Blasi FA, Casagranda I, Chieregato A,

Fumagalli R, Girardis M, Pieralli F, Plebani M, Rossolini GM, Sartelli M, Viaggi

B, Viale P, Viscoli C, Pea F. Procalcitonin-guided antibiotic therapy: an expert

consensus. Clin Chem Lab Med. 2018 Jul 26;56(8):1223-1229. doi:

10.1515/cclm-2018-0259. PubMed PMID: 29874192.

41: van der Does Y, Limper M, Jie KE, Schuit SCE, Jansen H, Pernot N, van

Rosmalen J, Poley MJ, Ramakers C, Patka P, van Gorp ECM, Rood PPM.

Procalcitonin-guided antibiotic therapy in patients with fever in a general

emergency department population: a multicentre non-inferiority randomized

clinical trial (HiTEMP study). Clin Microbiol Infect. 2018 Dec;24(12):1282-1289.

doi: 10.1016/j.cmi.2018.05.011. Epub 2018 Jun 2. PubMed PMID: 29870855.

42: Murri R, Mastrorosa I, Taccari F, Baroni S, Giovannenze F, Palazzolo C, Lardo

S, Scoppettuolo G, Ventura G, Cauda R, Fantoni M. Procalcitonin is useful in

driving the choice of early antibiotic treatment in patients with bloodstream

infections. Eur Rev Med Pharmacol Sci. 2018 May;22(10):3130-3137. doi:

10.26355/eurrev_201805_15072. PubMed PMID: 29863258.

43: Bilgin H, Haliloglu M, Yaman A, Ay P, Bilgili B, Arslantas MK, Ture Ozdemir

F, Haklar G, Cinel I, Mulazimoglu L. Sequential Measurements of Pentraxin 3 Serum

Levels in Patients with Ventilator-Associated Pneumonia: A Nested Case-Control

Study. Can J Infect Dis Med Microbiol. 2018 May 13;2018:4074169. doi:

10.1155/2018/4074169. eCollection 2018. PubMed PMID: 29861799; PubMed Central

PMCID: PMC5971295.

44: Nie S, Lu X, Jin Z, Gao J, Ma D, Deng J, Wu X, Hu YW, Zheng L, Wang Q.

Characterization of group B Streptococcus isolated from sterile and non-sterile

specimens in China. Diagn Microbiol Infect Dis. 2018 Sep;92(1):56-61. doi:

10.1016/j.diagmicrobio.2018.04.014. Epub 2018 May 16. PubMed PMID: 29858112.

45: Gautam-Goyal P, Malhotra P, Epstein M, Luka A, Schwartz RM, Burshtein A,

Burshtein J, Bensam B, Sy H, Ledoux D. Can Procalcitonin Be Used as a Reliable

Marker for Infectious Fever in Patients with Intracranial Hemorrhage? World

Neurosurg. 2018 Aug;116:e968-e974. doi: 10.1016/j.wneu.2018.05.142. Epub 2018 May

29. PubMed PMID: 29857212.

46: Titova E, Christensen A, Henriksen AH, Steinshamn S, Åsberg A. Comparison of

procalcitonin, C-reactive protein, white blood cell count and clinical status in

diagnosing pneumonia in patients hospitalized with acute exacerbations of COPD: A

prospective observational study. Chron Respir Dis. 2019

Jan-Dec;16:1479972318769762. doi: 10.1177/1479972318769762. Epub 2018 May 30.

PubMed PMID: 29848051; PubMed Central PMCID: PMC6302976.

47: Tang JH, Gao DP, Zou PF. Comparison of serum PCT and CRP levels in patients

infected by different pathogenic microorganisms: a systematic review and

meta-analysis. Braz J Med Biol Res. 2018;51(7):e6783. doi:

10.1590/1414-431x20176783. Epub 2018 May 28. Review. PubMed PMID: 29846409;

PubMed Central PMCID: PMC5995041.

48: Boran ÖF, Yazar FM, Boran M, Urfalıoğlu A, Bakacak Z, Yıldız M, Gül F, Güler

S. The Preseptic Period and Inflammatory Markers in the Prediction of the Course

of Sepsis. Med Sci Monit. 2018 May 27;24:3531-3539. doi: 10.12659/MSM.907687.

PubMed PMID: 29804126; PubMed Central PMCID: PMC5998727.

49: Fontela PS, O'Donnell S, Papenburg J. Can biomarkers improve the rational use

of antibiotics? Curr Opin Infect Dis. 2018 Aug;31(4):347-352. doi:

10.1097/QCO.0000000000000467. PubMed PMID: 29794541.

50: Pal E, Korva M, Resman Rus K, Kejžar N, Bogovič P, Kurent A, Avšič-Županc T,

Strle F. Sequential assessment of clinical and laboratory parameters in patients

with hemorrhagic fever with renal syndrome. PLoS One. 2018 May 23;13(5):e0197661.

doi: 10.1371/journal.pone.0197661. eCollection 2018. PubMed PMID: 29791494;

PubMed Central PMCID: PMC5965875.

51: Fujii Y, Yashiro M, Yamada M, Kikkawa T, Nosaka N, Saito Y, Tsukahara K,

Ikeda M, Morishima T, Tsukahara H. Serum Procalcitonin Levels in Acute

Encephalopathy with Biphasic Seizures and Late Reduced Diffusion. Dis Markers.

2018 Mar 14;2018:2380179. doi: 10.1155/2018/2380179. eCollection 2018. PubMed

PMID: 29725488; PubMed Central PMCID: PMC5872605.

52: Demir NA, Sumer S, Celik G, Afsar RE, Demir LS, Ural O. How should

procalcitonin and C-reactive protein levels be interpreted in haemodialysis

patients? Intern Med J. 2018 Oct;48(10):1222-1228. doi: 10.1111/imj.13952. PubMed

PMID: 29717808.

53: Korkmaz P, Koçak H, Onbaşı K, Biçici P, Özmen A, Uyar C, Özatağ DM. The Role

of Serum Procalcitonin, Interleukin-6, and Fibrinogen Levels in Differential

Diagnosis of Diabetic Foot Ulcer Infection. J Diabetes Res. 2018 Feb

21;2018:7104352. doi: 10.1155/2018/7104352. eCollection 2018. PubMed PMID:

29675434; PubMed Central PMCID: PMC5841040.

54: Quadir AF, Britton PN. Procalcitonin and C-reactive protein as biomarkers for

neonatal bacterial infection. J Paediatr Child Health. 2018 Jun;54(6):695-699.

doi: 10.1111/jpc.13931. Epub 2018 Apr 17. PubMed PMID: 29667256.

55: Tang J, Zhu H, Cai L, Tang T, Tang J, Sun Y, Liu M, Dai K, Qiao Z, Yu C.

Postoperative infection caused by Acinetobacter baumannii misdiagnosed as a

free-living amoeba species in a humeral head hemiarthroplasty patient: a case

report. Infect Dis Poverty. 2018 Mar 31;7(1):33. doi: 10.1186/s40249-018-0408-5.

PubMed PMID: 29631621; PubMed Central PMCID: PMC5890356.

56: Kumar N, Dayal R, Singh P, Pathak S, Pooniya V, Goyal A, Kamal R, Mohanty KK.

A Comparative Evaluation of Presepsin with Procalcitonin and CRP in Diagnosing

Neonatal Sepsis. Indian J Pediatr. 2019 Feb;86(2):177-179. doi:

10.1007/s12098-018-2659-3. Epub 2018 Mar 26. PubMed PMID: 29577181.

57: Pravin Charles MV, Kalaivani R, Venkatesh S, Kali A, Seetha KS. Evaluation of

procalcitonin as a diagnostic marker in neonatal sepsis. Indian J Pathol

Microbiol. 2018 Jan-Mar;61(1):81-84. doi: 10.4103/IJPM.IJPM_820_16. PubMed PMID:

29567889.

58: Garcia S, Echevarri J, Arana-Arri E, Sota M, Benito J, Mintegi S; Meningitis

group of RISEUP-SPERG. Outpatient management of children at low risk for

bacterial meningitis. Emerg Med J. 2018 Jun;35(6):361-366. doi:

10.1136/emermed-2017-206834. Epub 2018 Mar 21. PubMed PMID: 29563150.

59: Fuchs A, Gotta V, Decker ML, Szinnai G, Baumann P, Bonhoeffer J, Ritz N;

ProPAED study group. Cytokine kinetic profiles in children with acute lower

respiratory tract infection: a post hoc descriptive analysis from a randomized

control trial. Clin Microbiol Infect. 2018 Dec;24(12):1341.e1-1341.e7. doi:

10.1016/j.cmi.2018.03.016. Epub 2018 Mar 17. PubMed PMID: 29555393.

60: Sharma S, Uppal B, Manchanda V, Sud A. Diagnostic utility of procalcitonin as

biomarker of sepsis in children. Infect Dis (Lond). 2018 Jul;50(7):567-568. doi:

10.1080/23744235.2018.1453170. Epub 2018 Mar 19. PubMed PMID: 29553856.

61: Demirdal T, Sen P, Nemli SA. Diagnostic Value of Procalcitonin in Predicting

Bacteremia in Intensive Care Unit. Indian J Crit Care Med. 2018 Feb;22(2):78-84.

doi: 10.4103/ijccm.IJCCM_437_17. PubMed PMID: 29531446; PubMed Central PMCID:

PMC5842461.

62: Ulubay G, Ayvazoglu Soy E, Serifoglu I, Sozen F, Moray G, Haberal M. Utility

of Mean Platelet Volume to Diagnose Pneumonia in Patients With Solid-Organ

Transplant. Exp Clin Transplant. 2018 Mar;16 Suppl 1(Suppl 1):183-188. doi:

10.6002/ect.TOND-TDTD2017.P58. PubMed PMID: 29528024.

63: Shafiq N, Gautam V, Pandey AK, Kaur N, Garg S, Negi H, Kaur S, Ray P,

Malhotra S. A meta-analysis to assess usefulness of procalcitonin-guided

antibiotic usage for decision making. Indian J Med Res. 2017 Nov;146(5):576-584.

doi: 10.4103/ijmr.IJMR_613_15. Review. PubMed PMID: 29512600; PubMed Central

PMCID: PMC5861469.

64: Wongsurakiat P, Tulatamakit S. Clinical pulmonary infection score and a spot

serum procalcitonin level to guide discontinuation of antibiotics in

ventilator-associated pneumonia: a study in a single institution with high

prevalence of nonfermentative gram-negative bacilli infection. Ther Adv Respir

Dis. 2018 Jan-Dec;12:1753466618760134. doi: 10.1177/1753466618760134. PubMed

PMID: 29506460; PubMed Central PMCID: PMC5941665.

65: Levin PD, Cohen MJ, Ohev-Zion E, Tannus S, Stohl S, Avidan A, Cohen-Poraduso

R, Moses AE, Sprung CL, Benenson S. The Effect of Repeated Versus Initial

Procalcitonin Measurements on Diagnosis of Infection in the Intensive Care

Setting: A Prospective Observational Study. Anesth Analg. 2018 Mar 1. doi:

10.1213/ANE.0000000000003313. [Epub ahead of print] PubMed PMID: 29505445.

66: Dymova OV, Eremenko AA, Nikoda VV, Vasilieva ON, Bondarenko AV, Bogomolova

NS, Mikhailov YE. [DIAGNOSTIC VALUE OF THE HOMEOSTASIS PARAMETERS IN SEPSIS

DETECTING IN PATIENTS WITH INFECTIOUS-INFLAMMATORY COMPLICATIONS DURING THE

POSTOPERATIVE PERIOD.]. Anesteziol Reanimatol. 2017 Sep;61:196-201. Russian.

PubMed PMID: 29465204.

67: Brenner T, Decker SO, Grumaz S, Stevens P, Bruckner T, Schmoch T, Pletz MW,

Bracht H, Hofer S, Marx G, Weigand MA, Sohn K; TIFOnet Critical Care Trials

Group. Next-generation sequencing diagnostics of bacteremia in sepsis (Next

GeneSiS-Trial): Study protocol of a prospective, observational,

noninterventional, multicenter, clinical trial. Medicine (Baltimore). 2018

Feb;97(6):e9868. doi: 10.1097/MD.0000000000009868. PubMed PMID: 29419698; PubMed

Central PMCID: PMC5944698.

68: Strenger V, Merth G, Lackner H, Aberle SW, Kessler HH, Seidel MG, Schwinger

W, Sperl D, Sovinz P, Karastaneva A, Benesch M, Urban C. Malignancy and

chemotherapy induced haemophagocytic lymphohistiocytosis in children and

adolescents-a single centre experience of 20 years. Ann Hematol. 2018

Jun;97(6):989-998. doi: 10.1007/s00277-018-3254-4. Epub 2018 Feb 6. PubMed PMID:

29411124; PubMed Central PMCID: PMC5910490.

69: Bremmer DN, DiSilvio BE, Hammer C, Beg M, Vishwanathan S, Speredelozzi D,

Moffa MA, Hu K, Abdulmassih R, Makadia JT, Sandhu R, Naddour M, Chan-Tompkins NH,

Trienski TL, Watson C, Obringer TJ, Kuzyck J, Walsh TL. Impact of Procalcitonin

Guidance on Management of Adults Hospitalized with Chronic Obstructive Pulmonary

Disease Exacerbations. J Gen Intern Med. 2018 May;33(5):692-697. doi:

10.1007/s11606-018-4312-2. Epub 2018 Feb 5. PubMed PMID: 29404940; PubMed Central

PMCID: PMC5910348.

70: Tekerek NU, Akyildiz BN, Ercal BD, Muhtaroglu S. New Biomarkers to Diagnose

Ventilator Associated Pneumonia: Pentraxin 3 and Surfactant Protein D. Indian J

Pediatr. 2018 Jun;85(6):426-432. doi: 10.1007/s12098-018-2607-2. Epub 2018 Feb 2.

PubMed PMID: 29396775.

71: Abers MS, Musher DM. Procalcitonin as a Marker of Etiology in

Community-Acquired Pneumonia. Clin Infect Dis. 2018 May 2;66(10):1639. doi:

10.1093/cid/cix1089. PubMed PMID: 29360957.

72: Robinson P, De SK. How to use… Procalcitonin. Arch Dis Child Educ Pract Ed.

2018 Oct;103(5):257-262. doi: 10.1136/archdischild-2017-313699. Epub 2018 Jan 17.

PubMed PMID: 29343506.

73: Bozlu G, Tanriverdi H, Aslan G, Kuyucu N. The value of acute phase reactants

and LightCycler® SeptiFast test in the diagnosis of bacterial and viral

infections in pediatric patients. Arch Argent Pediatr. 2018 Feb 1;116(1):35-41.

doi: 10.5546/aap.2018.eng.35. English, Spanish. PubMed PMID: 29333810.

74: Nielsen SK, Lange P. [Not all exacerbations of chronic obstructive pulmonary

disease should be treated with antibiotics]. Ugeskr Laeger. 2017 Apr 10;179(15).

pii: V01170003. Review. Danish. PubMed PMID: 29332624.

75: Hamo Z, Azrad M, Nitzan O, Sagie A, Tkhawkho L, Binyamin D, Peretz A. Role of

Single Procalcitonin Test on Admission as a Biomarker for Predicting the Severity

of Clostridium difficile Infection. Front Microbiol. 2017 Dec 19;8:2532. doi:

10.3389/fmicb.2017.02532. eCollection 2017. PubMed PMID: 29312224; PubMed Central

PMCID: PMC5742163.

76: Lippi G, Sanchis-Gomar F. Procalcitonin in inflammatory bowel disease:

Drawbacks and opportunities. World J Gastroenterol. 2017 Dec 21;23(47):8283-8290.

doi: 10.3748/wjg.v23.i47.8283. Review. PubMed PMID: 29307988; PubMed Central

PMCID: PMC5743499.

77: K M, Batra P, Faridi MMA, Singh NP. Procalcitonin as Predictor of Bacterial

Infection in Meconium Aspiration Syndrome. Am J Perinatol. 2018

Jul;35(8):769-773. doi: 10.1055/s-0037-1615793. Epub 2017 Dec 29. PubMed PMID:

29287292.

78: Stover KR, Kenney RM, King ST, Gross AE. Evaluation of the Use of Novel

Biomarkers to Augment Antimicrobial Stewardship Program Activities.

Pharmacotherapy. 2018 Feb;38(2):271-283. doi: 10.1002/phar.2069. Epub 2018 Jan

16. Review. PubMed PMID: 29245184.

79: Memar MY, Varshochi M, Shokouhi B, Asgharzadeh M, Kafil HS. Procalcitonin:

The marker of pediatric bacterial infection. Biomed Pharmacother. 2017

Dec;96:936-943. doi: 10.1016/j.biopha.2017.11.149. Epub 2017 Dec 6. Review.

PubMed PMID: 29203386.

80: Jain V, Hussain NAFA, Siddiqui T, Sahu C, Ghar M, Prasad KN. Simultaneous

isolation of Chryseobacterium gleum from bloodstream and respiratory tract: first

case report from India. JMM Case Rep. 2017 Oct 16;4(10):e005122. doi:

10.1099/jmmcr.0.005122. eCollection 2017 Oct. PubMed PMID: 29188069; PubMed

Central PMCID: PMC5692238.

81: Hackner K, Riegler W, Handzhiev S, Bauer R, Veres J, Speiser M, Meisinger K,

Errhalt P. Fever after bronchoscopy: serum procalcitonin enables early diagnosis

of post-interventional bacterial infection. BMC Pulm Med. 2017 Nov 28;17(1):156.

doi: 10.1186/s12890-017-0508-1. PubMed PMID: 29179755; PubMed Central PMCID:

PMC5704393.

82: Chen Y, Wang Y, Jiang ZH, Xu LP, Zhang XH, Chen H, Chen YY, Wang FR, Wang JZ,

Han W, Zhang YY, Han TT, Tang FF, Mo XD, Sun YQ, Yan CH, Liu KY, Huan XJ.

[Analysis of risk factors related to the prognosis in patients with late-onset

severe pneumonia after allogeneic hematopoietic stem cell transplantation].

Zhonghua Nei Ke Za Zhi. 2017 Nov 1;56(11):804-809. doi:

10.3760/cma.j.issn.0578-1426.2017.11.006. Chinese. PubMed PMID: 29136708.

83: Tan Y, Zhou K, Tang X, Kudinha T, Wang L, Guo Z, Akova M, Zhuo C. Bacteremic

and non-bacteremic pneumonia caused by Acinetobacter baumannii in ICUs of South

China: A Clinical and Microbiological Study. Sci Rep. 2017 Nov 10;7(1):15279.

doi: 10.1038/s41598-017-13148-y. PubMed PMID: 29127419; PubMed Central PMCID:

PMC5681499.

84: Bruyndonckx R, Stuart B, Little P, Hens N, Ieven M, Butler CC, Verheij T,

Goossens H, Coenen S; GRACE project group. Amoxicillin for acute lower

respiratory tract infection in primary care: subgroup analysis by bacterial and

viral aetiology. Clin Microbiol Infect. 2018 Aug;24(8):871-876. doi:

10.1016/j.cmi.2017.10.032. Epub 2017 Nov 3. PubMed PMID: 29108950.

85: Trippella G, Galli L, De Martino M, Lisi C, Chiappini E. Procalcitonin

performance in detecting serious and invasive bacterial infections in children

with fever without apparent source: a systematic review and meta-analysis. Expert

Rev Anti Infect Ther. 2017 Nov;15(11):1041-1057. doi:

10.1080/14787210.2017.1400907. Epub 2017 Nov 15. Review. PubMed PMID: 29103336.

86: Ding X, Yang D, Ke C, Gong L, Zhan H, Yan R, Chen Y, Li H, Wang J. Value of

evaluating procalcitonin kinetics in diagnosis of infections in patients

undergoing laparoscopic radical cystectomy. Medicine (Baltimore). 2017

Oct;96(42):e8152. doi: 10.1097/MD.0000000000008152. PubMed PMID: 29049197; PubMed

Central PMCID: PMC5662363.

87: Dolin HH, Papadimos TJ, Stepkowski S, Chen X, Pan ZK. A Novel Combination of

Biomarkers to Herald the Onset of Sepsis Prior to the Manifestation of Symptoms.

Shock. 2018 Apr;49(4):364-370. doi: 10.1097/SHK.0000000000001010. PubMed PMID:

29016484; PubMed Central PMCID: PMC5811232.

88: Raineri SM, Cortegiani A, Vitale F, Iozzo P, Giarratano A. Procalcitonin for

the diagnosis of invasive candidiasis: what is the evidence? J Intensive Care.

2017 Sep 25;5:58. doi: 10.1186/s40560-017-0252-x. eCollection 2017. PubMed PMID:

28975031; PubMed Central PMCID: PMC5613326.

89: Walsh TL, DiSilvio BE, Hammer C, Beg M, Vishwanathan S, Speredelozzi D, Moffa

MA, Hu K, Abdulmassih R, Makadia JT, Sandhu R, Naddour M, Chan-Tompkins NH,

Trienski TL, Watson C, Obringer TJ, Kuzyck J, Bremmer DN. Impact of Procalcitonin

Guidance with an Educational Program on Management of Adults Hospitalized with

Pneumonia. Am J Med. 2018 Feb;131(2):201.e1-201.e8. doi:

10.1016/j.amjmed.2017.08.039. Epub 2017 Sep 22. PubMed PMID: 28947168.

90: Duployez C, Vachée A, Robineau O, Giraud F, Deny A, Senneville E, Wallet F,

Loïez C. Familial Transmission of emm12 Group A Streptococcus. Emerg Infect Dis.

2017 Oct;23(10):1745-1746. doi: 10.3201/eid2310.170343. PubMed PMID: 28930023;

PubMed Central PMCID: PMC5621563.

91: Tonkin-Crine SK, Tan PS, van Hecke O, Wang K, Roberts NW, McCullough A,

Hansen MP, Butler CC, Del Mar CB. Clinician-targeted interventions to influence

antibiotic prescribing behaviour for acute respiratory infections in primary

care: an overview of systematic reviews. Cochrane Database Syst Rev. 2017 Sep

7;9:CD012252. doi: 10.1002/14651858.CD012252.pub2. Review. PubMed PMID: 28881002.

92: Shokouhi B, Bookani KR, Ghasemi H, Khalouei M, Rezaei NJ, Samani SM.

Diagnostic and prognostic performances of serum procalcitonin in patients with

bloodstream infections: A parallel, case-control study comprising adults and

elderly. Rev Assoc Med Bras (1992). 2017 Jun;63(6):521-526. doi:

10.1590/1806-9282.63.06.521. PubMed PMID: 28876428.

93: Sanaei Dashti A, Alizadeh S, Karimi A, Khalifeh M, Shoja SA. Diagnostic value

of lactate, procalcitonin, ferritin, serum-C-reactive protein, and other

biomarkers in bacterial and viral meningitis: A cross-sectional study. Medicine

(Baltimore). 2017 Sep;96(35):e7637. doi: 10.1097/MD.0000000000007637. PubMed

PMID: 28858084; PubMed Central PMCID: PMC5585478.

94: Peng HL, Hu Y, Chen HJ, Song PP, Jiang L. Risk factors for poor prognosis in

children with refractory purulent meningitis and the discharge criteria. J Infect

Public Health. 2018 Mar - Apr;11(2):238-242. doi: 10.1016/j.jiph.2017.07.007.

Epub 2017 Aug 7. PubMed PMID: 28797539.

95: Dharmayanti A, Astrawinata D. Ventilator-Associated Pneumonia (VAP) in a

Patient with Guillain-Barre Syndrome. Acta Med Indones. 2017 Apr;49(2):151-157.

PubMed PMID: 28790230.

96: Gilbert DN. Role of Procalcitonin in the Management of Infected Patients in

the Intensive Care Unit. Infect Dis Clin North Am. 2017 Sep;31(3):435-453. doi:

10.1016/j.idc.2017.05.003. Review. PubMed PMID: 28779830.

97: Hamada Imam M, Gamal E. Procalcitonin level as a surrogate for

catheter-related blood stream infection among hemodialysis patients. J Vasc

Access. 2017 Nov 17;18(6):498-502. doi: 10.5301/jva.5000765. Epub 2017 Jul 26.

PubMed PMID: 28777420.

98: Markota A, Sinkovič A. Mortality, intensive care treatment, and cost

evaluation: Role of a polymerase chain reaction assay in patients with sepsis. J

Int Med Res. 2018 Jan;46(1):79-88. doi: 10.1177/0300060517719768. Epub 2017 Jul

21. PubMed PMID: 28730906; PubMed Central PMCID: PMC6011302.

99: Afzal Z, Kallumadanda S, Wang F, Hemmige V, Musher D. Acute Febrile Illness

and Complications Due to Murine Typhus, Texas, USA1,2. Emerg Infect Dis. 2017

Aug;23(8):1268-1273. doi: 10.3201/eid2308.161861. PubMed PMID: 28726607; PubMed

Central PMCID: PMC5547806.

100: Bénet T, Picot VS, Awasthi S, Pandey N, Bavdekar A, Kawade A, Robinson A,

Rakoto-Andrianarivelo M, Sylla M, Diallo S, Russomando G, Basualdo W,

Komurian-Pradel F, Endtz H, Vanhems P, Paranhos-Baccalà G, For The Gabriel

Network. Severity of Pneumonia in Under 5-Year-Old Children from Developing

Countries: A Multicenter, Prospective, Observational Study. Am J Trop Med Hyg.

2017 Jul;97(1):68-76. doi: 10.4269/ajtmh.16-0733. PubMed PMID: 28719310; PubMed

Central PMCID: PMC5508893.

101: Giacobbe DR, Mikulska M, Tumbarello M, Furfaro E, Spadaro M, Losito AR,

Mesini A, De Pascale G, Marchese A, Bruzzone M, Pelosi P, Mussap M, Molin A,

Antonelli M, Posteraro B, Sanguinetti M, Viscoli C, Del Bono V; ISGRI-SITA

(Italian Study Group on Resistant Infections of the Società Italiana Terapia

Antinfettiva). Combined use of serum (1,3)-β-D-glucan and procalcitonin for the

early differential diagnosis between candidaemia and bacteraemia in intensive

care units. Crit Care. 2017 Jul 10;21(1):176. doi: 10.1186/s13054-017-1763-5.

PubMed PMID: 28693606; PubMed Central PMCID: PMC5504626.

102: Wu RX, Chiu CC, Lin TC, Yang YS, Lee Y, Lin JC, Chang FY. Procalcitonin as a

diagnostic biomarker for septic shock and bloodstream infection in burn patients

from the Formosa Fun Coast dust explosion. J Microbiol Immunol Infect. 2017

Dec;50(6):872-878. doi: 10.1016/j.jmii.2016.08.021. Epub 2017 Jun 22. PubMed

PMID: 28690030.

103: Mizutani M, Hasegawa S, Matsushige T, Ohta N, Kittaka S, Hoshide M, Kusuda

T, Takahashi K, Ichihara K, Ohga S. Distinctive inflammatory profile between

acute focal bacterial nephritis and acute pyelonephritis in children. Cytokine.

2017 Nov;99:24-29. doi: 10.1016/j.cyto.2017.06.012. Epub 2017 Jul 3. PubMed PMID:

28683358.

104: Irwin AD, Grant A, Williams R, Kolamunnage-Dona R, Drew RJ, Paulus S,

Jeffers G, Williams K, Breen R, Preston J, Appelbe D, Chesters C, Newland P,

Marzouk O, McNamara PS, Diggle PJ, Carrol ED. Predicting Risk of Serious

Bacterial Infections in Febrile Children in the Emergency Department. Pediatrics.

2017 Aug;140(2). pii: e20162853. doi: 10.1542/peds.2016-2853. Epub 2017 Jul 5.

PubMed PMID: 28679639.

105: De Pascale G, Martucci G, Montini L, Panarello G, Cutuli SL, Di Carlo D, Di

Gravio V, Di Stefano R, Capitanio G, Vallecoccia MS, Polidori P, Spanu T,

Arcadipane A, Antonelli M. Double carbapenem as a rescue strategy for the

treatment of severe carbapenemase-producing Klebsiella pneumoniae infections: a

two-center, matched case-control study. Crit Care. 2017 Jul 5;21(1):173. doi:

10.1186/s13054-017-1769-z. PubMed PMID: 28679413; PubMed Central PMCID:

PMC5498909.

106: Matera G, Quirino A, Peronace C, Settembre P, Marano V, Loria MT, Marascio

N, Galati L, Barreca GS, Giancotti A, Amantea B, Liberto MC, Focà A. Soluble CD14

Subtype-A New Biomarker in Predicting the Outcome of Critically Ill Septic

Patients. Am J Med Sci. 2017 Jun;353(6):543-551. doi:

10.1016/j.amjms.2017.03.036. Epub 2017 Apr 7. PubMed PMID: 28641717.

107: Fung AWS, Beriault D, Diamandis EP, Burnham CD, Dorman T, Downing M, Hayden

J, Langford BJ. The Role of Procalcitonin in Diagnosis of Sepsis and Antibiotic

Stewardship: Opportunities and Challenges. Clin Chem. 2017 Sep;63(9):1436-1441.

doi: 10.1373/clinchem.2017.272294. Epub 2017 Jun 20. PubMed PMID: 28634222.

108: Kawamatawong T, Apiwattanaporn A, Siricharoonwong W. Serum inflammatory

biomarkers and clinical outcomes of COPD exacerbation caused by different

pathogens. Int J Chron Obstruct Pulmon Dis. 2017 May 31;12:1625-1630. doi:

10.2147/COPD.S132132. eCollection 2017. PubMed PMID: 28615935; PubMed Central

PMCID: PMC5459973.

109: Wang XJ, Tan TT, Lim ST, Farid M, Tao M, Quek R, Chan A, Tang T. Role of

Procalcitonin in Differentiating between Infectious and Noninfectious Fevers

among Patients with Lymphoma. Pharmacotherapy. 2017 Aug;37(8):908-915. doi:

10.1002/phar.1963. Epub 2017 Jul 18. PubMed PMID: 28556122.

110: Shapiro DS, Friedmann R, Husseini A, Ivgi H, Yinnon AM, Assous MV. Can

Procalcitonin Contribute to the Diagnosis of Clostridium difficile Colitis? Isr

Med Assoc J. 2017 May;19(5):313-316. PubMed PMID: 28513121.

111: Itenov TS, Jensen JU, Ostrowski SR, Johansson PI, Thormar KM, Lundgren JD,

Bestle MH; “Procalcitonin And Survival Study” study group. Endothelial Damage

Signals Refractory Acute Kidney Injury in Critically Ill Patients. Shock. 2017

Jun;47(6):696-701. doi: 10.1097/SHK.0000000000000804. PubMed PMID: 28505627.

112: R Nath S, Jayapalan S, Nair H, Kusumakumary P, Prema NS, Priyakumari T,

Rajamohanan K. Comparative diagnostic test evaluation of serum procalcitonin and

C-reactive protein in suspected bloodstream infections in children with cancer. J

Med Microbiol. 2017 May;66(5):622-627. doi: 10.1099/jmm.0.000478. Epub 2017 May

15. PubMed PMID: 28504925.

113: Póvoa P, Martin-Loeches I, Ramirez P, Bos LD, Esperatti M, Silvestre J, Gili

G, Goma G, Berlanga E, Espasa M, Gonçalves E, Torres A, Artigas A. Biomarkers

kinetics in the assessment of ventilator-associated pneumonia response to

antibiotics - results from the BioVAP study. J Crit Care. 2017 Oct;41:91-97. doi:

10.1016/j.jcrc.2017.05.007. Epub 2017 May 8. PubMed PMID: 28502892.

114: Karampela I, Kandri E, Antonakos G, Vogiatzakis E, Christodoulatos GS,

Nikolaidou A, Dimopoulos G, Armaganidis A, Dalamaga M. Kinetics of circulating

fetuin-A may predict mortality independently from adiponectin, high molecular

weight adiponectin and prognostic factors in critically ill patients with sepsis:

A prospective study. J Crit Care. 2017 Oct;41:78-85. doi:

10.1016/j.jcrc.2017.05.004. Epub 2017 May 6. PubMed PMID: 28500919.

115: Cazzola M, Rogliani P, Aliberti S, Blasi F, Matera MG. An update on the

pharmacotherapeutic management of lower respiratory tract infections. Expert Opin

Pharmacother. 2017 Jul;18(10):973-988. doi: 10.1080/14656566.2017.1328497. Epub

2017 May 19. Review. PubMed PMID: 28480770.

116: Alcoba G, Keitel K, Maspoli V, Lacroix L, Manzano S, Gehri M, Tabin R,

Gervaix A, Galetto-Lacour A. A three-step diagnosis of pediatric pneumonia at the

emergency department using clinical predictors, C-reactive protein, and

pneumococcal PCR. Eur J Pediatr. 2017 Jun;176(6):815-824. doi:

10.1007/s00431-017-2913-0. Epub 2017 May 4. PubMed PMID: 28474099.

117: Wu D, Zhou S, Hu S, Liu B. Inflammatory responses and histopathological

changes in a mouse model of Staphylococcus aureus-induced bloodstream infections.

J Infect Dev Ctries. 2017 Apr 30;11(4):294-305. doi: 10.3855/jidc.7800. PubMed

PMID: 28459220.

118: Yu Y, Li HJ. Diagnostic and prognostic value of procalcitonin for early

intracranial infection after craniotomy. Braz J Med Biol Res. 2017 Apr

20;50(5):e6021. doi: 10.1590/1414-431X20176021. PubMed PMID: 28443989; PubMed

Central PMCID: PMC5441286.

119: Aydin M, Barut S, Akbulut HH, Ucar S, Orman A. Application of Flow Cytometry

in the Early Diagnosis of Neonatal Sepsis. Ann Clin Lab Sci. 2017

Mar;47(2):184-190. PubMed PMID: 28442521.

120: Pontrelli G, De Crescenzo F, Buzzetti R, Jenkner A, Balduzzi S, Calò

Carducci F, Amodio D, De Luca M, Chiurchiù S, Davies EH, Copponi G, Simonetti A,

Ferretti E, Di Franco V, Rasi V, Della Corte M, Gramatica L, Ciabattini M,

Livadiotti S, Rossi P. Accuracy of serum procalcitonin for the diagnosis of

sepsis in neonates and children with systemic inflammatory syndrome: a

meta-analysis. BMC Infect Dis. 2017 Apr 24;17(1):302. doi:

10.1186/s12879-017-2396-7. Review. PubMed PMID: 28438138; PubMed Central PMCID:

PMC5404674.

121: Masiá M, Padilla S, Ortiz de la Tabla V, González M, Bas C, Gutiérrez F.

Procalcitonin for selecting the antibiotic regimen in outpatients with low-risk

community-acquired pneumonia using a rapid point-of-care testing: A single-arm

clinical trial. PLoS One. 2017 Apr 20;12(4):e0175634. doi:

10.1371/journal.pone.0175634. eCollection 2017. PubMed PMID: 28426811; PubMed

Central PMCID: PMC5398537.

122: Rebello A, Thabah MM, Dutta TK, Bobby Z, Harish BN, Mehalingam V.

Procalcitonin levels in sepsis and its association with clinical outcome in

southern India. Trop Doct. 2017 Oct;47(4):331-336. doi: 10.1177/0049475517702314.

Epub 2017 Apr 14. PubMed PMID: 28409531.

123: Self WH, Balk RA, Grijalva CG, Williams DJ, Zhu Y, Anderson EJ, Waterer GW,

Courtney DM, Bramley AM, Trabue C, Fakhran S, Blaschke AJ, Jain S, Edwards KM,

Wunderink RG. Procalcitonin as a Marker of Etiology in Adults Hospitalized With

Community-Acquired Pneumonia. Clin Infect Dis. 2017 Jul 15;65(2):183-190. doi:

10.1093/cid/cix317. PubMed PMID: 28407054; PubMed Central PMCID: PMC5850442.

124: Bilgin IA, Hatipoglu E, Aghayeva A, Arikan AE, Incir S, Mamal Torun M,

Dirican A, Erguney S. Predicting Value of Serum Procalcitonin, C-Reactive

Protein, Drain Fluid Culture, Drain Fluid Interleukin-6, and Tumor Necrosis

Factor-α Levels in Anastomotic Leakage after Rectal Resection. Surg Infect

(Larchmt). 2017 Apr;18(3):350-356. doi: 10.1089/sur.2016.222. Epub 2017 Feb 23.

PubMed PMID: 28394749.

125: Chauhan N, Tiwari S, Jain U. Potential biomarkers for effective screening of

neonatal sepsis infections: An overview. Microb Pathog. 2017 Jun;107:234-242.

doi: 10.1016/j.micpath.2017.03.042. Epub 2017 Apr 1. Review. PubMed PMID:

28377234.

126: Yumoto T, Kono Y, Kawano S, Kamoi C, Iida A, Nose M, Sato K, Ugawa T, Okada

H, Ujike Y, Nakao A. Citrobacter braakii bacteremia-induced septic shock after

colonoscopy preparation with polyethylene glycol in a critically ill patient: a

case report. Ann Clin Microbiol Antimicrob. 2017 Apr 4;16(1):22. doi:

10.1186/s12941-017-0201-5. PubMed PMID: 28376885; PubMed Central PMCID:

PMC5379765.

127: Ascione T, Balato G, Boccia G, De Caro F. Predictive value of fever

following arthroplasty in diagnosing an early infection. Infez Med. 2017 Mar

1;25(1):3-7. Review. PubMed PMID: 28353448.

128: Loonen AJM, Kesarsing C, Kusters R, Hilbink M, Wever PC, van den Brule AJC.

High pneumococcal DNA load, procalcitonin and suPAR levels correlate to severe

disease development in patients with pneumococcal pneumonia. Eur J Clin Microbiol

Infect Dis. 2017 Sep;36(9):1541-1547. doi: 10.1007/s10096-017-2963-2. Epub 2017

Mar 29. PubMed PMID: 28353184.

129: Casas-Alba D, de Sevilla MF, Valero-Rello A, Fortuny C, García-García JJ,

Ortez C, Muchart J, Armangué T, Jordan I, Luaces C, Barrabeig I, González-Sanz R,

Cabrerizo M, Muñoz-Almagro C, Launes C. Outbreak of brainstem encephalitis

associated with enterovirus-A71 in Catalonia, Spain (2016): a clinical

observational study in a children's reference centre in Catalonia. Clin Microbiol

Infect. 2017 Nov;23(11):874-881. doi: 10.1016/j.cmi.2017.03.016. Epub 2017 Mar

23. PubMed PMID: 28344164.

130: Villalobos Pinto E, Sánchez-Bayle M. [Construction of a diagnostic

prediction model of severe bacterial infection in febrile infants under 3 months

old]. An Pediatr (Barc). 2017 Dec;87(6):330-336. doi:

10.1016/j.anpedi.2017.02.003. Epub 2017 Mar 21. Spanish. PubMed PMID: 28341146.

131: Li YL, Zhai LC, Ji JH, Liu LY. Detection of combined procalcitonin and

c-reactive protein applied in the diagnosis of bacterial infections. J Biol Regul

Homeost Agents. 2017 Jan-Mar;31(1):177-181. PubMed PMID: 28337889.

132: Chu DC, Mehta AB, Walkey AJ. Practice Patterns and Outcomes Associated With

Procalcitonin Use in Critically Ill Patients With Sepsis. Clin Infect Dis. 2017

Jun 1;64(11):1509-1515. doi: 10.1093/cid/cix179. PubMed PMID: 28329238; PubMed

Central PMCID: PMC5434362.

133: Wan YM, Li YH, Xu ZY, Yang J, Yang LH, Xu Y, Yang JH. Therapeutic plasma

exchange versus double plasma molecular absorption system in hepatitis B

virus-infected acute-on-chronic liver failure treated by entercavir: A

prospective study. J Clin Apher. 2017 Dec;32(6):453-461. doi: 10.1002/jca.21535.

Epub 2017 Mar 17. PubMed PMID: 28304106.

134: Lehmann C, Berner R, Bogner JR, Cornely OA, de With K, Herold S, Kern WV,

Lemmen S, Pletz MW, Ruf B, Salzberger B, Stellbrink HJ, Suttorp N, Ullmann AJ,

Fätkenheuer G, Jung N. The "Choosing Wisely" initiative in infectious diseases.

Infection. 2017 Jun;45(3):263-268. doi: 10.1007/s15010-017-0997-0. Epub 2017 Mar

13. Review. PubMed PMID: 28290130.

135: Aksaray S, Alagoz P, Inan A, Cevan S, Ozgultekin A. Diagnostic value of

sTREM-1 and procalcitonin levels in the early diagnosis of sepsis. North Clin

Istanb. 2017 Jan 25;3(3):175-182. doi: 10.14744/nci.2016.26023. eCollection 2016.

PubMed PMID: 28275748; PubMed Central PMCID: PMC5336621.

136: Mallet M, Haq M, Tripon S, Bernard M, Benosman H, Thabut D, Rudler M.

Elevated procalcitonin is associated with bacterial infection during acute liver

failure only when unrelated to acetaminophen intoxication. Eur J Gastroenterol

Hepatol. 2017 Jul;29(7):811-816. doi: 10.1097/MEG.0000000000000862. PubMed PMID:

28272093.

137: Cho H, Lee ES, Lee YS, Kim YJ, Sohn CH, Ahn S, Seo DW, Lee JH, Kim WY, Lim

KS. Predictors of septic shock in initially stable patients with pyogenic liver

abscess. Scand J Gastroenterol. 2017 May;52(5):589-594. doi:

10.1080/00365521.2017.1288757. Epub 2017 Feb 15. PubMed PMID: 28270040.

138: Alvand A, Rezapoor M, Parvizi J. The Role of Biomarkers for the Diagnosis of

Implant-Related Infections in Orthopaedics and Trauma. Adv Exp Med Biol.

2017;971:69-79. doi: 10.1007/5584_2017_11. Review. PubMed PMID: 28243953.

139: Carannante N, Rossi M, Fraganza F, Coppola G, Chiesa D, Attanasio V, Sbrana

F, Corcione A, Tascini C. A high PCT level correlates with disease severity in

Plasmodium falciparum malaria in children. New Microbiol. 2017 Jan;40(1):72-74.

PubMed PMID: 28217817.

140: Caffarini EM, DeMott J, Patel G, Lat I. Determining the Clinical Utility of

an Absolute Procalcitonin Value for Predicting a Positive Culture Result.

Antimicrob Agents Chemother. 2017 Apr 24;61(5). pii: e02007-16. doi:

10.1128/AAC.02007-16. Print 2017 May. PubMed PMID: 28193661; PubMed Central

PMCID: PMC5404602.

141: Niño ME, Serrano SE, Niño DC, McCosham DM, Cardenas ME, Villareal VP, Lopez

M, Pazin-Filho A, Jaimes FA, Cunha F, Schulz R, Torres-Dueñas D. TIMP1 and MMP9

are predictors of mortality in septic patients in the emergency department and

intensive care unit unlike MMP9/TIMP1 ratio: Multivariate model. PLoS One. 2017

Feb 13;12(2):e0171191. doi: 10.1371/journal.pone.0171191. eCollection 2017.

PubMed PMID: 28192449; PubMed Central PMCID: PMC5305237.

142: Pan YP, Fang YP, Xu YH, Wang ZX, Shen JL. The Diagnostic Value of

Procalcitonin Versus Other Biomarkers in Prediction of Bloodstream Infection.

Clin Lab. 2017 Feb 1;63(2):277-285. doi: 10.7754/Clin.Lab.2016.160802. PubMed

PMID: 28182347.

143: Pieralli F, Corbo L, Torrigiani A, Mannini D, Antonielli E, Mancini A,

Corradi F, Arena F, Moggi Pignone A, Morettini A, Nozzoli C, Rossolini GM.

Usefulness of procalcitonin in differentiating Candida and bacterial blood stream

infections in critically ill septic patients outside the intensive care unit.

Intern Emerg Med. 2017 Aug;12(5):629-635. doi: 10.1007/s11739-017-1627-7. Epub

2017 Feb 4. PubMed PMID: 28161884.

144: Dixon G, Lama-Lopez A, Bintcliffe OJ, Morley AJ, Hooper CE, Maskell NA. The

role of serum procalcitonin in establishing the diagnosis and prognosis of

pleural infection. Respir Res. 2017 Feb 3;18(1):30. doi:

10.1186/s12931-017-0501-5. PubMed PMID: 28158976; PubMed Central PMCID:

PMC5291982.

145: Stockmann C, Ampofo K, Killpack J, Williams DJ, Edwards KM, Grijalva CG,

Arnold SR, McCullers JA, Anderson EJ, Wunderink RG, Self WH, Bramley A, Jain S,

Pavia AT, Blaschke AJ. Procalcitonin Accurately Identifies Hospitalized Children

With Low Risk of Bacterial Community-Acquired Pneumonia. J Pediatric Infect Dis

Soc. 2018 Feb 19;7(1):46-53. doi: 10.1093/jpids/piw091. PubMed PMID: 28158460;

PubMed Central PMCID: PMC6251689.

146: Mathioudakis AG, Chatzimavridou-Grigoriadou V, Corlateanu A, Vestbo J.

Procalcitonin to guide antibiotic administration in COPD exacerbations: a

meta-analysis. Eur Respir Rev. 2017 Jan 31;26(143). pii: 160073. doi:

10.1183/16000617.0073-2016. Print 2017 Jan. Review. PubMed PMID: 28143877.

147: Yeşilbaş O, Şevketoğlu E, Kıhtır HS, Talip Petmezci M, Bato E, Balkaya S,

Hatipoğlu N, Kuşkucu MA, Palabıyık F, Çakır E. [A case of bronchiolitis

obliterans secondary to human metapneumovirus bronchiolitis]. Mikrobiyol Bul.

2016 Oct;50(4):606-612. Turkish. PubMed PMID: 28124966.

148: Tan M, Wang J, Hu P, Wang B, Xu W, Chen J. Severe pneumonia due to infection

with Candida krusei in a case of suspected Middle East respiratory syndrome: A

case report and literature review. Exp Ther Med. 2016 Dec;12(6):4085-4088. doi:

10.3892/etm.2016.3892. Epub 2016 Nov 11. PubMed PMID: 28101187; PubMed Central

PMCID: PMC5228306.

149: Ratzinger F, Haslacher H, Stadlberger M, Schmidt RL, Obermüller M,

Schmetterer KG, Perkmann T, Makristathis A, Marculescu R, Burgmann H. 25(OH)D and

1,25(OH)D vitamin D fails to predict sepsis and mortality in a prospective cohort

study. Sci Rep. 2017 Jan 12;7:40646. doi: 10.1038/srep40646. PubMed PMID:

28079172; PubMed Central PMCID: PMC5228346.

150: Lang Y, Jiang Y, Gao M, Wang W, Wang N, Wang K, Zhang H, Chen G, Liu K, Liu

M, Yang M, Xiao X. Interleukin-1 Receptor 2: A New Biomarker for Sepsis Diagnosis

and Gram-Negative/Gram-Positive Bacterial Differentiation. Shock. 2017

Jan;47(1):119-124. PubMed PMID: 27984536.

151: Yan ST, Sun LC, Jia HB, Gao W, Yang JP, Zhang GQ. Procalcitonin levels in

bloodstream infections caused by different sources and species of bacteria. Am J

Emerg Med. 2017 Apr;35(4):579-583. doi: 10.1016/j.ajem.2016.12.017. Epub 2016 Dec

10. PubMed PMID: 27979420.

152: Yilmaz G, Salyan S, Aksoy F, Köksal İ. Individualized antibiotic therapy in

patients with ventilator-associated pneumonia. J Med Microbiol. 2017

Jan;66(1):78-82. doi: 10.1099/jmm.0.000401. Epub 2017 Feb 22. PubMed PMID:

27911257.

153: Li S, Rong H, Guo Q, Chen Y, Zhang G, Yang J. Serum procalcitonin levels

distinguish Gram-negative bacterial sepsis from Gram-positive bacterial and

fungal sepsis. J Res Med Sci. 2016 Jun 14;21:39. eCollection 2016. PubMed PMID:

27904585; PubMed Central PMCID: PMC5122113.

154: Papp M, Tornai T, Vitalis Z, Tornai I, Tornai D, Dinya T, Sumegi A,

Antal-Szalmas P. Presepsin teardown - pitfalls of biomarkers in the diagnosis and

prognosis of bacterial infection in cirrhosis. World J Gastroenterol. 2016 Nov

7;22(41):9172-9185. PubMed PMID: 27895404; PubMed Central PMCID: PMC5107598.

155: Ning J, Shao X, Ma Y, Lv D. Valuable hematological indicators for the

diagnosis and severity assessment of Chinese children with community-acquired

pneumonia: Prealbumin. Medicine (Baltimore). 2016 Nov;95(47):e5452. PubMed PMID:

27893691; PubMed Central PMCID: PMC5134884.

156: Gunasekaran V, Radhakrishnan N, Dinand V, Sachdeva A. Serum Procalcitonin

for Predicting Significant Infections and Mortality in Pediatric Oncology. Indian

Pediatr. 2016 Dec 15;53(12):1075-1078. Epub 2016 Nov 5. PubMed PMID: 27889712.

157: Jensen JS, Itenov TS, Thormar KM, Hein L, Mohr TT, Andersen MH, Løken J,

Tousi H, Lundgren B, Boesen HC, Johansen ME, Ostrowski SR, Johansson PI, Grarup

J, Vestbo J, Lundgren JD; Procalcitonin And Survival Study (PASS) Group.

Prediction of non-recovery from ventilator-demanding acute respiratory failure,

ARDS and death using lung damage biomarkers: data from a 1200-patient critical

care randomized trial. Ann Intensive Care. 2016 Dec;6(1):114. doi:

10.1186/s13613-016-0212-y. Epub 2016 Nov 21. PubMed PMID: 27873291; PubMed

Central PMCID: PMC5118375.

158: Arora R, Campbell JP, Simon G, Sahni N. Does serum procalcitonin aid in the

diagnosis of bloodstream infection regardless of whether patients exhibit the

systemic inflammatory response syndrome? Infection. 2017 Jun;45(3):291-298. doi:

10.1007/s15010-016-0965-0. Epub 2016 Nov 19. PubMed PMID: 27866368.

159: Ramirez P, Villarreal E, Gordon M, Gómez MD, de Hevia L, Vacacela K, Gisbert

T, Quinzá A, Ruiz J, Alonso R, Bonastre J, Vila J. Septic Participation in

Cardiogenic Shock: Exposure to Bacterial Endotoxin. Shock. 2017

May;47(5):588-592. doi: 10.1097/SHK.0000000000000798. PubMed PMID: 27861258.

160: Alons IM, Verheul RJ, Kuipers I, Jellema K, Wermer MJ, Algra A, Ponjee G.

Procalcitonin in cerebrospinal fluid in meningitis: a prospective diagnostic

study. Brain Behav. 2016 Aug 16;6(11):e00545. eCollection 2016 Nov. PubMed PMID:

27843698; PubMed Central PMCID: PMC5102643.

161: Liu J, Fan P, Chu J, Yang J, Yang X, Zhang L, Guo C. A contrast study of

Dermacyn on enterocoely irrigate to control intraoperative infection. Minerva

Chir. 2017 Apr;72(2):121-124. doi: 10.23736/S0026-4733.16.07192-3. Epub 2016 Nov

9. PubMed PMID: 27841850.

162: Tyagi A, Bansal A, Das S, Sethi AK, Kakkar A. Effect of thoracic epidural

block on infection-induced inflammatory response: A randomized controlled trial.

J Crit Care. 2017 Apr;38:6-12. doi: 10.1016/j.jcrc.2016.10.006. Epub 2016 Oct 18.

PubMed PMID: 27829181.

163: Rogina P, Stubljar D, Lejko Zupanc T, Ihan A, Skvarc M. Neutrophil CD64

molecule expression can predict bloodstream infection in septic shock patients.

Clin Chem Lab Med. 2017 May 1;55(6):e130-e132. doi: 10.1515/cclm-2016-0776.

PubMed PMID: 27816955.

164: Zawadzki M, Krzystek-Korpacka M, Gamian A, Witkiewicz W. Comparison of

inflammatory responses following robotic and open colorectal surgery: a

prospective study. Int J Colorectal Dis. 2017 Mar;32(3):399-407. doi:

10.1007/s00384-016-2697-0. Epub 2016 Nov 4. PubMed PMID: 27815698.

165: Savaş Bozbaş Ş, Er Dedekargınoğlu B, Ulubay G, Haberal M. Role of Serum

Procalcitonin Levels in Solid-Organ Transplant Patients. Exp Clin Transplant.

2016 Nov;14(Suppl 3):116-120. PubMed PMID: 27805529.

166: Akçay Ş, Doğrul I, Sezer S, Haberal M. Role of Procalcitonin in Differential

Diagnosis of Pneumonia and Pulmonary Congestion Associated With End-Stage Renal

Failure. Exp Clin Transplant. 2016 Nov;14(Suppl 3):71-73. PubMed PMID: 27805517.

167: Stoma I, Karpov I, Uss A, Rummo O, Milanovich N, Iskrov I. Diagnostic value

of sepsis biomarkers in hematopoietic stem cell transplant recipients in a

condition of high prevalence of gram-negative pathogens. Hematol Oncol Stem Cell

Ther. 2017 Mar;10(1):15-21. doi: 10.1016/j.hemonc.2016.09.002. Epub 2016 Oct 20.

PubMed PMID: 27793578.

168: Spoorenberg SM, Vestjens SM, Rijkers GT, Meek B, van Moorsel CH, Grutters

JC, Bos WJ; Ovidius Study Group. YKL-40, CCL18 and SP-D predict mortality in

patients hospitalized with community-acquired pneumonia. Respirology. 2017

Apr;22(3):542-550. doi: 10.1111/resp.12924. Epub 2016 Oct 26. PubMed PMID:

27782361.

169: Teepe J, Broekhuizen BDL, Loens K, Lammens C, Ieven M, Goossens H, Little P,

Butler CC, Coenen S, Godycki-Cwirko M, Verheij TJM; GRACE Consortium. Predicting

the presence of bacterial pathogens in the airways of primary care patients with

acute cough. CMAJ. 2017 Jan 16;189(2):E50-E55. doi: 10.1503/cmaj.151364. Epub

2016 Oct 24. PubMed PMID: 27777252; PubMed Central PMCID: PMC5235925.

170: Xia T, Xu X, Zhao N, Luo Z, Tang Y. Comparison of the diagnostic power of

cytokine patterns and procalcitonin for predicting infection among paediatric

haematology/oncology patients. Clin Microbiol Infect. 2016 Dec;22(12):996-1001.

doi: 10.1016/j.cmi.2016.09.013. Epub 2016 Sep 22. PubMed PMID: 27665705.

171: Şirinoğlu M, Soysal A, Karaaslan A, Kepenekli Kadayifci E, Yalındağ-Öztürk

N, Cinel İ, Yaman A, Haklar G, Şirikci Ö, Turan S, Altınkanat Gelmez G, Söyletir

G, Bakır M. The diagnostic value of soluble urokinase plasminogen activator

receptor (suPAR) compared to C-reactive protein (CRP) and procalcitonin (PCT) in

children with systemic inflammatory response syndrome (SIRS). J Infect Chemother.

2017 Jan;23(1):17-22. doi: 10.1016/j.jiac.2016.08.015. Epub 2016 Oct 19. PubMed

PMID: 27771157.

172: Watanabe Y, Oikawa N, Hariu M, Fuke R, Seki M. Ability of procalcitonin to

diagnose bacterial infection and bacteria types compared with blood culture

findings. Int J Gen Med. 2016 Sep 30;9:325-331. eCollection 2016. PubMed PMID:

27757046; PubMed Central PMCID: PMC5053384.

173: Gómez-Zorrilla S, Morandeira F, Castro MJ, Tubau F, Periche E, Cañizares R,

Dominguez MA, Ariza J, Peña C. Acute Inflammatory Response of Patients with

Pseudomonas aeruginosa Infections: A Prospective Study. Microb Drug Resist. 2017

Jun;23(4):523-530. doi: 10.1089/mdr.2016.0144. Epub 2016 Oct 18. PubMed PMID:

27754817; PubMed Central PMCID: PMC5488258.

174: Kumar P, Medigeshi GR, Mishra VS, Islam M, Randev S, Mukherjee A, Chaudhry

R, Kapil A, Ram Jat K, Lodha R, Kabra SK. Etiology of Acute Respiratory

Infections in Infants: A Prospective Birth Cohort Study. Pediatr Infect Dis J.

2017 Jan;36(1):25-30. PubMed PMID: 27753796.

175: Gao J, Zhang L, Zhang X, Zhao SL, Liu SY, Xing LH. Levels of serum

procalcitonin and C-reactive protein for evaluating pulmonary bacterial infection

in patients with lupus erythematosus. J Huazhong Univ Sci Technolog Med Sci. 2016

Oct;36(5):653-658. doi: 10.1007/s11596-016-1641-4. Epub 2016 Oct 18. PubMed PMID:

27752903.

176: Sharma SK, Gupta A, Biswas A, Sharma A, Malhotra A, Prasad KT, Vishnubhatla

S, Ajmani S, Mishra H, Soneja M, Broor S. Aetiology, outcomes & predictors of

mortality in acute respiratory distress syndrome from a tertiary care centre in

north India. Indian J Med Res. 2016 Jun;143(6):782-792. doi:

10.4103/0971-5916.192063. PubMed PMID: 27748303; PubMed Central PMCID:

PMC5094118.

177: van der Geest PJ, Mohseni M, Nieboer D, Duran S, Groeneveld ABJ.

Procalcitonin to guide taking blood cultures in the intensive care unit; a

cluster-randomized controlled trial. Clin Microbiol Infect. 2017 Feb;23(2):86-91.

doi: 10.1016/j.cmi.2016.10.004. Epub 2016 Oct 13. PubMed PMID: 27746396.

178: Díaz MG, García RP, Gamero DB, González-Tomé MI, Romero PC, Ferrer MM,

Contreras JR. Lack of Accuracy of Biomarkers and Physical Examination to Detect

Bacterial Infection in Febrile Infants. Pediatr Emerg Care. 2016

Oct;32(10):664-668. PubMed PMID: 25822238.

179: Arai T, Ohta S, Tsurukiri J, Kumasaka K, Nagata K, Okita T, Oomura T,

Hoshiai A, Koyama M, Yukioka T. Procalcitonin levels predict to identify

bacterial strains in blood cultures of septic patients. Am J Emerg Med. 2016

Nov;34(11):2150-2153. doi: 10.1016/j.ajem.2016.08.009. Epub 2016 Aug 7. PubMed

PMID: 27592459.

180: Thanachartwet V, Desakorn V, Sahassananda D, Jittmittraphap A, Oer-Areemitr

N, Osothsomboon S, Surabotsophon M, Wattanathum A. Serum Procalcitonin and

Peripheral Venous Lactate for Predicting Dengue Shock and/or Organ Failure: A

Prospective Observational Study. PLoS Negl Trop Dis. 2016 Aug 26;10(8):e0004961.

doi: 10.1371/journal.pntd.0004961. eCollection 2016 Aug. PubMed PMID: 27564863;

PubMed Central PMCID: PMC5001649.

181: Lindley LE, Stojadinovic O, Pastar I, Tomic-Canic M. Biology and Biomarkers

for Wound Healing. Plast Reconstr Surg. 2016 Sep;138(3 Suppl):18S-28S. doi:

10.1097/PRS.0000000000002682. Review. PubMed PMID: 27556760; PubMed Central

PMCID: PMC4998971.

182: Gies F, Tschiedel E, Felderhoff-Müser U, Rath PM, Steinmann J, Dohna-Schwake

C. Prospective evaluation of SeptiFast Multiplex PCR in children with systemic

inflammatory response syndrome under antibiotic treatment. BMC Infect Dis. 2016

Aug 8;16:378. doi: 10.1186/s12879-016-1722-9. PubMed PMID: 27503068; PubMed

Central PMCID: PMC4977629.

183: Kerin Povšič M, Ihan A, Beovič B. Post-Operative Infection Is an Independent

Risk Factor for Worse Long-Term Survival after Colorectal Cancer Surgery. Surg

Infect (Larchmt). 2016 Dec;17(6):700-712. Epub 2016 Aug 3. PubMed PMID: 27487109.

184: Kapasi AJ, Dittrich S, González IJ, Rodwell TC. Host Biomarkers for

Distinguishing Bacterial from Non-Bacterial Causes of Acute Febrile Illness: A

Comprehensive Review. PLoS One. 2016 Aug 3;11(8):e0160278. doi:

10.1371/journal.pone.0160278. eCollection 2016. Review. PubMed PMID: 27486746;

PubMed Central PMCID: PMC4972355.

185: Dotsenko TG, Shlikova GI, Teplyakova OV. [The analysis of synovial fluid:

clinical significance of derived results]. Klin Lab Diagn. 2016 Aug;61(8):478-84.

Review. Russian. PubMed PMID: 30601639.

186: van de Geijn GM, Denker S, Meuleman-van Waning V, Koeleman HG, Birnie E,

Braunstahl GJ, Njo TL. Evaluation of new laboratory tests to discriminate

bacterial from nonbacterial chronic obstructive pulmonary disease exacerbations.

Int J Lab Hematol. 2016 Dec;38(6):616-628. doi: 10.1111/ijlh.12550. Epub 2016 Jul

27. PubMed PMID: 27459873.

187: Mazankova LN, Milovanova OA, Moiseenkova DA, Soldatova IA, Mikhalinova EP.

[Neurological presentations of bacterial meningitis in children: current

possibilities of diagnosis and treatment]. Zh Nevrol Psikhiatr Im S S Korsakova.

2016;116(6):4-9. doi: 10.17116/jnevro2016116614-9. Russian. PubMed PMID:

27456896.

188: Zhou F, Gu L, Qu JX, Liu YM, Cao B; For CAP-China network. Evaluating the

utility of Binax NOW Streptococcus pneumoniae urinary antigen test in adults with

community acquired pneumonia in China. Clin Respir J. 2018 Feb;12(2):425-432.

doi: 10.1111/crj.12533. Epub 2016 Aug 8. PubMed PMID: 27437639.

189: Corti C, Fally M, Fabricius-Bjerre A, Mortensen K, Jensen BN, Andreassen HF,

Porsbjerg C, Knudsen JD, Jensen JU. Point-of-care procalcitonin test to reduce

antibiotic exposure in patients hospitalized with acute exacerbation of COPD. Int

J Chron Obstruct Pulmon Dis. 2016 Jun 22;11:1381-9. doi: 10.2147/COPD.S104051.

eCollection 2016. PubMed PMID: 27382274; PubMed Central PMCID: PMC4922826.

190: Kim SE. Serum Procalcitonin Is a Candidate Biomarker to Differentiate

Bacteremia from Disease Flares in Patients with Inflammatory Bowel Disease. Gut

Liver. 2016 Jul 15;10(4):491-2. doi: 10.5009/gnl16272. PubMed PMID: 27377736;

PubMed Central PMCID: PMC4933403.

191: Gilbert D, Gelfer G, Wang L, Myers J, Bajema K, Johnston M, Leggett J. The

potential of molecular diagnostics and serum procalcitonin levels to change the

antibiotic management of community-acquired pneumonia. Diagn Microbiol Infect

Dis. 2016 Sep;86(1):102-7. doi: 10.1016/j.diagmicrobio.2016.06.008. Epub 2016 Jun

15. PubMed PMID: 27377675.

192: Haliloglu M, Bilgili B, Haliloglu O, Gogas Yavuz D, Cinel I. Vitamin D level

is associated with mortality predictors in ventilator-associated pneumonia caused

by Acinetobacter baumannii. J Infect Dev Ctries. 2016 Jun 30;10(6):567-74. doi:

10.3855/jidc.8206. PubMed PMID: 27367004.

193: Ali FT, Ali MA, Elnakeeb MM, Bendary HN. Presepsin is an early monitoring

biomarker for predicting clinical outcome in patients with sepsis. Clin Chim

Acta. 2016 Sep 1;460:93-101. doi: 10.1016/j.cca.2016.06.030. Epub 2016 Jun 25.

PubMed PMID: 27353646.

194: Vincenzi B, Fioroni I, Pantano F, Angeletti S, Dicuonzo G, Zoccoli A,

Santini D, Tonini G. Procalcitonin as diagnostic marker of infection in solid

tumors patients with fever. Sci Rep. 2016 Jun 17;6:28090. doi: 10.1038/srep28090.

PubMed PMID: 27312877; PubMed Central PMCID: PMC4911581.

195: Tsujimoto K, Hata A, Fujita M, Hatachi S, Yagita M. Presepsin and

procalcitonin as biomarkers of systemic bacterial infection in patients with

rheumatoid arthritis. Int J Rheum Dis. 2018 Jul;21(7):1406-1413. doi:

10.1111/1756-185X.12899. Epub 2016 Jun 13. PubMed PMID: 27291096.

196: İşgüder R, Ceylan G, Ağın H, Gülfidan G, Ayhan Y, Devrim İ. New parameters

for childhood ventilator associated pneumonia diagnosis. Pediatr Pulmonol. 2017

Jan;52(1):119-128. doi: 10.1002/ppul.23504. Epub 2016 Jun 9. PubMed PMID:

27280471.

197: Qian W, Huang GZ. Neutrophil CD64 as a Marker of Bacterial Infection in

Acute Exacerbations of Chronic Obstructive Pulmonary Disease. Immunol Invest.

2016 Aug;45(6):490-503. doi: 10.1080/08820139.2016.1177540. Epub 2016 May 25.

PubMed PMID: 27224474.

198: Varetto G, Castagno C, Trucco A, Frola E, Bert F, Scozzari G, Rispoli P.

Serum Procalcitonin as a Valuable Diagnostic Tool in the Early Detection of

Infectious Complications after Open Abdominal Aortic Repair. Ann Vasc Surg. 2016

Jul;34:111-8. doi: 10.1016/j.avsg.2016.01.012. Epub 2016 May 9. PubMed PMID:

27157798.

199: Downes KJ, Weiss SL, Gerber JS, Klieger SB, Fitzgerald JC, Balamuth F, Kubis

SE, Tolomeo P, Bilker WB, Han X, Nachamkin I, Garrigan C, Han JH, Lautenbach E,

Coffin SE; Centers for Disease Control and Prevention Epicenters Program. A

Pragmatic Biomarker-Driven Algorithm to Guide Antibiotic Use in the Pediatric

Intensive Care Unit: The Optimizing Antibiotic Strategies in Sepsis (OASIS)

Study. J Pediatric Infect Dis Soc. 2017 Jun 1;6(2):134-141. doi:

10.1093/jpids/piw023. PubMed PMID: 27147715; PubMed Central PMCID: PMC5907860.

200: Sim JK, Oh JY, Lee EJ, Hur GY, Lee SH, Lee SY, Lee SY, Kim JH, Shin C, Shim

JJ, In KH, Kang KH, Min KH. Serum Procalcitonin for Differential Diagnosis of

Acute Exacerbation and Bacterial Pneumonia in Patients With Interstitial Lung

Disease. Am J Med Sci. 2016 May;351(5):499-505. doi: 10.1016/j.amjms.2016.02.029.

Epub 2016 Mar 31. PubMed PMID: 27140709.

201: Liu HH, Zhang MW, Guo JB, Li J, Su L. Procalcitonin and C-reactive protein

in early diagnosis of sepsis caused by either Gram-negative or Gram-positive

bacteria. Ir J Med Sci. 2017 Feb;186(1):207-212. doi: 10.1007/s11845-016-1457-z.

Epub 2016 May 2. PubMed PMID: 27139197.

202: Ozsurekci Y, Oktay Arıkan K, Bayhan C, Karadağ-Öncel E, Emre Aycan A, Gürbüz

V, Hasçelik G, Ceyhan M. Can procalcitonin be a diagnostic marker for

catheter-related blood stream infection in children? J Pediatr (Rio J). 2016

Jul-Aug;92(4):414-20. doi: 10.1016/j.jped.2015.11.004. Epub 2016 Apr 27. PubMed

PMID: 27131015.

203: Self WH, Grijalva CG, Williams DJ, Woodworth A, Balk RA, Fakhran S, Zhu Y,

Courtney DM, Chappell J, Anderson EJ, Qi C, Waterer GW, Trabue C, Bramley AM,

Jain S, Edwards KM, Wunderink RG. Procalcitonin as an Early Marker of the Need

for Invasive Respiratory or Vasopressor Support in Adults With Community-Acquired

Pneumonia. Chest. 2016 Oct;150(4):819-828. doi: 10.1016/j.chest.2016.04.010. Epub

2016 Apr 21. PubMed PMID: 27107491; PubMed Central PMCID: PMC5812770.

204: Huang MY, Chen CY, Chien JH, Wu KH, Chang YJ, Wu KH, Wu HP. Serum

Procalcitonin and Procalcitonin Clearance as a Prognostic Biomarker in Patients

with Severe Sepsis and Septic Shock. Biomed Res Int. 2016;2016:1758501. doi:

10.1155/2016/1758501. Epub 2016 Mar 20. PubMed PMID: 27088084; PubMed Central

PMCID: PMC4818793.

205: Ergan B, Şahin AA, Topeli A. Serum Procalcitonin as a Biomarker for the

Prediction of Bacterial Exacerbation and Mortality in Severe COPD Exacerbations

Requiring Mechanical Ventilation. Respiration. 2016;91(4):316-24. doi:

10.1159/000445440. Epub 2016 Apr 16. PubMed PMID: 27081845.

206: Póvoa P, Martin-Loeches I, Ramirez P, Bos LD, Esperatti M, Silvestre J, Gili

G, Goma G, Berlanga E, Espasa M, Gonçalves E, Torres A, Artigas A. Biomarker

kinetics in the prediction of VAP diagnosis: results from the BioVAP study. Ann

Intensive Care. 2016 Dec;6(1):32. doi: 10.1186/s13613-016-0134-8. Epub 2016 Apr

14. PubMed PMID: 27076187; PubMed Central PMCID: PMC4830786.

207: Sirinoglu M, Soysal A, Karaaslan A, Kepenekli Kadayifci E, Cinel I, Koç A,

Tokuç G, Yaman A, Haklar G, Şirikçi Ö, Turan S, Altınkanat Gelmez G, Söyletir G,

Bakır M. The diagnostic value of soluble urokinase plasminogen activator receptor

compared with C-reactive protein and procalcitonin in children with febrile

neutropenia. Pediatr Hematol Oncol. 2016 Apr;33(3):200-8. doi:

10.3109/08880018.2016.1155100. Epub 2016 Apr 8. PubMed PMID: 27057782.

208: Fukuzumi N, Osawa K, Sato I, Iwatani S, Ishino R, Hayashi N, Iijima K,

Saegusa J, Morioka I. Age-specific percentile-based reference curve of serum

procalcitonin concentrations in Japanese preterm infants. Sci Rep. 2016 Apr

1;6:23871. doi: 10.1038/srep23871. PubMed PMID: 27033746; PubMed Central PMCID:

PMC4817150.

209: Pilanci O, Ergin S, Sirekbasan S, Ersin I, Habip Z, Yuksel P, Kuvat N, Aslan

M, Dinc O, Saribas S, Kocazeybek B. Serum Neopterin and Procalcitonin Levels in

Relationship with Pediatric Burn Wound Infections. Acta Microbiol Immunol Hung.

2016 Mar;63(1):47-56. doi: 10.1556/030.63.2016.1.3. PubMed PMID: 27020868.

210: Povsic MK, Beovic B, Ihan A. Perioperative Increase in Neutrophil CD64

Expression is an Indicator for Intra-abdominal Infection after Colorectal Cancer

Surgery. Radiol Oncol. 2016 Mar 26;51(2):211-220. doi: 10.1515/raon-2016-0016.

eCollection 2017 Jun. PubMed PMID: 28740457; PubMed Central PMCID: PMC5514662.

211: Wagenlehner FM, Alidjanov J, Pilatz A. [Urosepsis. Update on diagnosis and

treatment]. Urologe A. 2016 Apr;55(4):454-9. doi: 10.1007/s00120-016-0066-9.

German. PubMed PMID: 27003569.

212: Sharma P, Patel K, Baria K, Lakhia K, Malhotra A, Shah K, Patel S.

Procalcitonin level for prediction of postoperative infection in cardiac surgery.

Asian Cardiovasc Thorac Ann. 2016 May;24(4):344-9. doi: 10.1177/0218492316640953.

Epub 2016 Mar 21. PubMed PMID: 27002098.

213: Kelly BJ, Lautenbach E, Nachamkin I, Coffin SE, Gerber JS, Fuchs BD,

Garrigan C, Han X, Bilker WB, Wise J, Tolomeo P, Han JH; Centers for Disease

Control and Prevention (CDC) Prevention Epicenters Program. Combined biomarkers

discriminate a low likelihood of bacterial infection among surgical intensive

care unit patients with suspected sepsis. Diagn Microbiol Infect Dis. 2016

May;85(1):109-15. doi: 10.1016/j.diagmicrobio.2016.01.003. Epub 2016 Jan 8.

PubMed PMID: 26971636; PubMed Central PMCID: PMC4841711.

214: Ercan N, Tuzcu N, Başbug O, Tuzcu M, Alim A. Diagnostic value of serum

procalcitonin, neopterin, and gamma interferon in neonatal calves with septicemic

colibacillosis. J Vet Diagn Invest. 2016 Mar;28(2):180-3. doi:

10.1177/1040638715626488. PubMed PMID: 26965240.

215: Bahloul M, Baccouch N, Chtara K, Turki M, Turki O, Hamida CB, Chelly H,

Ayedi F, Chaari A, Bouaziz M. Value of Serum Cholinesterase Activity in the

Diagnosis of Septic Shock Due to Bacterial Infections. J Intensive Care Med. 2017

Jun;32(5):346-352. doi: 10.1177/0885066616636549. Epub 2016 Mar 7. PubMed PMID:

26951579.

216: Zammarchi L, Antonelli A, Bartolini L, Pecile P, Trotta M, Rogasi PG,

Santini MG, Dilaghi B, Grifoni S, Rossolini GM, Bartoloni A. Louse-Borne

Relapsing Fever with Meningeal Involvement in an Immigrant from Somalia to Italy,

October 2015. Vector Borne Zoonotic Dis. 2016 May;16(5):352-5. doi:

10.1089/vbz.2015.1928. Epub 2016 Mar 3. PubMed PMID: 26938933.

217: Roques M, Chretien ML, Favennec C, Lafon I, Ferrant E, Legouge C, Plocque A,

Golfier C, Duvillard L, Amoureux L, Bastie JN, Maurin-Bernier L, Dalle F, Caillot

D. Evolution of procalcitonin, C-reactive protein and fibrinogen levels in

neutropenic leukaemia patients with invasive pulmonary aspergillosis or

mucormycosis. Mycoses. 2016 Jun;59(6):383-90. doi: 10.1111/myc.12487. Epub 2016

Mar 2. PubMed PMID: 26931315.

218: Abers MS, Sandvall BP, Sampath R, Zuno C, Uy N, Yu VL, Stager CE, Musher DM.

Postobstructive Pneumonia: An Underdescribed Syndrome. Clin Infect Dis. 2016 Apr

15;62(8):957-61. doi: 10.1093/cid/civ1212. Epub 2016 Feb 21. PubMed PMID:

26908806; PubMed Central PMCID: PMC4803103.

219: Wang T, Cui YL, Lin ZF, Chen DC. Comparative Study of Plasma Endotoxin with

Procalcitonin Levels in Diagnosis of Bacteremia in Intensive Care Unit Patients.

Chin Med J (Engl). 2016 Feb 20;129(4):417-23. doi: 10.4103/0366-6999.176064.

PubMed PMID: 26879015; PubMed Central PMCID: PMC4800842.

220: Heredia-Rodríguez M, Bustamante-Munguira J, Fierro I, Lorenzo M,

Jorge-Monjas P, Gómez-Sánchez E, Álvarez FJ, Bergese SD, Eiros JM, Bermejo-Martin

JF, Gómez-Herreras JI, Tamayo E. Procalcitonin cannot be used as a biomarker of

infection in heart surgery patients with acute kidney injury. J Crit Care. 2016

Jun;33:233-9. doi: 10.1016/j.jcrc.2016.01.015. Epub 2016 Jan 15. PubMed PMID:

26861073.

221: Douglas IS. New diagnostic methods for pneumonia in the ICU. Curr Opin

Infect Dis. 2016 Apr;29(2):197-204. doi: 10.1097/QCO.0000000000000249. Review.

PubMed PMID: 26859725.

222: Durnaś B, Wątek M, Wollny T, Niemirowicz K, Marzec M, Bucki R, Góźdź S.

Utility of blood procalcitonin concentration in the management of cancer patients

with infections. Onco Targets Ther. 2016 Jan 22;9:469-75. doi:

10.2147/OTT.S95600. eCollection 2016. Review. PubMed PMID: 26858528; PubMed

Central PMCID: PMC4731001.

223: Lee CS, Hwang JH, Lee JM, Lee JH. The clinical usefulness of serum

procalcitonin level in patients with scrub typhus. Korean J Intern Med. 2017

Jul;32(4):761-763. doi: 10.3904/kjim.2015.142. Epub 2016 Feb 4. PubMed PMID:

26842101; PubMed Central PMCID: PMC5511929.

224: Sato A, Kaido T, Iida T, Yagi S, Hata K, Okajima H, Takakura S, Ichiyama S,

Uemoto S. Bundled strategies against infection after liver transplantation:

Lessons from multidrug-resistant Pseudomonas aeruginosa. Liver Transpl. 2016

Apr;22(4):436-45. doi: 10.1002/lt.24407. PubMed PMID: 26824429.

225: Morales D, Moreno L, Herranz M, Bernaola E, Martínez-Baz I, Castilla J.

[Invasive meningococcal disease in Navarra in the era of a meningococcal C

vaccine]. An Pediatr (Barc). 2017 Apr;86(4):213-219. doi:

10.1016/j.anpedi.2015.12.001. Epub 2016 Jan 18. Spanish. PubMed PMID: 26795260.

226: Procalcitonin and febrile infants. Arch Dis Child. 2016 Feb;101(2):130. doi:

10.1136/archdischild-2015-310319. PubMed PMID: 26792901.

227: Tsalik EL, Henao R, Nichols M, Burke T, Ko ER, McClain MT, Hudson LL, Mazur

A, Freeman DH, Veldman T, Langley RJ, Quackenbush EB, Glickman SW, Cairns CB,

Jaehne AK, Rivers EP, Otero RM, Zaas AK, Kingsmore SF, Lucas J, Fowler VG Jr,

Carin L, Ginsburg GS, Woods CW. Host gene expression classifiers diagnose acute

respiratory illness etiology. Sci Transl Med. 2016 Jan 20;8(322):322ra11. doi:

10.1126/scitranslmed.aad6873. PubMed PMID: 26791949; PubMed Central PMCID:

PMC4905578.

228: Janež J, Korać T, Kodre AR, Jelenc F, Ihan A. Laparoscopically assisted

colorectal surgery provides better short-term clinical and inflammatory outcomes

compared to open colorectal surgery. Arch Med Sci. 2015 Dec 10;11(6):1217-26.

doi: 10.5114/aoms.2015.56348. Epub 2015 Dec 11. PubMed PMID: 26788083; PubMed

Central PMCID: PMC4697056.

229: Chung SH, Lee HW, Kim SW, Park SJ, Hong SP, Kim TI, Kim WH, Cheon JH.

Usefulness of Measuring Serum Procalcitonin Levels in Patients with Inflammatory

Bowel Disease. Gut Liver. 2016 Jul 15;10(4):574-80. doi: 10.5009/gnl15209. PubMed

PMID: 26780089; PubMed Central PMCID: PMC4933418.

230: Fukushima K, Kubo T, Ehara N, Nakano R, Matsutake T, Ishimatu Y, Tanaka Y,

Akamatsu S, Izumikawa K, Kohno S. A novel method for rapid detection of

Streptococcus pneumoniae antigens in blood. J Infect Chemother. 2016

Mar;22(3):143-8. doi: 10.1016/j.jiac.2015.12.004. Epub 2016 Jan 14. PubMed PMID:

26778250.

231: Duan J, Xie Y, Yang J, Luo Y, Guo Y, Wang C. Variation of Circulating

Inflammatory Mediators in Staphylococcus aureus and Escherichia coli Bloodstream

Infection. Med Sci Monit. 2016 Jan 16;22:161-71. PubMed PMID: 26772168; PubMed

Central PMCID: PMC4720174.

232: Dreger NM, Degener S, Ahmad-Nejad P, Wöbker G, Roth S. Urosepsis--Etiology,

Diagnosis, and Treatment. Dtsch Arztebl Int. 2015 Dec 4;112(49):837-47; quiz 848.

doi: 10.3238/arztebl.2015.0837. Review. PubMed PMID: 26754121; PubMed Central

PMCID: PMC4711296.

233: Rhee JY, Hong G, Ryu KM. Clinical Implications of 5 Cases of Middle East

Respiratory Syndrome Coronavirus Infection in a South Korean Outbreak. Jpn J

Infect Dis. 2016 Sep 21;69(5):361-6. doi: 10.7883/yoken.JJID.2015.445. Epub 2016

Jan 8. PubMed PMID: 26743151.

234: Angeletti S, Ciccozzi M, Fogolari M, Spoto S, Lo Presti A, Costantino S,

Dicuonzo G. Procalcitonin and MR-proAdrenomedullin combined score in the

diagnosis and prognosis of systemic and localized bacterial infections. J Infect.

2016 Mar;72(3):395-8. doi: 10.1016/j.jinf.2015.12.006. Epub 2015 Dec 23. PubMed

PMID: 26723912.

235: Pugni L, Pietrasanta C, Milani S, Vener C, Ronchi A, Falbo M, Arghittu M,

Mosca F. Presepsin (Soluble CD14 Subtype): Reference Ranges of a New Sepsis

Marker in Term and Preterm Neonates. PLoS One. 2015 Dec 31;10(12):e0146020. doi:

10.1371/journal.pone.0146020. eCollection 2015. PubMed PMID: 26720209; PubMed

Central PMCID: PMC4697794.

236: Singh S, Chatterji T, Sen M, Dhayal IR, Mishra S, Husain N, Goel A, Roy R.

Serum procalcitonin levels in combination with (1)H NMR spectroscopy: A rapid

indicator for differentiation of urosepsis. Clin Chim Acta. 2016 Jan

30;453:205-14. doi: 10.1016/j.cca.2015.12.021. Epub 2015 Dec 21. PubMed PMID:

26719034.

237: Zencir C, Akpek M, Senol S, Selvi M, Onay S, Cetin M, Akgullu C, Elbi H,

Gungor H. Association between hematologic parameters and in-hospital mortality in

patients with infective endocarditis. Kaohsiung J Med Sci. 2015 Dec;31(12):632-8.

doi: 10.1016/j.kjms.2015.10.004. Epub 2015 Nov 7. PubMed PMID: 26709225.

238: McHugh L, Seldon TA, Brandon RA, Kirk JT, Rapisarda A, Sutherland AJ,

Presneill JJ, Venter DJ, Lipman J, Thomas MR, Klein Klouwenberg PM, van Vught L,

Scicluna B, Bonten M, Cremer OL, Schultz MJ, van der Poll T, Yager TD, Brandon

RB. A Molecular Host Response Assay to Discriminate Between Sepsis and

Infection-Negative Systemic Inflammation in Critically Ill Patients: Discovery

and Validation in Independent Cohorts. PLoS Med. 2015 Dec 8;12(12):e1001916. doi:

10.1371/journal.pmed.1001916. eCollection 2015 Dec. PubMed PMID: 26645559; PubMed

Central PMCID: PMC4672921.

239: Yang M, Gao H, Chen J, Xu X, Tang L, Yang Y, Liang W, Yu L, Sheng J, Li L.

Bacterial coinfection is associated with severity of avian influenza A (H7N9),

and procalcitonin is a useful marker for early diagnosis. Diagn Microbiol Infect

Dis. 2016 Feb;84(2):165-9. doi: 10.1016/j.diagmicrobio.2015.10.018. Epub 2015 Oct

28. PubMed PMID: 26639228.

240: Angeletti S, Dicuonzo G, Fioravanti M, De Cesaris M, Fogolari M, Lo Presti

A, Ciccozzi M, De Florio L. Procalcitonin, MR-Proadrenomedullin, and Cytokines

Measurement in Sepsis Diagnosis: Advantages from Test Combination. Dis Markers.

2015;2015:951532. doi: 10.1155/2015/951532. Epub 2015 Nov 9. PubMed PMID:

26635427; PubMed Central PMCID: PMC4655267.

241: Yanai M, Gon Y, Suzuki K, Hayashi Y, Hasumi S, Otsuka H, Soma M. Clinical

usefulness of serum 2'-5'-oligoadenylate synthetase for early diagnosis of viral

infections among febrile adult patients. Infect Dis (Lond). 2016;48(5):338-42.

doi: 10.3109/23744235.2015.1118531. Epub 2015 Dec 1. PubMed PMID: 26624960.

242: Şahintürk Y, Çekiç B, Zorlu Görgülügil G, Harmandar FA, Uyar S, Çekin Y,

Çekin AH. Cirrhotic Ascites management via procalcitonin level and a new approach

B-mode gray-scale histogram. Turk J Gastroenterol. 2016 Jan;27(1):47-54. doi:

10.5152/tjg.2015.150345. Epub 2015 Nov 27. PubMed PMID: 26620960.

243: Abdel-Razik A, Mousa N, Elhammady D, Elhelaly R, Elzehery R, Elbaz S, Eissa

M, El-Wakeel N, Eldars W. Ascitic Fluid Calprotectin and Serum Procalcitonin as

Accurate Diagnostic Markers for Spontaneous Bacterial Peritonitis. Gut Liver.

2016 Jul 15;10(4):624-31. doi: 10.5009/gnl15120. PubMed PMID: 26601826; PubMed

Central PMCID: PMC4933425.

244: Sichev AA, Tabasaransky TΦ, Savin IA, Gorachev AS, Tenedieva VD, Abramov TA,

Oshorov AV, Polupan AA, Mazkovsky IV, Gavrilov AG, Potapov AA. [SEPTIC SHOCK IN

PATIENT WITH SEVERE HEAD TRAUMA]. Anesteziol Reanimatol. 2015 Jul-Aug;60(4):65-9.

Russian. PubMed PMID: 26596036.

245: Milcent K, Faesch S, Gras-Le Guen C, Dubos F, Poulalhon C, Badier I, Marc E,

Laguille C, de Pontual L, Mosca A, Nissack G, Biscardi S, Le Hors H, Louillet F,

Dumitrescu AM, Babe P, Vauloup-Fellous C, Bouyer J, Gajdos V. Use of

Procalcitonin Assays to Predict Serious Bacterial Infection in Young Febrile

Infants. JAMA Pediatr. 2016 Jan;170(1):62-9. doi:

10.1001/jamapediatrics.2015.3210. Erratum in: JAMA Pediatr. 2016 Jun

1;170(6):624. PubMed PMID: 26595253.

246: Kuppermann N, Mahajan P. Role of Serum Procalcitonin in Identifying Young

Febrile Infants With Invasive Bacterial Infections: One Step Closer to the Holy

Grail? JAMA Pediatr. 2016 Jan;170(1):17-8. doi: 10.1001/jamapediatrics.2015.3267.

PubMed PMID: 26595161.

247: Saeed K, Dale AP, Leung E, Cusack T, Mohamed F, Lockyer G, Arnaudov S, Wade

A, Moran B, Lewis G, Dryden M, Cecil T, Cepeda JA. Procalcitonin levels predict

infectious complications and response to treatment in patients undergoing

cytoreductive surgery for peritoneal malignancy. Eur J Surg Oncol. 2016

Feb;42(2):234-43. doi: 10.1016/j.ejso.2015.10.004. Epub 2015 Oct 29. PubMed PMID:

26560024.

248: Lubell Y, Blacksell SD, Dunachie S, Tanganuchitcharnchai A, Althaus T,

Watthanaworawit W, Paris DH, Mayxay M, Peto TJ, Dondorp AM, White NJ, Day NP,

Nosten F, Newton PN, Turner P. Performance of C-reactive protein and

procalcitonin to distinguish viral from bacterial and malarial causes of fever in

Southeast Asia. BMC Infect Dis. 2015 Nov 11;15:511. doi:

10.1186/s12879-015-1272-6. PubMed PMID: 26558692; PubMed Central PMCID:

PMC4642613.

249: Oussalah A, Ferrand J, Filhine-Tresarrieu P, Aissa N, Aimone-Gastin I,

Namour F, Garcia M, Lozniewski A, Guéant JL. Diagnostic Accuracy of Procalcitonin

for Predicting Blood Culture Results in Patients With Suspected Bloodstream

Infection: An Observational Study of 35,343 Consecutive Patients (A

STROBE-Compliant Article). Medicine (Baltimore). 2015 Nov;94(44):e1774. doi:

10.1097/MD.0000000000001774. PubMed PMID: 26554775; PubMed Central PMCID:

PMC4915876.

250: Massara M, De Caridi G, Serra R, Barillà D, Cutrupi A, Volpe A, Cutrupi F,

Alberti A, Volpe P. The role of procalcitonin as a marker of diabetic foot ulcer

infection. Int Wound J. 2017 Feb;14(1):31-34. doi: 10.1111/iwj.12536. Epub 2015

Oct 28. PubMed PMID: 26511007.

251: Bunz H, Weyrich P, Peter A, Baumann D, Tschritter O, Guthoff M, Beck R, Jahn

G, Artunc F, Häring HU, Heyne N, Wagner R. Urinary Neutrophil

Gelatinase-Associated Lipocalin (NGAL) and proteinuria predict severity of acute

kidney injury in Puumala virus infection. BMC Infect Dis. 2015 Oct 27;15:464.

doi: 10.1186/s12879-015-1180-9. PubMed PMID: 26503619; PubMed Central PMCID:

PMC4621931.

252: Xiao Z, Wilson C, Robertson HL, Roberts DJ, Ball CG, Jenne CN, Kirkpatrick

AW. Inflammatory mediators in intra-abdominal sepsis or injury - a scoping

review. Crit Care. 2015 Oct 27;19:373. doi: 10.1186/s13054-015-1093-4. Review.

PubMed PMID: 26502877; PubMed Central PMCID: PMC4623902.

253: Paño-Pardo JR, Schüffelmann-Gutiérrez C, Escosa-García L, Laplaza-González

M, Moreno-Ramos F, Gómez-Gil R, López JD, Jordán I, Téllez C, de la Oliva P.

Opportunities to improve antimicrobial use in paediatric intensive care units: a

nationwide survey in Spain. Clin Microbiol Infect. 2016 Feb;22(2):171-177. doi:

10.1016/j.cmi.2015.10.015. Epub 2015 Oct 21. PubMed PMID: 26498852.

254: Jiang N, Ma YF, Jiang Y, Zhao XQ, Xie GP, Hu YJ, Qin CH, Yu B. Clinical

Characteristics and Treatment of Extremity Chronic Osteomyelitis in Southern

China: A Retrospective Analysis of 394 Consecutive Patients. Medicine

(Baltimore). 2015 Oct;94(42):e1874. doi: 10.1097/MD.0000000000001874. PubMed

PMID: 26496345; PubMed Central PMCID: PMC4620766.

255: Mandell IM, Aghamohammadi S, Deakers T, Khemani RG. Procalcitonin to Detect

Suspected Bacterial Infections in the PICU. Pediatr Crit Care Med. 2016

Jan;17(1):e4-12. doi: 10.1097/PCC.0000000000000571. PubMed PMID: 26492060.

256: Shu LH, Xu JJ, Wang S, Zhong HQ, Dong XY, Jiang K, Zhang HY, Xiong Q, Wang

C, Sun T, Sun C, Lu Q. [Distribution of pathogenic microorganisms and its

relationship with clinical features in children with community-acquired

pneumonia]. Zhongguo Dang Dai Er Ke Za Zhi. 2015 Oct;17(10):1056-61. Chinese.

PubMed PMID: 26483223.

257: Jiang YN, Cai X, Zhou HM, Jin WD, Zhang M, Zhang Y, Du XX, Chen ZH.

Diagnostic and prognostic roles of soluble CD22 in patients with Gram-negative

bacterial sepsis. Hepatobiliary Pancreat Dis Int. 2015 Oct;14(5):523-9. PubMed

PMID: 26459729.

258: Hatipoglu M, Gozdas HT. The role of procalcitonin in febrile patients.

Indian J Med Res. 2015 Sep;142(3):346. doi: 10.4103/0971-5916.166603. PubMed

PMID: 26458353; PubMed Central PMCID: PMC4669872.

259: Miglietta F, Faneschi ML, Lobreglio G, Palumbo C, Rizzo A, Cucurachi M,

Portaccio G, Guerra F, Pizzolante M. Procalcitonin, C-reactive protein and serum

lactate dehydrogenase in the diagnosis of bacterial sepsis, SIRS and systemic

candidiasis. Infez Med. 2015 Sep;23(3):230-7. PubMed PMID: 26397291.

260: Autilio C, Morelli R, Milardi D, Grande G, Marana R, Pontecorvi A, Zuppi C,

Baroni S. Soluble urokinase-type plasminogen activator receptor as a putative

marker of male accessory gland inflammation. Andrology. 2015 Nov;3(6):1054-61.

doi: 10.1111/andr.12084. Epub 2015 Sep 18. PubMed PMID: 26384478.

261: Işlak Mutcalı S, Saltoğlu N, Balkan İİ, Özaras R, Yemişen M, Tabak F, Mert

A, Öztürk R, Öngören Ş, Başlar Z, Aydın Y, Ferhanoğlu B, Soysal T. Early Changes

of Mannose-Binding Lectin, H-Ficolin, and Procalcitonin in Patients with Febrile

Neutropenia: A Prospective Observational Study. Turk J Haematol. 2016 Dec

1;33(4):304-310. doi: 10.4274/tjh.2014.0385. Epub 2015 Aug 6. PubMed PMID:

26377840; PubMed Central PMCID: PMC5204185.

262: Thursky KA, Worth LJ. Can mortality of cancer patients with fever and

neutropenia be improved? Curr Opin Infect Dis. 2015 Dec;28(6):505-13. doi:

10.1097/QCO.0000000000000202. Review. PubMed PMID: 26374951.

263: Ryu JA, Yang JH, Lee D, Park CM, Suh GY, Jeon K, Cho J, Baek SY, Carriere

KC, Chung CR. Clinical Usefulness of Procalcitonin and C-Reactive Protein as

Outcome Predictors in Critically Ill Patients with Severe Sepsis and Septic

Shock. PLoS One. 2015 Sep 14;10(9):e0138150. doi: 10.1371/journal.pone.0138150.

eCollection 2015. PubMed PMID: 26367532; PubMed Central PMCID: PMC4569178.

264: Popiel KY, Gheorghe R, Eastmond J, Miller MA. Usefulness of Adjunctive Fecal

Calprotectin and Serum Procalcitonin in Individuals Positive for Clostridium

difficile Toxin Gene by PCR Assay. J Clin Microbiol. 2015 Nov;53(11):3667-9. doi:

10.1128/JCM.02230-15. Epub 2015 Sep 9. PubMed PMID: 26354814; PubMed Central

PMCID: PMC4609681.

265: Gelfer G, Leggett J, Myers J, Wang L, Gilbert DN. The clinical impact of the

detection of potential etiologic pathogens of community-acquired pneumonia. Diagn

Microbiol Infect Dis. 2015 Dec;83(4):400-6. doi:

10.1016/j.diagmicrobio.2015.08.001. Epub 2015 Aug 5. PubMed PMID: 26341706.

266: Lopardo G, Basombrío A, Clara L, Desse J, De Vedia L, Di Libero E, Gañete M,

López Furst MJ, Mykietiuk A, Nemirovsky C, Osuna C, Pensotti C, Scapellato P.

[Guidelines for management of community-acquired pneumonia in adults]. Medicina

(B Aires). 2015;75(4):245-57. Spanish. PubMed PMID: 26339883.

267: Yu Y, Li XX, Jiang LX, Du M, Liu ZG, Cen ZR, Wang H, Guo ZH, Chang P.

Procalcitonin levels in patients with positive blood culture, positive body fluid

culture, sepsis, and severe sepsis: a cross-sectional study. Infect Dis (Lond).

2016;48(1):63-9. doi: 10.3109/23744235.2015.1082618. Epub 2015 Sep 4. PubMed

PMID: 26337821.

268: Han JH, Nachamkin I, Coffin SE, Gerber JS, Fuchs B, Garrigan C, Han X,

Bilker WB, Wise J, Tolomeo P, Lautenbach E; Prevention Epicenters Program of the

Centers for Disease Control and Prevention. Use of a Combination Biomarker

Algorithm To Identify Medical Intensive Care Unit Patients with Suspected Sepsis

at Very Low Likelihood of Bacterial Infection. Antimicrob Agents Chemother. 2015

Oct;59(10):6494-500. doi: 10.1128/AAC.00958-15. Epub 2015 Aug 3. PubMed PMID:

26239984; PubMed Central PMCID: PMC4576082.

269: Martini S, Tumietto F, Sciutti R, Greco L, Faldella G, Corvaglia L.

Methicillin-resistant Staphylococcus aureus mandibular osteomyelitis in an

extremely low birth weight preterm infant. Ital J Pediatr. 2015 Aug 4;41:54. doi:

10.1186/s13052-015-0163-1. PubMed PMID: 26239708; PubMed Central PMCID:

PMC4523912.

270: Çelik HT, Portakal O, Yiğit Ş, Hasçelik G, Korkmaz A, Yurdakök M. Efficacy

of new leukocyte parameters versus serum C-reactive protein, procalcitonin, and

interleukin-6 in the diagnosis of neonatal sepsis. Pediatr Int. 2016

Feb;58(2):119-25. doi: 10.1111/ped.12754. PubMed PMID: 26190096.

271: Dipalo M, Guido L, Micca G, Pittalis S, Locatelli M, Motta A, Bianchi V,

Callegari T, Aloe R, Da Rin G, Lippi G. Multicenter comparison of automated

procalcitonin immunoassays. Pract Lab Med. 2015 Jul 17;2:22-28. doi:

10.1016/j.plabm.2015.07.001. eCollection 2015 Aug 1. PubMed PMID: 28932801;

PubMed Central PMCID: PMC5597721.

272: Topcuoglu S, Arslanbuga C, Gursoy T, Aktas A, Karatekin G, Uluhan R, Ovali

F. Role of presepsin in the diagnosis of late-onset neonatal sepsis in preterm

infants. J Matern Fetal Neonatal Med. 2016;29(11):1834-9. doi:

10.3109/14767058.2015.1064885. Epub 2015 Jul 30. PubMed PMID: 26135765.

273: Scicluna BP, Klein Klouwenberg PM, van Vught LA, Wiewel MA, Ong DS,

Zwinderman AH, Franitza M, Toliat MR, Nürnberg P, Hoogendijk AJ, Horn J, Cremer

OL, Schultz MJ, Bonten MJ, van der Poll T. A molecular biomarker to diagnose

community-acquired pneumonia on intensive care unit admission. Am J Respir Crit

Care Med. 2015 Oct 1;192(7):826-35. doi: 10.1164/rccm.201502-0355OC. PubMed PMID:

26121490.

274: Luo Q, Hao F, Zhang M, Guo C. Serum bacterial DNA detection in patients with

cholangitis after Kasai procedure. Pediatr Int. 2015 Oct;57(5):954-60. doi:

10.1111/ped.12737. PubMed PMID: 26096812.

275: Dias Costa F, Ramos Andrade D, Cunha FI, Fernandes A. Group B streptococcal

neonatal parotitis. BMJ Case Rep. 2015 Jun 10;2015. pii: bcr2014209115. doi:

10.1136/bcr-2014-209115. PubMed PMID: 26063107; PubMed Central PMCID: PMC4480078.

276: Angeletti S, Spoto S, Fogolari M, Cortigiani M, Fioravanti M, De Florio L,

Curcio B, Cavalieri D, Costantino S, Dicuonzo G. Diagnostic and prognostic role

of procalcitonin (PCT) and MR-pro-Adrenomedullin (MR-proADM) in bacterial

infections. APMIS. 2015 Sep;123(9):740-8. doi: 10.1111/apm.12406. Epub 2015 Jun

8. PubMed PMID: 26058482.

277: Yu X, Ma X, Ai Y. [Diagnostic value of serum procalcitonin for infection in

the immunocompromised critically ill patients with suspected infection]. Zhonghua

Wei Zhong Bing Ji Jiu Yi Xue. 2015 Jun;27(6):477-83. doi:

10.3760/cma.j.issn.2095-4352.2015.06.012. Chinese. PubMed PMID: 26049187.

278: Ratzinger F, Haslacher H, Perkmann T, Schmetterer KG, Poeppl W, Mitteregger

D, Dorffner G, Burgmann H. Sepsis biomarkers in neutropaenic systemic

inflammatory response syndrome patients on standard care wards. Eur J Clin

Invest. 2015 Aug;45(8):815-23. doi: 10.1111/eci.12476. PubMed PMID: 26046926.

279: Scheinpflug K, Schalk E, Grabert E, Achenbach HJ. Procalcitonin Is Not

Useful to Discriminate Between Infectious and Noninfectious CRP Elevation in

Patients with Non-Small Cell Lung Cancer. Infect Control Hosp Epidemiol. 2015

Sep;36(9):1117-8. doi: 10.1017/ice.2015.134. Epub 2015 May 26. PubMed PMID:

26006190.

280: Jordan I, Calzada Y, Monfort L, Vila-Pérez D, Felipe A, Ortiz J, Cambra FJ,

Muñoz-Almagro C. Clinical, biochemical and microbiological factors associated

with the prognosis of pneumococcal meningitis in children. Enferm Infecc

Microbiol Clin. 2016 Feb;34(2):101-7. doi: 10.1016/j.eimc.2015.03.004. Epub 2015

May 19. PubMed PMID: 25998267.

281: Guo SY, Zhou Y, Hu QF, Yao J, Wang H. Procalcitonin is a marker of

gram-negative bacteremia in patients with sepsis. Am J Med Sci. 2015

Jun;349(6):499-504. doi: 10.1097/MAJ.0000000000000477. PubMed PMID: 25992537;

PubMed Central PMCID: PMC4450899.

282: Fujita M, Takahashi A, Imaizumi H, Hayashi M, Okai K, Kanno Y, Abe K,

Watanabe H, Ohira H. Drug-induced Liver Injury with HHV-6 Reactivation. Intern

Med. 2015;54(10):1219-22. doi: 10.2169/internalmedicine.54.4329. Epub 2015 May

15. PubMed PMID: 25986259.

283: Hortmann M, Singler K, Geier F, Christ M. [Recognition of infections in

elderly emergency patients]. Z Gerontol Geriatr. 2015 Oct;48(7):601-7. doi:

10.1007/s00391-015-0903-2. Epub 2015 May 19. Review. German. PubMed PMID:

25986073.

284: Aliyev DA, Vezirova ZSh, Geyusheva TF. [Procalcitonin as a predictor of

bacteremia in pediatric patients with malignancies and febrile neutropenia]. Klin

Khir. 2015 Feb;(2):70-2. Russian. PubMed PMID: 25985703.

285: Cooke J, Butler C, Hopstaken R, Dryden MS, McNulty C, Hurding S, Moore M,

Livermore DM. Narrative review of primary care point-of-care testing (POCT) and

antibacterial use in respiratory tract infection (RTI). BMJ Open Respir Res. 2015

May 6;2(1):e000086. doi: 10.1136/bmjresp-2015-000086. eCollection 2015. PubMed

PMID: 25973210; PubMed Central PMCID: PMC4426285.

286: Hoshina T, Takimoto T, Nanishi E, Nishio H, Kusuhara K, Hara T. The

uselessness of procalcitonin in the diagnosis of focal bacterial central nervous

system infection. J Infect Chemother. 2015 Aug;21(8):620-2. doi:

10.1016/j.jiac.2015.04.003. Epub 2015 Apr 18. PubMed PMID: 25971424.

287: Dulay AT, Buhimschi IA, Zhao G, Bahtiyar MO, Thung SF, Cackovic M, Buhimschi

CS. Compartmentalization of acute phase reactants Interleukin-6, C-Reactive

Protein and Procalcitonin as biomarkers of intra-amniotic infection and

chorioamnionitis. Cytokine. 2015 Dec;76(2):236-243. doi:

10.1016/j.cyto.2015.04.014. Epub 2015 May 6. PubMed PMID: 25957466; PubMed

Central PMCID: PMC4824401.

288: Galván JM, Rajas O, Aspa J. Review of Non-Bacterial Infections in

Respiratory Medicine: Viral Pneumonia. Arch Bronconeumol. 2015 Nov;51(11):590-7.

doi: 10.1016/j.arbres.2015.02.015. Epub 2015 May 7. Review. English, Spanish.

PubMed PMID: 25957460.

289: Viaggi B, Sbrana F, Malacarne P, Tascini C. Ventilator-associated pneumonia

caused by colistin-resistant KPC-producing Klebsiella pneumoniae: a case report

and literature review. Respir Investig. 2015 May;53(3):124-8. doi:

10.1016/j.resinv.2015.01.001. Epub 2015 Feb 20. Review. PubMed PMID: 25951099.

290: Hatipoglu M, Karagoz E. Clinical usefulness of procalcitonin as a marker of

sepsis: a novel predictor of causative pathogens? Intern Med. 2015;54(9):1163.

doi: 10.2169/internalmedicine.54.3749. Epub 2015 May 1. PubMed PMID: 25948373.

291: Chang CH, Tsao KC, Hu HC, Huang CC, Kao KC, Chen NH, Yang CT, Tsai YH, Hsieh

MJ. Procalcitonin and C-reactive protein cannot differentiate bacterial or viral

infection in COPD exacerbation requiring emergency department visits. Int J Chron

Obstruct Pulmon Dis. 2015 Apr 13;10:767-74. doi: 10.2147/COPD.S76740. eCollection

2015. PubMed PMID: 25926728; PubMed Central PMCID: PMC4403815.

292: Zhou F, Ning H, Chen F, Wu W, Chen A, Zhang J. Burkholderia gladioli

infection isolated from the blood cultures of newborns in the neonatal intensive

care unit. Eur J Clin Microbiol Infect Dis. 2015 Aug;34(8):1533-7. doi:

10.1007/s10096-015-2382-1. Epub 2015 Apr 30. PubMed PMID: 25926303.

293: Branche AR, Walsh EE, Vargas R, Hulbert B, Formica MA, Baran A, Peterson DR,

Falsey AR. Serum Procalcitonin Measurement and Viral Testing to Guide Antibiotic

Use for Respiratory Infections in Hospitalized Adults: A Randomized Controlled

Trial. J Infect Dis. 2015 Dec 1;212(11):1692-700. doi: 10.1093/infdis/jiv252.

Epub 2015 Apr 24. PubMed PMID: 25910632; PubMed Central PMCID: PMC4633755.

294: Daneman N, Rishu AH, Xiong W, Bagshaw SM, Cook DJ, Dodek P, Hall R, Kumar A,

Lamontagne F, Lauzier F, Marshall JC, Martin CM, McIntyre L, Muscedere J,

Reynolds S, Stelfox HT, Fowler RA; Canadian Critical Care Trials Group.

Bacteremia Antibiotic Length Actually Needed for Clinical Effectiveness

(BALANCE): study protocol for a pilot randomized controlled trial. Trials. 2015

Apr 18;16:173. doi: 10.1186/s13063-015-0688-z. PubMed PMID: 25903783; PubMed

Central PMCID: PMC4407544.

295: Cai ZH, Fan CL, Zheng JF, Zhang X, Zhao WM, Li B, Li L, Dong PL, Ding HG.

Measurement of serum procalcitonin levels for the early diagnosis of spontaneous

bacterial peritonitis in patients with decompensated liver cirrhosis. BMC Infect

Dis. 2015 Feb 13;15:55. doi: 10.1186/s12879-015-0776-4. PubMed PMID: 25887691;

PubMed Central PMCID: PMC4332920.

296: Hegazy MA, Omar AS, Samir N, Moharram A, Weber S, Radwan WA. Amalgamation of

procalcitonin, C-reactive protein, and sequential organ failure scoring system in

predicting sepsis survival. Anesth Essays Res. 2014 Sep-Dec;8(3):296-301. doi:

10.4103/0259-1162.143115. PubMed PMID: 25886324; PubMed Central PMCID:

PMC4258970.

297: Sedef AM, Kose F, Mertsoylu H, Ozyilkan O. Procalcitonin as a biomarker for

infection-related mortality in cancer patients. Curr Opin Support Palliat Care.

2015 Jun;9(2):168-73. doi: 10.1097/SPC.0000000000000142. Review. PubMed PMID:

25872114.

298: Gul S, Ozturk DB, Kisa U, Kacmaz B, Yesilyurt M. Procalcitonin Level and Its

Predictive Effect on Mortality in Crimean-Congo Hemorrhagic Fever Patients. Jpn J

Infect Dis. 2015;68(6):511-3. doi: 10.7883/yoken.JJID.2014.485. Epub 2015 Apr 10.

PubMed PMID: 25866108.

299: Lüdemann CM, Schütze N, Rudert M. [Diagnosis of periprosthetic hip

infections]. Oper Orthop Traumatol. 2015 Jun;27(3):237-50; quiz 251. doi:

10.1007/s00064-015-0362-3. Epub 2015 Apr 11. German. PubMed PMID: 25860787.

300: Leli C, Ferranti M, Moretti A, Al Dhahab ZS, Cenci E, Mencacci A.

Procalcitonin levels in gram-positive, gram-negative, and fungal bloodstream

infections. Dis Markers. 2015;2015:701480. doi: 10.1155/2015/701480. Epub 2015

Mar 17. PubMed PMID: 25852221; PubMed Central PMCID: PMC4380090.

301: Weimann K, Zimmermann M, Spies CD, Wernecke KD, Vicherek O, Nachtigall I,

Tafelski S, Weimann A. Intensive Care Infection Score--A new approach to

distinguish between infectious and noninfectious processes in intensive care and

medicosurgical patients. J Int Med Res. 2015 Jun;43(3):435-51. doi:

10.1177/0300060514557711. Epub 2015 Apr 7. PubMed PMID: 25850686.

302: Stubljar D, Skvarc M. Effective Strategies for Diagnosis of Systemic

Inflammatory Response Syndrome (SIRS) due to Bacterial Infection in Surgical

Patients. Infect Disord Drug Targets. 2015;15(1):53-6. Review. PubMed PMID:

25809624.

303: Oved K, Cohen A, Boico O, Navon R, Friedman T, Etshtein L, Kriger O,

Bamberger E, Fonar Y, Yacobov R, Wolchinsky R, Denkberg G, Dotan Y, Hochberg A,

Reiter Y, Grupper M, Srugo I, Feigin P, Gorfine M, Chistyakov I, Dagan R, Klein

A, Potasman I, Eden E. A novel host-proteome signature for distinguishing between

acute bacterial and viral infections. PLoS One. 2015 Mar 18;10(3):e0120012. doi:

10.1371/journal.pone.0120012. eCollection 2015. PubMed PMID: 25785720; PubMed

Central PMCID: PMC4364938.

304: Glaudemans AW, Uçkay I, Lipsky BA. Challenges in diagnosing infection in the

diabetic foot. Diabet Med. 2015 Jun;32(6):748-59. doi: 10.1111/dme.12750. Epub

2015 Apr 15. Review. PubMed PMID: 25765225.

305: Verduri A, Luppi F, D'Amico R, Balduzzi S, Vicini R, Liverani A, Ruggieri V,

Plebani M, Barbaro MP, Spanevello A, Canonica GW, Papi A, Fabbri LM, Beghè B;

FARM58J2XH Study Group. Antibiotic treatment of severe exacerbations of chronic

obstructive pulmonary disease with procalcitonin: a randomized noninferiority

trial. PLoS One. 2015 Mar 11;10(3):e0118241. doi: 10.1371/journal.pone.0118241.

eCollection 2015. PubMed PMID: 25760346; PubMed Central PMCID: PMC4356612.

306: Lee WS, Kang DW, Back JH, Kim HL, Chung JH, Shin BC. Cutoff value of serum

procalcitonin as a diagnostic biomarker of infection in end-stage renal disease

patients. Korean J Intern Med. 2015 Mar;30(2):198-204. doi:

10.3904/kjim.2015.30.2.198. Epub 2015 Feb 27. PubMed PMID: 25750561; PubMed

Central PMCID: PMC4351326.

307: Zhu G, Zhu J, Song L, Cai W, Wang J. Combined use of biomarkers for

distinguishing between bacterial and viral etiologies in pediatric lower

respiratory tract infections. Infect Dis (Lond). 2015 May;47(5):289-93. doi:

10.3109/00365548.2014.987163. Epub 2015 Feb 24. PubMed PMID: 25712729.

308: Martínez L AB, Marañón P R, Cobo E PV, Tomatis S C, Guerra M L, Peñalba C

AC. [Use of procalcitonin as diagnostic marker in acute gastroenteritis]. Rev

Chil Pediatr. 2014 Apr;85(2):157-63. doi: 10.4067/S0370-41062014000200004.

Spanish. PubMed PMID: 25697203.

309: Barrett J, Edgeworth J, Wyncoll D. Shortening the course of antibiotic

treatment in the intensive care unit. Expert Rev Anti Infect Ther. 2015

Apr;13(4):463-71. doi: 10.1586/14787210.2015.1008451. Epub 2015 Feb 3. Review.

PubMed PMID: 25645293.

310: de Oliveira TH, Amorin AT, Rezende IS, Santos Barbosa M, Martins HB, Brito

AK, Andrade EF, Gonçalves GK, Campos GB, Silva RA, Timenetsky J, Marques LM.

Sepsis induced by Staphylococcus aureus: participation of biomarkers in a murine

model. Med Sci Monit. 2015 Jan 29;21:345-55. doi: 10.12659/MSM.892528. PubMed

PMID: 25630550; PubMed Central PMCID: PMC4321564.

311: Akin H, Akalin H, Budak F, Ener B, Ocakoğlu G, Gürcüoğlu E, Göral G, Oral

HB. Alterations of serum cytokine levels and their relation with inflammatory

markers in candidemia. Med Mycol. 2015 Apr;53(3):258-68. doi: 10.1093/mmy/myu084.

Epub 2015 Jan 26. PubMed PMID: 25627661.

312: Wang Z, Zhang X, Wu J, Zhang W, Kuang H, Li X, Xuan W, Wang K, Ma L.

[Diagnostic value of serum procalcitonin in identifying the etiology of

non-responding community-acquired pneumonia after initial antibiotic therapy].

Zhonghua Jie He He Hu Xi Za Zhi. 2014 Nov;37(11):824-30. Chinese. PubMed PMID:

25604112.

313: Song GG, Bae SC, Lee YH. Diagnostic accuracies of procalcitonin and

C-reactive protein for bacterial infection in patients with systemic rheumatic

diseases: a meta-analysis. Clin Exp Rheumatol. 2015 Mar-Apr;33(2):166-73. Epub

2015 Jan 20. Review. PubMed PMID: 25602442.

314: García de Guadiana-Romualdo L, Español-Morales I, Cerezuela-Fuentes P,

Consuegra-Sánchez L, Hernando-Holgado A, Esteban-Torrella P, Jiménez-Santos E,

Viqueira-González M, de Béjar-Almira Á, Albaladejo-Otón MD. Value of

lipopolysaccharide binding protein as diagnostic marker of infection in adult

cancer patients with febrile neutropenia: comparison with C-reactive protein,

procalcitonin, and interleukin 6. Support Care Cancer. 2015 Jul;23(7):2175-82.

doi: 10.1007/s00520-014-2589-1. Epub 2015 Jan 7. PubMed PMID: 25564222.

315: Tarai B, Ravishankar N, Vohra P, Das P. Hemophilus influenzae meningitis and

septicaemia in a 14-month-old child after primary immunisation. Indian J Med

Microbiol. 2015 Jan-Mar;33(1):158-60. doi: 10.4103/0255-0857.148431. PubMed PMID:

25560025.

316: Fujii K, Yamada K, Sano J, Mashima D, Takeda H. [Investigation of the

procalcitonin and microbiology test and antibiotics situation from our hospital].

Rinsho Biseibutshu Jinsoku Shindan Kenkyukai Shi. 2014;25(1):7-15. Japanese.

PubMed PMID: 25537179.

317: Poggi C, Bianconi T, Gozzini E, Generoso M, Dani C. Presepsin for the

detection of late-onset sepsis in preterm newborns. Pediatrics. 2015

Jan;135(1):68-75. doi: 10.1542/peds.2014-1755. Epub 2014 Dec 15. PubMed PMID:

25511124.

318: Iakovlev SV, Suvorova MP, Kolendo SE, Burmistrova EN, Sergeeva EV,

Cherkasova NA, Eremina LV. [Clinical efficacy of the antimicrobial drug furamag

in nosocomial urinary tract infections]. Ter Arkh. 2014;86(10):65-72. Russian.

PubMed PMID: 25509895.

319: Jensen JU, Hein L, Lundgren B, Bestle MH, Mohr T, Andersen MH, Løken J,

Tousi H, Søe-Jensen P, Lauritsen AØ, Strange D, Petersen JA, Thormar K, Larsen

KM, Drenck NE, Helweg-Larsen J, Johansen ME, Reinholdt K, Møller JK, Olesen B,

Arendrup MC, Østergaard C, Cozzi-Lepri A, Grarup J, Lundgren JD; Procalcitonin

and Survival Study Group. Invasive Candida infections and the harm from

antibacterial drugs in critically ill patients: data from a randomized,

controlled trial to determine the role of ciprofloxacin, piperacillin-tazobactam,

meropenem, and cefuroxime. Crit Care Med. 2015 Mar;43(3):594-602. doi:

10.1097/CCM.0000000000000746. PubMed PMID: 25493970.

320: Paosong S, Narongroeknawin P, Pakchotanon R, Asavatanabodee P, Chaiamnuay S.

Serum procalcitonin as a diagnostic aid in patients with acute bacterial septic

arthritis. Int J Rheum Dis. 2015 Mar;18(3):352-9. doi: 10.1111/1756-185X.12496.

Epub 2014 Dec 3. PubMed PMID: 25469944.

321: Karagöz E, Bektore B, Tanoglu A. Procalcitonin: an emerging prognostic

factor of bacterial coinfection in infants with acute bronchiolitis? Pediatr

Emerg Care. 2014 Dec;30(12):e8. doi: 10.1097/PEC.0000000000000300. PubMed PMID:

25469610.

322: Salerno D, Mushatt D, Myers L, Zhuang Y, de la Rua N, Calderon EJ, Welsh DA.

Serum and bal beta-D-glucan for the diagnosis of Pneumocystis pneumonia in HIV

positive patients. Respir Med. 2014 Nov;108(11):1688-95. PubMed PMID: 25448310;

PubMed Central PMCID: PMC4297544.

323: Green RS, Gorman SK. Emergency department antimicrobial considerations in

severe sepsis. Emerg Med Clin North Am. 2014 Nov;32(4):835-49. doi:

10.1016/j.emc.2014.07.014. Epub 2014 Sep 16. Review. PubMed PMID: 25441038.

324: Palmiere C, Egger C. Usefulness of pericardial and pleural fluids for the

postmortem diagnosis of sepsis. J Forensic Leg Med. 2014 Nov;28:15-8. doi:

10.1016/j.jflm.2014.09.006. Epub 2014 Sep 19. PubMed PMID: 25440141.

325: Pinzone MR, Cacopardo B, Abbo L, Nunnari G. Optimal duration of

antimicrobial therapy in ventilator-associated pneumonia: What is the role for

procalcitonin? J Glob Antimicrob Resist. 2014 Dec;2(4):239-244. doi:

10.1016/j.jgar.2014.06.004. Epub 2014 Jul 23. Review. PubMed PMID: 27873682.

326: Karagoz E, Tanoglu A. Clinical importance of serum procalcitonin in

ulcerative colitis patients. World J Gastroenterol. 2014 Nov 14;20(42):15941-2.

doi: 10.3748/wjg.v20.i42.15941. PubMed PMID: 25400483; PubMed Central PMCID:

PMC4229564.

327: Palmiere C, Egger C, Grabherr S, Jaton-Ogay K, Greub G. Postmortem

angiography using femoral cannulation and postmortem microbiology. Int J Legal

Med. 2015 Jul;129(4):861-7. doi: 10.1007/s00414-014-1099-5. Epub 2014 Nov 8.

PubMed PMID: 25381195.

328: Gürol G, Çiftci İH, Terizi HA, Atasoy AR, Ozbek A, Köroğlu M. Are there

standardized cutoff values for neutrophil-lymphocyte ratios in bacteremia or

sepsis? J Microbiol Biotechnol. 2015 Apr;25(4):521-5. PubMed PMID: 25341467.

329: Bloos F. Clinical diagnosis of sepsis and the combined use of biomarkers and

culture- and non-culture-based assays. Methods Mol Biol. 2015;1237:247-60. doi:

10.1007/978-1-4939-1776-1_19. PubMed PMID: 25319792.

330: Vincent JL, Van Nuffelen M, Lelubre C. Host response biomarkers in sepsis:

the role of procalcitonin. Methods Mol Biol. 2015;1237:213-24. doi:

10.1007/978-1-4939-1776-1_16. PubMed PMID: 25319789.

331: Mauri T, Coppadoro A, Bombino M, Bellani G, Zambelli V, Fornari C, Berra L,

Bittner EA, Schmidt U, Sironi M, Bottazzi B, Brambilla P, Mantovani A, Pesenti A.

Alveolar pentraxin 3 as an early marker of microbiologically confirmed pneumonia:

a threshold-finding prospective observational study. Crit Care. 2014 Oct

15;18(5):562. doi: 10.1186/s13054-014-0562-5. PubMed PMID: 25314919; PubMed

Central PMCID: PMC4219103.

332: Iarustovskiĭ MB, Abramian MV, Krotenko NP, Popov DA, Pliushch MG, Nazarova

EI, Gordeev SL. [Experience of using endotoxin selective adsorption in patients

with severe sepsis after open-heart surgery]. Anesteziol Reanimatol. 2014

May-Jun;(3):39-46. Russian. PubMed PMID: 25306683.

333: Cruz-Lagunas A, Jiménez-Alvarez L, Ramírez G, Mendoza-Milla C, García-Sancho

MC, Avila-Moreno F, Zamudio P, Urrea F, Ortiz-Quintero B, Campos-Toscuento VL,

Morán J, Barrera AA, Martínez-Briseño D, Fernández-Plata R, Sierra-Vargas MP,

Muñoz-Perea C, Illescas-Flores S, Bautista E, Suratt BT, Pérez-Padilla JR, Zuñiga

J. Obesity and pro-inflammatory mediators are associated with acute kidney injury

in patients with A/H1N1 influenza and acute respiratory distress syndrome. Exp

Mol Pathol. 2014 Dec;97(3):453-7. doi: 10.1016/j.yexmp.2014.10.006. Epub 2014 Oct

8. PubMed PMID: 25305354.

334: Zhang Y, Zhou L. [Diagnostic value of C-reactive protein and procalcitonin

for bacterial infection in acute exacerbations of chronic obstructive pulmonary

disease]. Zhong Nan Da Xue Xue Bao Yi Xue Ban. 2014 Sep;39(9):939-43. doi:

10.11817/j.issn.1672-7347.2014.09.013. Chinese. PubMed PMID: 25269493.

335: Anand D, Das S, Bhargava S, Srivastava LM, Garg A, Tyagi N, Taneja S, Ray S.

Procalcitonin as a rapid diagnostic biomarker to differentiate between

culture-negative bacterial sepsis and systemic inflammatory response syndrome: a

prospective, observational, cohort study. J Crit Care. 2015 Feb;30(1):218.e7-12.

doi: 10.1016/j.jcrc.2014.08.017. Epub 2014 Sep 6. PubMed PMID: 25263339.

336: Stubljar D, Skvarc M. Expression of CD64 on neutrophils can be used to

predict the severity of bloodstream infection before broad range 16S rRNA PCR.

Folia Microbiol (Praha). 2015 Mar;60(2):111-8. doi: 10.1007/s12223-014-0346-y.

Epub 2014 Sep 26. PubMed PMID: 25253263.

337: Friend KE, Burgess JN, Britt RC, Collins JN, Weireter LN, Novosel TJ, Britt

LD. Procalcitonin elevation suggests a septic source. Am Surg. 2014

Sep;80(9):906-9. PubMed PMID: 25197879.

338: Johansson N, Kalin M, Backman-Johansson C, Larsson A, Nilsson K, Hedlund J.

Procalcitonin levels in community-acquired pneumonia - correlation with aetiology

and severity. Scand J Infect Dis. 2014 Nov;46(11):787-91. doi:

10.3109/00365548.2014.945955. Epub 2014 Sep 8. PubMed PMID: 25195651.

339: Leli C, Cardaccia A, Ferranti M, Cesarini A, D'Alò F, Ferri C, Cenci E,

Mencacci A. Procalcitonin better than C-reactive protein, erythrocyte

sedimentation rate, and white blood cell count in predicting DNAemia in patients

with sepsis. Scand J Infect Dis. 2014 Nov;46(11):745-52. doi:

10.3109/00365548.2014.936493. Epub 2014 Sep 8. PubMed PMID: 25195647.

340: Sakata KK, Grys TE, Chang YH, Vikram HR, Blair JE. Serum procalcitonin

levels in patients with primary pulmonary coccidioidomycosis. Ann Am Thorac Soc.

2014 Oct;11(8):1239-43. doi: 10.1513/AnnalsATS.201404-180BC. PubMed PMID:

25168059.

341: Marciano S, Haddad L, Martínez AP, Posadas ML, Piñero F, Mora GJ, Guerrero

LN, Ridruejo E, Mandó OG, Giunta DH, Gadano AC. Ultra-sensitive procalcitonin may

help rule out bacterial infections in patients with cirrhosis. Ann Hepatol. 2014

Sep-Oct;13(5):541-7. PubMed PMID: 25152987.

342: Oktay B, Ozgur YA, Metin K, Naim A, Zeliha A, Mehmet Y, Ayse K, Sebahat B,

Bora A, Yasar N. Serum hsCRP and procalcitonin levels in dyspeptic patients

infected with CagA-positive Helicobacter pylori. J Clin Lab Anal. 2015

Nov;29(6):469-73. doi: 10.1002/jcla.21795. Epub 2014 Aug 17. PubMed PMID:

25132401.

343: Rogina P, Skvarc M, Stubljar D, Kofol R, Kaasch A. Diagnostic utility of

broad range bacterial 16S rRNA gene PCR with degradation of human and free

bacterial DNA in bloodstream infection is more sensitive than an in-house

developed PCR without degradation of human and free bacterial DNA. Mediators

Inflamm. 2014;2014:108592. doi: 10.1155/2014/108592. Epub 2014 Jul 9. PubMed

PMID: 25120284; PubMed Central PMCID: PMC4120914.

344: Albrich WC, Madhi SA, Adrian PV, van Niekerk N, Telles JN, Ebrahim N,

Messaoudi M, Paranhos-Baccalà G, Giersdorf S, Vernet G, Mueller B, Klugman KP.

Pneumococcal colonisation density: a new marker for disease severity in

HIV-infected adults with pneumonia. BMJ Open. 2014 Aug 11;4(8):e005953. doi:

10.1136/bmjopen-2014-005953. PubMed PMID: 25113557; PubMed Central PMCID:

PMC4127937.

345: Hoenigl M, Wagner J, Raggam RB, Prueller F, Prattes J, Eigl S, Leitner E,

Hönigl K, Valentin T, Zollner-Schwetz I, Grisold AJ, Krause R. Characteristics of

hospital-acquired and community-onset blood stream infections, South-East

Austria. PLoS One. 2014 Aug 8;9(8):e104702. doi: 10.1371/journal.pone.0104702.

eCollection 2014. PubMed PMID: 25105287; PubMed Central PMCID: PMC4126753.

346: Nijman RG, Moll HA, Smit FJ, Gervaix A, Weerkamp F, Vergouwe Y, de Rijke YB,

Oostenbrink R. C-reactive protein, procalcitonin and the lab-score for detecting

serious bacterial infections in febrile children at the emergency department: a

prospective observational study. Pediatr Infect Dis J. 2014 Nov;33(11):e273-9.

doi: 10.1097/INF.0000000000000466. PubMed PMID: 25093971.

347: Bello S, Mincholé E, Fandos S, Lasierra AB, Ruiz MA, Simon AL, Panadero C,

Lapresta C, Menendez R, Torres A. Inflammatory response in mixed viral-bacterial

community-acquired pneumonia. BMC Pulm Med. 2014 Jul 29;14:123. doi:

10.1186/1471-2466-14-123. PubMed PMID: 25073709; PubMed Central PMCID:

PMC4118651.

348: Shao F, Yang D. [Impact of microbial immune enteral nutrition on

postoperative insulin resistance and infectious complication of patients with

abdominal infection]. Zhonghua Wei Chang Wai Ke Za Zhi. 2014 Jul;17(7):676-9.

Chinese. PubMed PMID: 25070447.

349: Gorski A, Hamouda K, Özkur M, Leistner M, Sommer SP, Leyh R, Schimmer C.

Cardiac surgery antibiotic prophylaxis and calculated empiric antibiotic therapy.

Asian Cardiovasc Thorac Ann. 2015 Mar;23(3):282-8. doi: 10.1177/0218492314546028.

Epub 2014 Jul 24. PubMed PMID: 25061221.

350: Popov DA, Ovseenko ST, Vostrikova TY. [Procalcitonin as a predictor of

bacteremia in postoperative cardiosurgery patients]. Anesteziol Reanimatol. 2014

Mar-Apr;(2):4-9. Russian. PubMed PMID: 25055485.

351: van der Starre WE, Zunder SM, Vollaard AM, van Nieuwkoop C, Stalenhoef JE,

Delfos NM, Van't Wout JW, Spelt IC, Blom JW, Leyten EM, Koster T, Ablij HC, van

Dissel JT. Prognostic value of pro-adrenomedullin, procalcitonin and C-reactive

protein in predicting outcome of febrile urinary tract infection. Clin Microbiol

Infect. 2014 Oct;20(10):1048-54. doi: 10.1111/1469-0691.12645. PubMed PMID:

25039648.

352: Aimoto M, Koh H, Katayama T, Okamura H, Yoshimura T, Koh S, Nanno S,

Nishimoto M, Hirose A, Nakamae M, Nakane T, Nakamae H, Kakeya H, Hino M.

Diagnostic performance of serum high-sensitivity procalcitonin and serum

C-reactive protein tests for detecting bacterial infection in febrile

neutropenia. Infection. 2014 Dec;42(6):971-9. doi: 10.1007/s15010-014-0657-6.

Epub 2014 Jul 20. PubMed PMID: 25038889.

353: Jain S, Sinha S, Sharma SK, Samantaray JC, Aggrawal P, Vikram NK, Biswas A,

Sood S, Goel M, Das M, Vishnubhatla S, Khan N. Procalcitonin as a prognostic

marker for sepsis: a prospective observational study. BMC Res Notes. 2014 Jul

17;7:458. doi: 10.1186/1756-0500-7-458. PubMed PMID: 25034373; PubMed Central

PMCID: PMC4105100.

354: Nakajima A, Yazawa J, Sugiki D, Mizuguchi M, Sagara H, Fujisiro M, Shibazaki

M, Hitani A, To M, Haruki K. Clinical utility of procalcitonin as a marker of

sepsis: a potential predictor of causative pathogens. Intern Med.

2014;53(14):1497-503. Epub 2014 Jul 15. PubMed PMID: 25030560.

355: Hoshina T, Nanishi E, Kanno S, Nishio H, Kusuhara K, Hara T. The utility of

biomarkers in differentiating bacterial from non-bacterial lower respiratory

tract infection in hospitalized children: difference of the diagnostic

performance between acute pneumonia and bronchitis. J Infect Chemother. 2014

Oct;20(10):616-20. doi: 10.1016/j.jiac.2014.06.003. Epub 2014 Jul 11. PubMed

PMID: 25027057.

356: Vassiliou AG, Mastora Z, Orfanos SE, Jahaj E, Maniatis NA, Koutsoukou A,

Armaganidis A, Kotanidou A. Elevated biomarkers of endothelial

dysfunction/activation at ICU admission are associated with sepsis development.

Cytokine. 2014 Oct;69(2):240-7. doi: 10.1016/j.cyto.2014.06.010. Epub 2014 Jul

12. PubMed PMID: 25016133.

357: Karagoz E, Ulcay A. Re: Usefulness of serum procalcitonin level for

prediction of vesicoureteral reflux in pediatric urinary tract infection. Iran J

Kidney Dis. 2014 Jul;8(4):347-8. PubMed PMID: 25001146.

358: Lin KH, Wang FL, Wu MS, Jiang BY, Kao WL, Chao HY, Wu JY, Lee CC. Serum

procalcitonin and C-reactive protein levels as markers of bacterial infection in

patients with liver cirrhosis: a systematic review and meta-analysis. Diagn

Microbiol Infect Dis. 2014 Sep;80(1):72-8. doi:

10.1016/j.diagmicrobio.2014.03.029. Epub 2014 May 21. Review. PubMed PMID:

24974271.

359: Karakas A, Arslan E, Cakmak T, Aydin I, Akgul EO, Demirbas S. Predictive

Value of Soluble CD14, Interleukin-6 and Procalcitonin For Lower Extremity

Amputation in People with Diabetes with Foot Ulcers: A Pilot Study. Pak J Med

Sci. 2014 May;30(3):578-82. doi: 10.12669/pjms.303.4575. PubMed PMID: 24948983;

PubMed Central PMCID: PMC4048510.

360: Koufadaki AM, Karavanaki KA, Soldatou A, Tsentidis Ch, Sourani MP, Sdogou T,

Haliotis FA, Stefanidis CJ. Clinical and laboratory indices of severe renal

lesions in children with febrile urinary tract infection. Acta Paediatr. 2014

Sep;103(9):e404-9. doi: 10.1111/apa.12706. Epub 2014 Jul 1. PubMed PMID:

24862642.

361: Avdic E, Carroll KC. The role of the microbiology laboratory in

antimicrobial stewardship programs. Infect Dis Clin North Am. 2014

Jun;28(2):215-35. doi: 10.1016/j.idc.2014.01.002. Epub 2014 Apr 3. Review. PubMed

PMID: 24857389.

362: Choi SH, Hong SB, Hong HL, Kim SH, Huh JW, Sung H, Lee SO, Kim MN, Jeong JY,

Lim CM, Kim YS, Woo JH, Koh Y. Usefulness of cellular analysis of bronchoalveolar

lavage fluid for predicting the etiology of pneumonia in critically ill patients.

PLoS One. 2014 May 13;9(5):e97346. doi: 10.1371/journal.pone.0097346. eCollection

2014. PubMed PMID: 24824328; PubMed Central PMCID: PMC4019586.

363: Kapur N, Mackay IM, Sloots TP, Masters IB, Chang AB. Respiratory viruses in

exacerbations of non-cystic fibrosis bronchiectasis in children. Arch Dis Child.

2014 Aug;99(8):749-53. doi: 10.1136/archdischild-2013-305147. Epub 2014 May 12.

PubMed PMID: 24819370.

364: Yaroustovsky M, Abramyan M, Krotenko N, Popov D, Plyushch M, Popok Z.

Endotoxin adsorption using polymyxin B immobilized fiber cartridges in severe

sepsis patients following cardiac surgery. Int J Artif Organs. 2014

Apr;37(4):299-307. doi: 10.5301/ijao.5000322. Epub 2014 Apr 16. PubMed PMID:

24811184.

365: Lesińska M, Hartleb M, Gutkowski K, Nowakowska-Duława E. Procalcitonin and

macrophage inflammatory protein-1 beta (MIP-1β) in serum and peritoneal fluid of

patients with decompensated cirrhosis and spontaneous bacterial peritonitis. Adv

Med Sci. 2014 Mar;59(1):52-6. doi: 10.1016/j.advms.2013.07.006. Epub 2014 Mar 21.

PubMed PMID: 24797975.

366: Konstantinidis T, Cassimos D, Gioka T, Tsigalou C, Parasidis T,

Alexandropoulou I, Nikolaidis C, Kampouromiti G, Constantinidis T, Chatzimichael

A, Panopoulou M. Can Procalcitonin in Cerebrospinal Fluid be a Diagnostic Tool

for Meningitis? J Clin Lab Anal. 2015 May;29(3):169-74. doi: 10.1002/jcla.21746.

Epub 2014 May 5. PubMed PMID: 24797775.

367: Polewczyk A, Janion M, Podlaski R, Kutarski A. Clinical manifestations of

lead-dependent infective endocarditis: analysis of 414 cases. Eur J Clin

Microbiol Infect Dis. 2014 Sep;33(9):1601-8. doi: 10.1007/s10096-014-2117-8. Epub

2014 May 3. PubMed PMID: 24791953; PubMed Central PMCID: PMC4129226.

368: Karhu J, Ala-Kokko TI, Vuorinen T, Ohtonen P, Syrjälä H. Lower respiratory

tract virus findings in mechanically ventilated patients with severe

community-acquired pneumonia. Clin Infect Dis. 2014 Jul 1;59(1):62-70. doi:

10.1093/cid/ciu237. Epub 2014 Apr 11. PubMed PMID: 24729498; PubMed Central

PMCID: PMC4305142.

369: Dessì A, Corsello G, Stronati M, Gazzolo D, Caboni P, Carboni R, Fanos V.

New diagnostic possibilities in systemic neonatal infections: metabolomics. Early

Hum Dev. 2014 Mar;90 Suppl 1:S19-21. doi: 10.1016/S0378-3782(14)70007-6. Review.

PubMed PMID: 24709449.

370: Bodmann KF. [Diagnostic markers and assessment of efficacy of antibacterial

therapy]. Med Klin Intensivmed Notfmed. 2014 Apr;109(3):187-90. doi:

10.1007/s00063-013-0312-5. Epub 2014 Apr 5. Review. German. PubMed PMID:

24699883.

371: Woei-A-Jin FJ, van der Starre WE, Tesselaar ME, Garcia Rodriguez P, van

Nieuwkoop C, Bertina RM, van Dissel JT, Osanto S. Procoagulant tissue factor

activity on microparticles is associated with disease severity and bacteremia in

febrile urinary tract infections. Thromb Res. 2014 May;133(5):799-803. doi:

10.1016/j.thromres.2014.03.007. Epub 2014 Mar 6. PubMed PMID: 24657035.

372: Gong YX, Cui SN, Li L, Wang MM, Guo N. [Diagnostic value of human neutrophil

peptide in spontaneous bacterial peritonitis]. Zhonghua Gan Zang Bing Za Zhi.

2013 Dec;21(12):944-8. doi: 10.3760/cma.j.issn.1007-3418.2013.12.014. Chinese.

PubMed PMID: 24636299.

373: Chen A, Fei J, Deirmegian C. Diagnosis of periprosthetic infection: novel

developments. J Knee Surg. 2014 Aug;27(4):259-65. doi: 10.1055/s-0034-1371768.

Epub 2014 Mar 12. PubMed PMID: 24622913.

374: Dimitri E, Amialyanchyk V, Bordicchia E, D'Angelo G. [Diagnostic approach to

phlogoses: the validity of procalcitonin]. Pediatr Med Chir. 2013

Nov-Dec;35(6):276-80. Italian. PubMed PMID: 24620556.

375: Remington LT, Sligl WI. Community-acquired pneumonia. Curr Opin Pulm Med.

2014 May;20(3):215-24. doi: 10.1097/MCP.0000000000000052. Review. PubMed PMID:

24614242.

376: Christensen AM, Thomsen MK, Ovesen T, Klug TE. Are procalcitonin or other

infection markers useful in the detection of group A streptococcal acute

tonsillitis? Scand J Infect Dis. 2014 May;46(5):376-83. doi:

10.3109/00365548.2014.885656. Epub 2014 Mar 10. PubMed PMID: 24606046.

377: Hamaguchi S, Hirose T, Matsumoto N, Akeda Y, Irisawa T, Seki M, Hosotsubo H,

Yamamoto K, Tasaki O, Oishi K, Shimazu T, Tomono K. Neutrophil extracellular

traps in bronchial aspirates: a quantitative analysis. Eur Respir J. 2014

Jun;43(6):1709-18. doi: 10.1183/09031936.00139813. Epub 2014 Mar 6. PubMed PMID:

24603817.

378: Chen W, Zhao L, Niu S, Wang S, Sheng B, Zhen J, Gu X, Lyu C. [The diagnostic

value of different pro-inflammatory factor in early diagnosis of sepsis in

patients with bloodstream infection]. Zhonghua Wei Zhong Bing Ji Jiu Yi Xue. 2014

Mar;26(3):165-70. doi: 10.3760/cma.j.issn.2095-4352.2014.03.008. Chinese. PubMed

PMID: 24598289.

379: Randau TM, Friedrich MJ, Wimmer MD, Reichert B, Kuberra D, Stoffel-Wagner B,

Limmer A, Wirtz DC, Gravius S. Interleukin-6 in serum and in synovial fluid

enhances the differentiation between periprosthetic joint infection and aseptic

loosening. PLoS One. 2014 Feb 21;9(2):e89045. doi: 10.1371/journal.pone.0089045.

eCollection 2014. PubMed PMID: 24586496; PubMed Central PMCID: PMC3931684.

380: Wu J, Jiang F, Zeng T, Xu H, Lei Y, Zhong S, Zhou Z, Ren H. [Role of serum

procalcitonin assay for diagnosis of spontaneous bacterial peritonitis in

end-stage liver diseases]. Zhongguo Yi Xue Ke Xue Yuan Xue Bao. 2014

Feb;36(1):37-41. doi: 10.3881/j.issn.1000-503X.2014.01.007. Chinese. PubMed PMID:

24581126.

381: Romualdo LG, Torrella PE, González MV, Sánchez RJ, Holgado AH, Freire AO,

Acebes SR, Otón MD. Diagnostic accuracy of presepsin (soluble CD14 subtype) for

prediction of bacteremia in patients with systemic inflammatory response syndrome

in the Emergency Department. Clin Biochem. 2014 May;47(7-8):505-8. doi:

10.1016/j.clinbiochem.2014.02.011. Epub 2014 Feb 20. PubMed PMID: 24560955.

382: Cortegiani A, Russotto V, Montalto F, Foresta G, Accurso G, Palmeri C,

Raineri SM, Giarratano A. Procalcitonin as a marker of Candida species detection

by blood culture and polymerase chain reaction in septic patients. BMC

Anesthesiol. 2014 Feb 21;14:9. doi: 10.1186/1471-2253-14-9. PubMed PMID:

24559080; PubMed Central PMCID: PMC3936855.

383: Leli C, Cardaccia A, D'Alò F, Ferri C, Bistoni F, Mencacci A. A prediction

model for real-time PCR results in blood samples from febrile patients with

suspected sepsis. J Med Microbiol. 2014 May;63(Pt 5):649-58. doi:

10.1099/jmm.0.064097-0. Epub 2014 Feb 12. PubMed PMID: 24523157.

384: Lippi G, Danese E, Cervellin G, Montagnana M. Laboratory diagnostics of

spontaneous bacterial peritonitis. Clin Chim Acta. 2014 Mar 20;430:164-70. doi:

10.1016/j.cca.2014.01.023. Epub 2014 Feb 6. Review. PubMed PMID: 24508989.

385: Song JY, Eun BW, Nahm MH. Diagnosis of pneumococcal pneumonia: current

pitfalls and the way forward. Infect Chemother. 2013 Dec;45(4):351-66. doi:

10.3947/ic.2013.45.4.351. Epub 2013 Dec 27. Review. PubMed PMID: 24475349; PubMed

Central PMCID: PMC3902818.

386: Loonen AJ, de Jager CP, Tosserams J, Kusters R, Hilbink M, Wever PC, van den

Brule AJ. Biomarkers and molecular analysis to improve bloodstream infection

diagnostics in an emergency care unit. PLoS One. 2014 Jan 27;9(1):e87315. doi:

10.1371/journal.pone.0087315. eCollection 2014. PubMed PMID: 24475269; PubMed

Central PMCID: PMC3903623.

387: Hatipoglu M, Ulcay A, Karagoz E, Turhan V. Role of procalcitonin in the

diagnosis of infective endocarditis. Am J Emerg Med. 2014 Mar;32(3):285. doi:

10.1016/j.ajem.2013.12.004. Epub 2013 Dec 7. PubMed PMID: 24418442.

388: Ikeda T, Ikeda K, Suda S, Ueno T. Usefulness of the endotoxin activity assay

as a biomarker to assess the severity of endotoxemia in critically ill patients.

Innate Immun. 2014 Nov;20(8):881-7. doi: 10.1177/1753425913516885. Epub 2014 Jan

7. PubMed PMID: 24398861.

389: Simonsen KA, Anderson-Berry AL, Delair SF, Davies HD. Early-onset neonatal

sepsis. Clin Microbiol Rev. 2014 Jan;27(1):21-47. doi: 10.1128/CMR.00031-13.

Review. PubMed PMID: 24396135; PubMed Central PMCID: PMC3910904.

390: Niu WY, Wan YG, Li MY, Wu ZX, Zhang LG, Wang JX. The diagnostic value of

serum procalcitonin, IL-10 and C-reactive protein in community acquired pneumonia

and tuberculosis. Eur Rev Med Pharmacol Sci. 2013 Dec;17(24):3329-33. PubMed

PMID: 24379064.

391: Picot VS, Bénet T, Messaoudi M, Telles JN, Chou M, Eap T, Wang J, Shen K,

Pape JW, Rouzier V, Awasthi S, Pandey N, Bavdekar A, Sanghvi S, Robinson A,

Contamin B, Hoffmann J, Sylla M, Diallo S, Nymadawa P, Dash-Yandag B, Russomando

G, Basualdo W, Siqueira MM, Barreto P, Komurian-Pradel F, Vernet G, Endtz H,

Vanhems P, Paranhos-Baccalà G; pneumonia GABRIEL network. Multicenter

case-control study protocol of pneumonia etiology in children: Global Approach to

Biological Research, Infectious diseases and Epidemics in Low-income countries

(GABRIEL network). BMC Infect Dis. 2014 Dec 10;14:635. doi:

10.1186/s12879-014-0635-8. PubMed PMID: 25927410; PubMed Central PMCID:

PMC4272811.

392: Oksuz L, Somer A, Salman N, Erk O, Gurler N. Procalcitonin and C-reactive

protein in differantiating to contamination from bacteremia. Braz J Microbiol.

2015 Mar 4;45(4):1415-21. eCollection 2014. PubMed PMID: 25763049; PubMed Central

PMCID: PMC4323318.

393: Nakadai H, Yoshizawa S, Miyamoto H, Ikeda Y, Akizuki S, Ohnishi A. [Clinical

verification of serum procalcitonin measurement in sepsis]. Rinsho Byori. 2013

Sep;61(9):781-6. Japanese. PubMed PMID: 24369589.

394: Ratzinger F, Schuardt M, Eichbichler K, Tsirkinidou I, Bauer M, Haslacher H,

Mitteregger D, Binder M, Burgmann H. Utility of sepsis biomarkers and the

infection probability score to discriminate sepsis and systemic inflammatory

response syndrome in standard care patients. PLoS One. 2013 Dec 11;8(12):e82946.

doi: 10.1371/journal.pone.0082946. eCollection 2013. PubMed PMID: 24349403;

PubMed Central PMCID: PMC3859603.

395: Boyles TH, Whitelaw A, Bamford C, Moodley M, Bonorchis K, Morris V, Rawoot

N, Naicker V, Lusakiewicz I, Black J, Stead D, Lesosky M, Raubenheimer P, Dlamini

S, Mendelson M. Antibiotic stewardship ward rounds and a dedicated prescription

chart reduce antibiotic consumption and pharmacy costs without affecting

inpatient mortality or re-admission rates. PLoS One. 2013 Dec 9;8(12):e79747.

doi: 10.1371/journal.pone.0079747. eCollection 2013. PubMed PMID: 24348995;

PubMed Central PMCID: PMC3857167.

396: Hattori T, Nishiyama H, Kato H, Ikegami S, Nagayama M, Asami S, Usami M,

Suzuki M, Murakami I, Minoshima M, Yamagishi H, Yuasa N. Clinical value of

procalcitonin for patients with suspected bloodstream infection. Am J Clin

Pathol. 2014 Jan;141(1):43-51. doi: 10.1309/AJCP4GV7ZFDTANGC. PubMed PMID:

24343736.

397: Oludag T, Gode F, Caglayan E, Saatli B, Okyay RE, Altunyurt S. Value of

maternal procalcitonin levels for predicting subclinical intra-amniotic infection

in preterm premature rupture of membranes. J Obstet Gynaecol Res. 2014

Apr;40(4):954-60. doi: 10.1111/jog.12273. Epub 2013 Dec 10. PubMed PMID:

24320627.

398: Piatek J, Gibas-Dorna M, Budzynski W, Krauss H, Marzec E, Olszewski J,

Zukiewicz-Sobczak W. Urinary tract infection during pregnancy affects the level

of leptin, ghrelin and insulin in maternal and placental blood. Scand J Clin Lab

Invest. 2014 Mar;74(2):126-31. doi: 10.3109/00365513.2013.862848. Epub 2013 Dec

9. PubMed PMID: 24313547.

399: Saeed K, Ahmad N, Dryden M. The value of procalcitonin measurement in

localized skin and skin structure infection, diabetic foot infections, septic

arthritis and osteomyelitis. Expert Rev Mol Diagn. 2014 Jan;14(1):47-54. doi:

10.1586/14737159.2014.864238. Epub 2013 Dec 2. Review. PubMed PMID: 24308337.

400: Wu X, Wang CQ, Yan XF, Wang AM, He LY, Mi ZH, Yu H. [Clinical features and

molecular characteristics of methicillin-resistant Staphylococcus aureus in

children]. Zhonghua Er Ke Za Zhi. 2013 Jul;51(7):512-7. Chinese. PubMed PMID:

24267132.

401: Livermore DM, Wain J. Revolutionising bacteriology to improve treatment

outcomes and antibiotic stewardship. Infect Chemother. 2013 Mar;45(1):1-10. doi:

10.3947/ic.2013.45.1.1. Epub 2013 Mar 29. Review. PubMed PMID: 24265945; PubMed

Central PMCID: PMC3780945.

402: Musher DM, Bebko SP, Roig IL. Serum procalcitonin level, viral polymerase

chain reaction analysis, and lower respiratory tract infection. J Infect Dis.

2014 Feb 15;209(4):631-3. doi: 10.1093/infdis/jit579. Epub 2013 Nov 11. PubMed

PMID: 24218499.

403: Pereira JM, Teixeira-Pinto A, Basílio C, Sousa-Dias C, Mergulhão P, Paiva

JA. Can we predict pneumococcal bacteremia in patients with severe

community-acquired pneumonia? J Crit Care. 2013 Dec;28(6):970-4. doi:

10.1016/j.jcrc.2013.04.016. PubMed PMID: 24216331.

404: Shah BA, Padbury JF. Neonatal sepsis: an old problem with new insights.

Virulence. 2014 Jan 1;5(1):170-8. doi: 10.4161/viru.26906. Epub 2013 Nov 1.

Review. PubMed PMID: 24185532; PubMed Central PMCID: PMC3916371.

405: Duppenthaler A. [Fever without a localizing source in infants]. Ther Umsch.

2013 Nov;70(11):661-7. doi: 10.1024/0040-5930/a000462. Review. German. PubMed

PMID: 24168800.

406: Fu Y, Jiang H, Li LX, Chen J, Zhang JL, Wang LL. [Application value of

procalcitonin and immune inflammatory factors for prediction of bacteraemia in

patients with hematologic malignancy combined with febrile neutropenia]. Zhongguo

Shi Yan Xue Ye Xue Za Zhi. 2013 Oct;21(5):1296-300. doi:

10.7534/j.issn.1009-2137.2013.05.042. Chinese. PubMed PMID: 24156453.

407: Contou D, d'Ythurbide G, Messika J, Ridel C, Parrot A, Djibré M, Hertig A,

Rondeau E, Fartoukh M. Description and predictive factors of infection in

patients with chronic kidney disease admitted to the critical care unit. J

Infect. 2014 Feb;68(2):105-15. doi: 10.1016/j.jinf.2013.10.003. Epub 2013 Oct 15.

PubMed PMID: 24140065.

408: Julián-Jiménez A, Timón Zapata J, Laserna Mendieta EJ, Parejo Miguez R,

Flores Chacartegui M, Gallardo Schall P. [Ability of procalcitonin to predict

bacteremia in patients with community acquired pneumonia]. Med Clin (Barc). 2014

Apr 7;142(7):285-92. doi: 10.1016/j.medcli.2013.05.046. Epub 2013 Oct 8. Spanish.

PubMed PMID: 24120103.

409: Parmentier-Decrucq E, Nseir S, Makris D, Desrousseaux B, Soudan B, Favory R,

Mathieu D. Accuracy of leptin serum level in diagnosing ventilator-associated

pneumonia: a case-control study. Minerva Anestesiol. 2014 Jan;80(1):39-47. Epub

2013 Oct 9. PubMed PMID: 24107832.

410: Shimizu T, Obata T, Sonoda H, Akabori H, Miyake T, Yamamoto H, Tabata T,

Eguchi Y, Tani T. Diagnostic potential of endotoxin scattering photometry for

sepsis and septic shock. Shock. 2013 Dec;40(6):504-11. doi:

10.1097/SHK.0000000000000056. PubMed PMID: 24089007.

411: Lee CH, Kim J, Park Y, Park YC, Kim Y, Yoon KJ, Uh Y, Lee KA. Delta

neutrophil index discriminates true bacteremia from blood culture contamination.

Clin Chim Acta. 2014 Jan 1;427:11-4. doi: 10.1016/j.cca.2013.09.016. Epub 2013

Sep 28. PubMed PMID: 24080435.

412: Cakır Madenci Ö, Yakupoğlu S, Benzonana N, Yücel N, Akbaba D, Orçun

Kaptanağası A. Evaluation of soluble CD14 subtype (presepsin) in burn sepsis.

Burns. 2014 Jun;40(4):664-9. doi: 10.1016/j.burns.2013.08.024. Epub 2013 Sep 26.

PubMed PMID: 24074718.

413: Keramidaris D, Koronakis N, Lagoudianakis EE, Pappas A, Koukoutsis I,

Chrysikos I, Karavitis G, Toutouzas K, Manouras A. Procalcitonin in patients with

colorectal cancer. J BUON. 2013 Jul-Sep;18(3):623-8. PubMed PMID: 24065474.

414: Wang H, Yin F, Shen DX, Zhang YJ, Luo YP, Liu CJ, Wang KF, Zhou G, Ye LY,

Chen G, Wang XN. Predictive value of procalcitonin for excluding bloodstream

infection: results of a retrospective study and utility of a rapid, quantitative

test for procalcitonin. J Int Med Res. 2013 Oct;41(5):1671-81. doi:

10.1177/0300060513497558. Epub 2013 Sep 24. PubMed PMID: 24065454.

415: Kitanovski L, Jazbec J, Hojker S, Derganc M. Diagnostic accuracy of

lipopolysaccharide-binding protein for predicting bacteremia/clinical sepsis in

children with febrile neutropenia: comparison with interleukin-6, procalcitonin,

and C-reactive protein. Support Care Cancer. 2014 Jan;22(1):269-77. doi:

10.1007/s00520-013-1978-1. Epub 2013 Sep 21. PubMed PMID: 24057110.

416: Dabrowski W, Kocki T, Pilat J, Parada-Turska J, Malbrain ML. Changes in

plasma kynurenic acid concentration in septic shock patients undergoing

continuous veno-venous haemofiltration. Inflammation. 2014 Feb;37(1):223-34. doi:

10.1007/s10753-013-9733-9. PubMed PMID: 24043287; PubMed Central PMCID:

PMC3929023.

417: Phua J, Ngerng W, See K, Tay C, Kiong T, Lim H, Chew M, Yip H, Tan A,

Khalizah H, Capistrano R, Lee K, Mukhopadhyay A. Characteristics and outcomes of

culture-negative versus culture-positive severe sepsis. Crit Care. 2013 Sep

12;17(5):R202. doi: 10.1186/cc12896. PubMed PMID: 24028771; PubMed Central PMCID:

PMC4057416.

418: Bilir F, Akdemir N, Ozden S, Cevrioglu AS, Bilir C. Increased serum

procalcitonin levels in pregnant patients with asymptomatic bacteriuria. Ann Clin

Microbiol Antimicrob. 2013 Sep 5;12:25. doi: 10.1186/1476-0711-12-25. PubMed

PMID: 24006912; PubMed Central PMCID: PMC3846744.

419: Gariani K, Drifte G, Dunn-Siegrist I, Pugin J, Jornayvaz FR. Increased FGF21

plasma levels in humans with sepsis and SIRS. Endocr Connect. 2013 Sep

17;2(3):146-53. doi: 10.1530/EC-13-0040. Print 2013. PubMed PMID: 23999826;

PubMed Central PMCID: PMC3845842.

420: Dumea R, Siriopol D, Hogas S, Mititiuc I, Covic A. Procalcitonin: diagnostic

value in systemic infections in chronic kidney disease or renal transplant

patients. Int Urol Nephrol. 2014 Feb;46(2):461-8. doi: 10.1007/s11255-013-0542-8.

Epub 2013 Aug 30. PubMed PMID: 23990496.

421: Calò Carducci FI, Aufiero LR, Folgori L, Vittucci AC, Amodio D, De Luca M,

Li Pira G, Bergamini A, Pontrelli G, Finocchi A, D'Argenio P. Serum soluble ST2

as diagnostic marker of systemic inflammatory reactive syndrome of bacterial

etiology in children. Pediatr Infect Dis J. 2014 Feb;33(2):199-203. doi:

10.1097/INF.0000000000000030. PubMed PMID: 23989103.

422: Cekin Y, Cekin AH, Duman A, Yilmaz U, Yesil B, Yolcular BO. The role of

serum procalcitonin levels in predicting ascitic fluid infection in hospitalized

cirrhotic and non-cirrhotic patients. Int J Med Sci. 2013 Aug 20;10(10):1367-74.

doi: 10.7150/ijms.6014. eCollection 2013. PubMed PMID: 23983598; PubMed Central

PMCID: PMC3752724.

423: Riedel S, Carroll KC. Laboratory detection of sepsis: biomarkers and

molecular approaches. Clin Lab Med. 2013 Sep;33(3):413-37. doi:

10.1016/j.cll.2013.03.006. Review. PubMed PMID: 23931833.

424: Estavoyer JM, Chirouze C, Faucher JF, Floret N, Couetdic G, Leroy J, Hoen B.

Leptospirosis in Franche-Comté (FRANCE): clinical, biological, and therapeutic

data. Med Mal Infect. 2013 Sep;43(9):379-85. doi: 10.1016/j.medmal.2013.06.018.

Epub 2013 Jul 30. PubMed PMID: 23910938.

425: Wong HR, Lindsell CJ, Lahni P, Hart KW, Gibot S. Interleukin 27 as a sepsis

diagnostic biomarker in critically ill adults. Shock. 2013 Nov;40(5):382-6. doi:

10.1097/SHK.0b013e3182a67632. PubMed PMID: 23903853; PubMed Central PMCID:

PMC3800476.

426: O'Brien DJ, Gould IM. Maximizing the impact of antimicrobial stewardship:

the role of diagnostics, national and international efforts. Curr Opin Infect

Dis. 2013 Aug;26(4):352-8. doi: 10.1097/QCO.0b013e3283631046. Review. PubMed

PMID: 23806898.

427: Friederichs J, Hutter M, Hierholzer C, Novotny A, Friess H, Bühren V,

Hungerer S. Procalcitonin ratio as a predictor of successful surgical treatment

of severe necrotizing soft tissue infections. Am J Surg. 2013 Sep;206(3):368-73.

doi: 10.1016/j.amjsurg.2012.11.024. Epub 2013 Jun 25. PubMed PMID: 23806825.

428: Sato M, Matsuyama R, Kadokura T, Mori R, Kumamoto T, Nojiri K, Taniguchi K,

Takeda K, Kubota K, Tanaka K, Endo I. Severity and prognostic assessment of the

endotoxin activity assay in biliary tract infection. J Hepatobiliary Pancreat

Sci. 2014 Feb;21(2):120-7. doi: 10.1002/jhbp.10. Epub 2013 Jun 20. PubMed PMID:

23798326.

429: Ha YE, Kang CI, Wi YM, Chung DR, Kang ES, Lee NY, Song JH, Peck KR.

Diagnostic usefulness of procalcitonin as a marker of bacteremia in patients with

acute pyelonephritis. Scand J Clin Lab Invest. 2013 Aug;73(5):444-8. doi:

10.3109/00365513.2013.803231. Epub 2013 Jun 17. PubMed PMID: 23772894.

430: Kaya S, Köksal I, Menteşe A, Sönmez M, Sümer A, Yıldırım SS, Yılmaz G. The

significance of serum urokinase plasminogen activation receptor (suPAR) in the

diagnosis and follow-up of febrile neutropenic patients with hematologic

malignancies. Int J Infect Dis. 2013 Nov;17(11):e1056-9. doi:

10.1016/j.ijid.2013.04.004. Epub 2013 Jun 3. PubMed PMID: 23742830.

431: Vodnik T, Kaljevic G, Tadic T, Majkic-Singh N. Presepsin (sCD14-ST) in

preoperative diagnosis of abdominal sepsis. Clin Chem Lab Med. 2013

Oct;51(10):2053-62. doi: 10.1515/cclm-2013-0061. PubMed PMID: 23740685.

432: Ohtaki H, Ohkusu K, Ohta H, Miyazaki T, Yonetamari J, Usui T, Mori I, Ito H,

Ishizuka T, Seishima M. A case of sepsis caused by Streptococcus canis in a dog

owner: a first case report of sepsis without dog bite in Japan. J Infect

Chemother. 2013 Dec;19(6):1206-9. doi: 10.1007/s10156-013-0625-6. Epub 2013 Jun

6. PubMed PMID: 23740090.

433: Datsenko BM, Borisenko VB. [Criteria of diagnosis and principles of

treatment of obturation jaundice and its complicated forms--an acute cholangitis

and biliary sepsis]. Klin Khir. 2013 Mar;(3):5-8. Russian. PubMed PMID: 23718023.

434: Marková M, Brodská H, Malíčková K, Válková V, Cetkovský P, Kolář M, Haluzík

M. Substantially elevated C-reactive protein (CRP), together with low levels of

procalcitonin (PCT), contributes to diagnosis of fungal infection in

immunocompromised patients. Support Care Cancer. 2013 Oct;21(10):2733-42. doi:

10.1007/s00520-013-1844-1. Epub 2013 May 28. PubMed PMID: 23712521.

435: Galetto-Lacour A, Alcoba G, Posfay-Barbe KM, Cevey-Macherel M, Gehri M, Ochs

MM, Brookes RH, Siegrist CA, Gervaix A. Elevated inflammatory markers combined

with positive pneumococcal urinary antigen are a good predictor of pneumococcal

community-acquired pneumonia in children. Pediatr Infect Dis J. 2013

Nov;32(11):1175-9. doi: 10.1097/INF.0b013e31829ba62a. PubMed PMID: 23694836.

436: Lee H. Procalcitonin as a biomarker of infectious diseases. Korean J Intern

Med. 2013 May;28(3):285-91. doi: 10.3904/kjim.2013.28.3.285. Epub 2013 May 1.

Review. PubMed PMID: 23682219; PubMed Central PMCID: PMC3654123.

437: Michail M, Jude E, Liaskos C, Karamagiolis S, Makrilakis K, Dimitroulis D,

Michail O, Tentolouris N. The performance of serum inflammatory markers for the

diagnosis and follow-up of patients with osteomyelitis. Int J Low Extrem Wounds.

2013 Jun;12(2):94-9. doi: 10.1177/1534734613486152. Epub 2013 May 9. PubMed PMID:

23667102.

438: Falsey AR, Becker KL, Swinburne AJ, Nylen ES, Formica MA, Hennessey PA,

Criddle MM, Peterson DR, Baran A, Walsh EE. Bacterial complications of

respiratory tract viral illness: a comprehensive evaluation. J Infect Dis. 2013

Aug 1;208(3):432-41. doi: 10.1093/infdis/jit190. Epub 2013 May 9. PubMed PMID:

23661797; PubMed Central PMCID: PMC3699009.

439: Müller Campanile VS, Giannopoulou C, Campanile G, Cancela JA, Mombelli A.

Single or repeated antimicrobial photodynamic therapy as adjunct to ultrasonic

debridement in residual periodontal pockets: clinical, microbiological, and local

biological effects. Lasers Med Sci. 2015 Jan;30(1):27-34. doi:

10.1007/s10103-013-1337-y. Epub 2013 May 10. PubMed PMID: 23660738; PubMed

Central PMCID: PMC4289155.

440: Saeed K, Dryden M, Sitjar A, White G. Measuring synovial fluid procalcitonin

levels in distinguishing cases of septic arthritis, including prosthetic joints,

from other causes of arthritis and aseptic loosening. Infection. 2013

Aug;41(4):845-9. doi: 10.1007/s15010-013-0467-2. Epub 2013 May 4. PubMed PMID:

23645456.

441: Jabłoński S, Kozakiewicz M. Evaluation of recovery in iatrogenic evoked

acute mediatinitis. Inflammation. 2013 Oct;36(5):1055-63. doi:

10.1007/s10753-013-9637-8. PubMed PMID: 23619916; PubMed Central PMCID:

PMC3781308.

442: Magrini L, Travaglino F, Marino R, Ferri E, De Berardinis B, Cardelli P,

Salerno G, Di Somma S. Procalcitonin variations after Emergency Department

admission are highly predictive of hospital mortality in patients with acute

infectious diseases. Eur Rev Med Pharmacol Sci. 2013 Feb;17 Suppl 1:133-42.

PubMed PMID: 23436675.

443: Lederer W, Stichlberger M, Hausdorfer J, Fuchs D, Mutz NJ, Wiedermann FJ.

Alveolar neopterin, procalcitonin, and IL-6 in relation to serum levels and

severity of lung injury in ARDS. Clin Chem Lab Med. 2013 Sep;51(9):e213-5. doi:

10.1515/cclm-2013-0078. PubMed PMID: 23612545.

444: Dimitri E, Amialyanchyk V, D'Angelo G, Bordicchia F, Gabrieli O. [Diagnostic

approach to flogosis: procalcitonin validity]. Minerva Pediatr. 2013

Apr;65(2):167-72. Italian. PubMed PMID: 23612261.

445: Shomali W, Hachem R, Chaftari AM, Bahu R, Helou GE, Jiang Y, Hanania A,

Reitzel R, Raad I. Can procalcitonin differentiate Staphylococcus aureus from

coagulase-negative staphylococci in clustered gram-positive bacteremia? Diagn

Microbiol Infect Dis. 2013 Jun;76(2):158-61. doi:

10.1016/j.diagmicrobio.2013.03.004. Epub 2013 Apr 8. PubMed PMID: 23578976.

446: Haubitz S, Mueller B, Schuetz P. Streamlining antibiotic therapy with

procalcitonin protocols: consensus and controversies. Expert Rev Respir Med. 2013

Apr;7(2):145-57. doi: 10.1586/ers.13.6. Review. PubMed PMID: 23547991.

447: Tuvim MJ, Clement CG, Huang ES, Cote GJ, Evans SE, Lei X, Deftos LJ, Gagel

RF, Dickey BF. Deletion of the gene encoding calcitonin and calcitonin

gene-related peptide α does not affect the outcome of severe infection in mice.

Am J Respir Cell Mol Biol. 2013 Jul;49(1):151-5. doi: 10.1165/rcmb.2012-0489OC.

PubMed PMID: 23526213; PubMed Central PMCID: PMC3727888.

448: Barbier F, Andremont A, Wolff M, Bouadma L. Hospital-acquired pneumonia and

ventilator-associated pneumonia: recent advances in epidemiology and management.

Curr Opin Pulm Med. 2013 May;19(3):216-28. doi: 10.1097/MCP.0b013e32835f27be.

Review. PubMed PMID: 23524477.

449: Musher DM, Roig IL, Cazares G, Stager CE, Logan N, Safar H. Can an etiologic

agent be identified in adults who are hospitalized for community-acquired

pneumonia: results of a one-year study. J Infect. 2013 Jul;67(1):11-8. doi:

10.1016/j.jinf.2013.03.003. Epub 2013 Mar 19. PubMed PMID: 23523447.

450: Drozdov D, Thomer A, Meili M, Schwarz S, Kouegbe RB, Regez K, Guglielmetti

M, Schild U, Conca A, Schäfer P, Reutlinger B, Ottiger C, Buchkremer F, Litke A,

Schuetz P, Huber A, Bürgi U, Fux CA, Bock A, Müller B, Albrich WC; Triple P study

group. Procalcitonin, pyuria and proadrenomedullin in the management of urinary

tract infections--'triple p in uti': study protocol for a randomized controlled

trial. Trials. 2013 Mar 22;14:84. doi: 10.1186/1745-6215-14-84. PubMed PMID:

23522152; PubMed Central PMCID: PMC3614534.

451: Faix JD. Biomarkers of sepsis. Crit Rev Clin Lab Sci. 2013

Jan-Feb;50(1):23-36. doi: 10.3109/10408363.2013.764490. Review. PubMed PMID:

23480440; PubMed Central PMCID: PMC3613962.

452: Tsalkidou EA, Roilides E, Gardikis S, Trypsianis G, Kortsaris A,

Chatzimichael A, Tentes I. Lipopolysaccharide-binding protein: a potential marker

of febrile urinary tract infection in childhood. Pediatr Nephrol. 2013

Jul;28(7):1091-7. doi: 10.1007/s00467-013-2432-9. Epub 2013 Mar 6. PubMed PMID:

23463341.

453: van Bremen T, Drömann D, Luitjens K, Dodt C, Dalhoff K, Goldmann T, Schaaf

B. Triggering receptor expressed on myeloid cells-1 (Trem-1) on blood neutrophils

is associated with cytokine inducibility in human E. coli sepsis. Diagn Pathol.

2013 Feb 15;8:24. doi: 10.1186/1746-1596-8-24. PubMed PMID: 23414215; PubMed

Central PMCID: PMC3584978.

454: Silva JM Jr, dos Santos Sde S. Sepsis in AIDS patients: clinical,

etiological and inflammatory characteristics. J Int AIDS Soc. 2013 Jan

30;16:17344. doi: 10.7448/IAS.16.1.17344. PubMed PMID: 23374857; PubMed Central

PMCID: PMC3564973.

455: Oku E, Nomura K, Nakamura T, Morishige S, Seki R, Imamura R, Hashiguchi M,

Osaki K, Mizuno S, Nagafuji K, Okamura T. [Amebic colitis and liver abscess

complicated by high serum procalcitonin in acute myeloid leukemia]. Kansenshogaku

Zasshi. 2012 Nov;86(6):773-7. Japanese. PubMed PMID: 23367854.

456: Held J, Kohlberger I, Rappold E, Busse Grawitz A, Häcker G. Comparison of

(1->3)-β-D-glucan, mannan/anti-mannan antibodies, and Cand-Tec Candida antigen as

serum biomarkers for candidemia. J Clin Microbiol. 2013 Apr;51(4):1158-64. doi:

10.1128/JCM.02473-12. Epub 2013 Jan 30. PubMed PMID: 23363830; PubMed Central

PMCID: PMC3666776.

457: Mencacci A, Leli C, Cardaccia A, Meucci M, Moretti A, D'Alò F, Farinelli S,

Pagliochini R, Barcaccia M, Bistoni F. Procalcitonin predicts real-time PCR

results in blood samples from patients with suspected sepsis. PLoS One.

2012;7(12):e53279. doi: 10.1371/journal.pone.0053279. Epub 2012 Dec 27. PubMed

PMID: 23300907; PubMed Central PMCID: PMC3531374.

458: Leroy S, Gervaix A. [Procalcitonin, a useful biomarker in pediatric urinary

tract infection]. Arch Pediatr. 2013 Jan;20(1):54-62. doi:

10.1016/j.arcped.2012.10.025. Epub 2012 Nov 27. French. PubMed PMID: 23199564.

459: Meem M, Modak JK, Mortuza R, Morshed M, Islam MS, Saha SK. Biomarkers for

diagnosis of neonatal infections: A systematic analysis of their potential as a

point-of-care diagnostics. J Glob Health. 2011 Dec;1(2):201-9. PubMed PMID:

23198119; PubMed Central PMCID: PMC3484777.

460: Su DH, Zhuo C, Liao K, Cheng WB, Cheng H, Zhao XF. Value of serum

procalcitonin levels in predicting spontaneous bacterial peritonitis.

Hepatogastroenterology. 2013 Jun;60(124):641-6. Review. PubMed PMID: 23159389.

461: Hoenigl M, Raggam RB, Wagner J, Valentin T, Leitner E, Seeber K,

Zollner-Schwetz I, Krammer W, Prüller F, Grisold AJ, Krause R. Diagnostic

accuracy of soluble urokinase plasminogen activator receptor (suPAR) for

prediction of bacteremia in patients with systemic inflammatory response

syndrome. Clin Biochem. 2013 Feb;46(3):225-9. doi:

10.1016/j.clinbiochem.2012.11.004. Epub 2012 Nov 13. PubMed PMID: 23159293.

462: Su LX, Meng K, Zhang X, Wang HJ, Yan P, Jia YH, Feng D, Xie LX. Diagnosing

ventilator-associated pneumonia in critically ill patients with sepsis. Am J Crit

Care. 2012 Nov;21(6):e110-9. doi: 10.4037/ajcc2012732. PubMed PMID: 23117911.

463: Sankar V, Webster NR. Clinical application of sepsis biomarkers. J Anesth.

2013 Apr;27(2):269-83. doi: 10.1007/s00540-012-1502-7. Epub 2012 Oct 30. Review.

PubMed PMID: 23108494.

464: Angeletti S, Battistoni F, Fioravanti M, Bernardini S, Dicuonzo G.

Procalcitonin and mid-regional pro-adrenomedullin test combination in sepsis

diagnosis. Clin Chem Lab Med. 2013 May;51(5):1059-67. PubMed PMID: 23072859.

465: Bloos F, Sachse S, Kortgen A, Pletz MW, Lehmann M, Straube E, Riedemann NC,

Reinhart K, Bauer M. Evaluation of a polymerase chain reaction assay for pathogen

detection in septic patients under routine condition: an observational study.

PLoS One. 2012;7(9):e46003. doi: 10.1371/journal.pone.0046003. Epub 2012 Sep 27.

PubMed PMID: 23029360; PubMed Central PMCID: PMC3459981.

466: Kasem AJ, Bulloch B, Henry M, Shah K, Dalton H. Procalcitonin as a marker of

bacteremia in children with fever and a central venous catheter presenting to the

emergency department. Pediatr Emerg Care. 2012 Oct;28(10):1017-21. doi:

10.1097/PEC.0b013e31826caac2. PubMed PMID: 23023470.

467: Yo CH, Hsieh PS, Lee SH, Wu JY, Chang SS, Tasi KC, Lee CC. Comparison of the

test characteristics of procalcitonin to C-reactive protein and leukocytosis for

the detection of serious bacterial infections in children presenting with fever

without source: a systematic review and meta-analysis. Ann Emerg Med. 2012

Nov;60(5):591-600. doi: 10.1016/j.annemergmed.2012.05.027. Epub 2012 Aug 22.

Review. PubMed PMID: 22921165.

468: ten Oever J, Tromp M, Bleeker-Rovers CP, Joosten LA, Netea MG, Pickkers P,

van de Veerdonk FL. Combination of biomarkers for the discrimination between

bacterial and viral lower respiratory tract infections. J Infect. 2012

Dec;65(6):490-5. doi: 10.1016/j.jinf.2012.08.004. Epub 2012 Aug 13. PubMed PMID:

22898387.

469: León C, Ruiz-Santana S, Saavedra P, Castro C, Ubeda A, Loza A,

Martín-Mazuelos E, Blanco A, Jerez V, Ballús J, Alvarez-Rocha L, Utande-Vázquez

A, Fariñas O. Value of β-D-glucan and Candida albicans germ tube antibody for

discriminating between Candida colonization and invasive candidiasis in patients

with severe abdominal conditions. Intensive Care Med. 2012 Aug;38(8):1315-25.

doi: 10.1007/s00134-012-2616-y. Epub 2012 Jun 30. PubMed PMID: 22752333.

470: Marrie TJ, Costain N, La Scola B, Patrick W, Forgie S, Xu Z, McNeil SA. The

role of atypical pathogens in community-acquired pneumonia. Semin Respir Crit

Care Med. 2012 Jun;33(3):244-56. doi: 10.1055/s-0032-1315636. Epub 2012 Jun 20.

Review. PubMed PMID: 22718210.

471: Riedel S. Procalcitonin and the role of biomarkers in the diagnosis and

management of sepsis. Diagn Microbiol Infect Dis. 2012 Jul;73(3):221-7. doi:

10.1016/j.diagmicrobio.2012.05.002. Review. PubMed PMID: 22704255.

472: Montagna MT, Coretti C, Rella A, Barbuti G, Manca F, Montagna O, Laforgia N,

Caggiano G. The role of procalcitonin in neonatal intensive care unit patients

with candidemia. Folia Microbiol (Praha). 2013 Jan;58(1):27-31. doi:

10.1007/s12223-012-0169-7. Epub 2012 Jun 12. PubMed PMID: 22688898; PubMed

Central PMCID: PMC3521642.

473: Brodská H, Malíčková K, Adámková V, Benáková H, Šťastná MM, Zima T.

Significantly higher procalcitonin levels could differentiate Gram-negative

sepsis from Gram-positive and fungal sepsis. Clin Exp Med. 2013 Aug;13(3):165-70.

doi: 10.1007/s10238-012-0191-8. Epub 2012 May 27. PubMed PMID: 22644264.

474: Mauro MV, Cavalcanti P, Perugini D, Noto A, Sperlì D, Giraldi C. Diagnostic

utility of LightCycler SeptiFast and procalcitonin assays in the diagnosis of

bloodstream infection in immunocompromised patients. Diagn Microbiol Infect Dis.

2012 Aug;73(4):308-11. doi: 10.1016/j.diagmicrobio.2012.04.006. Epub 2012 May 22.

PubMed PMID: 22626731.

475: Biju PG, Garg S, Wang W, Choudhry MA, Kovacs EJ, Fink LM, Hauer-Jensen M.

Procalcitonin as a predictive biomarker for total body irradiation-induced

bacterial load and lethality in mice. Shock. 2012 Aug;38(2):170-6. doi:

10.1097/SHK.0b013e31825b2db3. PubMed PMID: 22576002; PubMed Central PMCID:

PMC3399035.

476: Matera G, Quirino A, Giancotti A, Pulicari MC, Rametti L, Rodríguez ML,

Liberto MC, Focà A. Procalcitonin neutralizes bacterial LPS and reduces

LPS-induced cytokine release in human peripheral blood mononuclear cells. BMC

Microbiol. 2012 May 8;12:68. doi: 10.1186/1471-2180-12-68. PubMed PMID: 22568957;

PubMed Central PMCID: PMC3406977.

477: Sangil A, Calbo E, Robles A, Benet S, Viladot ME, Pascual V, Cuchí E, Pérez

J, Barreiro B, Sánchez B, Torres J, Canales L, De Marcos JA, Garau J. Aetiology

of community-acquired pneumonia among adults in an H1N1 pandemic year: the role

of respiratory viruses. Eur J Clin Microbiol Infect Dis. 2012 Oct;31(10):2765-72.

Epub 2012 May 2. PubMed PMID: 22549730.

478: Nnanna II, Ehis OJ, Sidiquo II, Nnanna IG, Adekunle O. Serum procalcitonin:

Early detection of neonatal bacteremia and septicemia in a tertiary healthcare

facility. N Am J Med Sci. 2011 Mar;3(3):157-60. doi: 10.4297/najms.2011.3157.

PubMed PMID: 22540083; PubMed Central PMCID: PMC3336904.

479: Mian A, Becton D, Saylors R, James L, Tang X, Bhutta A, Prodhan P.

Biomarkers for risk stratification of febrile neutropenia among children with

malignancy: a pilot study. Pediatr Blood Cancer. 2012 Aug;59(2):238-45. doi:

10.1002/pbc.24158. Epub 2012 Apr 25. PubMed PMID: 22535591.

480: Soler N, Esperatti M, Ewig S, Huerta A, Agustí C, Torres A. Sputum

purulence-guided antibiotic use in hospitalised patients with exacerbations of

COPD. Eur Respir J. 2012 Dec;40(6):1344-53. doi: 10.1183/09031936.00150211. Epub

2012 Apr 20. PubMed PMID: 22523352.

481: Bauer TT, Nilius G, Grüning W, Rasche K. [Diagnosis and therapy of COPD

exacerbation]. Med Klin Intensivmed Notfmed. 2012 Apr;107(3):172-8. doi:

10.1007/s00063-011-0065-y. Epub 2012 Apr 4. Review. German. PubMed PMID:

22476704.

482: Schuetz P, Amin DN, Greenwald JL. Role of procalcitonin in managing adult

patients with respiratory tract infections. Chest. 2012 Apr;141(4):1063-1073.

doi: 10.1378/chest.11-2430. Review. PubMed PMID: 22474148.

483: Falsey AR, Becker KL, Swinburne AJ, Nylen ES, Snider RH, Formica MA,

Hennessey PA, Criddle MM, Peterson DR, Walsh EE. Utility of serum procalcitonin

values in patients with acute exacerbations of chronic obstructive pulmonary

disease: a cautionary note. Int J Chron Obstruct Pulmon Dis. 2012;7:127-35. doi:

10.2147/COPD.S29149. Epub 2012 Feb 23. PubMed PMID: 22399852; PubMed Central

PMCID: PMC3292390.

484: Hui C, Neto G, Tsertsvadze A, Yazdi F, Tricco AC, Tsouros S, Skidmore B,

Daniel R. Diagnosis and management of febrile infants (0-3 months). Evid Rep

Technol Assess (Full Rep). 2012 Mar;(205):1-297. Review. PubMed PMID: 24422856;

PubMed Central PMCID: PMC4781391.

485: te Witt R, Hassing RJ, Petit PP, van Belkum A, van Genderen PJ.

Procalcitonin and neopterin levels do not accurately distinguish bacterial from

viral infections in ill-returned travellers with fever. Trans R Soc Trop Med Hyg.

2012 Apr;106(4):264-6. doi: 10.1016/j.trstmh.2012.01.001. Epub 2012 Feb 21.

PubMed PMID: 22357400.

486: Previsdomini M, Gini M, Cerutti B, Dolina M, Perren A. Predictors of

positive blood cultures in critically ill patients: a retrospective evaluation.

Croat Med J. 2012 Feb 15;53(1):30-9. PubMed PMID: 22351576; PubMed Central PMCID:

PMC3284177.

487: Hettwer S, Wilhelm J, Schürmann M, Ebelt H, Hammer D, Amoury M, Hofmann F,

Oehme A, Wilhelms D, Kekulé AS, Klöss T, Werdan K. Microbial diagnostics in

patients with presumed severe infection in the emergency department. Med Klin

Intensivmed Notfmed. 2012 Feb;107(1):53-62. doi: 10.1007/s00063-011-0051-4.

PubMed PMID: 22349478.

488: Lanoix JP, Schmit JL, Douadi Y. Bacterial lung sepsis in patients with

febrile neutropenia. Curr Opin Pulm Med. 2012 May;18(3):175-80. doi:

10.1097/MCP.0b013e328351f8e8. Review. PubMed PMID: 22343426.

489: Watkins RR, Lemonovich TL. Serum procalcitonin in the diagnosis and

management of intra-abdominal infections. Expert Rev Anti Infect Ther. 2012

Feb;10(2):197-205. doi: 10.1586/eri.11.164. Review. PubMed PMID: 22339193.

490: Kim HA, Jeon JY, An JM, Koh BR, Suh CH. C-reactive protein is a more

sensitive and specific marker for diagnosing bacterial infections in systemic

lupus erythematosus compared to S100A8/A9 and procalcitonin. J Rheumatol. 2012

Apr;39(4):728-34. doi: 10.3899/jrheum.111044. Epub 2012 Feb 15. PubMed PMID:

22337236.

491: Kaya S, Yilmaz G, Arslan M, Oztuna F, Ozlu T, Koksal I. Predictive factors

for fatality in pandemic influenza A (H1N1) virus infected patients. Saudi Med J.

2012 Feb;33(2):146-51. PubMed PMID: 22327754.

492: Pieri M, Greco T, De Bonis M, Maj G, Fumagalli L, Zangrillo A, Pappalardo F.

Diagnosis of infection in patients undergoing extracorporeal membrane

oxygenation: a case-control study. J Thorac Cardiovasc Surg. 2012

Jun;143(6):1411-6. doi: 10.1016/j.jtcvs.2012.01.005. Epub 2012 Feb 2. PubMed

PMID: 22305552.

493: Shu CC, Lee LN, Wu MF, Lee CH, Wang JT, Wang JY, Yu CJ; TAMI Group. Use of

soluble triggering receptor expressed on myeloid cells-1 in non-tuberculous

mycobacterial lung disease. Int J Tuberc Lung Dis. 2011 Oct;15(10):1415-20. doi:

10.5588/ijtld.10.0786. PubMed PMID: 22283904.

494: Lipinska-Gediga M, Mierzchala M, Durek G. Pro-atrial natriuretic peptide

(pro-ANP) level in patients with severe sepsis and septic shock: prognostic and

diagnostic significance. Infection. 2012 Jun;40(3):303-9. doi:

10.1007/s15010-011-0235-0. Epub 2012 Jan 12. PubMed PMID: 22237469; PubMed

Central PMCID: PMC3386487.

495: Pasyar N, Alborzi A, Pouladfar GR. Evaluation of serum procalcitonin levels

for diagnosis of secondary bacterial infections in visceral leishmaniasis

patients. Am J Trop Med Hyg. 2012 Jan;86(1):119-21. doi:

10.4269/ajtmh.2012.11-0079. PubMed PMID: 22232461; PubMed Central PMCID:

PMC3247119.

496: Horie M, Ugajin M, Suzuki M, Noguchi S, Tanaka W, Yoshihara H, Kawakami M,

Kichikawa Y, Sakamoto Y. Diagnostic and prognostic value of procalcitonin in

community-acquired pneumonia. Am J Med Sci. 2012 Jan;343(1):30-5. doi:

10.1097/MAJ.0b013e31821d33ef. PubMed PMID: 22207498.

497: Menéndez R, Sahuquillo-Arce JM, Reyes S, Martínez R, Polverino E, Cillóniz

C, Córdoba JG, Montull B, Torres A. Cytokine activation patterns and biomarkers

are influenced by microorganisms in community-acquired pneumonia. Chest. 2012

Jun;141(6):1537-1545. doi: 10.1378/chest.11-1446. Epub 2011 Dec 22. PubMed PMID:

22194589.

498: Papp M, Vitalis Z, Altorjay I, Tornai I, Udvardy M, Harsfalvi J, Vida A,

Kappelmayer J, Lakatos PL, Antal-Szalmas P. Acute phase proteins in the diagnosis

and prediction of cirrhosis associated bacterial infections. Liver Int. 2012

Apr;32(4):603-11. doi: 10.1111/j.1478-3231.2011.02689.x. Epub 2011 Dec 6. PubMed

PMID: 22145664.

499: Lodes U, Bohmeier B, Lippert H, König B, Meyer F. PCR-based rapid sepsis

diagnosis effectively guides clinical treatment in patients with new onset of

SIRS. Langenbecks Arch Surg. 2012 Mar;397(3):447-55. doi:

10.1007/s00423-011-0870-z. Epub 2011 Nov 23. PubMed PMID: 22109826.

500: Sutter R, Tschudin-Sutter S, Grize L, Widmer AF, Marsch S, Rüegg S. Acute

phase proteins and white blood cell levels for prediction of infectious

complications in status epilepticus. Crit Care. 2011;15(6):R274. doi:

10.1186/cc10555. Epub 2011 Nov 18. PubMed PMID: 22099124; PubMed Central PMCID:

PMC3388641.

501: Bello S, Lasierra AB, Mincholé E, Fandos S, Ruiz MA, Vera E, de Pablo F,

Ferrer M, Menendez R, Torres A. Prognostic power of proadrenomedullin in

community-acquired pneumonia is independent of aetiology. Eur Respir J. 2012

May;39(5):1144-55. doi: 10.1183/09031936.00080411. Epub 2011 Nov 10. PubMed PMID:

22075489.

502: Lu Z, Liu Y, Dong YH, Zhan XB, Du YQ, Gao J, Gong YF, Li ZS. Soluble

triggering receptor expressed on myeloid cells in severe acute pancreatitis: a

biological marker of infected necrosis. Intensive Care Med. 2012 Jan;38(1):69-75.

doi: 10.1007/s00134-011-2369-z. Epub 2011 Oct 27. PubMed PMID: 22037716.

503: Lavrentieva A, Papadopoulou S, Kioumis J, Kaimakamis E, Bitzani M. PCT as a

diagnostic and prognostic tool in burn patients. Whether time course has a role

in monitoring sepsis treatment. Burns. 2012 May;38(3):356-63. doi:

10.1016/j.burns.2011.08.021. Epub 2011 Oct 28. PubMed PMID: 22037153.

504: Altunhan H, Annagür A, Örs R, Mehmetoğlu I. Procalcitonin measurement at 24

hours of age may be helpful in the prompt diagnosis of early-onset neonatal

sepsis. Int J Infect Dis. 2011 Dec;15(12):e854-8. doi:

10.1016/j.ijid.2011.09.007. Epub 2011 Oct 22. PubMed PMID: 22019570.

505: Ahn S, Lee YS, Chun YH, Lim KS, Kim W, Lee JL. Predictive factors of

bacteraemia in low-risk patients with febrile neutropenia. Emerg Med J. 2012

Sep;29(9):715-9. doi: 10.1136/emermed-2011-200012. Epub 2011 Oct 19. PubMed PMID:

22011971.

506: Zegleń S, Sioła M, Woźniak-Grygiel E, Łaszewska A, Sindera P, Wojarski J,

Ochman M, Kucewicz E, Karolak W, Szewczyk M, Zembala M. Procalcitonin serum

concentration in lung transplant recipients during mold colonization or

infection. Transplant Proc. 2011 Oct;43(8):3089-91. doi:

10.1016/j.transproceed.2011.08.057. PubMed PMID: 21996233.

507: Pugh R, Grant C, Cooke RP, Dempsey G. Short-course versus prolonged-course

antibiotic therapy for hospital-acquired pneumonia in critically ill adults.

Cochrane Database Syst Rev. 2011 Oct 5;(10):CD007577. doi:

10.1002/14651858.CD007577.pub2. Review. Update in: Cochrane Database Syst Rev.

2015;8:CD007577. PubMed PMID: 21975771.

508: Greinacher A, Friesecke S, Abel P, Dressel A, Stracke S, Fiene M, Ernst F,

Selleng K, Weissenborn K, Schmidt BM, Schiffer M, Felix SB, Lerch MM, Kielstein

JT, Mayerle J. Treatment of severe neurological deficits with IgG depletion

through immunoadsorption in patients with Escherichia coli O104:H4-associated

haemolytic uraemic syndrome: a prospective trial. Lancet. 2011 Sep

24;378(9797):1166-73. doi: 10.1016/S0140-6736(11)61253-1. Epub 2011 Sep 2. PubMed

PMID: 21890192.

509: Amezcua-Guerra LM, Springall R, Arrieta-Alvarado AA, Rodríguez V,

Rivera-Martinez E, Castillo-Martinez D, Bojalil R. C-reactive protein and

complement components but not other acute-phase reactants discriminate between

clinical subsets and organ damage in systemic lupus erythematosus. Clin Lab.

2011;57(7-8):607-13. PubMed PMID: 21888025.

510: Rustici MC, Chiappini E, Salvadori M, Sollai S, Galli L, de Martino M.

Clinical usefulness of the semiquantitative procalcitonin test in the diagnosis

of bacterial infections in a third level children's hospital. Clin Lab.

2011;57(7-8):497-506. PubMed PMID: 21888013.

511: Torfoss D, Sandstad B, Mollnes TE, Høiby EA, Holte H, Bjerner J, Bjøro T,

Gaudernack G, Kvalheim G, Kvaløy S. The mild inflammatory response in febrile

neutropenic lymphoma patients with low risk of complications is more pronounced

in patients receiving tobramycin once daily compared with three times daily.

Scand J Immunol. 2011 Dec;74(6):632-9. doi: 10.1111/j.1365-3083.2011.02618.x.

PubMed PMID: 21883353.

512: Luyt CE, Combes A, Trouillet JL, Chastre J. Biomarkers to optimize

antibiotic therapy for pneumonia due to multidrug-resistant pathogens. Clin Chest

Med. 2011 Sep;32(3):431-8. doi: 10.1016/j.ccm.2011.05.004. Review. PubMed PMID:

21867813.

513: Gac AC, Parienti JJ, Chantepie S, Fradin S, Le Coutour X, Leclercq R, Reman

O. Dynamics of procalcitonin and bacteremia in neutropenic adults with acute

myeloid leukemia. Leuk Res. 2011 Oct;35(10):1294-6. doi:

10.1016/j.leukres.2011.05.035. Epub 2011 Aug 9. PubMed PMID: 21831426.

514: Yilmaz G, Köksal I, Karahan SC, Mentese A. The diagnostic and prognostic

significance of soluble urokinase plasminogen activator receptor in systemic

inflammatory response syndrome. Clin Biochem. 2011 Oct;44(14-15):1227-30. doi:

10.1016/j.clinbiochem.2011.07.006. Epub 2011 Jul 26. PubMed PMID: 21816136.

515: Aabenhus R, Jensen JU. Procalcitonin-guided antibiotic treatment of

respiratory tract infections in a primary care setting: are we there yet? Prim

Care Respir J. 2011 Dec;20(4):360-7. doi: 10.4104/pcrj.2011.00064. Review. PubMed

PMID: 21808938.

516: Sinha M, Desai S, Mantri S, Kulkarni A. Procalcitonin as an adjunctive

biomarker in sepsis. Indian J Anaesth. 2011 May;55(3):266-70. doi:

10.4103/0019-5049.82676. PubMed PMID: 21808399; PubMed Central PMCID: PMC3141151.

517: Otto GP, Sossdorf M, Claus RA, Rödel J, Menge K, Reinhart K, Bauer M,

Riedemann NC. The late phase of sepsis is characterized by an increased

microbiological burden and death rate. Crit Care. 2011 Jul 28;15(4):R183. doi:

10.1186/cc10332. PubMed PMID: 21798063; PubMed Central PMCID: PMC3387626.

518: Estella A. Cytokine levels in bronchoalveolar lavage and serum in 3 patients

with 2009 Influenza A(H1N1)v severe pneumonia. J Infect Dev Ctries. 2011 Jul

27;5(7):540-3. PubMed PMID: 21795823.

519: Blanchais T, Legrand A, Allain Launay E, Leclair MD, Caillon J,

Roussey-Kesler G. [Comparison of two protocols of febrile urinary tract infection

management in children]. Arch Pediatr. 2011 Sep;18(9):955-61. doi:

10.1016/j.arcped.2011.06.005. Epub 2011 Jul 26. French. PubMed PMID: 21795028.

520: Koo CY, Eisenhut M. Towards evidence-based emergency medicine: best BETs

from the Manchester Royal Infirmary. Can inflammatory markers distinguish

streptococcal from viral tonsillitis? Emerg Med J. 2011 Aug;28(8):715-7. doi:

10.1136/emj.2010.099531. Review. PubMed PMID: 21788241.

521: Gilbert DN. Procalcitonin and pulmonary aspiration: another possible

interpretation. Crit Care Med. 2011 Aug;39(8):2019-20; author reply 2020-1. doi:

10.1097/CCM.0b013e31821e8570. Erratum in: Crit Care Med. 2011 Oct;39(10):2392.

PubMed PMID: 21768821.

522: Chalupa P, Beran O, Herwald H, Kaspříková N, Holub M. Evaluation of

potential biomarkers for the discrimination of bacterial and viral infections.

Infection. 2011 Oct;39(5):411-7. doi: 10.1007/s15010-011-0126-4. Epub 2011 Jul 1.

PubMed PMID: 21720792.

523: Fica C A, Cifuentes D M, Hervé E B. [Update of the consensus document on

ventilator-associated pneumonia: part I. Diagnostic aspects]. Rev Chilena

Infectol. 2011 Apr;28(2):130-51. doi: /S0716-10182011000200005. Epub 2011 May 5.

Spanish. PubMed PMID: 21720692.

524: Tokman S, Schuetz P, Bent S. Procalcitonin-guided antibiotic therapy for

chronic obstructive pulmonary disease exacerbations. Expert Rev Anti Infect Ther.

2011 Jun;9(6):727-35. doi: 10.1586/eri.11.45. Review. PubMed PMID: 21692680.

525: Su CP, Chen TH, Chen SY, Ghiang WC, Wu GH, Sun HY, Lee CC, Wang JL, Chang

SC, Chen YC, Yen AM, Chen WJ, Hsueh PR. Predictive model for bacteremia in adult

patients with blood cultures performed at the emergency department: a preliminary

report. J Microbiol Immunol Infect. 2011 Dec;44(6):449-55. doi:

10.1016/j.jmii.2011.04.006. Epub 2011 Jun 17. PubMed PMID: 21684227.

526: Song JY, Cheong HJ, Heo JY, Noh JY, Yong HS, Kim YK, Kang EY, Choi WS, Jo

YM, Kim WJ. Clinical, laboratory and radiologic characteristics of 2009 pandemic

influenza A/H1N1 pneumonia: primary influenza pneumonia versus

concomitant/secondary bacterial pneumonia. Influenza Other Respir Viruses. 2011

Nov;5(6):e535-43. doi: 10.1111/j.1750-2659.2011.00269.x. Epub 2011 Jun 20. PubMed

PMID: 21682848; PubMed Central PMCID: PMC5780671.

527: Chazan R. [Contemporary clinical diagnostics of respiratory tract

infections]. Pol Merkur Lekarski. 2011 May;30(179):316-9. Review. Polish. PubMed

PMID: 21675131.

528: Ivády B, Béres BJ, Szabó D. Recent advances in sepsis research: novel

biomarkers and therapeutic targets. Curr Med Chem. 2011;18(21):3211-25. Review.

PubMed PMID: 21671855.

529: Jeddi R, Ghédira H, Ben Amor R, Turki A, Kacem K, Ben Abdennebi Y, Ben

Lakhal R, Aissaoui L, Ben Abid H, Bel Hadjali Z, Meddeb B. Risk factors of septic

shock in patients with hematologic malignancies and Pseudomonas infections.

Hematology. 2011 May;16(3):160-5. doi: 10.1179/102453311X12953015767293. PubMed

PMID: 21669056.

530: Ahn S, Kim WY, Kim SH, Hong S, Lim CM, Koh Y, Lim KS, Kim W. Role of

procalcitonin and C-reactive protein in differentiation of mixed bacterial

infection from 2009 H1N1 viral pneumonia. Influenza Other Respir Viruses. 2011

Nov;5(6):398-403. doi: 10.1111/j.1750-2659.2011.00244.x. Epub 2011 Mar 30. PubMed

PMID: 21668682; PubMed Central PMCID: PMC5780656.

531: Hochreiter M, Schroeder S. [Procalcitonin-based algorithm. Management of

antibiotic therapy in critically ill patients]. Anaesthesist. 2011

Jul;60(7):661-73. doi: 10.1007/s00101-011-1884-1. Review. German. PubMed PMID:

21660525.

532: Drago L, Vassena C, Dozio E, Corsi MM, De Vecchi E, Mattina R, Romanò C.

Procalcitonin, C-reactive protein, interleukin-6, and soluble intercellular

adhesion molecule-1 as markers of postoperative orthopaedic joint prosthesis

infections. Int J Immunopathol Pharmacol. 2011 Apr-Jun;24(2):433-40. PubMed PMID:

21658317.

533: Saeed K, Dryden M, Bourne S, Paget C, Proud A. Reduction in antibiotic use

through procalcitonin testing in patients in the medical admission unit or

intensive care unit with suspicion of infection. J Hosp Infect. 2011

Aug;78(4):289-92. doi: 10.1016/j.jhin.2011.03.018. Epub 2011 Jun 2. PubMed PMID:

21636167.

534: Průcha M, Matejovic M, Sedlácková L. [Diagnostics of sepsis]. Cas Lek Cesk.

2011;150(4-5):283-8. Review. Czech. PubMed PMID: 21634209.

535: Guinard-Barbier S, Grabar S, Chenevier-Gobeaux C, Quinquis L, Schmidt J,

Kierzek G, Guérin S, Hausfater P, Bernot B, Brun P, Gayet A, Casalino E,

Andreotti C, Renaud B, Claessens YE. Is mid-regional pro-atrial natriuretic

peptide (MRproANP) an accurate marker of bacteremia in pyelonephritis?

Biomarkers. 2011 Jun;16(4):355-63. doi: 10.3109/1354750X.2011.576769. PubMed

PMID: 21595569.

536: Robinson JO, Lamoth F, Bally F, Knaup M, Calandra T, Marchetti O. Monitoring

procalcitonin in febrile neutropenia: what is its utility for initial diagnosis

of infection and reassessment in persistent fever? PLoS One. 2011 Apr

25;6(4):e18886. doi: 10.1371/journal.pone.0018886. PubMed PMID: 21541027; PubMed

Central PMCID: PMC3081821.

537: Kordek A, Torbé A, Podraza W, Łoniewska B, Jursa-Kulesza J, Rudnicki J. Does

prenatal antibiotic therapy compromise the diagnosis of early-onset infection and

management of the neonate? J Perinat Med. 2011 May;39(3):337-42. doi:

10.1515/JPM.2011.031. Epub 2011 May 3. PubMed PMID: 21534881.

538: Popowski T, Goffinet F, Batteux F, Maillard F, Kayem G. [Prediction of

maternofetal infection in preterm premature rupture of membranes: serum maternal

markers]. Gynecol Obstet Fertil. 2011 May;39(5):302-8. doi:

10.1016/j.gyobfe.2010.11.006. Epub 2011 Apr 22. Review. French. PubMed PMID:

21515086.

539: Luyt CE, Combes A, Trouillet JL, Chastre J. Value of the serum procalcitonin

level to guide antimicrobial therapy for patients with ventilator-associated

pneumonia. Semin Respir Crit Care Med. 2011 Apr;32(2):181-7. doi:

10.1055/s-0031-1275530. Epub 2011 Apr 19. Review. PubMed PMID: 21506054.

540: Hammond NE, Corley A, Fraser JF. The utility of procalcitonin in diagnosis

of H1N1 influenza in intensive care patients. Anaesth Intensive Care. 2011

Mar;39(2):238-41. PubMed PMID: 21485672.

541: Lacoma A, Prat C, Andreo F, Lores L, Ruiz-Manzano J, Ausina V, Domínguez J.

Value of procalcitonin, C-reactive protein, and neopterin in exacerbations of

chronic obstructive pulmonary disease. Int J Chron Obstruct Pulmon Dis.

2011;6:157-69. doi: 10.2147/COPD.S16070. Epub 2011 Feb 28. PubMed PMID: 21468168;

PubMed Central PMCID: PMC3064422.

542: Ibrahim KA, Abdel-Wahab AA, Ibrahim AS. Diagnostic value of serum

procalcitonin levels in children with meningitis: a comparison with blood

leukocyte count and C-reactive protein. J Pak Med Assoc. 2011 Apr;61(4):346-51.

PubMed PMID: 21465970.

543: Vernet G, Saha S, Satzke C, Burgess DH, Alderson M, Maisonneuve JF, Beall

BW, Steinhoff MC, Klugman KP. Laboratory-based diagnosis of pneumococcal

pneumonia: state of the art and unmet needs. Clin Microbiol Infect. 2011 May;17

Suppl 3:1-13. doi: 10.1111/j.1469-0691.2011.03496.x. PubMed PMID: 21457174.

544: Khubutiia MSh, Abakumov MM, Aleksandrova IV, Reĭ SI, Il'inskiĭ ME, Khvatov

VB, Borovkova NV, Pertsev AS, Zinkin VIu. [Selective adsorption of endotoxin in

the complex treatment of patients with severe sepsis]. Anesteziol Reanimatol.

2010 Sep-Oct;(5):65-9. Russian. PubMed PMID: 21395145.

545: Séguéla PE, Joram N, Romefort B, Manteau C, Orsonneau JL, Branger B, Gournay

V, Rozé JC, Gras-Le Guen C. Procalcitonin as a marker of bacterial infection in

children undergoing cardiac surgery with cardiopulmonary bypass. Cardiol Young.

2011 Aug;21(4):392-9. doi: 10.1017/S104795111100014X. Epub 2011 Mar 9. PubMed

PMID: 21385512.

546: Cuquemelle E, Soulis F, Villers D, Roche-Campo F, Ara Somohano C, Fartoukh

M, Kouatchet A, Mourvillier B, Dellamonica J, Picard W, Schmidt M, Boulain T,

Brun-Buisson C; A/H1N1 REVA-SRLF Study Group. Can procalcitonin help identify

associated bacterial infection in patients with severe influenza pneumonia? A

multicentre study. Intensive Care Med. 2011 May;37(5):796-800. doi:

10.1007/s00134-011-2189-1. Epub 2011 Mar 3. PubMed PMID: 21369807.

547: Le UN, Kim HS, Kwon JS, Kim MY, Nguyen VH, Jiang SN, Lee BI, Hong Y, Shin

MG, Rhee JH, Bom HS, Ahn Y, Gambhir SS, Choy HE, Min JJ. Engineering and

visualization of bacteria for targeting infarcted myocardium. Mol Ther. 2011

May;19(5):951-9. doi: 10.1038/mt.2011.25. Epub 2011 Mar 1. PubMed PMID: 21364539;

PubMed Central PMCID: PMC3098638.

548: Siloşi C, Ghelase F, Siloşi I, Ghelase SM, Rogoz S, Cioară F, Râmboiu S,

Bratiloveanu T, Patrascu S, Neaţă G. [The evaluation of immunoinflammatory

response in acute bacterial peritonitis]. Chirurgia (Bucur). 2010

Nov-Dec;105(6):789-96. Romanian. PubMed PMID: 21355176.

549: Jereb M, Lunaček NK, Kotar T, Saksida A, Petrovec M, Avšič-Županc T.

Procalcitonin in hantavirus infections. Scand J Clin Lab Invest. 2011

Jul;71(4):287-91. doi: 10.3109/00365513.2011.560675. Epub 2011 Feb 23. PubMed

PMID: 21344982.

550: Kim MH, Lim G, Kang SY, Lee WI, Suh JT, Lee HJ. Utility of procalcitonin as

an early diagnostic marker of bacteremia in patients with acute fever. Yonsei Med

J. 2011 Mar;52(2):276-81. doi: 10.3349/ymj.2011.52.2.276. PubMed PMID: 21319346;

PubMed Central PMCID: PMC3051230.

551: Koivula I, Hämäläinen S, Jantunen E, Pulkki K, Kuittinen T, Nousiainen T,

Juutilainen A. Elevated procalcitonin predicts Gram-negative sepsis in

haematological patients with febrile neutropenia. Scand J Infect Dis. 2011

Jul;43(6-7):471-8. doi: 10.3109/00365548.2011.554855. Epub 2011 Feb 7. PubMed

PMID: 21299364.

552: Williamson DR, Albert M, Perreault MM, Delisle MS, Muscedere J, Rotstein C,

Jiang X, Heyland DK. The relationship between Candida species cultured from the

respiratory tract and systemic inflammation in critically ill patients with

ventilator-associated pneumonia. Can J Anaesth. 2011 Mar;58(3):275-84. doi:

10.1007/s12630-010-9439-5. Epub 2010 Dec 14. PubMed PMID: 21287306.

553: El-Solh AA, Vora H, Knight PR 3rd, Porhomayon J. Diagnostic use of serum

procalcitonin levels in pulmonary aspiration syndromes. Crit Care Med. 2011

Jun;39(6):1251-6. doi: 10.1097/CCM.0b013e31820a942c. PubMed PMID: 21283001;

PubMed Central PMCID: PMC3102149.

554: Manzano S, Bailey B, Gervaix A, Cousineau J, Delvin E, Girodias JB. Markers

for bacterial infection in children with fever without source. Arch Dis Child.

2011 May;96(5):440-6. doi: 10.1136/adc.2010.203760. Epub 2011 Jan 29. PubMed

PMID: 21278424.

555: Kordek A. Concentrations of procalcitonin and C-reactive protein, white

blood cell count, and the immature-to-total neutrophil ratio in the blood of

neonates with nosocomial infections: Gram-negative bacilli vs coagulase-negative

staphylococci. Eur J Clin Microbiol Infect Dis. 2011 Mar;30(3):455-7. doi:

10.1007/s10096-010-0956-5. Epub 2011 Jan 28. PubMed PMID: 21274587; PubMed

Central PMCID: PMC3034916.

556: Kern WV. [Bacteraemia and sepsis]. Dtsch Med Wochenschr. 2011

Feb;136(5):182-5. doi: 10.1055/s-0031-1272505. Epub 2011 Jan 26. Review. German.

PubMed PMID: 21271477.

557: Turan H, Horasanli B, Ugur M, Arslan H. Procalcitonin levels in migraine

patients. Can J Neurol Sci. 2011 Jan;38(1):124-8. PubMed PMID: 21156441.

558: Reinhart K, Hartog CS. Biomarkers as a guide for antimicrobial therapy. Int

J Antimicrob Agents. 2010 Dec;36 Suppl 2:S17-21. doi:

10.1016/j.ijantimicag.2010.11.009. Epub 2010 Dec 3. Review. PubMed PMID:

21129930.

559: van Nieuwkoop C, Bonten TN, van't Wout JW, Kuijper EJ, Groeneveld GH, Becker

MJ, Koster T, Wattel-Louis GH, Delfos NM, Ablij HC, Leyten EM, van Dissel JT.

Procalcitonin reflects bacteremia and bacterial load in urosepsis syndrome: a

prospective observational study. Crit Care. 2010;14(6):R206. doi: 10.1186/cc9328.

Epub 2010 Nov 17. PubMed PMID: 21083886; PubMed Central PMCID: PMC3220019.

560: Díez-Padrisa N, Bassat Q, Machevo S, Quintó L, Morais L, Nhampossa T,

O'Callaghan-Gordo C, Torres A, Alonso PL, Roca A. Procalcitonin and C-reactive

protein for invasive bacterial pneumonia diagnosis among children in Mozambique,

a malaria-endemic area. PLoS One. 2010 Oct 14;5(10):e13226. doi:

10.1371/journal.pone.0013226. PubMed PMID: 20976241; PubMed Central PMCID:

PMC2954814.

561: Knudsen JB, Fuursted K, Petersen E, Wierup P, Mølgaard H, Poulsen SH,

Egeblad H. Procalcitonin in 759 patients clinically suspected of infective

endocarditis. Am J Med. 2010 Dec;123(12):1121-7. doi:

10.1016/j.amjmed.2010.07.018. Epub 2010 Oct 1. PubMed PMID: 20870199.

562: te Witt R, van Wolfswinkel ME, Petit PL, van Hellemond JJ, Koelewijn R, van

Belkum A, van Genderen PJ. Neopterin and procalcitonin are suitable biomarkers

for exclusion of severe Plasmodium falciparum disease at the initial clinical

assessment of travellers with imported malaria. Malar J. 2010 Sep 14;9:255. doi:

10.1186/1475-2875-9-255. PubMed PMID: 20840738; PubMed Central PMCID: PMC2946356.

563: Chen SC, Kontoyiannis DP. New molecular and surrogate biomarker-based tests

in the diagnosis of bacterial and fungal infection in febrile neutropenic

patients. Curr Opin Infect Dis. 2010 Dec;23(6):567-77. doi:

10.1097/QCO.0b013e32833ef7d1. Review. PubMed PMID: 20827189.

564: Guervilly C, Coisel Y, Botelho-Nevers E, Dizier S, Castanier M,

Lepaul-Ercole R, Brissy O, Roch A, Forel JM, Papazian L. Significance of high

levels of procalcitonin in patients with influenza A (H1N1) pneumonia. J Infect.

2010 Oct;61(4):355-8. doi: 10.1016/j.jinf.2010.07.013. Epub 2010 Jul 27. PubMed

PMID: 20670651.

565: Worthington T, Dunlop D, Casey A, Lambert R, Luscombe J, Elliott T. Serum

procalcitonin, interleukin-6, soluble intercellular adhesin molecule-1 and IgG to

short-chain exocellular lipoteichoic acid as predictors of infection in total

joint prosthesis revision. Br J Biomed Sci. 2010;67(2):71-6. PubMed PMID:

20669762.

566: Limper M, Smit PM, Bongers KM, van Zanten AP, Smits PH, Brandjes DP, Mulder

JW, von Rosenstiel IA, van Gorp EC. Procalcitonin in children with suspected

novel influenza A (H1N1) infection. J Infect. 2010 Oct;61(4):351-3. doi:

10.1016/j.jinf.2010.07.005. Epub 2010 Jul 16. PubMed PMID: 20638412.

567: Wunderink RG. Surrogate markers and microbiologic end points. Clin Infect

Dis. 2010 Aug 1;51 Suppl 1:S126-30. doi: 10.1086/653061. Review. PubMed PMID:

20597662.

568: Kato M, Kaneko S, Takagaki K, Kohayagawa Y, Watanabe T, Yoshida Y, Yamamoto

O. Procalcitonin as a biomarker for toxic shock syndrome. Acta Derm Venereol.

2010 Jul;90(4):441-3. doi: 10.2340/00015555-0873. PubMed PMID: 20574626.

569: Kopyra P, Seremak-Mrozikiewicz A, Drews K. [Usefulness of PCT, IL-6, CRP

measurement in the prediction of intraamniotic infection and newborn status in

pregnant women with premature rupture of membranes]. Ginekol Pol. 2010

May;81(5):336-41. Polish. PubMed PMID: 20568512.

570: Galetto-Lacour A, Zamora SA, Andreola B, Bressan S, Lacroix L, Da Dalt L,

Gervaix A. Validation of a laboratory risk index score for the identification of

severe bacterial infection in children with fever without source. Arch Dis Child.

2010 Dec;95(12):968-73. doi: 10.1136/adc.2009.176800. Epub 2010 Jun 1. PubMed

PMID: 20515973.

571: Ternhag A, Cars O, Norman C, Struwe J. [Antibiotics or not--procalcitonin

can guide therapeutic choices. At least at the emergency department for adults

with lower respiratory tract infection]. Lakartidningen. 2010 Apr

14-20;107(15):985-8. Review. Swedish. PubMed PMID: 20464915.

572: Nascimento-Carvalho CM, Cardoso MR, Barral A, Araújo-Neto CA, Guerin S,

Saukkoriipi A, Paldanius M, Vainionpää R, Lebon P, Leinonen M, Ruuskanen O,

Gendrel D. Procalcitonin is useful in identifying bacteraemia among children with

pneumonia. Scand J Infect Dis. 2010 Sep;42(9):644-9. doi:

10.3109/00365541003796775. PubMed PMID: 20438288.

573: Kherad O, Kaiser L, Bridevaux PO, Sarasin F, Thomas Y, Janssens JP,

Rutschmann OT. Upper-respiratory viral infection, biomarkers, and COPD

exacerbations. Chest. 2010 Oct;138(4):896-904. doi: 10.1378/chest.09-2225. Epub

2010 Apr 30. PubMed PMID: 20435659.

574: Gilbert DN. Use of plasma procalcitonin levels as an adjunct to clinical

microbiology. J Clin Microbiol. 2010 Jul;48(7):2325-9. doi: 10.1128/JCM.00655-10.

Epub 2010 Apr 26. Review. PubMed PMID: 20421436; PubMed Central PMCID:

PMC2897488.

575: Limper M, de Kruif MD, Duits AJ, Brandjes DP, van Gorp EC. The diagnostic

role of procalcitonin and other biomarkers in discriminating infectious from

non-infectious fever. J Infect. 2010 Jun;60(6):409-16. doi:

10.1016/j.jinf.2010.03.016. Epub 2010 Mar 27. Review. PubMed PMID: 20347867.

576: Müller F, Christ-Crain M, Bregenzer T, Krause M, Zimmerli W, Mueller B,

Schuetz P; ProHOSP Study Group. Procalcitonin levels predict bacteremia in

patients with community-acquired pneumonia: a prospective cohort trial. Chest.

2010 Jul;138(1):121-9. doi: 10.1378/chest.09-2920. Epub 2010 Mar 18. PubMed PMID:

20299634.

577: Martini A, Gottin L, Menestrina N, Schweiger V, Simion D, Vincent JL.

Procalcitonin levels in surgical patients at risk of candidemia. J Infect. 2010

Jun;60(6):425-30. doi: 10.1016/j.jinf.2010.03.003. Epub 2010 Mar 10. PubMed PMID:

20226210.

578: Porcel JM. Pleural fluid tests to identify complicated parapneumonic

effusions. Curr Opin Pulm Med. 2010 Jul;16(4):357-61. doi:

10.1097/MCP.0b013e328338a108. Review. PubMed PMID: 20224408.

579: Jung B, Embriaco N, Roux F, Forel JM, Demory D, Allardet-Servent J, Jaber S,

La Scola B, Papazian L. Microbiogical data, but not procalcitonin improve the

accuracy of the clinical pulmonary infection score. Intensive Care Med. 2010

May;36(5):790-8. doi: 10.1007/s00134-010-1833-5. Epub 2010 Mar 9. PubMed PMID:

20217042.

580: Baruti Gafurri Z, Pacarizi H, Zhubi B, Begolli L, Topciu V. The importance

of determining procalcitonin and C reactive protein in different stages of

sepsis. Bosn J Basic Med Sci. 2010 Feb;10(1):60-4. PubMed PMID: 20192933; PubMed

Central PMCID: PMC5596613.

581: Gendrel D, Vallet C, Gelmetti C, Moulin F, Brasme JF, Chalumeau M, Cohen R,

Raymond J. [Pneumococcal pneumonia highly probable in immunized children cared

for in-group settings]. Arch Pediatr. 2010 Apr;17(4):373-7. doi:

10.1016/j.arcped.2009.11.031. Epub 2010 Feb 26. French. PubMed PMID: 20189361.

582: Sakorafas GH, Lappas C, Mastoraki A, Delis SG, Safioleas M. Current trends

in the management of infected necrotizing pancreatitis. Infect Disord Drug

Targets. 2010 Feb;10(1):9-14. Review. PubMed PMID: 20180753.

583: Balci C, Sivaci R, Akbulut G, Karabekir HS. Procalcitonin levels as an early

marker in patients with multiple trauma under intensive care. J Int Med Res. 2009

Nov-Dec;37(6):1709-17. PubMed PMID: 20146868.

584: Tasdelen Fisgin N, Aliyazicioglu Y, Tanyel E, Coban AY, Ulger F, Zivalioglu

M, Esen S, Leblebicioglu H. The value of neopterin and procalcitonin in patients

with sepsis. South Med J. 2010 Mar;103(3):216-9. doi:

10.1097/SMJ.0b013e3181cf11a1. PubMed PMID: 20134373.

585: Roos KL, van de Beek D. Bacterial meningitis. Handb Clin Neurol.

2010;96:51-63. doi: 10.1016/S0072-9752(09)96004-3. Epub 2010 Jan 19. PubMed PMID:

20109674.

586: Ingram PR, Inglis T, Moxon D, Speers D. Procalcitonin and C-reactive protein

in severe 2009 H1N1 influenza infection. Intensive Care Med. 2010

Mar;36(3):528-32. doi: 10.1007/s00134-009-1746-3. PubMed PMID: 20069274.

587: Eisenhut M. Use of procalcitonin measurement to identify bacterial

co-infection in patients with H1N1 influenza. Acta Paediatr. 2010

Apr;99(4):487-8. doi: 10.1111/j.1651-2227.2009.01663.x. Epub 2010 Jan 8. PubMed

PMID: 20064131.

588: Schuetz P, Batschwaroff M, Dusemund F, Albrich W, Bürgi U, Maurer M,

Brutsche M, Huber AR, Müller B. Effectiveness of a procalcitonin algorithm to

guide antibiotic therapy in respiratory tract infections outside of study

conditions: a post-study survey. Eur J Clin Microbiol Infect Dis. 2010

Mar;29(3):269-77. doi: 10.1007/s10096-009-0851-0. Epub 2009 Dec 29. PubMed PMID:

20039090.

589: Zeglen S, Wojarski J, Wozniak-Grygiel E, Siola M, Szewczyk M, Kucewicz-Czech

E, Nozynski J, Zembala M. Procalcitonin serum concentration during Pneumocystis

jiroveci colonization or Pseudomonas aeruginosa infection/colonization in lung

transplant recipients. Transplant Proc. 2009 Oct;41(8):3225-7. doi:

10.1016/j.transproceed.2009.08.007. PubMed PMID: 19857716.

590: Mantadakis E, Plessa E, Vouloumanou EK, Karageorgopoulos DE, Chatzimichael

A, Falagas ME. Serum procalcitonin for prediction of renal parenchymal

involvement in children with urinary tract infections: a meta-analysis of

prospective clinical studies. J Pediatr. 2009 Dec;155(6):875-881.e1. doi:

10.1016/j.jpeds.2009.06.037. Epub 2009 Oct 21. PubMed PMID: 19850301.

591: Suberviola Cañas B, González Castro A, Holanda Peña MS, Fernández Miret B.

[Usefulness of procalcitonin in the diagnosis of infection in lung transplant

patients]. Med Intensiva. 2009 Oct;33(7):358-9. doi: 10.1016/j.medin.2008.10.001.

Epub 2009 Sep 13. Spanish. PubMed PMID: 19828399.

592: Hatzistilianou M, Rekliti A, Athanassiadou F, Catriu D. Procalcitonin as an

early marker of bacterial infection in neutropenic febrile children with acute

lymphoblastic leukemia. Inflamm Res. 2010 May;59(5):339-47. doi:

10.1007/s00011-009-0100-0. Epub 2009 Oct 6. PubMed PMID: 19806318.

593: Stolz D, Smyrnios N, Eggimann P, Pargger H, Thakkar N, Siegemund M, Marsch

S, Azzola A, Rakic J, Mueller B, Tamm M. Procalcitonin for reduced antibiotic

exposure in ventilator-associated pneumonia: a randomised study. Eur Respir J.

2009 Dec;34(6):1364-75. doi: 10.1183/09031936.00053209. Epub 2009 Sep 24. PubMed

PMID: 19797133.

594: Daubin C, Parienti JJ, Fradin S, Vabret A, Ramakers M, Terzi N, Freymuth F,

Charbonneau P, du Cheyron D. Procalcitonin levels and bacterial aetiology among

COPD patients admitted to the ICU with severe pneumonia: a prospective cohort

study. BMC Infect Dis. 2009 Sep 21;9:157. doi: 10.1186/1471-2334-9-157. PubMed

PMID: 19772586; PubMed Central PMCID: PMC2754485.

595: Charles PE, Castro C, Ruiz-Santana S, León C, Saavedra P, Martín E. Serum

procalcitonin levels in critically ill patients colonized with Candida spp: new

clues for the early recognition of invasive candidiasis? Intensive Care Med. 2009

Dec;35(12):2146-50. doi: 10.1007/s00134-009-1623-0. Epub 2009 Sep 16. PubMed

PMID: 19760210.

596: Groselj-Grenc M, Ihan A, Pavcnik-Arnol M, Kopitar AN, Gmeiner-Stopar T,

Derganc M. Neutrophil and monocyte CD64 indexes, lipopolysaccharide-binding

protein, procalcitonin and C-reactive protein in sepsis of critically ill

neonates and children. Intensive Care Med. 2009 Nov;35(11):1950-8. doi:

10.1007/s00134-009-1637-7. PubMed PMID: 19756501.

597: Martinez-Albarran M, Perez-Molina Jde J, Gallegos-Castorena S,

Sanchez-Zubieta F, Del Toro-Arreola S, Troyo-Sanroman R, Gonzalez-Ramella O.

Procalcitonin and C-reactive protein serum levels as markers of infection in a

pediatric population with febrile neutropenia and cancer. Pediatr Hematol Oncol.

2009 Sep;26(6):414-25. doi: 10.3109/08880010903044797. PubMed PMID: 19657991.

598: Kim OY, Monsel A, Bertrand M, Cavaillon JM, Coriat P, Adib-Conquy M.

Translocation of bacterial NOD2 agonist and its link with inflammation. Crit

Care. 2009;13(4):R124. doi: 10.1186/cc7980. Epub 2009 Jul 28. PubMed PMID:

19638210; PubMed Central PMCID: PMC2750177.

599: Zhen ZJ, Xia Y, Ling JY, Tong GL, Lin L, Cai Y, Sun XF. [Prophylaxis and

treatment of modified BFM-90 regimen for lymphoblastic lymphoma in children and

adolescents accompanied with infection]. Ai Zheng. 2009 Jul;28(7):718-24.

Chinese. PubMed PMID: 19624898.

600: Krüger S, Ewig S, Papassotiriou J, Kunde J, Marre R, von Baum H, Suttor N,

Welte T; CAPNETZ Study Group. Inflammatory parameters predict etiologic patterns

but do not allow for individual prediction of etiology in patients with CAP:

results from the German competence network CAPNETZ. Respir Res. 2009 Jul

12;10:65. doi: 10.1186/1465-9921-10-65. PubMed PMID: 19594893; PubMed Central

PMCID: PMC2714042.

601: von Lilienfeld-Toal M, Lehmann LE, Raadts AD, Hahn-Ast C, Orlopp KS,

Marklein G, Purr I, Cook G, Hoeft A, Glasmacher A, Stüber F. Utility of a

commercially available multiplex real-time PCR assay to detect bacterial and

fungal pathogens in febrile neutropenia. J Clin Microbiol. 2009

Aug;47(8):2405-10. doi: 10.1128/JCM.00491-09. Epub 2009 Jul 1. PubMed PMID:

19571034; PubMed Central PMCID: PMC2725651.

602: Jesús Cremades M, Luiza de Souza-Galvão M, García JM, Menéndez R.

[Respiratory infections research: a perspective from the tuberculosis and

respiratory infections area (TIR)]. Arch Bronconeumol. 2009;45 Suppl 1:11-5. doi:

10.1016/S0300-2896(09)70265-0. Review. Spanish. PubMed PMID: 19303524.

603: Chen DY, Chen YM, Ho WL, Chen HH, Shen GH, Lan JL. Diagnostic value of

procalcitonin for differentiation between bacterial infection and non-infectious

inflammation in febrile patients with active adult-onset Still's disease. Ann

Rheum Dis. 2009 Jun;68(6):1074-5. doi: 10.1136/ard.2008.098335. PubMed PMID:

19435724.

604: Carbonnelle E. [Laboratory diagnosis of bacterial meningitis: usefulness of

various tests for the determination of the etiological agent]. Med Mal Infect.

2009 Jul-Aug;39(7-8):581-605. doi: 10.1016/j.medmal.2009.02.017. Epub 2009 Apr

23. Review. French. PubMed PMID: 19398286.

605: Meng FS, Su L, Tang YQ, Wen Q, Liu YS, Liu ZF. Serum procalcitonin at the

time of admission to the ICU as a predictor of short-term mortality. Clin

Biochem. 2009 Jul;42(10-11):1025-31. doi: 10.1016/j.clinbiochem.2009.03.012. Epub

2009 Mar 24. PubMed PMID: 19324026.

606: Damoiseaux RA. [Determination of procalcitonin. Valuable for reducing

ineffective antibiotic use is debatable]. Ned Tijdschr Geneeskd. 2009 Feb

14;153(7):268-9. Dutch. PubMed PMID: 19291938.

607: Kiliç D, Gunaydin S, Kisa U, Sari T, Deveci O, Zorlutuna Y. Clinical

efficacy of leukofiltration on cardiopulmonary bypass related inflammatory

response: Fact or Foe? Inflamm Res. 2009 Jun;58(6):292-7. doi:

10.1007/s00011-008-7244-1. PubMed PMID: 19266265.

608: Bender L, Thaarup J, Varming K, Krarup H, Ellermann-Eriksen S, Ebbesen F.

Early and late markers for the detection of early-onset neonatal sepsis. Dan Med

Bull. 2008 Nov;55(4):219-23. PubMed PMID: 19232162.

609: Polverino E, Torres A. Diagnostic strategies for healthcare-associated

pneumonia. Semin Respir Crit Care Med. 2009 Feb;30(1):36-45. doi:

10.1055/s-0028-1119807. Epub 2009 Feb 6. Review. PubMed PMID: 19199185.

610: Kaiga T, Ogiwara T, Murakami M. [New methods for diagnosis of sepsis].

Rinsho Byori. 2008 Nov;56(11):1043-9. Review. Japanese. PubMed PMID: 19086461.

611: Apiwattanakul N, Wanitkun S, Chongtrakool P, Sirinavin S. A patient with

penicillin-resistant viridans group streptococcal endocarditis and unusual

reactions to vancomycin. Southeast Asian J Trop Med Public Health. 2008

Nov;39(6):1088-91. PubMed PMID: 19062700.

612: Sandri MT, Passerini R, Leon ME, Peccatori FA, Zorzino L, Salvatici M,

Riggio D, Cassatella C, Cinieri S, Martinelli G. Procalcitonin as a useful marker

of infection in hemato-oncological patients with fever. Anticancer Res. 2008

Sep-Oct;28(5B):3061-5. PubMed PMID: 19031957.

613: Klugman KP, Madhi SA, Albrich WC. Novel approaches to the identification of

Streptococcus pneumoniae as the cause of community-acquired pneumonia. Clin

Infect Dis. 2008 Dec 1;47 Suppl 3:S202-6. doi: 10.1086/591405. Review. PubMed

PMID: 18986290.

614: Niederman MS. Biological markers to determine eligibility in trials for

community-acquired pneumonia: a focus on procalcitonin. Clin Infect Dis. 2008 Dec

1;47 Suppl 3:S127-32. doi: 10.1086/591393. Review. PubMed PMID: 18986278.

615: Rudensky B, Sirota G, Erlichman M, Yinnon AM, Schlesinger Y. Neutrophil CD64

expression as a diagnostic marker of bacterial infection in febrile children

presenting to a hospital emergency department. Pediatr Emerg Care. 2008

Nov;24(11):745-8. doi: 10.1097/PEC.0b013e31818c2679. PubMed PMID: 18955911.

616: Daubin C, Parienti JJ, Vabret A, Ramakers M, Fradin S, Terzi N, Freymuth F,

Charbonneau P, du Cheyron D. Procalcitonin levels in acute exacerbation of COPD

admitted in ICU: a prospective cohort study. BMC Infect Dis. 2008 Oct 23;8:145.

doi: 10.1186/1471-2334-8-145. PubMed PMID: 18947382; PubMed Central PMCID:

PMC2577677.

617: Kotoula A, Gardikis S, Tsalkidis A, Mantadakis E, Zissimopoulos A, Kambouri

K, Deftereos S, Tripsianis G, Manolas K, Chatzimichael A, Vaos G. Procalcitonin

for the early prediction of renal parenchymal involvement in children with UTI:

preliminary results. Int Urol Nephrol. 2009;41(2):393-9. doi:

10.1007/s11255-008-9472-2. Epub 2008 Oct 3. PubMed PMID: 18836845.

618: Beloborodova NV, Vostrikova TIu, Chernevskaia EA. [Etiology of postoperative

bacteremias in an intensive care unit: an association with the level of

procalcitonin]. Anesteziol Reanimatol. 2008 Jul-Aug;(4):22-7. Russian. PubMed

PMID: 18819390.

619: Ebell M. Procalcitonin-guided treatment of respiratory tract infections. Am

Fam Physician. 2008 Sep 15;78(6):756-7. Review. PubMed PMID: 18819243.

620: Strassburg A, Rupp J, Herth FJ, Magnussen H, Zabel P, Lange C. [Infection

diagnostics in pneumology. Part 1. Survey and methods]. Pneumologie. 2008

Dec;62(12):730-43. doi: 10.1055/s-2008-1038235. Epub 2008 Sep 5. Review. German.

PubMed PMID: 18777468.

621: Bulut C, Oztürk R, Yilmaz GR, Parpucu H, Irmak H, Kinikli S, Duranay M,

Demiröz AP. [Evaluation of the epidemiological, clinical and laboratory findings

in continuous ambulatory peritoneal dialysis related peritonitis attacks].

Mikrobiyol Bul. 2008 Apr;42(2):255-64. Turkish. PubMed PMID: 18697423.

622: Haeuptle J, Zaborsky R, Fiumefreddo R, Trampuz A, Steffen I, Frei R,

Christ-Crain M, Müller B, Schuetz P. Prognostic value of procalcitonin in

Legionella pneumonia. Eur J Clin Microbiol Infect Dis. 2009 Jan;28(1):55-60. doi:

10.1007/s10096-008-0592-5. Epub 2008 Aug 2. PubMed PMID: 18677519.

623: Jensen JU, Lundgren B, Hein L, Mohr T, Petersen PL, Andersen LH, Lauritsen

AO, Hougaard S, Mantoni T, Bømler B, Thornberg KJ, Thormar K, Løken J, Steensen

M, Carl P, Petersen JA, Tousi H, Søe-Jensen P, Bestle M, Hestad S, Andersen MH,

Fjeldborg P, Larsen KM, Rossau C, Thomsen CB, Ostergaard C, Kjaer J, Grarup J,

Lundgren JD. The Procalcitonin And Survival Study (PASS) - a randomised

multi-center investigator-initiated trial to investigate whether daily

measurements biomarker Procalcitonin and pro-active diagnostic and therapeutic

responses to abnormal Procalcitonin levels, can improve survival in intensive

care unit patients. Calculated sample size (target population): 1000 patients.

BMC Infect Dis. 2008 Jul 13;8:91. doi: 10.1186/1471-2334-8-91. PubMed PMID:

18620598; PubMed Central PMCID: PMC2491622.

624: Hügle T, Schuetz P, Mueller B, Laifer G, Tyndall A, Regenass S, Daikeler T.

Serum procalcitonin for discrimination between septic and non-septic arthritis.

Clin Exp Rheumatol. 2008 May-Jun;26(3):453-6. PubMed PMID: 18578968.

625: Nseir S, Cavestri B, Di Pompeo C, Diarra M, Brisson H, Lemyze M,

Roussel-Delvallez M, Durocher A. Factors predicting bacterial involvement in

severe acute exacerbations of chronic obstructive pulmonary disease. Respiration.

2008;76(3):253-60. doi: 10.1159/000139611. Epub 2008 Jun 12. PubMed PMID:

18547944.

626: Cuculi F, Toggweiler S, Auer M, der Maur ChA, Zuber M, Erne P. Serum

procalcitonin has the potential to identify Staphylococcus aureus endocarditis.

Eur J Clin Microbiol Infect Dis. 2008 Nov;27(11):1145-9. doi:

10.1007/s10096-008-0541-3. Epub 2008 Jun 3. PubMed PMID: 18521635.

627: Groeneveld AB, Hack CE. The role of the innate immune response in hospital-

versus community-acquired infection in febrile medical patients. Int J Infect

Dis. 2008 Nov;12(6):660-70. doi: 10.1016/j.ijid.2008.03.009. Epub 2008 Jun 2.

PubMed PMID: 18514561.

628: Chatzimavroudis G, Pavlidis TE, Koutelidakis I, Giamarrelos-Bourboulis EJ,

Atmatzidis S, Kontopoulou K, Marakis G, Atmatzidis K. CO(2) pneumoperitoneum

prolongs survival in an animal model of peritonitis compared to laparotomy. J

Surg Res. 2009 Mar;152(1):69-75. doi: 10.1016/j.jss.2008.02.030. Epub 2008 Mar

18. PubMed PMID: 18499131.

629: Kulabukhov VV. Use of an endotoxin adsorber in the treatment of severe

abdominal sepsis. Acta Anaesthesiol Scand. 2008 Aug;52(7):1024-5. doi:

10.1111/j.1399-6576.2008.01677.x. Epub 2008 May 20. PubMed PMID: 18494849.

630: Linssen CF, Bekers O, Drent M, Jacobs JA. C-reactive protein and

procalcitonin concentrations in bronchoalveolar lavage fluid as a predictor of

ventilator-associated pneumonia. Ann Clin Biochem. 2008 May;45(Pt 3):293-8. doi:

10.1258/acb.2007.007133. PubMed PMID: 18482918.

631: Rau B, Steinbach G, Baumgart K, Gansauge F, Grünert A, Beger HG. The

clinical value of procalcitonin in the prediction of infected necrosis in acute

pancreatitis. Intensive Care Med. 2000 Mar;26 Suppl 2:S159-64. doi:

10.1007/BF02900730. PubMed PMID: 18470712.

632: Rea-Neto A, Youssef NC, Tuche F, Brunkhorst F, Ranieri VM, Reinhart K, Sakr

Y. Diagnosis of ventilator-associated pneumonia: a systematic review of the

literature. Crit Care. 2008;12(2):R56. doi: 10.1186/cc6877. Epub 2008 Apr 21.

Review. PubMed PMID: 18426596; PubMed Central PMCID: PMC2447611.

633: Thia KT, Chan ES, Ling KL, Ng WY, Jacob E, Ooi CJ. Role of procalcitonin in

infectious gastroenteritis and inflammatory bowel disease. Dig Dis Sci. 2008

Nov;53(11):2960-8. doi: 10.1007/s10620-008-0254-6. Epub 2008 Apr 16. PubMed PMID:

18415679.

634: Pelosi P, Barassi A, Severgnini P, Gomiero B, Finazzi S, Merlini G, d'Eril

GM, Chiaranda M, Niederman MS. Prognostic role of clinical and laboratory

criteria to identify early ventilator-associated pneumonia in brain injury.

Chest. 2008 Jul;134(1):101-8. doi: 10.1378/chest.07-2546. Epub 2008 Apr 10.

PubMed PMID: 18403669.

635: Tasabehji WR, Al-Quobaili FA, Al-Daher NA. Usefulness of procalcitonin and

some inflammatory parameters in septic patients. Saudi Med J. 2008

Apr;29(4):520-5. PubMed PMID: 18382791.

636: Carboni GL, Fahrner R, Gazdhar A, Printzen G, Schmid RA, Hoksch B.

Comparison of procalcitonin and CrP in the postoperative course after lung

decortication. Eur J Cardiothorac Surg. 2008 May;33(5):777-80. doi:

10.1016/j.ejcts.2008.02.013. PubMed PMID: 18374593.

637: Charles PE, Ladoire S, Aho S, Quenot JP, Doise JM, Prin S, Olsson NO,

Blettery B. Serum procalcitonin elevation in critically ill patients at the onset

of bacteremia caused by either Gram negative or Gram positive bacteria. BMC

Infect Dis. 2008 Mar 26;8:38. doi: 10.1186/1471-2334-8-38. PubMed PMID: 18366777;

PubMed Central PMCID: PMC2289831.

638: Kalocheretis P, Revela I, Spanou E, Drouzas A, Makriniotou I, Iatrou C.

Strong correlation of B2-microglobulin (B2-m) with procalcitonin (PCT) in the

serum of chronic hemodialysis patients: a role for infections in the

dialysis-related amyloidosis? Ren Fail. 2008;30(3):261-5. doi:

10.1080/08860220701857134. PubMed PMID: 18350445.

639: Lisboa T, Rello J. Diagnosis of ventilator-associated pneumonia: is there a

gold standard and a simple approach? Curr Opin Infect Dis. 2008 Apr;21(2):174-8.

doi: 10.1097/QCO.0b013e3282f55dd1. Review. PubMed PMID: 18317042.

640: Póvoa P. Serum markers in community-acquired pneumonia and

ventilator-associated pneumonia. Curr Opin Infect Dis. 2008 Apr;21(2):157-62.

doi: 10.1097/QCO.0b013e3282f47c32. Review. PubMed PMID: 18317039.

641: Fottner A, Birkenmaier C, von Schulze Pellengahr C, Wegener B, Jansson V.

Can serum procalcitonin help to differentiate between septic and nonseptic

arthritis? Arthroscopy. 2008 Feb;24(2):229-33. doi: 10.1016/j.arthro.2007.07.029.

Epub 2007 Nov 5. PubMed PMID: 18237709.

642: Lam HS, Ng PC. Biochemical markers of neonatal sepsis. Pathology. 2008

Feb;40(2):141-8. doi: 10.1080/00313020701813735. Review. PubMed PMID: 18203036.

643: Ali AM, Moaz MA, Ghoniem E, Abd El Motaleb T, Sheri N. Reliability of serum

procalcitonin concentrations for the diagnosis of sepsis in neonates. Egypt J

Immunol. 2008;15(1):75-84. PubMed PMID: 20306671.

644: Piotrowski A, Stengert W, Stolarska M, Fendler W. [Acute respiratory failure

in a 16-yr-old girl with alveolar soft part sarcoma]. Anestezjol Intens Ter. 2008

Jan-Mar;40(1):28-31. Polish. PubMed PMID: 19469095.

645: Uzun G, Solmazgul E, Curuksulu H, Turhan V, Ardic N, Top C, Yildiz S, Cimsit

M. Procalcitonin as a diagnostic aid in diabetic foot infections. Tohoku J Exp

Med. 2007 Dec;213(4):305-12. PubMed PMID: 18075234.

646: Schmidt N, Palma J, King A, Santolaya ME. [C reactive protein and

procalcitonin levels for the diagnosis of invasive bacterial infections in

allogenic hematopoietic stem cell transplantation recipients]. Rev Med Chil. 2007

Aug;135(8):982-9. Epub 2007 Oct 25. Spanish. PubMed PMID: 17989854.

647: Fendler WM, Piotrowski AJ. Procalcitonin in the early diagnosis of

nosocomial sepsis in preterm neonates. J Paediatr Child Health. 2008

Mar;44(3):114-8. Epub 2007 Oct 10. PubMed PMID: 17927729.

648: Rosmarakis ES, Prapas SN, Rellos K, Michalopoulos A, Samonis G, Falagas ME.

Nosocomial infections after off-pump coronary artery bypass surgery: frequency,

characteristics, and risk factors. Interact Cardiovasc Thorac Surg. 2007

Dec;6(6):759-67. Epub 2007 Sep 28. PubMed PMID: 17905781.

649: Schuetz P, Mueller B, Trampuz A. Serum procalcitonin for discrimination of

blood contamination from bloodstream infection due to coagulase-negative

staphylococci. Infection. 2007 Oct;35(5):352-5. Epub 2007 Sep 19. PubMed PMID:

17882355.

650: Myers C, Gervaix A. Streptococcus pneumoniae bacteraemia in children. Int J

Antimicrob Agents. 2007 Nov;30 Suppl 1:S24-8. Epub 2007 Aug 20. Review. PubMed

PMID: 17707612.

651: Ip M, Rainer TH, Lee N, Chan C, Chau SS, Leung W, Leung MF, Tam TK, Antonio

GE, Lui G, Lau TK, Hui DS, Fuchs D, Renneberg R, Chan PK. Value of serum

procalcitonin, neopterin, and C-reactive protein in differentiating bacterial

from viral etiologies in patients presenting with lower respiratory tract

infections. Diagn Microbiol Infect Dis. 2007 Oct;59(2):131-6. Epub 2007 Jul 26.

PubMed PMID: 17662560.

652: Schuetz P, Christ-Crain M, Wolbers M, Schild U, Thomann R, Falconnier C,

Widmer I, Neidert S, Blum CA, Schönenberger R, Henzen C, Bregenzer T, Hoess C,

Krause M, Bucher HC, Zimmerli W, Müller B; ProHOSP study group. Procalcitonin

guided antibiotic therapy and hospitalization in patients with lower respiratory

tract infections: a prospective, multicenter, randomized controlled trial. BMC

Health Serv Res. 2007 Jul 5;7:102. PubMed PMID: 17615073; PubMed Central PMCID:

PMC1947969.

653: Watkin RW, Harper LV, Vernallis AB, Lang S, Lambert PA, Ranasinghe AM,

Elliott TS. Pro-inflammatory cytokines IL6, TNF-alpha, IL1beta, procalcitonin,

lipopolysaccharide binding protein and C-reactive protein in infective

endocarditis. J Infect. 2007 Sep;55(3):220-5. Epub 2007 Jun 22. PubMed PMID:

17586049.

654: Gibot S, Cravoisy A, Dupays R, Barraud D, Nace L, Levy B, Bollaert PE.

Combined measurement of procalcitonin and soluble TREM-1 in the diagnosis of

nosocomial sepsis. Scand J Infect Dis. 2007;39(6-7):604-8. PubMed PMID: 17577825.

655: Knudsen TB, Larsen K, Kristiansen TB, Møller HJ, Tvede M, Eugen-Olsen J,

Kronborg G. Diagnostic value of soluble CD163 serum levels in patients suspected

of meningitis: comparison with CRP and procalcitonin. Scand J Infect Dis.

2007;39(6-7):542-53. PubMed PMID: 17577816.

656: Morath C, Sis J, Haensch GM, Zeier M, Andrassy K, Schwenger V. Procalcitonin

as marker of infection in patients with Goodpasture's syndrome is misleading.

Nephrol Dial Transplant. 2007 Sep;22(9):2701-4. Epub 2007 Jun 7. PubMed PMID:

17556410.

657: Völl K, Haase G, Fritz H, Riebe J, Huszka C, Kindler J. [Septic shock with

purpura fulminans after a dog bite]. Dtsch Med Wochenschr. 2007 Jun

15;132(24):1321-4. German. PubMed PMID: 17551886.

658: Michelow IC, Katz K, McCracken GH, Hardy RD. Systemic cytokine profile in

children with community-acquired pneumonia. Pediatr Pulmonol. 2007

Jul;42(7):640-5. PubMed PMID: 17534977.

659: Bustos Betanzo RO. [Procalcitonin, C-reactive protein and leukocyte count in

very-low birth weight infants with late neonatal sepsis]. An Pediatr (Barc). 2007

May;66(5):541-2. Spanish. PubMed PMID: 17517212.

660: Lee BE, Davies HD. Aseptic meningitis. Curr Opin Infect Dis. 2007

Jun;20(3):272-7. Review. PubMed PMID: 17471037.

661: Rau BM, Kemppainen EA, Gumbs AA, Büchler MW, Wegscheider K, Bassi C,

Puolakkainen PA, Beger HG. Early assessment of pancreatic infections and overall

prognosis in severe acute pancreatitis by procalcitonin (PCT): a prospective

international multicenter study. Ann Surg. 2007 May;245(5):745-54. PubMed PMID:

17457167; PubMed Central PMCID: PMC1877072.

662: Flückiger U, Battegay M, Laifer G. [Diagnostic procedures for patients with

community acquired pneumonia]. Internist (Berl). 2007 May;48(5):468, 470-2,

474-5. Review. German. PubMed PMID: 17390118.

663: Herd D. In children under age three does procalcitonin help exclude serious

bacterial infection in fever without focus? Arch Dis Child. 2007 Apr;92(4):362-4.

Review. PubMed PMID: 17376946; PubMed Central PMCID: PMC2083685.

664: Don M, Valent F, Korppi M, Falleti E, De Candia A, Fasoli L, Tenore A,

Canciani M. Efficacy of serum procalcitonin in evaluating severity of

community-acquired pneumonia in childhood. Scand J Infect Dis. 2007;39(2):129-37.

PubMed PMID: 17366029.

665: Masiá M, Gutiérrez F, Padilla S, Soldán B, Mirete C, Shum C, Hernández I,

Royo G, Martin-Hidalgo A. Clinical characterisation of pneumonia caused by

atypical pathogens combining classic and novel predictors. Clin Microbiol Infect.

2007 Feb;13(2):153-161. doi: 10.1111/j.1469-0691.2006.01629.x. PubMed PMID:

17328727.

666: Ray P, Badarou-Acossi G, Viallon A, Boutoille D, Arthaud M, Trystram D, Riou

B. Accuracy of the cerebrospinal fluid results to differentiate bacterial from

non bacterial meningitis, in case of negative gram-stained smear. Am J Emerg Med.

2007 Feb;25(2):179-84. PubMed PMID: 17276808.

667: Bottner F, Wegner A, Winkelmann W, Becker K, Erren M, Götze C.

Interleukin-6, procalcitonin and TNF-alpha: markers of peri-prosthetic infection

following total joint replacement. J Bone Joint Surg Br. 2007 Jan;89(1):94-9.

PubMed PMID: 17259424.

668: Dubos F, Moulin F, Raymond J, Gendrel D, Bréart G, Chalumeau M. [Distinction

between bacterial and aseptic meningitis in children: refinement of a clinical

decision rule]. Arch Pediatr. 2007 May;14(5):434-8. Epub 2007 Jan 26. French.

PubMed PMID: 17258439.

669: Chawes BL, Rechnitzer C, Schmiegelow K, Tvede M. [Procalcitonin for early

diagnosis of bacteraemia in children with cancer]. Ugeskr Laeger. 2007 Jan

8;169(2):138-42. Danish. PubMed PMID: 17227662.

670: Turner D, Hammerman C, Rudensky B, Schlesinger Y, Schimmel MS. The role of

procalcitonin as a predictor of nosocomial sepsis in preterm infants. Acta

Paediatr. 2006 Dec;95(12):1571-6. PubMed PMID: 17129964.

671: Peters RP, Twisk JW, van Agtmael MA, Groeneveld AB. The role of

procalcitonin in a decision tree for prediction of bloodstream infection in

febrile patients. Clin Microbiol Infect. 2006 Dec;12(12):1207-13. PubMed PMID:

17121627.

672: Chang C, Yao WZ, Chen YH, Liu ZY, Zhang XW. [The changes and clinical

implications of serum procalcitonin in acute exacerbations of chronic obstructive

pulmonary disease]. Zhonghua Jie He He Hu Xi Za Zhi. 2006 Jul;29(7):444-7.

Chinese. PubMed PMID: 17045042.

673: Sponholz C, Sakr Y, Reinhart K, Brunkhorst F. Diagnostic value and

prognostic implications of serum procalcitonin after cardiac surgery: a

systematic review of the literature. Crit Care. 2006;10(5):R145. Review. PubMed

PMID: 17038199; PubMed Central PMCID: PMC1751067.

674: Makhoul IR, Yacoub A, Smolkin T, Sujov P, Kassis I, Sprecher H. Values of

C-reactive protein, procalcitonin, and Staphylococcus-specific PCR in neonatal

late-onset sepsis. Acta Paediatr. 2006 Oct;95(10):1218-23. PubMed PMID: 16982493.

675: Seligman R, Meisner M, Lisboa TC, Hertz FT, Filippin TB, Fachel JM, Teixeira

PJ. Decreases in procalcitonin and C-reactive protein are strong predictors of

survival in ventilator-associated pneumonia. Crit Care. 2006;10(5):R125. PubMed

PMID: 16956405; PubMed Central PMCID: PMC1751074.

676: Jensen JU, Heslet L, Jensen TH, Espersen K, Steffensen P, Tvede M.

Procalcitonin increase in early identification of critically ill patients at high

risk of mortality. Crit Care Med. 2006 Oct;34(10):2596-602. PubMed PMID:

16915118.

677: Heper Y, Akalin EH, Mistik R, Akgöz S, Töre O, Göral G, Oral B, Budak F,

Helvaci S. Evaluation of serum C-reactive protein, procalcitonin, tumor necrosis

factor alpha, and interleukin-10 levels as diagnostic and prognostic parameters

in patients with community-acquired sepsis, severe sepsis, and septic shock. Eur

J Clin Microbiol Infect Dis. 2006 Aug;25(8):481-91. PubMed PMID: 16896829.

678: Chang C, Yao WZ, Chen YH, Liu ZY, Zhang XW. [Value of serum procalcitonin in

diagnosing bacterial lower respiratory tract infections in people with

exacerbation of Chronic Obstructive Pulmonary Disease]. Beijing Da Xue Xue Bao Yi

Xue Ban. 2006 Aug 18;38(4):389-92. Chinese. PubMed PMID: 16892144.

679: Beloborodova NV, Nonikov VE, Bachinskaia EN. [Diagnostic and therapeutic

aspects of pneumonias after cardiosurgical operations associated with artificial

ventilation]. Anesteziol Reanimatol. 2006 May-Jun;(3):83-7. Russian. PubMed PMID:

16889222.

680: Popov DA, Beloborodova NV, Iarustovskiĭ MB. [Systemic inflammation after

operations on the open heart: significance of a microbial factor]. Anesteziol

Reanimatol. 2006 May-Jun;(3):79-83. Review. Russian. PubMed PMID: 16889221.

681: Slŭncheva B, Vakrilova L, Emilova Z, Iarŭkova N, Shishkova R, Popivanova A.

[Inborn bacterial infections during neonatal period]. Akush Ginekol (Sofiia).

2006;45(4):42-8. Bulgarian. PubMed PMID: 16889200.

682: Stolz D, Christ-Crain M, Gencay MM, Bingisser R, Huber PR, Müller B, Tamm M.

Diagnostic value of signs, symptoms and laboratory values in lower respiratory

tract infection. Swiss Med Wkly. 2006 Jul 8;136(27-28):434-40. PubMed PMID:

16862463.

683: Dahaba AA, Hagara B, Fall A, Rehak PH, List WF, Metzler H. Procalcitonin for

early prediction of survival outcome in postoperative critically ill patients

with severe sepsis. Br J Anaesth. 2006 Oct;97(4):503-8. Epub 2006 Jul 18. PubMed

PMID: 16849384.

684: Bignardi GE, Dhar R, Heycock R, Bansal S, Majmudar N. Can procalcitonin

testing reduce antibiotic prescribing for respiratory infections? Age Ageing.

2006 Nov;35(6):625-6. Epub 2006 Jul 5. PubMed PMID: 16822805.

685: Baylan O, Balkan A, Inal A, Kisa O, Albay A, Doganci L, Acikel CH. The

predictive value of serum procalcitonin levels in adult patients with active

pulmonary tuberculosis. Jpn J Infect Dis. 2006 Jun;59(3):164-7. PubMed PMID:

16785696.

686: Kitanovski L, Jazbec J, Hojker S, Gubina M, Derganc M. Diagnostic accuracy

of procalcitonin and interleukin-6 values for predicting bacteremia and clinical

sepsis in febrile neutropenic children with cancer. Eur J Clin Microbiol Infect

Dis. 2006 Jun;25(6):413-5. PubMed PMID: 16767494.

687: Nyamande K, Lalloo UG. Serum procalcitonin distinguishes CAP due to

bacteria, Mycobacterium tuberculosis and PJP. Int J Tuberc Lung Dis. 2006

May;10(5):510-5. PubMed PMID: 16704032.

688: Beloborodova NV, Arkhipova AS, Beloborodov DM, Boĭko NB, Mel'ko AI, Olenin

AIu. [Chromatographic mass spectrometric determination of low-molecular-weight

aromatic compounds of microbial origin in the serum from patients with sepsis].

Klin Lab Diagn. 2006 Feb;(2):3-6. Russian. PubMed PMID: 16610621.

689: Elefsiniotis IS, Skounakis M, Vezali E, Pantazis KD, Petrocheilou A,

Pirounaki M, Papatsibas G, Kontou-Kastellanou C, Moulakakis A. Clinical

significance of serum procalcitonin levels in patients with acute or chronic

liver disease. Eur J Gastroenterol Hepatol. 2006 May;18(5):525-30. PubMed PMID:

16607149.

690: Christ-Crain M, Stolz D, Bingisser R, Müller C, Miedinger D, Huber PR,

Zimmerli W, Harbarth S, Tamm M, Müller B. Procalcitonin guidance of antibiotic

therapy in community-acquired pneumonia: a randomized trial. Am J Respir Crit

Care Med. 2006 Jul 1;174(1):84-93. Epub 2006 Apr 7. PubMed PMID: 16603606.

691: Hellerstein S. Acute urinary tract infection--evaluation and treatment. Curr

Opin Pediatr. 2006 Apr;18(2):134-8. Review. PubMed PMID: 16601492.

692: Ertugrul BM, Yilmabasar A, Ertugrul O, Ayabakan HB, Kizilirmak S, Turkmen S.

Do C-reactive protein and procalcitonin predict hospital-acquired infection in

patients with trauma? Saudi Med J. 2006 Apr;27(4):560-2. PubMed PMID: 16598344.

693: Bogar L, Molnar Z, Kenyeres P, Tarsoly P. Sedimentation characteristics of

leucocytes can predict bacteraemia in critical care patients. J Clin Pathol. 2006

May;59(5):523-5. Epub 2006 Mar 13. PubMed PMID: 16533954; PubMed Central PMCID:

PMC1860278.

694: Walder G, Fuchs D, Sarcletti M, Berek K, Falkensammer B, Huber K, Petrovec

M, Dierich MP, Würzner R. Human granulocytic anaplasmosis in Austria:

epidemiological, clinical, and laboratory findings in five consecutive patients

from Tyrol, Austria. Int J Med Microbiol. 2006 May;296 Suppl 40:297-301. Epub

2006 Mar 10. PubMed PMID: 16531117.

695: Elsammak M, Hanna H, Ghazal A, Edeen FB, Kandil M. Diagnostic value of serum

procalcitonin and C-reactive protein in Egyptian children with streptococcal

tonsillopharyngitis. Pediatr Infect Dis J. 2006 Feb;25(2):174-6. PubMed PMID:

16462299.

696: Pincíková T, Bucová M, Slobodníková L. [Influrence of recombinant human

procalcitonin on phagocytic and candidacidal ability of polymorphonuclear

leukocytes and on killing mechanisms of serum and blood aganist bacteria

Staphylococcus aureus and Escherichia coli]. Vnitr Lek. 2005 Dec;51(12):1365-70.

Slovak. PubMed PMID: 16430103.

697: Butbul-Aviel Y, Koren A, Halevy R, Sakran W. Procalcitonin as a diagnostic

aid in osteomyelitis and septic arthritis. Pediatr Emerg Care. 2005

Dec;21(12):828-32. PubMed PMID: 16340758.

698: Chevalier I, Gauthier M. Procalcitonin and vesicoureteral reflux in children

with urinary tract infection. Pediatrics. 2005 Nov;116(5):1261-2; author reply

1262-3. PubMed PMID: 16264022.

699: de Mendonça-Filho HT, Gomes GS, Nogueira PM, Fernandes MA, Tura BR, Santos

M, Castro-Faria-Neto HC. Macrophage migration inhibitory factor is associated

with positive cultures in patients with sepsis after cardiac surgery. Shock. 2005

Oct;24(4):313-7. PubMed PMID: 16205314.

700: Tavares E, Maldonado R, Ojeda ML, Miñano FJ. Circulating inflammatory

mediators during start of fever in differential diagnosis of gram-negative and

gram-positive infections in leukopenic rats. Clin Diagn Lab Immunol. 2005

Sep;12(9):1085-93. PubMed PMID: 16148175; PubMed Central PMCID: PMC1235789.

701: Gürgöze MK, Akarsu S, Yilmaz E, Gödekmerdan A, Akça Z, Ciftçi I, Aygün AD.

Proinflammatory cytokines and procalcitonin in children with acute

pyelonephritis. Pediatr Nephrol. 2005 Oct;20(10):1445-8. Epub 2005 Aug 4. PubMed

PMID: 16079986.

702: Jeena P. The role of HIV infection in acute respiratory infections among

children in sub-Saharan Africa. Int J Tuberc Lung Dis. 2005 Jul;9(7):708-15.

Review. PubMed PMID: 16013764.

703: Thayyil S, Shenoy M, Hamaluba M, Gupta A, Frater J, Verber IG. Is

procalcitonin useful in early diagnosis of serious bacterial infections in

children? Acta Paediatr. 2005 Feb;94(2):155-8. PubMed PMID: 15981747.

704: Gibot S, Cravoisy A. Soluble form of the triggering receptor expressed on

myeloid cells-1 as a marker of microbial infection. Clin Med Res. 2004

Aug;2(3):181-7. Review. PubMed PMID: 15931355; PubMed Central PMCID: PMC1069091.

705: Kafetzis DA, Tigani GS, Costalos C. Immunologic markers in the neonatal

period: diagnostic value and accuracy in infection. Expert Rev Mol Diagn. 2005

Mar;5(2):231-9. Review. PubMed PMID: 15833052.

706: Davis BH. Improved diagnostic approaches to infection/sepsis detection.

Expert Rev Mol Diagn. 2005 Mar;5(2):193-207. Review. PubMed PMID: 15833049.

707: Wojtacha A, Juszczyk J, Czarniak E, Samet A. [Spontaneous bacterial

peritonitis in patients with decompensated liver cirrhosis based on

bacteriological and biochemical results]. Przegl Epidemiol. 2004;58(4):597-607.

Polish. PubMed PMID: 15810501.

708: Oláh A, Belágyi T, Issekutz A, Makay R, Zaborszky A. Value of procalcitonin

quick test in the differentiation between sterile and infected forms of acute

pancreatitis. Hepatogastroenterology. 2005 Jan-Feb;52(61):243-5. PubMed PMID:

15783040.

709: Dornbusch HJ, Strenger V, Kerbl R, Lackner H, Schwinger W, Sovinz P, Urban

C. Procalcitonin--a marker of invasive fungal infection? Support Care Cancer.

2005 May;13(5):343-6. Epub 2005 Jan 19. PubMed PMID: 15657690.

710: Schrøder H, Lodahl D. [Procalcitonin as a marker of severe bacterial

infection in children]. Ugeskr Laeger. 2004 Oct 18;166(43):3804-7. Review.

Danish. PubMed PMID: 15544109.

711: Tvede M. [Procalcitonin--acute inflammatory marker in infections]. Ugeskr

Laeger. 2004 Oct 18;166(43):3795. Danish. PubMed PMID: 15544106.

712: Ballot DE, Perovic O, Galpin J, Cooper PA. Serum procalcitonin as an early

marker of neonatal sepsis. S Afr Med J. 2004 Oct;94(10):851-4. PubMed PMID:

15532763.

713: Kawczyński P, Piotrowski A. [Procalcitonin and C-reactive protein as a

markers of neonatal sepsis]. Ginekol Pol. 2004 Jun;75(6):439-44. Polish. PubMed

PMID: 15524419.

714: van Rossum AM, Wulkan RW, Oudesluys-Murphy AM. Procalcitonin as an early

marker of infection in neonates and children. Lancet Infect Dis. 2004

Oct;4(10):620-30. Review. PubMed PMID: 15451490.

715: Luyt CE, Guérin V, Combes A, Trouillet JL, Ayed SB, Bernard M, Gibert C,

Chastre J. Procalcitonin kinetics as a prognostic marker of ventilator-associated

pneumonia. Am J Respir Crit Care Med. 2005 Jan 1;171(1):48-53. Epub 2004 Sep 24.

PubMed PMID: 15447947.

716: Muñoz P, Simarro N, Rivera M, Alonso R, Alcalá L, Bouza E. Evaluation of

procalcitonin as a marker of infection in a nonselected sample of febrile

hospitalized patients. Diagn Microbiol Infect Dis. 2004 Aug;49(4):237-41. PubMed

PMID: 15313527.

717: Aalto H, Takala A, Kautiainen H, Repo H. Laboratory markers of systemic

inflammation as predictors of bloodstream infection in acutely ill patients

admitted to hospital in medical emergency. Eur J Clin Microbiol Infect Dis. 2004

Sep;23(9):699-704. Epub 2004 Aug 10. PubMed PMID: 15309668.

718: Ortega M, Rovira M, Filella X, Almela M, Puig de la Bellacasa J, Carreras E,

Mensa J. Prospective evaluation of procalcitonin in adults with febrile

neutropenia after haematopoietic stem cell transplantation. Br J Haematol. 2004

Aug;126(3):372-6. PubMed PMID: 15257709.

719: Yonetci N, Sungurtekin U, Oruc N, Yilmaz M, Sungurtekin H, Kaleli I,

Kaptanoglu B, Yuce G, Ozutemiz O. Is procalcitonin a reliable marker for the

diagnosis of infected pancreatic necrosis? ANZ J Surg. 2004 Jul;74(7):591-5.

PubMed PMID: 15230799.

720: Giamarellou H, Giamarellos-Bourboulis EJ, Repoussis P, Galani L,

Anagnostopoulos N, Grecka P, Lubos D, Aoun M, Athanassiou K, Bouza E, Devigili E,

Krçmery V, Menichetti F, Panaretou E, Papageorgiou E, Plachouras D. Potential use

of procalcitonin as a diagnostic criterion in febrile neutropenia: experience

from a multicentre study. Clin Microbiol Infect. 2004 Jul;10(7):628-33. PubMed

PMID: 15214875.

721: Saribas S, Kocazeybek B, Aslan M, Altun S, Seyhun Y, Oner YA, Memisoglu N.

Do procalcitonin and C-reactive protein levels have a place in the diagnosis and

follow-up of Helicobacter pylori infections? J Med Microbiol. 2004 Jul;53(Pt

7):639-44. PubMed PMID: 15184535.

722: Braun JJ, de Graaff CS, de Goey J, Zwinderman AH, Petit PL.

[Community-acquired pneumonia: pathogens and course in patients admitted to a

general hospital]. Ned Tijdschr Geneeskd. 2004 Apr 24;148(17):836-40. Dutch.

PubMed PMID: 15141651.

723: Husová L, Husa P, Senkyrík M, Lata J. [Procalcitonin as an indicator of

infection in patients with liver cirrhosis]. Vnitr Lek. 2004 Feb;50(2):153-6.

Review. Czech. PubMed PMID: 15077592.

724: Mueller C, Huber P, Laifer G, Mueller B, Perruchoud AP. Procalcitonin and

the early diagnosis of infective endocarditis. Circulation. 2004 Apr

13;109(14):1707-10. Epub 2004 Apr 5. PubMed PMID: 15066945.

725: Chua AP, Lee KH. Procalcitonin in severe acute respiratory syndrome (SARS).

J Infect. 2004 May;48(4):303-6. PubMed PMID: 15066330.

726: Michelow IC, Olsen K, Lozano J, Rollins NK, Duffy LB, Ziegler T, Kauppila J,

Leinonen M, McCracken GH Jr. Epidemiology and clinical characteristics of

community-acquired pneumonia in hospitalized children. Pediatrics. 2004

Apr;113(4):701-7. PubMed PMID: 15060215.

727: Distefano G, Curreri R, Betta P, Romeo MG, Amato M. Procalcitonin serum

levels in perinatal bacterial and fungal infection of preterm infants. Acta

Paediatr. 2004 Feb;93(2):216-9. PubMed PMID: 15046277.

728: Korczowski B, Szybist W. Serum procalcitonin and C-reactive protein in

children with diarrhoea of various aetiologies. Acta Paediatr. 2004

Feb;93(2):169-73. PubMed PMID: 15046268.

729: Christ-Crain M, Jaccard-Stolz D, Bingisser R, Gencay MM, Huber PR, Tamm M,

Müller B. Effect of procalcitonin-guided treatment on antibiotic use and outcome

in lower respiratory tract infections: cluster-randomised, single-blinded

intervention trial. Lancet. 2004 Feb 21;363(9409):600-7. PubMed PMID: 14987884.

730: Prat C, Domínguez J, Rodrigo C, Giménez M, Azuara M, Blanco S, Ausina V. Use

of quantitative and semiquantitative procalcitonin measurements to identify

children with sepsis and meningitis. Eur J Clin Microbiol Infect Dis. 2004

Feb;23(2):136-8. Epub 2003 Dec 19. PubMed PMID: 14689316.

731: Prat C, Domínguez J, Rodrigo C, Giménez M, Azuara M, Jiménez O, Galí N,

Ausina V. Procalcitonin, C-reactive protein and leukocyte count in children with

lower respiratory tract infection. Pediatr Infect Dis J. 2003 Nov;22(11):963-8.

PubMed PMID: 14614368.

732: Fernández Lopez A, Luaces Cubells C, García García JJ, Fernández Pou J;

Spanish Society of Pediatric Emergencies. Procalcitonin in pediatric emergency

departments for the early diagnosis of invasive bacterial infections in febrile

infants: results of a multicenter study and utility of a rapid qualitative test

for this marker. Pediatr Infect Dis J. 2003 Oct;22(10):895-903. PubMed PMID:

14551491.

733: Scott LK, Grier LR, Arnold TC, Conrad SA. Serum procalcitonin concentration

as a negative predictor of serious bacterial infection in acute sickle cell pain

crisis. Med Sci Monit. 2003 Oct;9(10):CR426-31. PubMed PMID: 14523331.

734: Kandemir O, Uluba B, Polat G, Sezer C, Camdeviren H, Kaya A. Elevation of

procalcitonin level in patients with pulmonary tuberculosis and in medical staff

with close patient contact. Arch Med Res. 2003 Jul-Aug;34(4):311-4. PubMed PMID:

12957529.

735: Wrodycki W. [Usefulness of plasma procalcitonin (PCT) estimation to diagnose

patients in departments of infectious diseases]. Przegl Epidemiol.

2003;57(1):211-9. Polish. PubMed PMID: 12926330.

736: Holzheimer RG. Oral antibiotic prophylaxis can influence the inflammatory

response in aortic aneurysm repair: results of a randomized clinical study. J

Chemother. 2003 Apr;15(2):157-64. PubMed PMID: 12797394.

737: Resch B, Gusenleitner W, Müller W. Procalcitonin, interleukin-6, C-reactive

protein and leukocyte counts in infants with bronchiolitis. Pediatr Infect Dis J.

2003 May;22(5):475-6. PubMed PMID: 12792393.

738: Makay R, Issekutz A, Banga P, Belágyi T, Oláh A. [Role of procalcitonin

rapid test in the differential diagnosis of uninfected and infected forms of

acute pancreatitis]. Magy Seb. 2003 Feb;56(1):31-3. Hungarian. PubMed PMID:

12764990.

739: Kocazeybek B, Küçükoğlu S, Oner YA. Procalcitonin and C-reactive protein in

infective endocarditis: correlation with etiology and prognosis. Chemotherapy.

2003 May;49(1-2):76-84. PubMed PMID: 12714816.

740: Monneret G, Arpin M, Venet F, Maghni K, Debard AL, Pachot A, Lepape A,

Bienvenu J. Calcitonin gene related peptide and N-procalcitonin modulate CD11b

upregulation in lipopolysaccharide activated monocytes and neutrophils. Intensive

Care Med. 2003 Jun;29(6):923-928. doi: 10.1007/s00134-003-1759-2. Epub 2003 Apr

24. PubMed PMID: 12712241.

741: Mariscalco MM. Is plasma procalcitonin ready for prime time in the pediatric

intensive care unit? Pediatr Crit Care Med. 2003 Jan;4(1):118-9. PubMed PMID:

12656558.

742: Han YY, Doughty LA, Kofos D, Sasser H, Carcillo JA. Procalcitonin is

persistently increased among children with poor outcome from bacterial sepsis.

Pediatr Crit Care Med. 2003 Jan;4(1):21-5. PubMed PMID: 12656537.

743: Delèvaux I, André M, Colombier M, Albuisson E, Meylheuc F, Bègue RJ, Piette

JC, Aumaître O. Can procalcitonin measurement help in differentiating between

bacterial infection and other kinds of inflammatory processes? Ann Rheum Dis.

2003 Apr;62(4):337-40. PubMed PMID: 12634233; PubMed Central PMCID: PMC1754509.

744: Hryniewiecki T, Sitkiewicz D, Rawczyńska-Englert I. [Role of procalcitonin

in the diagnosis of uncomplicated infective endocarditis]. Przegl Lek.

2002;59(10):793-5. Polish. PubMed PMID: 12632914.

745: Debard AL, Vautrin C, Pariset C, Bienvenu J, Monneret G. High serum

procalcitonin levels do not predict bacteremia in adult patients with acute

fever. Clin Infect Dis. 2003 Mar 15;36(6):825-6; author reply 826-7. PubMed PMID:

12627373.

746: van Dissel JT. Procalcitonin: what should be its role in the clinical

management of febrile patients admitted to the hospital? Clin Infect Dis. 2003

Mar 15;36(6):824-5; author reply 826-7. PubMed PMID: 12627372.

747: Nylen E, Muller B, Becker KL, Snider R. The future diagnostic role of

procalcitonin levels: the need for improved sensitivity. Clin Infect Dis. 2003

Mar 15;36(6):823-4; author reply 826-7. PubMed PMID: 12627371.

748: Villanueva JL, Cervin RJ. Serum procalcitonin levels and empirical

antibiotic treatment of patients with community-acquired febrile syndromes. Clin

Infect Dis. 2003 Mar 15;36(6):822; author reply 826-7. PubMed PMID: 12627370.

749: Tsokos M. Fatal Waterhouse-Friderichsen syndrome due to Ewingella americana

infection. Am J Forensic Med Pathol. 2003 Mar;24(1):41-4. PubMed PMID: 12604997.

750: Van der Kaay DC, De Kleijn ED, De Rijke YB, Hop WC, De Groot R, Hazelzet JA.

Procalcitonin as a prognostic marker in meningococcal disease. Intensive Care

Med. 2002 Nov;28(11):1606-12. Epub 2002 Oct 4. PubMed PMID: 12415448.

751: Balog A, Ocsovszki I, Mándi Y. Flow cytometric analysis of procalcitonin

expression in human monocytes and granulocytes. Immunol Lett. 2002 Dec

3;84(3):199-203. PubMed PMID: 12413737.

752: Blommendahl J, Janas M, Laine S, Miettinen A, Ashorn P. Comparison of

procalcitonin with CRP and differential white blood cell count for diagnosis of

culture-proven neonatal sepsis. Scand J Infect Dis. 2002;34(8):620-2. PubMed

PMID: 12238581.

753: Berger C, Schwarz S, Schaebitz WR, Aschoff A, Schwab S. Serum procalcitonin

in cerebral ventriculitis. Crit Care Med. 2002 Aug;30(8):1778-81. PubMed PMID:

12163792.

754: Czyzewska M, Lachowska M, Gajewska E. [Evaluation of diagnostic value of

procalcitonin (PCT) as a marker of congenital infection in newborns]. Przegl Lek.

2002;59 Suppl 1:46-9. Polish. PubMed PMID: 12108072.

755: Chirouze C, Schuhmacher H, Rabaud C, Gil H, Khayat N, Estavoyer JM, May T,

Hoen B. Low serum procalcitonin level accurately predicts the absence of

bacteremia in adult patients with acute fever. Clin Infect Dis. 2002 Jul

15;35(2):156-61. Epub 2002 Jun 17. PubMed PMID: 12087521.

756: Dahaba AA, Elawady GA, Rehak PH, List WF. Procalcitonin and proinflammatory

cytokine clearance during continuous venovenous haemofiltration in septic

patients. Anaesth Intensive Care. 2002 Jun;30(3):269-74. PubMed PMID: 12075632.

757: Andermahr J, Greb A, Hensler T, Helling HJ, Bouillon B, Sauerland S, Rehm

KE, Neugebauer E. Pneumonia in multiple injured patients: a prospective

controlled trial on early prediction using clinical and immunological parameters.

Inflamm Res. 2002 May;51(5):265-72. PubMed PMID: 12056515.

758: Carrol ED, Newland P, Riordan FA, Thomson AP, Curtis N, Hart CA.

Procalcitonin as a diagnostic marker of meningococcal disease in children

presenting with fever and a rash. Arch Dis Child. 2002 Apr;86(4):282-5. PubMed

PMID: 11919107; PubMed Central PMCID: PMC1719162.

759: Shimetani N, Shimetani K, Mori M. Levels of three inflammation markers,

C-reactive protein, serum amyloid A protein and procalcitonin, in the serum and

cerebrospinal fluid of patients with meningitis. Scand J Clin Lab Invest.

2001;61(7):567-74. PubMed PMID: 11763415.

760: Rintala EM, Aittoniemi J, Laine S, Nevalainen TJ, Nikoskelainen J. Early

identification of bacteremia by biochemical markers of systemic inflammation.

Scand J Clin Lab Invest. 2001;61(7):523-30. PubMed PMID: 11763410.

761: Svaldi M, Hirber J, Lanthaler AI, Mayr O, Faes S, Peer E, Mitterer M.

Procalcitonin-reduced sensitivity and specificity in heavily leucopenic and

immunosuppressed patients. Br J Haematol. 2001 Oct;115(1):53-7. PubMed PMID:

11722409.

762: Liaudat S, Dayer E, Praz G, Bille J, Troillet N. Usefulness of procalcitonin

serum level for the diagnosis of bacteremia. Eur J Clin Microbiol Infect Dis.

2001 Aug;20(8):524-7. PubMed PMID: 11681430.

763: Eloy O, Vauloup C, Thérond P, Pina P, Allouch P, Pangon B, Bedos JP,

Ghnassia JC. [Procalcitonin level in deep-seated Candida infections]. Ann Biol

Clin (Paris). 2001 Jul-Aug;59(4):502-5. French. PubMed PMID: 11470651.

764: von Landenberg P, Shoenfeld Y. New approaches in the diagnosis of sepsis.

Isr Med Assoc J. 2001 Jun;3(6):439-42. Review. PubMed PMID: 11433639.

765: Mándi Y, Farkas G, Takács T, Boda K, Lonovics J. Diagnostic relevance of

procalcitonin, IL-6, and sICAM-1 in the prediction of infected necrosis in acute

pancreatitis. Int J Pancreatol. 2000 Aug;28(1):41-9. PubMed PMID: 11185709.

766: de Bont ES, Vellenga E, Swaanenburg J, Kamps W. Procalcitonin: a diagnostic

marker of bacterial infection in neutropenic cancer patients with fever?

Infection. 2000 Nov-Dec;28(6):398-400. PubMed PMID: 11139163.

767: Slavakis A, Papadimas J. Procalcitonin: does it play a role in male

reproduction? Fertil Steril. 2000 Dec;74(6):1227-8. PubMed PMID: 11119755.

768: van Langevelde P, Joop K, van Loon J, Frölich M, Groeneveld PH, Westendorp

RG, van Dissel JT. Endotoxin, cytokines, and procalcitonin in febrile patients

admitted to the hospital: identification of subjects at high risk of mortality.

Clin Infect Dis. 2000 Dec;31(6):1343-8. Epub 2000 Nov 29. PubMed PMID: 11096000.

769: Blijlevens NM, Donnelly JP, Meis JF, De Keizer MH, De Pauw BE. Procalcitonin

does not discriminate infection from inflammation after allogeneic bone marrow

transplantation. Clin Diagn Lab Immunol. 2000 Nov;7(6):889-92. PubMed PMID:

11063493; PubMed Central PMCID: PMC95980.

770: Viallon A, Zeni F, Pouzet V, Lambert C, Quenet S, Aubert G, Guyomarch S,

Tardy B, Bertrand JC. Serum and ascitic procalcitonin levels in cirrhotic

patients with spontaneous bacterial peritonitis: diagnostic value and

relationship to pro-inflammatory cytokines. Intensive Care Med. 2000

Aug;26(8):1082-8. PubMed PMID: 11030164.

771: Hedlund J, Hansson LO. Procalcitonin and C-reactive protein levels in

community-acquired pneumonia: correlation with etiology and prognosis. Infection.

2000 Mar-Apr;28(2):68-73. PubMed PMID: 10782390.

772: Petros S, Leonhardt U, Engelmann L. Serum procalcitonin and proinflammatory

cytokines in a patient with acute severe leptospirosis. Scand J Infect Dis.

2000;32(1):104-5. PubMed PMID: 10716092.

773: Lorrot M, Moulin F, Coste J, Ravilly S, Guérin S, Lebon P, Lacombe C,

Raymond J, Bohuon C, Gendrel D. [Procalcitonin in pediatric emergencies:

comparison with C-reactive protein, interleukin-6 and interferon alpha in the

differentiation between bacterial and viral infections]. Presse Med. 2000 Jan

29;29(3):128-34. French. PubMed PMID: 10686961.

774: Müller CA, Uhl W, Printzen G, Gloor B, Bischofberger H, Tcholakov O, Büchler

MW. Role of procalcitonin and granulocyte colony stimulating factor in the early

prediction of infected necrosis in severe acute pancreatitis. Gut. 2000

Feb;46(2):233-8. PubMed PMID: 10644318; PubMed Central PMCID: PMC1727805.

775: Brunkhorst FM, Clark AL, Forycki ZF, Anker SD. Pyrexia, procalcitonin,

immune activation and survival in cardiogenic shock: the potential importance of

bacterial translocation. Int J Cardiol. 1999 Dec 15;72(1):3-10. PubMed PMID:

10636626.

776: Bonač B, Derganc M, Wraber B, Hojker S. lnterleukin-8 and procalcitonin in

early diagnosis of early severe bacterial infection in critically ill neonates.

Pflugers Arch. 2000 Jan;440(Suppl 1):R072-R074. doi: 10.1007/s004240000011.

PubMed PMID: 28008487.

777: Brunkhorst FM, Eberhard OK, Brunkhorst R. Discrimination of infectious and

noninfectious causes of early acute respiratory distress syndrome by

procalcitonin. Crit Care Med. 1999 Oct;27(10):2172-6. PubMed PMID: 10548201.

778: Hatherill M, Tibby SM, Sykes K, Turner C, Murdoch IA. Diagnostic markers of

infection: comparison of procalcitonin with C reactive protein and leucocyte

count. Arch Dis Child. 1999 Nov;81(5):417-21. PubMed PMID: 10519716; PubMed

Central PMCID: PMC1718133.

779: Franz AR, Kron M, Pohlandt F, Steinbach G. Comparison of procalcitonin with

interleukin 8, C-reactive protein and differential white blood cell count for the

early diagnosis of bacterial infections in newborn infants. Pediatr Infect Dis J.

1999 Aug;18(8):666-71. PubMed PMID: 10462333.

780: Carlet J. Rapid diagnostic methods in the detection of sepsis. Infect Dis

Clin North Am. 1999 Jun;13(2):483-94, xi. Review. PubMed PMID: 10340179.

781: von Heimburg D, Stieghorst W, Khorram-Sefat R, Pallua N. Procalcitonin--a

sepsis parameter in severe burn injuries. Burns. 1998 Dec;24(8):745-50. PubMed

PMID: 9915676.

782: Petzold L, Guibourdenche J, Boissinot C, Benoist JF, Luton D, Demelier JF,

Porquet D. [Determination of procalcitonin in the diagnosis of maternal-fetal

infections]. Ann Biol Clin (Paris). 1998 Sep-Oct;56(5):599-602. French. PubMed

PMID: 9769506.

783: Benoist JF, Mimoz O, Assicot M. [Procalcitonin in severe trauma]. Ann Biol

Clin (Paris). 1998 Sep-Oct;56(5):571-4. French. PubMed PMID: 9769483.

784: Benoist JF, Mimoz O, Assicot M, Edouard A. Serum procalcitonin, but not

C-reactive protein, identifies sepsis in trauma patients. Clin Chem. 1998

Aug;44(8 Pt 1):1778-9. PubMed PMID: 9702980.

785: Beaune G, Bienvenu F, Pondarré C, Monneret G, Bienvenu J, Souillet G. Serum

procalcitonin rise is only slight in two cases of disseminated aspergillosis.

Infection. 1998 May-Jun;26(3):168-9. PubMed PMID: 9646109.

786: Rau B, Steinbach G, Gansauge F, Mayer JM, Grünert A, Beger HG. The potential

role of procalcitonin and interleukin 8 in the prediction of infected necrosis in

acute pancreatitis. Gut. 1997 Dec;41(6):832-40. PubMed PMID: 9462219; PubMed

Central PMCID: PMC1891610.

787: Gérard Y, Hober D, Assicot M, Alfandari S, Ajana F, Bourez JM, Chidiac C,

Mouton Y, Bohuon C, Wattre P. Procalcitonin as a marker of bacterial sepsis in

patients infected with HIV-1. J Infect. 1997 Jul;35(1):41-6. PubMed PMID:

9279723.

788: al-Nawas B, Shah PM. Procalcitonin in patients with and without

immunosuppression and sepsis. Infection. 1996 Nov-Dec;24(6):434-6. PubMed PMID:

9007590.

789: Gérard Y, Hober D, Petitjean S, Assicot M, Bohuon C, Mouton Y, Wattré P.

High serum procalcitonin level in a 4-year-old liver transplant recipient with a

disseminated candidiasis. Infection. 1995 Sep-Oct;23(5):310-1. PubMed PMID:

8557394.

790: Assicot M, Gendrel D, Carsin H, Raymond J, Guilbaud J, Bohuon C. High serum

procalcitonin concentrations in patients with sepsis and infection. Lancet. 1993

Feb 27;341(8844):515-8. PubMed PMID: 8094770.
