# Supplementary material for: Procalcitonin levels in candidemia versus bacteremia: a systematic review
Source: Crit Care. 2019 May 28;23:190. doi: 10.1186/s13054-019-2481-y (PMC6537202; doi:10.1186/s13054-019-2481-y)
Supplement: Supplementary file 2 — Search output from EMBASE. Full search output from EMBASE. (DOCX 88 kb) [file 13054_2019_2481_MOESM2_ESM.docx]

**EMBASE search output**

**RECORD 1**
**TITLE**
 **Pelvic inflammatory disease and sepsis: the diagnostic challenge**
**AUTHOR NAMES**
 Kornete A.; Grabe Z.
**SOURCE**
 European Journal of Obstetrics Gynecology and Reproductive Biology (2019) 234 (e62). Date of Publication: 1 Mar 2019
**VOLUME**
 234
**FIRST PAGE**
 e62
**DATE OF PUBLICATION**
 1 Mar 2019
**DOI**
 10.1016/j.ejogrb.2018.08.291

**RECORD 2**
**TITLE**
 **First isolation of Ascotricha chartarum from bronchoalveolar lavage of two patients with pulmonary infections**
**AUTHOR NAMES**
 Khan Z.; Ahmad S.; Jeragh A.; Alfouzan W.; Al Foudri H.; Hassan N.; Asadzadeh M.; Joseph L.; Varghese S.
**SOURCE**
 New Microbes and New Infections (2019) 28 (11-16). Date of Publication: 1 Mar 2019
**VOLUME**
 28
**FIRST PAGE**
 11
**LAST PAGE**
 16
**DATE OF PUBLICATION**
 1 Mar 2019
**DOI**
 10.1016/j.nmni.2018.12.002

**RECORD 3**
**TITLE**
 **Role of procalcitonin in bacteremic patients and its potential use in predicting infection etiology**
**AUTHOR NAMES**
 Bassetti M.; Russo A.; Righi E.; Dolso E.; Merelli M.; D’Aurizio F.; Sartor A.; Curcio F.
**SOURCE**
 Expert Review of Anti-Infective Therapy (2019) 17:2 (99-105). Date of Publication: 1 Feb 2019
**VOLUME**
 17
**ISSUE**
 2
**FIRST PAGE**
 99
**LAST PAGE**
 105
**DATE OF PUBLICATION**
 1 Feb 2019
**DOI**
 10.1080/14787210.2019.1562335

**RECORD 4**
**TITLE**
 **SEOM clinical practice guideline: management and prevention of febrile neutropenia in adults with solid tumors (2018)**
**AUTHOR NAMES**
 Carmona-Bayonas A.; Jimenez-Fonseca P.; de Castro E.M.; Mata E.; Biosca M.; Custodio A.; Espinosa J.; Vázquez E.G.; Henao F.; Ayala de la Peña F.
**SOURCE**
 Clinical and Translational Oncology (2019) 21:1 (75-86). Date of Publication: 25 Jan 2019
**VOLUME**
 21
**ISSUE**
 1
**FIRST PAGE**
 75
**LAST PAGE**
 86
**DATE OF PUBLICATION**
 25 Jan 2019
**DOI**
 10.1007/s12094-018-1983-4

**RECORD 5**
**TITLE**
 **Cure of recurring Klebsiella pneumoniae carbapenemase-producing Klebsiella pneumoniae septic shock episodes due to complicated soft tissue infection using a ceftazidime and avibactam-based regimen: A case report**
**AUTHOR NAMES**
 Parruti G.; Frattari A.; Polilli E.; Savini V.; Sciacca A.; Consorte A.; Cibelli D.C.; Agostinone A.; Di Masi F.; Pieri A.; Cacciatore P.; Di Iorio G.; Fazii P.; Spina T.
**SOURCE**
 Journal of Medical Case Reports (2019) 13:1 Article Number: 20. Date of Publication: 22 Jan 2019
**VOLUME**
 13
**ISSUE**
 1
**DATE OF PUBLICATION**
 22 Jan 2019
**DOI**
 10.1186/s13256-018-1934-2

**RECORD 6**
**TITLE**
 **Severe acute pancreatitis with blood infection by Candida glabrata complicated severe agranulocytosis: A case report**
**AUTHOR NAMES**
 Shi R.; Zhou Q.; Fang R.; Xiong X.; Wang Q.
**SOURCE**
 BMC Infectious Diseases (2018) 18:1 Article Number: 706. Date of Publication: 29 Dec 2018
**VOLUME**
 18
**ISSUE**
 1
**DATE OF PUBLICATION**
 29 Dec 2018
**DOI**
 10.1186/s12879-018-3623-6

**RECORD 7**
**TITLE**
 **Successful outcome of disseminated mucormycosis in a 3-year-old child suffering from acute leukaemia: The role of isavuconazole? A case report**
**AUTHOR NAMES**
 Cornu M.; Bruno B.; Loridant S.; Navarin P.; François N.; Lanternier F.; Amzallag-Bellenger E.; Dubos F.; Mazingue F.; Sendid B.
**SOURCE**
 BMC Pharmacology and Toxicology (2018) 19:1 Article Number: 273. Date of Publication: 6 Dec 2018
**VOLUME**
 19
**ISSUE**
 1
**DATE OF PUBLICATION**
 6 Dec 2018
**DOI**
 10.1186/s40360-018-0273-7

**RECORD 8**
**TITLE**
 **Intravascular Catheter–Related Bloodstream Infections**
**AUTHOR NAMES**
 Rupp M.E.; Karnatak R.
**SOURCE**
 Infectious Disease Clinics of North America (2018) 32:4 (765-787). Date of Publication: 1 Dec 2018
**VOLUME**
 32
**ISSUE**
 4
**FIRST PAGE**
 765
**LAST PAGE**
 787
**DATE OF PUBLICATION**
 1 Dec 2018
**DOI**
 10.1016/j.idc.2018.06.002

**RECORD 9**
**TITLE**
 **Successful treatment of catheter related blood stream infection by Millerozyma Farinosa with Micafungin: A case report**
**AUTHOR NAMES**
 Hong S.I.; Suh Y.S.; Kim H.-O.; Bae I.-G.; Shin J.H.; Cho O.-H.
**SOURCE**
 Infection and Chemotherapy (2018) 50:4 (362-366). Date of Publication: 1 Dec 2018
**VOLUME**
 50
**ISSUE**
 4
**FIRST PAGE**
 362
**LAST PAGE**
 366
**DATE OF PUBLICATION**
 1 Dec 2018
**DOI**
 10.3947/ic.2018.50.4.362

**RECORD 10**
**TITLE**
 **Increased presepsin levels are associated with the severity of fungal bloodstream infections**
**AUTHOR NAMES**
 Bamba Y.; Moro H.; Aoki N.; Koizumi T.; Ohshima Y.; Watanabe S.; Sakagami T.; Koya T.; Takada T.; Kikuchi T.
**SOURCE**
 PLoS ONE (2018) 13:10 Article Number: e0206089. Date of Publication: 1 Oct 2018
**VOLUME**
 13
**ISSUE**
 10
**DATE OF PUBLICATION**
 1 Oct 2018
**DOI**
 10.1371/journal.pone.0206089

**RECORD 11**
**TITLE**
 **A study of procalcitonin as biomarker in sepsis patients admitted in super speciality hospital**
**AUTHOR NAMES**
 Neupane N.; Kumari B.; Lakhanpal J.; Bhutani A.; Prabhakar P.K.
**SOURCE**
 Asian Journal of Pharmaceutics (2018) 12:4 Supplement (S1408-S1412). Date of Publication: 1 Oct 2018
**VOLUME**
 12
**ISSUE**
 4
**FIRST PAGE**
 S1408
**LAST PAGE**
 S1412
**DATE OF PUBLICATION**
 1 Oct 2018

**RECORD 12**
**TITLE**
 **Epidemiology, risk factor analysis and comparison of diagnostic methods for rapid diagnosis of fungal pneumonia in critically ill cirrhotics**
**AUTHOR NAMES**
 Kale P.; Khillan V.; Sarin S.K.
**SOURCE**
 Hepatology (2018) 68 Supplement 1 (1338A). Date of Publication: 1 Oct 2018
**VOLUME**
 68
**FIRST PAGE**
 1338A
**DATE OF PUBLICATION**
 1 Oct 2018
**DOI**
 10.1002/hep.30257

**RECORD 13**
**TITLE**
 **Clinical characteristics and predictors of mortality in patients with candidemia: a six-year retrospective study**
**AUTHOR NAMES**
 Jia X.; Li C.; Cao J.; Wu X.; Zhang L.
**SOURCE**
 European Journal of Clinical Microbiology and Infectious Diseases (2018) 37:9 (1717-1724). Date of Publication: 1 Sep 2018
**VOLUME**
 37
**ISSUE**
 9
**FIRST PAGE**
 1717
**LAST PAGE**
 1724
**DATE OF PUBLICATION**
 1 Sep 2018
**DOI**
 10.1007/s10096-018-3304-9

**RECORD 14**
**TITLE**
 **Procalcitonin, c-reactive protein, leukocyte, mean platelet volume levels in bloodstream infections**
**AUTHOR NAMES**
 İrvem A.; Aksaray S.
**SOURCE**
 Journal of Clinical and Analytical Medicine (2018) 9:5 (391-395). Date of Publication: 1 Sep 2018
**VOLUME**
 9
**ISSUE**
 5
**FIRST PAGE**
 391
**LAST PAGE**
 395
**DATE OF PUBLICATION**
 1 Sep 2018
**DOI**
 10.4328/JCAM.5766

**RECORD 15**
**TITLE**
 **Artificial neural networks predict intra-abdominal infection in moderately severe and severe acute pancreatitis**
**AUTHOR NAMES**
 Qiu Q.; Tang L.; Guo Y.; Wen L.-Z.; Wang B.; Chen D.-F.; Liu K.-J.
**SOURCE**
 Journal of Digestive Diseases (2018) 19 Supplement 1 (84-85). Date of Publication: 1 Sep 2018
**VOLUME**
 19
**FIRST PAGE**
 84
**LAST PAGE**
 85
**DATE OF PUBLICATION**
 1 Sep 2018
**DOI**
 10.1111/1751-2980.12665

**RECORD 16**
**TITLE**
 **Inflammatory cytokine expression in patients with sepsis at an intensive care unit**
**AUTHOR NAMES**
 Wang L.; Zhao H.; Wang D.
**SOURCE**
 Experimental and Therapeutic Medicine (2018) 16:3 (2126-2131). Date of Publication: 1 Sep 2018
**VOLUME**
 16
**ISSUE**
 3
**FIRST PAGE**
 2126
**LAST PAGE**
 2131
**DATE OF PUBLICATION**
 1 Sep 2018
**DOI**
 10.3892/etm.2018.6376

**RECORD 17**
**TITLE**
 **Fungal pericarditis due to Aspergillus nidulans: A rare case report**
**AUTHOR NAMES**
 Kombade S.P.; Abhishek K.S.; Nag V.L.
**SOURCE**
 Journal of Clinical and Diagnostic Research (2018) 12:9 (DD05-DD06). Date of Publication: 1 Sep 2018
**VOLUME**
 12
**ISSUE**
 9
**FIRST PAGE**
 DD05
**LAST PAGE**
 DD06
**DATE OF PUBLICATION**
 1 Sep 2018
**DOI**
 10.7860/JCDR/2018/37176.12071

**RECORD 18**
**TITLE**
 **A case of Phaeohyphomycosis caused by Corynespora cassiicola infection**
**AUTHOR NAMES**
 Xie Z.; Wu W.; Meng D.; Zhang Q.; Ma Y.; Liu W.; Chen J.
**SOURCE**
 BMC Infectious Diseases (2018) 18:1 Article Number: 444. Date of Publication: 31 Aug 2018
**VOLUME**
 18
**ISSUE**
 1
**DATE OF PUBLICATION**
 31 Aug 2018
**DOI**
 10.1186/s12879-018-3342-z

**RECORD 19**
**TITLE**
 **C-reactive protein and procalcitonin to discriminate between tuberculosis, Pneumocystis jirovecii pneumonia, and bacterial pneumonia in HIV-infected inpatients meeting WHO criteria for seriously ill: A prospective cohort study**
**AUTHOR NAMES**
 Mendelson F.; Griesel R.; Tiffin N.; Rangaka M.; Boulle A.; Mendelson M.; Maartens G.
**SOURCE**
 BMC Infectious Diseases (2018) 18:1 Article Number: 399. Date of Publication: 14 Aug 2018
**VOLUME**
 18
**ISSUE**
 1
**DATE OF PUBLICATION**
 14 Aug 2018
**DOI**
 10.1186/s12879-018-3303-6

**RECORD 20**
**TITLE**
 **Serum level of procalcitonin in bloodstream infective patients and its role in empirical anti-infective therapy**
**AUTHOR NAMES**
 Yang F.; Zhang N.; Cai H.; Liu M.; Qu J.; Liu Y.
**SOURCE**
 Pharmaceutical Care and Research (2018) 18:4 (256-260). Date of Publication: 1 Aug 2018
**VOLUME**
 18
**ISSUE**
 4
**FIRST PAGE**
 256
**LAST PAGE**
 260
**DATE OF PUBLICATION**
 1 Aug 2018
**DOI**
 10.5428/pcar20180405

**RECORD 21**
**TITLE**
 **Predictive value of serum gelsolin and Gc globulin in sepsis-a pilot study**
**AUTHOR NAMES**
 Horváth-Szalai Z.; Kustán P.; Szirmay B.; Lakatos A.; Christensen P.H.; Huber T.; Bugyi B.; Mühl D.; Ludány A.; Miseta A.; Kovács G.L.; Koszegi T.
**SOURCE**
 Clinical Chemistry and Laboratory Medicine (2018) 56:8 (1373-1382). Date of Publication: 26 Jul 2018
**VOLUME**
 56
**ISSUE**
 8
**FIRST PAGE**
 1373
**LAST PAGE**
 1382
**DATE OF PUBLICATION**
 26 Jul 2018
**DOI**
 10.1515/cclm-2017-0782

**RECORD 22**
**TITLE**
 **Resurgence of global opportunistic multidrug-resistant Stenotrophomonas maltophilia**
**AUTHOR NAMES**
 Gupta P.; Kale P.; Khillan V.
**SOURCE**
 Indian Journal of Critical Care Medicine (2018) 22:7 (503-508). Date of Publication: 1 Jul 2018
**VOLUME**
 22
**ISSUE**
 7
**FIRST PAGE**
 503
**LAST PAGE**
 508
**DATE OF PUBLICATION**
 1 Jul 2018
**DOI**
 10.4103/ijccm.IJCCM_106_18

**RECORD 23**
**TITLE**
 **Up-regulation of chemokine CXCL13 in systemic candidiasis**
**AUTHOR NAMES**
 Li C.; Cao J.; Wang L.; Jia X.; He J.; Zhang L.
**SOURCE**
 Clinical Immunology (2018) 191 (1-9). Date of Publication: 1 Jun 2018
**VOLUME**
 191
**FIRST PAGE**
 1
**LAST PAGE**
 9
**DATE OF PUBLICATION**
 1 Jun 2018
**DOI**
 10.1016/j.clim.2017.11.015

**RECORD 24**
**TITLE**
 **Treatment of septic shock due to candida tropicalis infection in critically ill children**
**AUTHOR NAMES**
 Trisnawati Y.
**SOURCE**
 Pediatric Critical Care Medicine (2018) 19:6 Supplement 1 (98). Date of Publication: 1 Jun 2018
**VOLUME**
 19
**ISSUE**
 6
**FIRST PAGE**
 98
**DATE OF PUBLICATION**
 1 Jun 2018

**RECORD 25**
**TITLE**
 **Elevated levels of IL-17, IL-6 and IL-10 in candidemic patients compared with levels in bacteremic patients**
**AUTHOR NAMES**
 Taj-Aldeen S.J.; Mir F.A.; AbdulWahab A.
**SOURCE**
 Medical Mycology (2018) 56 Supplement 2 (S110). Date of Publication: 1 Jun 2018
**VOLUME**
 56
**FIRST PAGE**
 S110
**DATE OF PUBLICATION**
 1 Jun 2018
**DOI**
 10.1093/mmy/myy036

**RECORD 26**
**TITLE**
 **Molecular epidemiology, risk factor analysis and Comparison of diagnostic methods for Rapid Diagnosis of Fungal Pneumonia in Critically ill Cirrhotics**
**AUTHOR NAMES**
 Kale P.R.; Khillan V.; Mitra L.G.; Sarin S.K.
**SOURCE**
 Medical Mycology (2018) 56 Supplement 2 (S69). Date of Publication: 1 Jun 2018
**VOLUME**
 56
**FIRST PAGE**
 S69
**DATE OF PUBLICATION**
 1 Jun 2018
**DOI**
 10.1093/mmy/myy036

**RECORD 27**
**TITLE**
 **Causes of acute undifferentiated fever and the utility of biomarkers in Chiangrai, northern Thailand**
**AUTHOR NAMES**
 Wangrangsimakul T.; Althaus T.; Mukaka M.; Kantipong P.; Wuthiekanun V.; Chierakul W.; Blacksell S.D.; Day N.P.; Laongnualpanich A.; Paris D.H.
**SOURCE**
 PLoS Neglected Tropical Diseases (2018) 12:5 Article Number: e0006477. Date of Publication: 31 May 2018
**VOLUME**
 12
**ISSUE**
 5
**DATE OF PUBLICATION**
 31 May 2018
**DOI**
 10.1371/journal.pntd.0006477

**RECORD 28**
**TITLE**
 **Influence of pathogen and focus of infection on procalcitonin values in sepsis patients with bacteremia or candidemia**
**AUTHOR NAMES**
 Thomas-Rüddel D.O.; Poidinger B.; Kott M.; Weiss M.; Reinhart K.; Bloos F.
**SOURCE**
 Critical Care (2018) 22:1 Article Number: 128. Date of Publication: 13 May 2018
**VOLUME**
 22
**ISSUE**
 1
**DATE OF PUBLICATION**
 13 May 2018
**DOI**
 10.1186/s13054-018-2050-9

**RECORD 29**
**TITLE**
 **Persistent candidemia despite appropriate fungal therapy: First case of Candida auris from the United Arab Emirates**
**AUTHOR NAMES**
 Alatoom A.; Sartawi M.; Lawlor K.; AbdelWareth L.; Thomsen J.; Nusair A.; Mirza I.
**SOURCE**
 International Journal of Infectious Diseases (2018) 70 (36-37). Date of Publication: 1 May 2018
**VOLUME**
 70
**FIRST PAGE**
 36
**LAST PAGE**
 37
**DATE OF PUBLICATION**
 1 May 2018
**DOI**
 10.1016/j.ijid.2018.02.005

**RECORD 30**
**TITLE**
 **Serum procalcitonin levels of patients with candidemia hospitalized in Intensive Care Units**
**AUTHOR NAMES**
 Yazdani M.R.; Khorvash F.; Hakamifard A.
**SOURCE**
 Journal of Medical Sciences (Taiwan) (2018) 38:3 (113-116). Date of Publication: 1 May 2018
**VOLUME**
 38
**ISSUE**
 3
**FIRST PAGE**
 113
**LAST PAGE**
 116
**DATE OF PUBLICATION**
 1 May 2018
**DOI**
 10.4103/jmedsci.jmedsci_97_17

**RECORD 31**
**TITLE**
 **Predictive efficacy of procalcitonin, platelets, and white blood cells for sepsis in pediatric patients undergoing cardiac surgeries who are admitted to intensive care units: Single-center experience**
**AUTHOR NAMES**
 Surti J.; Jain I.; Shah K.; Mishra A.; Kandre Y.; Garg P.; Shah J.; Shah A.; Tripathi P.
**SOURCE**
 Annals of Pediatric Cardiology (2018) 11:2 (137-142). Date of Publication: 1 May 2018
**VOLUME**
 11
**ISSUE**
 2
**FIRST PAGE**
 137
**LAST PAGE**
 142
**DATE OF PUBLICATION**
 1 May 2018
**DOI**
 10.4103/apc.APC_36_17

**RECORD 32**
**TITLE**
 **Pancitopenic combination**
**AUTHOR NAMES**
 Di Biase J.; Buccella D.; Vannucci M.; Desideri G.
**SOURCE**
 Italian Journal of Medicine (2018) 12:2 Supplement 1 (53). Date of Publication: 1 May 2018
**VOLUME**
 12
**ISSUE**
 2
**FIRST PAGE**
 53
**DATE OF PUBLICATION**
 1 May 2018
**DOI**
 10.4081/itjm.2018.s2

**RECORD 33**
**TITLE**
 **Psoriatic spondyloarthritis: An unsual presentation**
**AUTHOR NAMES**
 Laria A.; Lurati A.M.; Faggio P.; Mazzocchi D.; Marrazza M.G.; Re K.A.; Gilardi A.G.; Tamburello A.; Castelnovo L.; Mazzone A.
**SOURCE**
 Italian Journal of Medicine (2018) 12:2 Supplement 1 (70). Date of Publication: 1 May 2018
**VOLUME**
 12
**ISSUE**
 2
**FIRST PAGE**
 70
**DATE OF PUBLICATION**
 1 May 2018
**DOI**
 10.4081/itjm.2018.s2

**RECORD 34**
**TITLE**
 **Clinical Utility of Serum Procalcitonin Level and Infection in the Neurosurgical Intensive Care Unit**
**AUTHOR NAMES**
 Rotman L.E.; Agee B.S.; Chagoya G.; Davis M.C.; Markert J.M.
**SOURCE**
 World Neurosurgery (2018) 112 (e368-e374). Date of Publication: 1 Apr 2018
**VOLUME**
 112
**FIRST PAGE**
 e368
**LAST PAGE**
 e374
**DATE OF PUBLICATION**
 1 Apr 2018
**DOI**
 10.1016/j.wneu.2018.01.050

**RECORD 35**
**TITLE**
 **Aspergillus Pneumonia in a Patient With Adult-Onset Still Disease Successfully Treated With Anakinra**
**AUTHOR NAMES**
 Bilgin E.; Erden A.; Kilic L.; Sari A.; Armagan B.; Kalyoncu U.; Karadag Ö.
**SOURCE**
 Journal of Clinical Rheumatology (2018) 24:3 (156-158). Date of Publication: 1 Apr 2018
**VOLUME**
 24
**ISSUE**
 3
**FIRST PAGE**
 156
**LAST PAGE**
 158
**DATE OF PUBLICATION**
 1 Apr 2018
**DOI**
 10.1097/RHU.0000000000000631

**RECORD 36**
**TITLE**
 **Late-onset aortoesophageal fistula after treatment of a chronic type B aortic dissection with a three-step approach**
**AUTHOR NAMES**
 Usai M.V.; Gottschalk A.; Schönefeld T.; Schaefers J.F.; Torsello G.B.; Rukosujew A.
**SOURCE**
 Journal of Vascular Surgery Cases and Innovative Techniques (2018) 4:1 (50-53). Date of Publication: 1 Mar 2018
**VOLUME**
 4
**ISSUE**
 1
**FIRST PAGE**
 50
**LAST PAGE**
 53
**DATE OF PUBLICATION**
 1 Mar 2018
**DOI**
 10.1016/j.jvscit.2017.11.010

**RECORD 37**
**TITLE**
 **A rare case of Candida glabrata spondylodiscitis: case report and literature review**
**AUTHOR NAMES**
 Gagliano M.; Marchiani C.; Bandini G.; Bernardi P.; Palagano N.; Cioni E.; Finocchi M.; Bellando Randone S.; Moggi Pignone A.
**SOURCE**
 International Journal of Infectious Diseases (2018) 68 (31-35). Date of Publication: 1 Mar 2018
**VOLUME**
 68
**FIRST PAGE**
 31
**LAST PAGE**
 35
**DATE OF PUBLICATION**
 1 Mar 2018
**DOI**
 10.1016/j.ijid.2018.01.003

**RECORD 38**
**TITLE**
 **Clinical evaluation and comparison of infectious biomarkers in sepsis patients admitted to super-speciality hospital in Punjab**
**AUTHOR NAMES**
 Neupane N.; Lakhanpal J.
**SOURCE**
 Asian Journal of Pharmaceutical and Clinical Research (2018) 11:3 (38). Date of Publication: 1 Mar 2018
**VOLUME**
 11
**ISSUE**
 3
**FIRST PAGE**
 38
**DATE OF PUBLICATION**
 1 Mar 2018

**RECORD 39**
**TITLE**
 **Evaluation of the Use of Novel Biomarkers to Augment Antimicrobial Stewardship Program Activities**
**AUTHOR NAMES**
 Stover K.R.; Kenney R.M.; King S.T.; Gross A.E.
**SOURCE**
 Pharmacotherapy (2018) 38:2 (271-283). Date of Publication: 1 Feb 2018
**VOLUME**
 38
**ISSUE**
 2
**FIRST PAGE**
 271
**LAST PAGE**
 283
**DATE OF PUBLICATION**
 1 Feb 2018
**DOI**
 10.1002/phar.2069

**RECORD 40**
**TITLE**
 **Evaluation of a model to improve collection of blood cultures in patients with sepsis in the emergency room**
**AUTHOR NAMES**
 Mariani B.; Corbella M.; Seminari E.; Sacco L.; Cambieri P.; Capra Marzani F.; Martino I.F.; Bressan M.A.; Muzzi A.; Marena C.; Tinelli C.; Marone P.
**SOURCE**
 European Journal of Clinical Microbiology and Infectious Diseases (2018) 37:2 (241-246). Date of Publication: 1 Feb 2018
**VOLUME**
 37
**ISSUE**
 2
**FIRST PAGE**
 241
**LAST PAGE**
 246
**DATE OF PUBLICATION**
 1 Feb 2018
**DOI**
 10.1007/s10096-017-3122-5

**RECORD 41**
**TITLE**
 **Major publications in the critical care pharmacotherapy literature: January–December 2016**
**AUTHOR NAMES**
 Horner D.; Altshuler D.; Droege C.; Feih J.; Ferguson K.; Fiorenza M.; Greathouse K.; Hamilton L.; Pfaff C.; Roller L.; Stollings J.; Wong A.
**SOURCE**
 Journal of Critical Care (2018) 43 (327-339). Date of Publication: 1 Feb 2018
**VOLUME**
 43
**FIRST PAGE**
 327
**LAST PAGE**
 339
**DATE OF PUBLICATION**
 1 Feb 2018
**DOI**
 10.1016/j.jcrc.2017.09.178

**RECORD 42**
**TITLE**
 **Next-generation sequencing diagnostics of bacteremia in sepsis (Next GeneSiS-Trial) Study protocol of a prospective, observational, noninterventional, multicenter, clinical trial**
**AUTHOR NAMES**
 Brenner T.; Decker S.O.; Grumaz S.; Stevens P.; Bruckner T.; Schmoch T.; Pletz M.W.; Bracht H.; Hofer S.; Marx G.; Weigand M.A.; Sohn K.
**SOURCE**
 Medicine (United States) (2018) 97:6 Article Number: e9868. Date of Publication: 1 Feb 2018
**VOLUME**
 97
**ISSUE**
 6
**DATE OF PUBLICATION**
 1 Feb 2018
**DOI**
 10.1097/MD.0000000000009868

**RECORD 43**
**TITLE**
 **Miliary tuberculosis with co-existing pulmonary cryptococcosis in non-HIV patient without underlying diseases: A case report**
**AUTHOR NAMES**
 Sawai T.; Nakao T.; Koga S.; Ide S.; Yoshioka S.; Matsuo N.; Mukae H.
**SOURCE**
 BMC Pulmonary Medicine (2018) 18:1 Article Number: 6. Date of Publication: 16 Jan 2018
**VOLUME**
 18
**ISSUE**
 1
**DATE OF PUBLICATION**
 16 Jan 2018
**DOI**
 10.1186/s12890-018-0578-8

**RECORD 44**
**TITLE**
 **Evaluation of the (1,3)-ß-D-glucan assay for the diagnosis of neonatal invasive yeast infections**
**AUTHOR NAMES**
 Cornu M.; Goudjil S.; Kongolo G.; Leke A.; Poulain D.; Chouaki T.; Sendid B.
**SOURCE**
 Medical Mycology (2018) 56:1 (78-87). Date of Publication: 1 Jan 2018
**VOLUME**
 56
**ISSUE**
 1
**FIRST PAGE**
 78
**LAST PAGE**
 87
**DATE OF PUBLICATION**
 1 Jan 2018
**DOI**
 10.1093/mmy/myx021

**RECORD 45**
**TITLE**
 **Presepsin: A new marker of catheter related blood stream infections in pediatric patients**
**AUTHOR NAMES**
 Tanır Basaranoglu S.; Karadag-Oncel E.; Aykac K.; Ozsurekci Y.; Aycan A.E.; Cengiz A.B.; Kara A.; Ceyhan M.
**SOURCE**
 Journal of Infection and Chemotherapy (2018) 24:1 (25-30). Date of Publication: 1 Jan 2018
**VOLUME**
 24
**ISSUE**
 1
**FIRST PAGE**
 25
**LAST PAGE**
 30
**DATE OF PUBLICATION**
 1 Jan 2018
**DOI**
 10.1016/j.jiac.2017.08.012

**RECORD 46**
**TITLE**
 **Ochrobactrum anthropi pneumonia: A rare cause of acute respiratory distress syndrome (ARDS)**
**AUTHOR NAMES**
 Hafeez Z.; Ohar J.; Ahmad M.I.; Zeeshan A.; Sunkara P.R.; Marupudi S.; Usman S.; Malik A.
**SOURCE**
 American Journal of Respiratory and Critical Care Medicine (2018) 197:MeetingAbstracts. Date of Publication: 2018
**VOLUME**
 197
**ISSUE**
 MeetingAbstracts
**DATE OF PUBLICATION**
 2018

**RECORD 47**
**TITLE**
 **Evaluation of procalcitonin as a diagnostic marker in neonatal sepsis**
**AUTHOR NAMES**
 Charles M.V.P.; Kalaivani R.; Venkatesh S.; Kali A.; Seetha K.S.
**SOURCE**
 Indian Journal of Pathology and Microbiology (2018) 61:1 (81-84). Date of Publication: 1 Jan 2018
**VOLUME**
 61
**ISSUE**
 1
**FIRST PAGE**
 81
**LAST PAGE**
 84
**DATE OF PUBLICATION**
 1 Jan 2018
**DOI**
 10.4103/IJPM.IJPM_820_16

**RECORD 48**
**TITLE**
 **Pancreatitis is a silent killer in peritoneal dialysis with difficult diagnostic approach**
**AUTHOR NAMES**
 Aliotta R.; Zanoli L.; Lauretta I.; Giunta R.; Ferrario S.; Rastelli S.; Rapisarda S.; Rahbari E.; Rapisarda F.
**SOURCE**
 Clinical Medicine Insights: Case Reports (2018) 11. Date of Publication: 1 Jan 2018
**VOLUME**
 11
**DATE OF PUBLICATION**
 1 Jan 2018
**DOI**
 10.1177/1179547618765761

**RECORD 49**
**TITLE**
 **Clinical value of procalcitonin for suspected nosocomial bloodstream infection**
**AUTHOR NAMES**
 Cha J.K.; Kwon K.H.; Byun S.J.; Ryoo S.R.; Lee J.H.; Chung J.-W.; Huh H.J.; Chae S.L.; Park S.Y.
**SOURCE**
 Korean Journal of Internal Medicine (2018) 33:1 (176-184). Date of Publication: 1 Jan 2018
**VOLUME**
 33
**ISSUE**
 1
**FIRST PAGE**
 176
**LAST PAGE**
 184
**DATE OF PUBLICATION**
 1 Jan 2018
**DOI**
 10.3904/kjim.2016.119

**RECORD 50**
**TITLE**
 **Wrong fungus man!**
**AUTHOR NAMES**
 Steffen S.; Smith M.P.
**SOURCE**
 Journal of General Internal Medicine (2018) 33:2 Supplement 1 (672). Date of Publication: 2018
**VOLUME**
 33
**ISSUE**
 2
**FIRST PAGE**
 672
**DATE OF PUBLICATION**
 2018

**RECORD 51**
**TITLE**
 **Multi-organ failure and Takotsubo cardiomyopathy after offlabel use of baclofen for treatment of alcohol addiction: A case report**
**AUTHOR NAMES**
 Schmoll S.; Romanek K.; Pfab R.; Eyer F.
**SOURCE**
 Clinical Toxicology (2018) 56:6 (568-569). Date of Publication: 2018
**VOLUME**
 56
**ISSUE**
 6
**FIRST PAGE**
 568
**LAST PAGE**
 569
**DATE OF PUBLICATION**
 2018
**DOI**
 10.1080/15563650.2018.1457818

**RECORD 52**
**TITLE**
 **Involvement of aromatic metabolites in the pathogenesis of septic shock**
**AUTHOR NAMES**
 Beloborodova N.V.; Sarshor Y.N.; Bedova A.Yu.; Chernevskaya E.A.; Pautova A.K.
**SOURCE**
 Shock (2018) 50:3 (273-279). Date of Publication: 2018
**VOLUME**
 50
**ISSUE**
 3
**FIRST PAGE**
 273
**LAST PAGE**
 279
**DATE OF PUBLICATION**
 2018
**DOI**
 10.1097/SHK.0000000000001064

**RECORD 53**
**TITLE**
 **Acute respiratory distress syndrome secondary to fat embolism**
**AUTHOR NAMES**
 Heckman A.J.; Helgeson S.; Kwon M.; Patel N.J.; Patel N.M.; Guru P.K.
**SOURCE**
 American Journal of Respiratory and Critical Care Medicine (2018) 197:MeetingAbstracts. Date of Publication: 2018
**VOLUME**
 197
**ISSUE**
 MeetingAbstracts
**DATE OF PUBLICATION**
 2018

**RECORD 54**
**TITLE**
 **Procalcitonin is useful in driving the choice of early antibiotic treatment in patients with bloodstream infections**
**AUTHOR NAMES**
 Murri R.; Mastrorosa I.; Taccari F.; Baroni S.; Giovannenze F.; Palazzolo C.; Lardo S.; Scoppettuolo G.; Ventura G.; Cauda R.; Fantoni M.
**SOURCE**
 European Review for Medical and Pharmacological Sciences (2018) 22:10 (3130-3137). Date of Publication: 2018
**VOLUME**
 22
**ISSUE**
 10
**FIRST PAGE**
 3130
**LAST PAGE**
 3137
**DATE OF PUBLICATION**
 2018

**RECORD 55**
**TITLE**
 **Vertebral osteomyelitis caused by Scedosporium apiospermum in an immunocompetent male: A case report**
**AUTHOR NAMES**
 Cao D.; Li D.; Yu L.; Bi H.; Deng R.; Wang L.
**SOURCE**
 International Journal of Clinical and Experimental Medicine (2018) 11:8 (8672-8676) Article Number: IJCEM0066200. Date of Publication: 2018
**VOLUME**
 11
**ISSUE**
 8
**FIRST PAGE**
 8672
**LAST PAGE**
 8676
**DATE OF PUBLICATION**
 2018

**RECORD 56**
**TITLE**
 **Regional ileitis as a non-specific symptom of Boletus species poisoning**
**AUTHOR NAMES**
 Goncalves R.; Giraud F.; Cordier L.; Ely O.; Labadie M.
**SOURCE**
 Clinical Toxicology (2018) 56:6 (580). Date of Publication: 2018
**VOLUME**
 56
**ISSUE**
 6
**FIRST PAGE**
 580
**DATE OF PUBLICATION**
 2018
**DOI**
 10.1080/15563650.2018.1457818

**RECORD 57**
**TITLE**
 **DcR3, a new biomarker for sepsis, correlates with infection severity and procalcitonin**
**AUTHOR NAMES**
 Gao L.; Yang B.; Zhang H.; Ou Q.; Lin Y.; Zhang M.; Zhang Z.; Kim S.; Wu B.; Wang Z.; Fu L.; Lin J.; Chen R.; Lan R.; Chen J.; Chen W.; Chen L.; Zhang H.; Han D.; Chen J.; Okunieff P.; Lin J.; Zhang L.
**SOURCE**
 Oncotarget (2018) 9:13 (10934-10944). Date of Publication: 2018
**VOLUME**
 9
**ISSUE**
 13
**FIRST PAGE**
 10934
**LAST PAGE**
 10944
**DATE OF PUBLICATION**
 2018
**DOI**
 10.18632/oncotarget.23736

**RECORD 58**
**TITLE**
 **Breathless with dapsone**
**AUTHOR NAMES**
 Munir A.; Thandra A.; Rakhra A.K.; Huang D.; Wells A.D.
**SOURCE**
 American Journal of Respiratory and Critical Care Medicine (2018) 197:MeetingAbstracts. Date of Publication: 2018
**VOLUME**
 197
**ISSUE**
 MeetingAbstracts
**DATE OF PUBLICATION**
 2018

**RECORD 59**
**TITLE**
 **Trichosporon asahii septic thrombophlebitis following lower extremity amputation in an immunocompetent host**
**AUTHOR NAMES**
 Mada P.K.; Ayoade F.; Li A.; Todd J.
**SOURCE**
 BMJ Case Reports (2018) 2018 Article Number: 221441. Date of Publication: 2018
**VOLUME**
 2018
**DATE OF PUBLICATION**
 2018
**DOI**
 10.1136/bcr-2017-221441

**RECORD 60**
**TITLE**
 **Broad adsorption of sepsis-related PAMP and DAMP molecules, mycotoxins, and cytokines from whole blood using CytoSorb® sorbent porous polymer beads**
**AUTHOR NAMES**
 Gruda M.C.; Ruggeberg K.-G.; O'Sullivan P.; Guliashvili T.; Scheirer A.R.; Golobish T.D.; Capponi V.J.; Chan P.P.
**SOURCE**
 PLoS ONE (2018) 13:1 Article Number: e0191676. Date of Publication: 1 Jan 2018
**VOLUME**
 13
**ISSUE**
 1
**DATE OF PUBLICATION**
 1 Jan 2018
**DOI**
 10.1371/journal.pone.0191676

**RECORD 61**
**TITLE**
 **Pulmonary blastomycosis induced acute respiratory distress syndrome requiring veno-venous extra corporeal membrane oxygenation**
**AUTHOR NAMES**
 Low D.; Denson J.L.
**SOURCE**
 American Journal of Respiratory and Critical Care Medicine (2018) 197:MeetingAbstracts. Date of Publication: 2018
**VOLUME**
 197
**ISSUE**
 MeetingAbstracts
**DATE OF PUBLICATION**
 2018

**RECORD 62**
**TITLE**
 **Alternative topical anesthesia for bronchoscopy in a case of severe lidocaine allergy**
**AUTHOR NAMES**
 Hensley M.; Singer B.H.
**SOURCE**
 Respiratory Medicine Case Reports (2018) 23 (90-92). Date of Publication: 1 Jan 2018
**VOLUME**
 23
**FIRST PAGE**
 90
**LAST PAGE**
 92
**DATE OF PUBLICATION**
 1 Jan 2018
**DOI**
 10.1016/j.rmcr.2017.12.010

**RECORD 63**
**TITLE**
 **Pseudomembranous invasive tracheobronchial aspergillosis with fulminant hepatitis and hemophagocytic syndrome**
**AUTHOR NAMES**
 Majima S.; Okachi S.; Asano M.; Wakahara K.; Hashimoto N.; Sato M.; Ishigami M.; Hasegawa Y.
**SOURCE**
 Internal Medicine (2018) 57:16 (2371-2375). Date of Publication: 2018
**VOLUME**
 57
**ISSUE**
 16
**FIRST PAGE**
 2371
**LAST PAGE**
 2375
**DATE OF PUBLICATION**
 2018
**DOI**
 10.2169/internalmedicine.9673-17

**RECORD 64**
**TITLE**
 **Successful treatment of cerebral aspergillosis: Case report of a patient with T-cell large granular lymphocytic leukemia (T-LGL)**
**AUTHOR NAMES**
 Turki A.T.; Rashidi-Alavijeh J.; Dürig J.; Gerken G.; Rath P.-M.; Witzke O.
**SOURCE**
 BMC Infectious Diseases (2017) 17:1 Article Number: 797. Date of Publication: 28 Dec 2017
**VOLUME**
 17
**ISSUE**
 1
**DATE OF PUBLICATION**
 28 Dec 2017
**DOI**
 10.1186/s12879-017-2877-8

**RECORD 65**
**TITLE**
 **Comparison of the power of procalcitonin and c-reactive protein to differentiate between different etiologies of febrile neutropenia in patients with prolonged profound neutropenia**
**AUTHOR NAMES**
 Verlinden A.; De Vroey V.; Roelant E.; Van De Velde A.L.R.; Berneman Z.; Schroyens W.; Gadisseur A.
**SOURCE**
 Blood (2017) 130 Supplement 1. Date of Publication: 1 Dec 2017
**VOLUME**
 130
**DATE OF PUBLICATION**
 1 Dec 2017

**RECORD 66**
**TITLE**
 **Diagnosis and empirical treatment of fever of unknown origin (FUO) in adult neutropenic patients: guidelines of the Infectious Diseases Working Party (AGIHO) of the German Society of Hematology and Medical Oncology (DGHO)**
**AUTHOR NAMES**
 Heinz W.J.; Buchheidt D.; Christopeit M.; von Lilienfeld-Toal M.; Cornely O.A.; Einsele H.; Karthaus M.; Link H.; Mahlberg R.; Neumann S.; Ostermann H.; Penack O.; Ruhnke M.; Sandherr M.; Schiel X.; Vehreschild J.J.; Weissinger F.; Maschmeyer G.
**SOURCE**
 Annals of Hematology (2017) 96:11 (1775-1792). Date of Publication: 1 Nov 2017
**VOLUME**
 96
**ISSUE**
 11
**FIRST PAGE**
 1775
**LAST PAGE**
 1792
**DATE OF PUBLICATION**
 1 Nov 2017
**DOI**
 10.1007/s00277-017-3098-3

**RECORD 67**
**TITLE**
 **Multiple opportunistic infections in a patient of eosinophilic granulomatosis with polyangiitis**
**AUTHOR NAMES**
 Lin P.-C.; Wang L.-S.; Tsai S.-T.; Wu Y.-J.; Lin C.-B.; Lin T.-Y.
**SOURCE**
 International Journal of Antimicrobial Agents (2017) 50 Supplement 2 (S98). Date of Publication: 1 Nov 2017
**VOLUME**
 50
**FIRST PAGE**
 S98
**DATE OF PUBLICATION**
 1 Nov 2017

**RECORD 68**
**TITLE**
 **Refractory hypoxemia in an infant-A case for extracorporeal support**
**AUTHOR NAMES**
 Mehra B.; Sachdev A.; Gupta D.; Gupta N.; Joshi R.
**SOURCE**
 Current Medicine Research and Practice (2017) 7:6 (252-256). Date of Publication: 1 Nov 2017
**VOLUME**
 7
**ISSUE**
 6
**FIRST PAGE**
 252
**LAST PAGE**
 256
**DATE OF PUBLICATION**
 1 Nov 2017
**DOI**
 10.1016/j.cmrp.2017.10.002

**RECORD 69**
**TITLE**
 **Sepsis: A Review of Advances in Management**
**AUTHOR NAMES**
 Rello J.; Valenzuela-Sánchez F.; Ruiz-Rodriguez M.; Moyano S.
**SOURCE**
 Advances in Therapy (2017) 34:11 (2393-2411). Date of Publication: 1 Nov 2017
**VOLUME**
 34
**ISSUE**
 11
**FIRST PAGE**
 2393
**LAST PAGE**
 2411
**DATE OF PUBLICATION**
 1 Nov 2017
**DOI**
 10.1007/s12325-017-0622-8

**RECORD 70**
**TITLE**
 **Concurrent cytomegalovirus colitis and Aspergillus pneumonia in a patient with plasmacytoma**
**AUTHOR NAMES**
 Su M.-Y.; Yu W.-L.; Tan C.-K.
**SOURCE**
 International Journal of Antimicrobial Agents (2017) 50 Supplement 2 (S92). Date of Publication: 1 Nov 2017
**VOLUME**
 50
**FIRST PAGE**
 S92
**DATE OF PUBLICATION**
 1 Nov 2017

**RECORD 71**
**TITLE**
 **First description of spontaneous fungal peritonitis caused by Fusarium solani in a critically ill patient with liver cirrhosis**
**AUTHOR NAMES**
 Mayr U.; Rasch S.; Schmid R.M.; Huber W.; Lahmer T.
**SOURCE**
 New Microbes and New Infections (2017) 20 (16-17). Date of Publication: 1 Nov 2017
**VOLUME**
 20
**FIRST PAGE**
 16
**LAST PAGE**
 17
**DATE OF PUBLICATION**
 1 Nov 2017
**DOI**
 10.1016/j.nmni.2017.08.003

**RECORD 72**
**TITLE**
 **The clinical utility of serum procalcitonin as a diagnostic assay for predicting bacteremia in patients with sepsis**
**AUTHOR NAMES**
 Youkhana K.; Lettich T.; Younus M.; Ismail-Sayed I.; Penupolu S.; Mehta Y.
**SOURCE**
 Chest (2017) 152:4 Supplement 1 (A347). Date of Publication: 1 Oct 2017
**VOLUME**
 152
**ISSUE**
 4
**FIRST PAGE**
 A347
**DATE OF PUBLICATION**
 1 Oct 2017
**DOI**
 10.1016/j.chest.2017.08.373

**RECORD 73**
**TITLE**
 **Procalcitonin for the diagnosis of invasive candidiasis: What is the evidence?**
**AUTHOR NAMES**
 Raineri S.M.; Cortegiani A.; Vitale F.; Iozzo P.; Giarratano A.
**SOURCE**
 Journal of Intensive Care (2017) 5:1 Article Number: 58. Date of Publication: 25 Sep 2017
**VOLUME**
 5
**ISSUE**
 1
**DATE OF PUBLICATION**
 25 Sep 2017
**DOI**
 10.1186/s40560-017-0252-x

**RECORD 74**
**TITLE**
 **Unmet needs in the management of intra-abdominal infections**
**AUTHOR NAMES**
 Montravers P.; Tashk P.; Tran Dinh A.
**SOURCE**
 Expert Review of Anti-Infective Therapy (2017) 15:9 (839-850). Date of Publication: 2 Sep 2017
**VOLUME**
 15
**ISSUE**
 9
**FIRST PAGE**
 839
**LAST PAGE**
 850
**DATE OF PUBLICATION**
 2 Sep 2017
**DOI**
 10.1080/14787210.2017.1372750

**RECORD 75**
**TITLE**
 **Clinical features of patients in negative blood culture with positive catheter-tip culture with Candida species; similar to positive blood culture or not?**
**AUTHOR NAMES**
 Yuji H.; Matsuda A.; Takahashi Y.; Uehara Y.; Abe N.; Naito T.
**SOURCE**
 Mycoses (2017) 60 Supplement 2 (147-148). Date of Publication: 1 Sep 2017
**VOLUME**
 60
**FIRST PAGE**
 147
**LAST PAGE**
 148
**DATE OF PUBLICATION**
 1 Sep 2017
**DOI**
 10.1111/myc.12674

**RECORD 76**
**TITLE**
 **Necrotizing fasciitis resulting in fatal lung aspergillosis: Uncommon pathogenesis. A case report**
**AUTHOR NAMES**
 Mammadli T.; Kim B.-S.; Rennekampff H.-O.; Pallua N.
**SOURCE**
 Journal de Mycologie Medicale (2017) 27:3 (400-406). Date of Publication: 1 Sep 2017
**VOLUME**
 27
**ISSUE**
 3
**FIRST PAGE**
 400
**LAST PAGE**
 406
**DATE OF PUBLICATION**
 1 Sep 2017
**DOI**
 10.1016/j.mycmed.2017.04.007

**RECORD 77**
**TITLE**
 **Combination liposomal amphotericin B, posaconazole and oral amphotericin B for treatment of gastrointestinal Mucorales in an immunocompromised patient**
**AUTHOR NAMES**
 Anderson A.; McManus D.; Perreault S.; Lo Y.-C.; Seropian S.; Topal J.E.
**SOURCE**
 Medical Mycology Case Reports (2017) 17 (11-13). Date of Publication: 1 Sep 2017
**VOLUME**
 17
**FIRST PAGE**
 11
**LAST PAGE**
 13
**DATE OF PUBLICATION**
 1 Sep 2017
**DOI**
 10.1016/j.mmcr.2017.05.004

**RECORD 78**
**TITLE**
 **Clinical features and pathogens of bloodstream infections in patients with hematologic malignancy**
**AUTHOR NAMES**
 Tian L.; Wang J.; Jing H.; Zhao W.; Dong F.; Wan W.; Ke X.; Hu K.
**SOURCE**
 Chinese Journal of Infection and Chemotherapy (2017) 17:5 (504-508) Article Number: 1009-7708(2017)05-0504-05. Date of Publication: 1 Sep 2017
**VOLUME**
 17
**ISSUE**
 5
**FIRST PAGE**
 504
**LAST PAGE**
 508
**DATE OF PUBLICATION**
 1 Sep 2017
**DOI**
 10.16718/j.1009-7708.2017.05.004

**RECORD 79**
**TITLE**
 **Electrical impedance tomography for diagnosis and monitoring of pulmonary function disorders in the intensive care unit - Case report and review of literature**
**AUTHOR NAMES**
 Białka S.; Copik M.; Rybczyk K.; Misiołek H.
**SOURCE**
 Anaesthesiology Intensive Therapy (2017) 49:3 (222-226). Date of Publication: 13 Aug 2017
**VOLUME**
 49
**ISSUE**
 3
**FIRST PAGE**
 222
**LAST PAGE**
 226
**DATE OF PUBLICATION**
 13 Aug 2017
**DOI**
 10.5603/AIT.2017.0040

**RECORD 80**
**TITLE**
 **Case of recurrent severe cellulitis and cutaneous candidiasis during psoriasis treatment with ustekinumab**
**AUTHOR NAMES**
 Miyachi H.; Nakamura Y.; Wakabayashi S.; Iwasawa M.T.; Oikawa A.; Watanabe A.; Matsue H.
**SOURCE**
 Journal of Dermatology (2017) 44:8 (e206-e207). Date of Publication: 1 Aug 2017
**VOLUME**
 44
**ISSUE**
 8
**FIRST PAGE**
 e206
**LAST PAGE**
 e207
**DATE OF PUBLICATION**
 1 Aug 2017
**DOI**
 10.1111/1346-8138.13884

**RECORD 81**
**TITLE**
 **Gastrointestinal: Aspergillus granulomatous gastritis**
**AUTHOR NAMES**
 AbdullGaffar B.; Al-Quraishi H.
**SOURCE**
 Journal of Gastroenterology and Hepatology (Australia) (2017) 32:8 (1421). Date of Publication: 1 Aug 2017
**VOLUME**
 32
**ISSUE**
 8
**FIRST PAGE**
 1421
**DATE OF PUBLICATION**
 1 Aug 2017
**DOI**
 10.1111/jgh.13662

**RECORD 82**
**TITLE**
 **Aspergillus Niger bloodstream infection in gastric cancer after common hepatic artery embolization: A case report**
**AUTHOR NAMES**
 Lin L.; Zhao C.-H.; Yin X.-Y.; Chen Y.-L.; Zhai H.-Y.; Xu C.-W.; Wang Y.; Ge F.-J.; Xu J.-M.
**SOURCE**
 Experimental and Therapeutic Medicine (2017) 14:2 (1427-1432). Date of Publication: 1 Aug 2017
**VOLUME**
 14
**ISSUE**
 2
**FIRST PAGE**
 1427
**LAST PAGE**
 1432
**DATE OF PUBLICATION**
 1 Aug 2017
**DOI**
 10.3892/etm.2017.4693

**RECORD 83**
**TITLE**
 **Combined use of serum (1,3)-β-d-glucan and procalcitonin for the early differential diagnosis between candidaemia and bacteraemia in intensive care units**
**AUTHOR NAMES**
 Giacobbe D.R.; Mikulska M.; Tumbarello M.; Furfaro E.; Spadaro M.; Losito A.R.; Mesini A.; De Pascale G.; Marchese A.; Bruzzone M.; Pelosi P.; Mussap M.; Molin A.; Antonelli M.; Posteraro B.; Sanguinetti M.; Viscoli C.; Del Bono V.
**SOURCE**
 Critical Care (2017) 21:1 Article Number: 176. Date of Publication: 10 Jul 2017
**VOLUME**
 21
**ISSUE**
 1
**DATE OF PUBLICATION**
 10 Jul 2017
**DOI**
 10.1186/s13054-017-1763-5

**RECORD 84**
**TITLE**
 **The impact of real life treatment strategies for Candida peritonitis—A retrospective analysis**
**AUTHOR NAMES**
 Dubler S.; Laun M.; Koch C.; Hecker A.; Weiterer S.; Siegler B.H.; Röhrig R.; Weigand M.A.; Lichtenstern C.
**SOURCE**
 Mycoses (2017) 60:7 (440-446). Date of Publication: 1 Jul 2017
**VOLUME**
 60
**ISSUE**
 7
**FIRST PAGE**
 440
**LAST PAGE**
 446
**DATE OF PUBLICATION**
 1 Jul 2017
**DOI**
 10.1111/myc.12615

**RECORD 85**
**TITLE**
 **Serum procalcitonin measurement is not a useful biomarker in the detection of primary infectious spondylodiscitis**
**AUTHOR NAMES**
 Dubost J.-J.; Lopez J.; Pereira B.; Couderc M.; Tournadre A.; Soubrier M.
**SOURCE**
 Joint Bone Spine (2017) 84:4 (503-504). Date of Publication: 1 Jul 2017
**VOLUME**
 84
**ISSUE**
 4
**FIRST PAGE**
 503
**LAST PAGE**
 504
**DATE OF PUBLICATION**
 1 Jul 2017
**DOI**
 10.1016/j.jbspin.2016.07.012

**RECORD 86**
**TITLE**
 **Clinical spectrum and therapeutic management of systemic lupus erythematosus-associated macrophage activation syndrome: A study of 103 episodes in 89 adult patients**
**AUTHOR NAMES**
 Gavand P.-E.; Serio I.; Arnaud L.; Costedoat-Chalumeau N.; Carvelli J.; Dossier A.; Hinschberger O.; Mouthon L.; Le Guern V.; Korganow A.-S.; Poindron V.; Gourguechon C.; Lavigne C.; Maurier F.; Labro G.; Heymonet M.; Artifoni M.; Viau A.B.; Deligny C.; Sene T.; Terriou L.; Sibilia J.; Mathian A.; Bloch-Queyrat C.; Larroche C.; Amoura Z.; Martin T.
**SOURCE**
 Autoimmunity Reviews (2017) 16:7 (743-749). Date of Publication: 1 Jul 2017
**VOLUME**
 16
**ISSUE**
 7
**FIRST PAGE**
 743
**LAST PAGE**
 749
**DATE OF PUBLICATION**
 1 Jul 2017
**DOI**
 10.1016/j.autrev.2017.05.010

**RECORD 87**
**TITLE**
 **Does serum procalcitonin aid in the diagnosis of bloodstream infection regardless of whether patients exhibit the systemic inflammatory response syndrome?**
**AUTHOR NAMES**
 Arora R.; Campbell J.P.; Simon G.; Sahni N.
**SOURCE**
 Infection (2017) 45:3 (291-298). Date of Publication: 1 Jun 2017
**VOLUME**
 45
**ISSUE**
 3
**FIRST PAGE**
 291
**LAST PAGE**
 298
**DATE OF PUBLICATION**
 1 Jun 2017
**DOI**
 10.1007/s15010-016-0965-0

**RECORD 88**
**TITLE**
 **Trametes polyzona, an emerging filamentous basidiomycete in Réunion Island**
**AUTHOR NAMES**
 Gauthier A.; Jaubert J.; Traversier N.; Lemant J.; Balu L.; Garcia-Hermoso D.; Welti S.; Favel A.; Picot S.; Hoarau G.
**SOURCE**
 Mycoses (2017) 60:6 (412-415). Date of Publication: 1 Jun 2017
**VOLUME**
 60
**ISSUE**
 6
**FIRST PAGE**
 412
**LAST PAGE**
 415
**DATE OF PUBLICATION**
 1 Jun 2017
**DOI**
 10.1111/myc.12609

**RECORD 89**
**TITLE**
 **The “Choosing Wisely” initiative in infectious diseases**
**AUTHOR NAMES**
 Lehmann C.; Berner R.; Bogner J.R.; Cornely O.A.; de With K.; Herold S.; Kern W.V.; Lemmen S.; Pletz M.W.; Ruf B.; Salzberger B.; Stellbrink H.J.; Suttorp N.; Ullmann A.J.; Fätkenheuer G.; Jung N.
**SOURCE**
 Infection (2017) 45:3 (263-268). Date of Publication: 1 Jun 2017
**VOLUME**
 45
**ISSUE**
 3
**FIRST PAGE**
 263
**LAST PAGE**
 268
**DATE OF PUBLICATION**
 1 Jun 2017
**DOI**
 10.1007/s15010-017-0997-0

**RECORD 90**
**TITLE**
 **Successful treatment of granulomatosis with polyangiitis with hydropneumothorax using corticosteroids and immunosuppressant**
**AUTHOR NAMES**
 Shi X.-H.; Zhang Y.-F.; Lu Y.-W.
**SOURCE**
 Experimental and Therapeutic Medicine (2017) 13:6 (3586-3590). Date of Publication: 1 Jun 2017
**VOLUME**
 13
**ISSUE**
 6
**FIRST PAGE**
 3586
**LAST PAGE**
 3590
**DATE OF PUBLICATION**
 1 Jun 2017
**DOI**
 10.3892/etm.2017.4440

**RECORD 91**
**TITLE**
 **Soluble CD14 (presepsin) as a potential biomarker to discriminate infection vs. activity in patients with systemic lupus erythematosus**
**AUTHOR NAMES**
 Posso-Osorio I.; Echeverry A.; Aguirre-Valencia D.; Castaño G.; Tobón G.
**SOURCE**
 Annals of the Rheumatic Diseases (2017) 76 Supplement 2 (1225). Date of Publication: 1 Jun 2017
**VOLUME**
 76
**FIRST PAGE**
 1225
**DATE OF PUBLICATION**
 1 Jun 2017
**DOI**
 10.1136/annrheumdis-2017-eular.2495

**RECORD 92**
**TITLE**
 **Graft Infection Masquerading as Rheumatologic Disease: a Rare Case of Aortobifemoral Graft Infection Presenting as Hypertrophic Osteoarthropathy**
**AUTHOR NAMES**
 Chapman S.A.; Delgadillo D.; MacGuidwin E.; Greenberg J.I.; Jameson A.P.
**SOURCE**
 Annals of Vascular Surgery (2017) 41 (283.e11-283.e18). Date of Publication: 1 May 2017
**VOLUME**
 41
**FIRST PAGE**
 283.e11
**LAST PAGE**
 283.e18
**DATE OF PUBLICATION**
 1 May 2017
**DOI**
 10.1016/j.avsg.2016.10.041

**RECORD 93**
**TITLE**
 **Early Rehospitalization Post–Kidney Transplant Due to Infectious Complications: Can We Predict the Patients at Risk?**
**AUTHOR NAMES**
 Leal R.; Pinto H.; Galvão A.; Rodrigues L.; Santos L.; Romãozinho C.; Macário F.; Alves R.; Campos M.; Mota A.; Figueiredo A.
**SOURCE**
 Transplantation Proceedings (2017) 49:4 (783-786). Date of Publication: 1 May 2017
**VOLUME**
 49
**ISSUE**
 4
**FIRST PAGE**
 783
**LAST PAGE**
 786
**DATE OF PUBLICATION**
 1 May 2017
**DOI**
 10.1016/j.transproceed.2017.01.062

**RECORD 94**
**TITLE**
 **Procalcitonin levels in bloodstream infections caused by different sources and species of bacteria**
**AUTHOR NAMES**
 Yan S.T.; Sun L.C.; Jia H.B.; Gao W.; Yang J.P.; Zhang G.Q.
**SOURCE**
 American Journal of Emergency Medicine (2017) 35:4 (579-583). Date of Publication: 1 Apr 2017
**VOLUME**
 35
**ISSUE**
 4
**FIRST PAGE**
 579
**LAST PAGE**
 583
**DATE OF PUBLICATION**
 1 Apr 2017
**DOI**
 10.1016/j.ajem.2016.12.017

**RECORD 95**
**TITLE**
 **Histoplasma capsulatum in a peripheral blood smear in a non-HIV patient**
**AUTHOR NAMES**
 Jha B.; Gajendra S.
**SOURCE**
 Annals of Hematology (2017) 96:4 (709-710). Date of Publication: 1 Apr 2017
**VOLUME**
 96
**ISSUE**
 4
**FIRST PAGE**
 709
**LAST PAGE**
 710
**DATE OF PUBLICATION**
 1 Apr 2017
**DOI**
 10.1007/s00277-016-2906-5

**RECORD 96**
**TITLE**
 **Infections in the Asia pacific region**
**AUTHOR NAMES**
 Louthrenoo W.
**SOURCE**
 Lupus Science and Medicine (2017) 4 Supplement 1 (A11). Date of Publication: 1 Mar 2017
**VOLUME**
 4
**FIRST PAGE**
 A11
**DATE OF PUBLICATION**
 1 Mar 2017
**DOI**
 10.1136/lupus-2017-000215.21

**RECORD 97**
**TITLE**
 **Fatal invasive aspergillosis: a rare co-infection with an unexpected image presentation in a patient with dengue shock syndrome**
**AUTHOR NAMES**
 Wang H.-C.; Chang K.; Lu P.-L.; Tsai K.-B.; Chen H.-C.
**SOURCE**
 Clinical Respiratory Journal (2017) 11:2 (248-253). Date of Publication: 1 Mar 2017
**VOLUME**
 11
**ISSUE**
 2
**FIRST PAGE**
 248
**LAST PAGE**
 253
**DATE OF PUBLICATION**
 1 Mar 2017
**DOI**
 10.1111/crj.12323

**RECORD 98**
**TITLE**
 **Blood eosinophilia (BEO) in granulomatosis with polyangiitis (GPA): A case report**
**AUTHOR NAMES**
 Alessandra Gregorini G.; Carlassara L.; Possenti S.; Manili L.; Volonghi I.; Gulletta M.
**SOURCE**
 Rheumatology (United Kingdom) (2017) 56 Supplement 3 (iii67-iii68). Date of Publication: 1 Mar 2017
**VOLUME**
 56
**FIRST PAGE**
 iii67
**LAST PAGE**
 iii68
**DATE OF PUBLICATION**
 1 Mar 2017
**DOI**
 10.1093/rheumatology/kex095

**RECORD 99**
**TITLE**
 **Fever of unknown origin revealed to be primary splenic lymphoma: A rare case report with review of the literature**
**AUTHOR NAMES**
 Sun P.-G.; Cheng B.; Wang J.-F.; He P.
**SOURCE**
 Molecular and Clinical Oncology (2017) 6:2 (177-181). Date of Publication: 1 Feb 2017
**VOLUME**
 6
**ISSUE**
 2
**FIRST PAGE**
 177
**LAST PAGE**
 181
**DATE OF PUBLICATION**
 1 Feb 2017
**DOI**
 10.3892/mco.2016.1110

**RECORD 100**
**TITLE**
 **Wickerhamomyces anomalus blood stream infection in a term newborn with pneumonia**
**AUTHOR NAMES**
 Yılmaz-Semerci S.; Demirel G.; Taştekin A.
**SOURCE**
 Turkish Journal of Pediatrics (2017) 59:3 (349-351). Date of Publication: 2017
**VOLUME**
 59
**ISSUE**
 3
**FIRST PAGE**
 349
**LAST PAGE**
 351
**DATE OF PUBLICATION**
 2017
**DOI**
 10.24953/turkjped.2017.03.021

**RECORD 101**
**TITLE**
 **Serum Level of HMGB1 Protein and Inflammatory Markers in Patients with Secondary Peritonitis: Time Course and the Association with Clinical Status**
**AUTHOR NAMES**
 Milić L.; Grigorov I.; Krstić S.; Ćeranić M.S.; Jovanović B.; Stevanović J.; Peško P.
**SOURCE**
 Journal of Medical Biochemistry (2017) 36:1 (44-53). Date of Publication: 1 Jan 2017
**VOLUME**
 36
**ISSUE**
 1
**FIRST PAGE**
 44
**LAST PAGE**
 53
**DATE OF PUBLICATION**
 1 Jan 2017
**DOI**
 10.1515/jomb-2016-0016

**RECORD 102**
**TITLE**
 **A case of Candida septic arthritis with rice body formation in a 2-month-old infant**
**AUTHOR NAMES**
 D‘Aleo F.; Bonanno R.; Midiri A.; Mancuso G.; Cordaro S.; Warm A.; Verduci E.; Beninati C.; Biondo C.
**SOURCE**
 Infezioni in Medicina (2017) 25:4 (374-376). Date of Publication: 2017
**VOLUME**
 25
**ISSUE**
 4
**FIRST PAGE**
 374
**LAST PAGE**
 376
**DATE OF PUBLICATION**
 2017

**RECORD 103**
**TITLE**
 **Hypersensitivity pneumonitis caused by a home ultrasonic humidifier contaminated with Candida guilliermondii**
**AUTHOR NAMES**
 Ando A.; Hagiya H.; Nada T.; Kimura K.; Waseda K.; Rai K.; Hanayama Y.; Otsuka F.
**SOURCE**
 Internal Medicine (2017) 56:22 (3109-3112). Date of Publication: 2017
**VOLUME**
 56
**ISSUE**
 22
**FIRST PAGE**
 3109
**LAST PAGE**
 3112
**DATE OF PUBLICATION**
 2017
**DOI**
 10.2169/internalmedicine.9055-17

**RECORD 104**
**TITLE**
 **Serial measurement of presepsin, procalcitonin, and C-reactive protein in the early postoperative period and the response to antithymocyte globulin administration after heart transplantation**
**AUTHOR NAMES**
 Franeková J.; Sečník P.; Lavríková P.; Kubíček Z.; Hošková L.; Kieslichová E.; Jabor A.
**SOURCE**
 Clinical Transplantation (2017) 31:1 Article Number: e12870. Date of Publication: 1 Jan 2017
**VOLUME**
 31
**ISSUE**
 1
**DATE OF PUBLICATION**
 1 Jan 2017
**DOI**
 10.1111/ctr.12870

**RECORD 105**
**TITLE**
 **Iatrogenic perforation of hypopharynx as a cause of severe descending necrotizing mediastinitis: A case report**
**AUTHOR NAMES**
 Smolar M.; Dzian A.; Hamzik J.; Saniova B.; Laca L.
**SOURCE**
 Neuroendocrinology Letters (2017) 38:5 (325-328). Date of Publication: 2017
**VOLUME**
 38
**ISSUE**
 5
**FIRST PAGE**
 325
**LAST PAGE**
 328
**DATE OF PUBLICATION**
 2017

**RECORD 106**
**TITLE**
 **The Diagnostic Value of Procalcitonin Versus Other Biomarkers in Prediction of Bloodstream Infection**
**AUTHOR NAMES**
 Pan Y.-P.; Fang Y.-P.; Xu Y.-H.; Wang Z.-X.; Shen J.-L.
**SOURCE**
 Clinical Laboratory (2017) 63:2 (277-285). Date of Publication: 2017
**VOLUME**
 63
**ISSUE**
 2
**FIRST PAGE**
 277
**LAST PAGE**
 285
**DATE OF PUBLICATION**
 2017
**DOI**
 10.7754/Clin.Lab.2016.160802

**RECORD 107**
**TITLE**
 **Acute periprosthetic knee infection: Is there still a role for DAIR?**
**AUTHOR NAMES**
 Di Benedetto P.; Di Benedetto E.D.; Salviato D.; Beltrame A.; Gisonni R.; Cainero V.; Causero A.
**SOURCE**
 Acta Biomedica (2017) 88 Supplement 2 (84-91). Date of Publication: 2017
**VOLUME**
 88
**FIRST PAGE**
 84
**LAST PAGE**
 91
**DATE OF PUBLICATION**
 2017
**DOI**
 10.23750/abm.v88i2-S.6518

**RECORD 108**
**TITLE**
 **A rare cause of organizing pneumonia: Idelalisib, an oral inhibitor of phosphoinositide 3-kinase delta**
**AUTHOR NAMES**
 Shakespeare A.; Franco R.; Sikka P.; Bhat U.
**SOURCE**
 American Journal of Respiratory and Critical Care Medicine (2017) 195. Date of Publication: 2017
**VOLUME**
 195
**DATE OF PUBLICATION**
 2017
**DOI**
 10.1164/ajrccmconference.2017.C43

**RECORD 109**
**TITLE**
 **Disseminated histoplasmosis in a patient with common variable immunodeficiency: A coincidence or the result of T cell defects?**
**AUTHOR NAMES**
 Johnson M.E.; Rojas-Moreno C.; Salzer W.; Regunath H.
**SOURCE**
 IDCases (2017) 10 (105-107). Date of Publication: 2017
**VOLUME**
 10
**FIRST PAGE**
 105
**LAST PAGE**
 107
**DATE OF PUBLICATION**
 2017
**DOI**
 10.1016/j.idcr.2017.10.004

**RECORD 110**
**TITLE**
 **Infections in severe alcoholic hepatitis**
**AUTHOR NAMES**
 Karakike E.; Moreno C.; Gustot T.
**SOURCE**
 Annals of Gastroenterology (2017) 30:2 (152-160). Date of Publication: 2017
**VOLUME**
 30
**ISSUE**
 2
**FIRST PAGE**
 152
**LAST PAGE**
 160
**DATE OF PUBLICATION**
 2017
**DOI**
 10.20524/aog.2016.0101

**RECORD 111**
**TITLE**
 **Unusual mushroom poisoning in an immigrant: A case report**
**AUTHOR NAMES**
 Golob N.; Dobaja M.; Brvar M.
**SOURCE**
 Clinical Toxicology (2017) 55:5 (375). Date of Publication: 2017
**VOLUME**
 55
**ISSUE**
 5
**FIRST PAGE**
 375
**DATE OF PUBLICATION**
 2017
**DOI**
 10.1080/15563650.2017.1309792

**RECORD 112**
**TITLE**
 **Acute pulmonary blastomycosis with ards in an immunocompetent patient**
**AUTHOR NAMES**
 Chambers J.; Sehring M.; Smith N.A.
**SOURCE**
 American Journal of Respiratory and Critical Care Medicine (2017) 195. Date of Publication: 2017
**VOLUME**
 195
**DATE OF PUBLICATION**
 2017
**DOI**
 10.1164/ajrccm-conference.2017.A63

**RECORD 113**
**TITLE**
 **Presepsin as a marker of infectious complications during high-dose chemotherapy following autologous hematopoietic stem cell transplantation in lymphoma patients**
**AUTHOR NAMES**
 Dubinina Y.; Sarzhevskiy V.; Melnichenko V.
**SOURCE**
 Bone Marrow Transplantation (2017) 52 Supplement 1 (389-390). Date of Publication: 2017
**VOLUME**
 52
**FIRST PAGE**
 389
**LAST PAGE**
 390
**DATE OF PUBLICATION**
 2017

**RECORD 114**
**TITLE**
 **Invasive pulmonary aspergillosis in patients with chronic obstructive pulmonary disease: A case report and review of the literature**
**AUTHOR NAMES**
 Bao Z.; Chen H.; Zhou M.; Shi G.; Li Q.; Wan H.
**SOURCE**
 Oncotarget (2017) 8:23 (38069-38074). Date of Publication: 2017
**VOLUME**
 8
**ISSUE**
 23
**FIRST PAGE**
 38069
**LAST PAGE**
 38074
**DATE OF PUBLICATION**
 2017
**DOI**
 10.18632/oncotarget.16971

**RECORD 115**
**TITLE**
 **Soluble CD14 as a Diagnostic and Prognostic Biomarker in Hematological Patients with Febrile Neutropenia**
**AUTHOR NAMES**
 Korpelainen S.; Intke C.; Hämäläinen S.; Jantunen E.; Juutilainen A.; Pulkki K.
**SOURCE**
 Disease Markers (2017) 2017 Article Number: 9805609. Date of Publication: 2017
**VOLUME**
 2017
**DATE OF PUBLICATION**
 2017
**DOI**
 10.1155/2017/9805609

**RECORD 116**
**TITLE**
 **Linezolid use in acute respiratory distress syndrome(ARDS) due to pulmonary nocardiosis**
**AUTHOR NAMES**
 Virk J.; Yashi K.; Gill D.; Paul M.P.
**SOURCE**
 American Journal of Respiratory and Critical Care Medicine (2017) 195. Date of Publication: 2017
**VOLUME**
 195
**DATE OF PUBLICATION**
 2017
**DOI**
 10.1164/ajrccm-conference.2017.B66.

**RECORD 117**
**TITLE**
 **The first croatian pediatric patient with geotrichum capitatum respiratory infection**
**ORIGINAL (NON-ENGLISH) TITLE**
 **Prvi hrvatski pedijatrijski bolesnik s geotrichum capitatum respiratornom infekcijom**
**AUTHOR NAMES**
 Markić J.; Polić B.; Kovačević T.; Veljačić D.; Goić-Barišić I.; Tonkić M.
**SOURCE**
 Infektoloski Glasnik (2017) 37:1 (23-26). Date of Publication: 2017
**VOLUME**
 37
**ISSUE**
 1
**FIRST PAGE**
 23
**LAST PAGE**
 26
**DATE OF PUBLICATION**
 2017

**RECORD 118**
**TITLE**
 **Diagnostic value of procalcitomin on pediatric severe infection**
**AUTHOR NAMES**
 Fang C.; Hong A.; Ying D.
**SOURCE**
 Biomedical Research (India) (2017) 28:22 (9898-9902). Date of Publication: 2017
**VOLUME**
 28
**ISSUE**
 22
**FIRST PAGE**
 9898
**LAST PAGE**
 9902
**DATE OF PUBLICATION**
 2017

**RECORD 119**
**TITLE**
 **Acute eosinophilic pneumonia in a patient with influenza infection-a coincidence or an association?**
**AUTHOR NAMES**
 Velani S.; Myers C.; Manvar S.; Van Lunteren E.; Faress J.
**SOURCE**
 American Journal of Respiratory and Critical Care Medicine (2017) 195. Date of Publication: 2017
**VOLUME**
 195
**DATE OF PUBLICATION**
 2017
**DOI**
 10.1164/ajrccm-conference.2017.B42

**RECORD 120**
**TITLE**
 **Early quantitative determination of serum procalcitonin can help distinguishing strains of different bloodstream infections in patients with hematologic diseases**
**AUTHOR NAMES**
 Luo X.; Ren J.; Chen Z.; Yang T.; Hu J.
**SOURCE**
 Blood (2016) 128:22. Date of Publication: 2 Dec 2016
**VOLUME**
 128
**ISSUE**
 22
**DATE OF PUBLICATION**
 2 Dec 2016

**RECORD 121**
**TITLE**
 **Severe pneumonia due to infection with Candida krusei in a case of suspected Middle East respiratory syndrome: A case report and literature review**
**AUTHOR NAMES**
 Tan M.; Wang J.; Hu P.; Wang B.; Xu W.; Chen J.
**SOURCE**
 Experimental and Therapeutic Medicine (2016) 12:6 (4085-4088). Date of Publication: 1 Dec 2016
**VOLUME**
 12
**ISSUE**
 6
**FIRST PAGE**
 4085
**LAST PAGE**
 4088
**DATE OF PUBLICATION**
 1 Dec 2016
**DOI**
 10.3892/etm.2016.3892

**RECORD 122**
**TITLE**
 **The diagnostic role of procalcitonin in differentiation of bloodstream pathogens: A meta-analysis**
**AUTHOR NAMES**
 Liu C.; Li J.; Peng Z.
**SOURCE**
 Critical Care Medicine (2016) 44:12 Supplement 1 (250). Date of Publication: 1 Dec 2016
**VOLUME**
 44
**ISSUE**
 12
**FIRST PAGE**
 250
**DATE OF PUBLICATION**
 1 Dec 2016
**DOI**
 10.1097/01.ccm.0000509372.23544.3f

**RECORD 123**
**TITLE**
 **Bacterial-fungal interactions including quorum sensing, between 2 opportunistic pathogens, resulting in post-traumatic sepsis in a child presenting with a closed femoral fracture**
**AUTHOR NAMES**
 Bradford R.; O'Loughlin K.; Munro A.; Jani B.R.; Singham S.; Cansick J.
**SOURCE**
 Pediatric Infectious Disease Journal (2016) 35:12 (1360-1362). Date of Publication: 28 Nov 2016
**VOLUME**
 35
**ISSUE**
 12
**FIRST PAGE**
 1360
**LAST PAGE**
 1362
**DATE OF PUBLICATION**
 28 Nov 2016
**DOI**
 10.1097/INF.0000000000001337

**RECORD 124**
**TITLE**
 **Successful treatment of severe Pneumocystis pneumonia in an immunosuppressed patient using caspofungin combined with clindamycin: A case report and literature review**
**AUTHOR NAMES**
 Li H.; Huang H.; He H.
**SOURCE**
 BMC Pulmonary Medicine (2016) 16:1 Article Number: 144. Date of Publication: 11 Nov 2016
**VOLUME**
 16
**ISSUE**
 1
**DATE OF PUBLICATION**
 11 Nov 2016
**DOI**
 10.1186/s12890-016-0307-0

**RECORD 125**
**TITLE**
 **Empirical antifungal therapy in critically ill patients with sepsis**
**AUTHOR NAMES**
 Siddharthan T.; Karakousis P.C.; Checkley W.
**SOURCE**
 JAMA - Journal of the American Medical Association (2016) 316:15 (1549-1550). Date of Publication: 18 Oct 2016
**VOLUME**
 316
**ISSUE**
 15
**FIRST PAGE**
 1549
**LAST PAGE**
 1550
**DATE OF PUBLICATION**
 18 Oct 2016
**DOI**
 10.1001/jama.2016.13801

**RECORD 126**
**TITLE**
 **Seeing the forest and not just the trees: A case of recurrent fever, cough, and respiratory failure**
**AUTHOR NAMES**
 Myers M.; Clay R.; Escalante P.
**SOURCE**
 Chest (2016) 150:4 Supplement 1 (1202A). Date of Publication: 1 Oct 2016
**VOLUME**
 150
**ISSUE**
 4
**FIRST PAGE**
 1202A
**DATE OF PUBLICATION**
 1 Oct 2016
**DOI**
 10.1016/j.chest.2016.08.1311

**RECORD 127**
**TITLE**
 **The utility of procalcitonin in the prediction of serious bacterial infection in a tertiary paediatric intensive care unit**
**AUTHOR NAMES**
 Matha S.M.; Rahiman S.N.; Gelbart B.G.; Duke T.D.
**SOURCE**
 Anaesthesia and Intensive Care (2016) 44:5 (607-614). Date of Publication: 1 Sep 2016
**VOLUME**
 44
**ISSUE**
 5
**FIRST PAGE**
 607
**LAST PAGE**
 614
**DATE OF PUBLICATION**
 1 Sep 2016

**RECORD 128**
**TITLE**
 **Combined detection of Serum ferritin and procalcitonin, fungi D glucan is valueable of for AODS and infectious fever disease**
**AUTHOR NAMES**
 Chen L.
**SOURCE**
 International Journal of Rheumatic Diseases (2016) 19 Supplement 2 (103). Date of Publication: 1 Sep 2016
**VOLUME**
 19
**FIRST PAGE**
 103
**DATE OF PUBLICATION**
 1 Sep 2016
**DOI**
 10.1111/1756-185X.12962

**RECORD 129**
**TITLE**
 **An observational study demonstrating a possible link between a procalcitonin driven reduction in antibiotic use and systemic fungal infections**
**AUTHOR NAMES**
 Sheils M.A.; Patel C.; Mohankumar L.; Akhtar N.
**SOURCE**
 Intensive Care Medicine Experimental (2016) 4 Supplement 1. Date of Publication: 1 Sep 2016
**VOLUME**
 4
**DATE OF PUBLICATION**
 1 Sep 2016
**DOI**
 10.1186/s40635-016-0100-7

**RECORD 130**
**TITLE**
 **Rhodotorula mucilaginosa as a cause of recurrent pulmonary infection and liver infiltration in a patient with CLL**
**AUTHOR NAMES**
 Fischer J.; Hamacher L.; Fries J.; Hallek M.; Cornely O.A.; Kochanek M.; Boell B.
**SOURCE**
 Annals of Hematology (2016) 95:9 (1569-1570). Date of Publication: 1 Sep 2016
**VOLUME**
 95
**ISSUE**
 9
**FIRST PAGE**
 1569
**LAST PAGE**
 1570
**DATE OF PUBLICATION**
 1 Sep 2016
**DOI**
 10.1007/s00277-016-2726-7

**RECORD 131**
**TITLE**
 **New role of biomarkers: Mid-regional pro-adrenomedullin, the biomarker of organ failure**
**AUTHOR NAMES**
 Valenzuela-Sánchez F.; Valenzuela-Méndez B.; Rodríguez-Gutiérrez J.F.; Estella-García A.; González-García M.A.
**SOURCE**
 Annals of Translational Medicine (2016) 4:17 Article Number: 329. Date of Publication: 1 Sep 2016
**VOLUME**
 4
**ISSUE**
 17
**DATE OF PUBLICATION**
 1 Sep 2016
**DOI**
 10.21037/atm.2016.08.65

**RECORD 132**
**TITLE**
 **Abdominal Sepsis**
**AUTHOR NAMES**
 De Waele J.J.
**SOURCE**
 Current Infectious Disease Reports (2016) 18:8 Article Number: 23. Date of Publication: 1 Aug 2016
**VOLUME**
 18
**ISSUE**
 8
**DATE OF PUBLICATION**
 1 Aug 2016
**DOI**
 10.1007/s11908-016-0531-z

**RECORD 133**
**TITLE**
 **The intensive care infection score - a novel marker for the prediction of infection and its severity**
**AUTHOR NAMES**
 van der Geest P.J.; Mohseni M.; Linssen J.; Duran S.; de Jonge R.; Groeneveld A.B.J.
**SOURCE**
 Critical Care (2016) 20:1 Article Number: 180. Date of Publication: 7 Jul 2016
**VOLUME**
 20
**ISSUE**
 1
**DATE OF PUBLICATION**
 7 Jul 2016
**DOI**
 10.1186/s13054-016-1366-6

**RECORD 134**
**TITLE**
 **Cutaneous mucormycosis postcosmetic surgery**
**AUTHOR NAMES**
 Al-Tarrah K.; Abdelaty M.; Behbahani A.; Mokaddas E.; Soliman H.; Albader A.
**SOURCE**
 Medicine (United States) (2016) 95:27 Article Number: e4185. Date of Publication: 1 Jul 2016
**VOLUME**
 95
**ISSUE**
 27
**DATE OF PUBLICATION**
 1 Jul 2016
**DOI**
 10.1097/MD.0000000000004185

**RECORD 135**
**TITLE**
 **Improvement in detecting bacterial infection in lower respiratory tract infections using the Intensive Care Infection Score (ICIS)**
**AUTHOR NAMES**
 Kaeslin M.; Brunner S.; Raths J.; Huber A.
**SOURCE**
 LaboratoriumsMedizin (2016) 40:3 (175-182). Date of Publication: 1 Jun 2016
**VOLUME**
 40
**ISSUE**
 3
**FIRST PAGE**
 175
**LAST PAGE**
 182
**DATE OF PUBLICATION**
 1 Jun 2016
**DOI**
 10.1515/labmed-2016-0021

**RECORD 136**
**TITLE**
 **Cryptococcemia in an elderly woman with retroperitoneal diffuse large B-cell lymphoma after rituximab-containing chemotherapy**
**AUTHOR NAMES**
 Cheng M.-W.; Wu A.Y.-J.; Liu C.-P.; Lim K.-H.; Weng S.-L.; Tseng H.-K.
**SOURCE**
 International Journal of Gerontology (2016) 10:2 (112-116). Date of Publication: 1 Jun 2016
**VOLUME**
 10
**ISSUE**
 2
**FIRST PAGE**
 112
**LAST PAGE**
 116
**DATE OF PUBLICATION**
 1 Jun 2016
**DOI**
 10.1016/j.ijge.2015.02.005

**RECORD 137**
**TITLE**
 **Septic-metastasizing Aspergillus-encephalitis mimicking massive cerebral infarction in a heart transplant recipient: A case report**
**AUTHOR NAMES**
 Ballázs C.; Akhyari P.; Mehdiani A.; Kamiya H.; Reinecke P.; Felsberg J.; Saeed D.; Lichtenberg A.; Boeken U.
**SOURCE**
 Experimental and Clinical Transplantation (2016) 14:3 (349-352). Date of Publication: 1 Jun 2016
**VOLUME**
 14
**ISSUE**
 3
**FIRST PAGE**
 349
**LAST PAGE**
 352
**DATE OF PUBLICATION**
 1 Jun 2016
**DOI**
 10.6002/ect.2014.0115

**RECORD 138**
**TITLE**
 **DCD liver transplant infection: Experience from a single centre in China**
**AUTHOR NAMES**
 Tu Z.; Xiang P.; Xu X.; Zhou L.; Zhuang L.; Wu J.; Wang W.; Zheng S.
**SOURCE**
 International Journal of Clinical Practice (2016) 70 Supplement 185 (3-10). Date of Publication: 1 Jun 2016
**VOLUME**
 70
**FIRST PAGE**
 3
**LAST PAGE**
 10
**DATE OF PUBLICATION**
 1 Jun 2016
**DOI**
 10.1111/ijcp.12810

**RECORD 139**
**TITLE**
 **Candida glabrata spondylodiscitis following esophageal surgery**
**AUTHOR NAMES**
 Antonielli E.; Turchi V.; Rocchi F.; Florenzi C.; De Marzi G.; Blasi E.; Baroncelli S.; Crociani A.; Pieralli F.; Nozzoli C.
**SOURCE**
 Italian Journal of Medicine (2016) 10 Supplement 2 (2). Date of Publication: 1 May 2016
**VOLUME**
 10
**FIRST PAGE**
 2
**DATE OF PUBLICATION**
 1 May 2016

**RECORD 140**
**TITLE**
 **Procalcitonin is a useful tool in differentiating Candida and bacterial bloodstream infections in critically ill septic patients outside the Intensive Care Unit**
**AUTHOR NAMES**
 Pieralli F.; Corbo L.; Torrigiani A.; Mannini D.; Antonielli E.; Mancini A.; Corradi F.; Innocenti R.; Moggi Pignone A.; Morettini A.; Nozzoli C.
**SOURCE**
 Italian Journal of Medicine (2016) 10 Supplement 2 (92). Date of Publication: 1 May 2016
**VOLUME**
 10
**FIRST PAGE**
 92
**DATE OF PUBLICATION**
 1 May 2016

**RECORD 141**
**TITLE**
 **Legionnaire's disease treated with levofloxacin and tigecycline**
**AUTHOR NAMES**
 Hase J.; Hauck K.
**SOURCE**
 Journal of General Internal Medicine (2016) 31:2 SUPPL. 1 (S663). Date of Publication: May 2016
**VOLUME**
 31
**ISSUE**
 2
**FIRST PAGE**
 S663
**DATE OF PUBLICATION**
 May 2016

**RECORD 142**
**TITLE**
 **Invasive mucormycosis in chronic granulomatous disease**
**AUTHOR NAMES**
 Al-Otaibi A.M.; Al-Shahrani D.A.; Al-Idrissi E.M.; Al-Abdely H.M.
**SOURCE**
 Saudi Medical Journal (2016) 37:5 (567-569). Date of Publication: 1 May 2016
**VOLUME**
 37
**ISSUE**
 5
**FIRST PAGE**
 567
**LAST PAGE**
 569
**DATE OF PUBLICATION**
 1 May 2016
**DOI**
 10.15537/smj.2016.5.14239

**RECORD 143**
**TITLE**
 **A prospective study to evaluate the role of procalcitonin in differentiating bloodstream infections from non-infectious fevers among patients with lymphoma**
**AUTHOR NAMES**
 Wang X.J.; Chan A.; Shih V.; Tan T.T.; Lim S.T.; Mohamad F.; Tao M.; Quek R.H.H.; Tang T.P.L.
**SOURCE**
 Journal of Clinical Oncology (2016) 34 Supplement 15. Date of Publication: 1 May 2016
**VOLUME**
 34
**DATE OF PUBLICATION**
 1 May 2016

**RECORD 144**
**TITLE**
 **Bloodstream infections in internal medicine**
**AUTHOR NAMES**
 Del Bono V.; Giacobbe D.R.
**SOURCE**
 Virulence (2016) 7:3 (353-365). Date of Publication: 2 Apr 2016
**VOLUME**
 7
**ISSUE**
 3
**FIRST PAGE**
 353
**LAST PAGE**
 365
**DATE OF PUBLICATION**
 2 Apr 2016
**DOI**
 10.1080/21505594.2016.1140296

**RECORD 145**
**TITLE**
 **How to manage lung infiltrates in adults suffering from haematological malignancies outside allogeneic haematopoietic stem cell transplantation**
**AUTHOR NAMES**
 Maschmeyer G.; Donnelly J.P.
**SOURCE**
 British Journal of Haematology (2016) 173:2 (179-189). Date of Publication: 1 Apr 2016
**VOLUME**
 173
**ISSUE**
 2
**FIRST PAGE**
 179
**LAST PAGE**
 189
**DATE OF PUBLICATION**
 1 Apr 2016
**DOI**
 10.1111/bjh.13934

**RECORD 146**
**TITLE**
 **Chaetomium atrobrunneum and Aspergillus fumigatus in multiple tracheal aspirates: Copathogens or symbiosis**
**AUTHOR NAMES**
 Wang H.; Liu Y.; Chen S.C.-A.; Long Y.; Kong F.; Xu Y.-C.
**SOURCE**
 Journal of Microbiology, Immunology and Infection (2016) 49:2 (281-285). Date of Publication: 1 Apr 2016
**VOLUME**
 49
**ISSUE**
 2
**FIRST PAGE**
 281
**LAST PAGE**
 285
**DATE OF PUBLICATION**
 1 Apr 2016
**DOI**
 10.1016/j.jmii.2015.12.011

**RECORD 147**
**TITLE**
 **Elevated levels of procalcitonin during acute liver failure are not associated with sepsis or worth outcome**
**AUTHOR NAMES**
 Mallet M.; Tripon S.; Thabut D.; Rudler M.
**SOURCE**
 Journal of Hepatology (2016) 64:2 SUPPL. 1 (S308-S309). Date of Publication: April 2016
**VOLUME**
 64
**ISSUE**
 2
**FIRST PAGE**
 S308
**LAST PAGE**
 S309
**DATE OF PUBLICATION**
 April 2016

**RECORD 148**
**TITLE**
 **Outbreak of prototheca wickerhamii algaemia and sepsis in a tertiary care chemotherapy oncology unit**
**AUTHOR NAMES**
 Khan I.D.
**SOURCE**
 International Journal of Infectious Diseases (2016) 45 SUPPL. 1 (195). Date of Publication: April 2016
**VOLUME**
 45
**FIRST PAGE**
 195
**DATE OF PUBLICATION**
 April 2016
**DOI**
 10.1016/j.ijid.2016.02.452

**RECORD 149**
**TITLE**
 **Successful treatment of pyogenic liver abscesses by daptomycin**
**AUTHOR NAMES**
 Ye H.; Ge B.; Yu K.; Ma Y.; Liang B.; Xing C.
**SOURCE**
 International Journal of Clinical and Experimental Medicine (2016) 9:2 (4742-4747). Date of Publication: 29 Feb 2016
**VOLUME**
 9
**ISSUE**
 2
**FIRST PAGE**
 4742
**LAST PAGE**
 4747
**DATE OF PUBLICATION**
 29 Feb 2016

**RECORD 150**
**TITLE**
 **Comparative study of plasma endotoxin with procalcitonin levels in diagnosis of bacteremia in intensive care unit patients**
**AUTHOR NAMES**
 Wang T.; Cui Y.-L.; Lin Z.-F.; Chen D.-C.
**SOURCE**
 Chinese Medical Journal (2016) 129:4 (417-423). Date of Publication: 20 Feb 2016
**VOLUME**
 129
**ISSUE**
 4
**FIRST PAGE**
 417
**LAST PAGE**
 423
**DATE OF PUBLICATION**
 20 Feb 2016
**DOI**
 10.4103/0366-6999.176064

**RECORD 151**
**TITLE**
 **Procalcitonin (PCT) levels for ruling-out bacterial coinfection in ICU patients with influenza: A CHAID decision-tree analysis**
**AUTHOR NAMES**
 Rodríguez A.H.; Avilés-Jurado F.X.; Díaz E.; Schuetz P.; Trefler S.I.; Solé-Violán J.; Cordero L.; Vidaur L.; Estella Á.; Pozo Laderas J.C.; Socias L.; Vergara J.C.; Zaragoza R.; Bonastre J.; Guerrero J.E.; Suberviola B.; Cilloniz C.; Restrepo M.I.; Martín-Loeches I.
**SOURCE**
 Journal of Infection (2016) 72:2 (143-151). Date of Publication: 1 Feb 2016
**VOLUME**
 72
**ISSUE**
 2
**FIRST PAGE**
 143
**LAST PAGE**
 151
**DATE OF PUBLICATION**
 1 Feb 2016
**DOI**
 10.1016/j.jinf.2015.11.007

**RECORD 152**
**TITLE**
 **Serum procalcitonin levels distinguish Gram-negative bacterial sepsis from Gram-positive bacterial and fungal sepsis**
**AUTHOR NAMES**
 Li S.; Rong H.; Guo Q.; Chen Y.; Zhang G.; Yang J.
**SOURCE**
 Journal of Research in Medical Sciences (2016) 21:3. Date of Publication: 2016
**VOLUME**
 21
**ISSUE**
 3
**DATE OF PUBLICATION**
 2016

**RECORD 153**
**TITLE**
 **Impact of Candida spp. isolation in the respiratory tract in patients with intensive care unit-acquired pneumonia**
**AUTHOR NAMES**
 Terraneo S.; Ferrer M.; Martín-Loeches I.; Esperatti M.; Di Pasquale M.; Giunta V.; Rinaudo M.; de Rosa F.; Li Bassi G.; Centanni S.; Torres A.
**SOURCE**
 Clinical Microbiology and Infection (2016) 22:1 (94.e1-94.e8). Date of Publication: 1 Jan 2016
**VOLUME**
 22
**ISSUE**
 1
**FIRST PAGE**
 94.e1
**LAST PAGE**
 94.e8
**DATE OF PUBLICATION**
 1 Jan 2016
**DOI**
 10.1016/j.cmi.2015.09.002

**RECORD 154**
**TITLE**
 **Urinary tract infections in intensive care unit patients-a single-centre, 3-year observational study according to the INICC project**
**AUTHOR NAMES**
 Duszyñska W.; Rosenthal V.D.; Szczêsny A.; Woznica E.; Ulfik K.; Ostrowska E.; Litwin A.; Kübler A.
**SOURCE**
 Anaesthesiology Intensive Therapy (2016) 48:1 (1-6). Date of Publication: 2016
**VOLUME**
 48
**ISSUE**
 1
**FIRST PAGE**
 1
**LAST PAGE**
 6
**DATE OF PUBLICATION**
 2016
**DOI**
 10.5603/AIT.2016.0001

**RECORD 155**
**TITLE**
 **The unfavorable risk factors for HIV infected persons with positive blood cultures hospitalized at the National Institute for Infectious Diseases Prof. Dr. Matei Balş in 2015**
**AUTHOR NAMES**
 Popa A.-A.; Ducu G.; Camburu D.; Cozma A.; Podani M.; Dumitriu R.; Gavriliu L.; Benea S.; Benea E.
**SOURCE**
 BMC Infectious Diseases (2016) 16:4. Date of Publication: 2016
**VOLUME**
 16
**ISSUE**
 4
**DATE OF PUBLICATION**
 2016
**DOI**
 10.1186/s12879-016-1877-4

**RECORD 156**
**TITLE**
 **Utility of procalcitonin, c-reactive protein, white blood cell count, neutrophils count and percentage in early diagnosis of bloodstream infection**
**AUTHOR NAMES**
 Pan Y.; Shen J.; Xu Y.; Wang Z.
**SOURCE**
 Chinese Journal of Infection and Chemotherapy (2016) 16:5 (571-577) Article Number: 1009-7708(2016)05-0571-07. Date of Publication: 2016
**VOLUME**
 16
**ISSUE**
 5
**FIRST PAGE**
 571
**LAST PAGE**
 577
**DATE OF PUBLICATION**
 2016
**DOI**
 10.16718/j.1009-7708.2016.05.008

**RECORD 157**
**TITLE**
 **Antibiotic de-escalation in the intensive therapy unit- A review**
**AUTHOR NAMES**
 Sharma J.; Kosey S.; Kumar R.
**SOURCE**
 International Journal of Pharmaceutical Sciences and Research (2016) 7:9 (3596-3601). Date of Publication: 2016
**VOLUME**
 7
**ISSUE**
 9
**FIRST PAGE**
 3596
**LAST PAGE**
 3601
**DATE OF PUBLICATION**
 2016
**DOI**
 10.13040/IJPSR.0975-8232.7(9).3596-01

**RECORD 158**
**TITLE**
 **Long chain polyphosphates identified in infectious fever patients in the department of hematology**
**AUTHOR NAMES**
 Yang X.; Wan M.; Yang K.; Chen F.
**SOURCE**
 Acta Medica Mediterranea (2016) 32:2 (377-383). Date of Publication: 2016
**VOLUME**
 32
**ISSUE**
 2
**FIRST PAGE**
 377
**LAST PAGE**
 383
**DATE OF PUBLICATION**
 2016
**DOI**
 10.19193/0393-6384_2016_2_57

**RECORD 159**
**TITLE**
 **Polymicrobial intensive care unit-acquired pneumonia: Prevalence, microbiology and outcome**
**AUTHOR NAMES**
 Ferrer M.; Difrancesco L.F.; Liapikou A.; Rinaudo M.; Carbonara M.; Li Bassi G.; Gabarrus A.; Torres A.
**SOURCE**
 Critical Care (2015) 19:1 Article Number: 450. Date of Publication: 23 Dec 2015
**VOLUME**
 19
**ISSUE**
 1
**DATE OF PUBLICATION**
 23 Dec 2015
**DOI**
 10.1186/s13054-015-1165-5

**RECORD 160**
**TITLE**
 **Tigecycline lock therapy for catheter-related bloodstream infection caused by KPC-producing Klebsiella pneumoniae in two pediatric hematological patients**
**AUTHOR NAMES**
 Foresti S.; Di Bella S.; Rovelli A.; Sala A.; Verna M.; Bisi L.; Nisii C.; Gori A.
**SOURCE**
 Antimicrobial Agents and Chemotherapy (2015) 59:12 (7919-7920). Date of Publication: 1 Dec 2015
**VOLUME**
 59
**ISSUE**
 12
**FIRST PAGE**
 7919
**LAST PAGE**
 7920
**DATE OF PUBLICATION**
 1 Dec 2015
**DOI**
 10.1128/AAC.01855-15

**RECORD 161**
**TITLE**
 **Comparison of serum procalcitonin in respiratory infections and bloodstream infections**
**AUTHOR NAMES**
 Zhu Y.; Yuan Y.; Huang H.
**SOURCE**
 International Journal of Clinical and Experimental Medicine (2015) 8:11 (21586-21592). Date of Publication: 30 Nov 2015
**VOLUME**
 8
**ISSUE**
 11
**FIRST PAGE**
 21586
**LAST PAGE**
 21592
**DATE OF PUBLICATION**
 30 Nov 2015

**RECORD 162**
**TITLE**
 **Diagnostic Accuracy of Procalcitonin for Predicting Blood Culture Results in Patients with Suspected Bloodstream Infection**
**AUTHOR NAMES**
 Oussalah A.; Ferrand J.; Filhine-Tresarrieu P.; Aissa N.; Aimone-Gastin I.; Namour F.; Garcia M.; Lozniewski A.; Gueánt J.-L.
**SOURCE**
 Medicine (United States) (2015) 94:44 (e1774). Date of Publication: 1 Nov 2015
**VOLUME**
 94
**ISSUE**
 44
**FIRST PAGE**
 e1774
**DATE OF PUBLICATION**
 1 Nov 2015
**DOI**
 10.1097/MD.0000000000001774

**RECORD 163**
**TITLE**
 **Bacterial infections in children treated for haematological malignancy**
**AUTHOR NAMES**
 Naidu G.; Wainwright R.; MacKinnon D.; Poiyadjis S.; Rowe B.; Izu A.; Madhi S.
**SOURCE**
 Pediatric Blood and Cancer (2015) 62 Supplement 4 (S387). Date of Publication: 1 Nov 2015
**VOLUME**
 62
**FIRST PAGE**
 S387
**DATE OF PUBLICATION**
 1 Nov 2015
**DOI**
 10.1002/pbc.25715

**RECORD 164**
**TITLE**
 **Rapid diagnosis of neonatal sepsis using molecular techniques**
**AUTHOR NAMES**
 Ramilo O.
**SOURCE**
 Journal of Perinatal Medicine (2015) 43 SUPPL. 1. Date of Publication: October 2015
**VOLUME**
 43
**DATE OF PUBLICATION**
 October 2015
**DOI**
 10.1515/jpm-2015-2001

**RECORD 165**
**TITLE**
 **An unusual case of accelerated variant of idiopathic pulmonary fibrosis presenting with blastomyces dermatitidis on serology**
**AUTHOR NAMES**
 Khan M.S.; Huebert C.A.; Van Schooneveld T.
**SOURCE**
 American Journal of Respiratory and Critical Care Medicine (2015) 191 MeetingAbstracts. Date of Publication: 2015
**VOLUME**
 191
**DATE OF PUBLICATION**
 2015

**RECORD 166**
**TITLE**
 **Procalcitonin as diagnostic marker in febrile cancer patients**
**AUTHOR NAMES**
 Fioroni I.; Vincenzi B.; Pantano F.; Angeletti S.; Dicuonzo G.; Zoccoli A.; Santini D.; Tonini G.
**SOURCE**
 Annals of Oncology (2015) 26 SUPPL. 6. Date of Publication: October 2015
**VOLUME**
 26
**DATE OF PUBLICATION**
 October 2015
**DOI**
 10.1093/annonc/mdv348.16

**RECORD 167**
**TITLE**
 **A molecular biomarker to diagnose community-acquired pneumonia on intensive care unit admission**
**AUTHOR NAMES**
 Scicluna B.P.; Klein Klouwenberg P.M.C.; Van Vught L.A.; Wiewel M.A.; Ong D.S.Y.; Zwinderman A.H.; Franitza M.; Toliat M.R.; Nürnberg P.; Hoogendijk A.J.; Horn J.; Cremer O.L.; Schultz M.J.; Bonten M.J.; Van Der Poll T.
**SOURCE**
 American Journal of Respiratory and Critical Care Medicine (2015) 192:7 (826-835). Date of Publication: 1 Oct 2015
**VOLUME**
 192
**ISSUE**
 7
**FIRST PAGE**
 826
**LAST PAGE**
 835
**DATE OF PUBLICATION**
 1 Oct 2015
**DOI**
 10.1164/rccm.201502-0355OC

**RECORD 168**
**TITLE**
 **A unique case of inhaled mushroom palytoxin poisoning presenting as acute respiratory distress syndrome (ARDS)**
**AUTHOR NAMES**
 Kyereme-Tuah E.; Thakur L.K.; Layon A.J.
**SOURCE**
 American Journal of Respiratory and Critical Care Medicine (2015) 191 MeetingAbstracts. Date of Publication: 2015
**VOLUME**
 191
**DATE OF PUBLICATION**
 2015

**RECORD 169**
**TITLE**
 **Features of systemic inflammation in patients with severe community-acquired (CAP) pneumonia of different etiology in HIV-infected patients**
**AUTHOR NAMES**
 Pertseva T.; Kireyeva T.; Bielosludtseva K.
**SOURCE**
 European Respiratory Journal (2015) 46 SUPPL. 59. Date of Publication: 1 Sep 2015
**VOLUME**
 46
**DATE OF PUBLICATION**
 1 Sep 2015
**DOI**
 10.1183/13993003.congress2015.PA2645

**RECORD 170**
**TITLE**
 **Antifungal therapy in patients with pulmonary Candida spp. colonization may have no beneficial effects**
**AUTHOR NAMES**
 Lindau S.; Nadermann M.; Ackermann H.; Bingold T.M.; Stephan C.; Kempf V.A.J.; Herzberger P.; Beiras-Fernandez A.; Zacharowski K.; Meybohm P.
**SOURCE**
 Journal of Intensive Care (2015) 3:1 Article Number: 31. Date of Publication: 3 Jul 2015
**VOLUME**
 3
**ISSUE**
 1
**DATE OF PUBLICATION**
 3 Jul 2015
**DOI**
 10.1186/s40560-015-0097-0

**RECORD 171**
**TITLE**
 **Role of procalcitonin as an early market in diagnosis and follow up of surgical site infection in Al Azhar University Hospital-New Damietta, Egypt**
**AUTHOR NAMES**
 Abdelnasser M.; Gohar M.; Saleh M.; Abdelhamid M.; Balboula M.
**SOURCE**
 Antimicrobial Resistance and Infection Control (2015) 4 SUPPL. 1. Date of Publication: June 16, 2015
**VOLUME**
 4
**DATE OF PUBLICATION**
 June 16, 2015

**RECORD 172**
**TITLE**
 **Diagnostic value of serum procalcitonin for infection in the immunocompromised critically ill patients with suspected infection**
**AUTHOR NAMES**
 Yu X.; Ma X.; Ai Y.
**SOURCE**
 Zhonghua Wei Zhong Bing Ji Jiu Yi Xue (2015) 27:6 (477-483). Date of Publication: 1 Jun 2015
**VOLUME**
 27
**ISSUE**
 6
**FIRST PAGE**
 477
**LAST PAGE**
 483
**DATE OF PUBLICATION**
 1 Jun 2015
**DOI**
 10.3760/cma.j.issn.2095-4352.2015.06.012

**RECORD 173**
**TITLE**
 **Diagnostic value of serum procalcitonin for infection in the immunocompromised critically ill patients with suspected infection**
**AUTHOR NAMES**
 Yu X.; Ma X.; Ai Y.
**SOURCE**
 Chinese Critical Care Medicine (2015) 27:6 (477-483). Date of Publication: 1 Jun 2015
**VOLUME**
 27
**ISSUE**
 6
**FIRST PAGE**
 477
**LAST PAGE**
 483
**DATE OF PUBLICATION**
 1 Jun 2015
**DOI**
 10.3760/cma.j.issn.2095-4352.2015.06.012

**RECORD 174**
**TITLE**
 **Accumulation of air and a retroperitoneal abscess during typeB influenza virus infection in an older adult**
**AUTHOR NAMES**
 Saitoh M.; Wajima N.; Yamaya M.
**SOURCE**
 Geriatrics and Gerontology International (2015) 15:5 (668-669). Date of Publication: 1 May 2015
**VOLUME**
 15
**ISSUE**
 5
**FIRST PAGE**
 668
**LAST PAGE**
 669
**DATE OF PUBLICATION**
 1 May 2015
**DOI**
 10.1111/ggi.12452

**RECORD 175**
**TITLE**
 **Resolution of septic shock in a patient with myelomeningocele (spina bifida) complicated with deforming elephantiasis nostras verrucosa**
**AUTHOR NAMES**
 Manzione A.; Barberi S.; Scrivano J.; Maresca B.; Moioli A.; Federico Salvi P.; Stoppacciaro A.; Pucci E.; Mené P.; Punzo G.; Tammaro A.; Gaspari A.A.
**SOURCE**
 Journal of the American Academy of Dermatology (2015) 72:5 SUPPL. 1 (AB151). Date of Publication: May 2015
**VOLUME**
 72
**ISSUE**
 5
**FIRST PAGE**
 AB151
**DATE OF PUBLICATION**
 May 2015

**RECORD 176**
**TITLE**
 **Characteristics of serum endocan levels in infection**
**AUTHOR NAMES**
 Seo K.; Kitazawa T.; Yoshino Y.; Koga I.; Ota Y.
**SOURCE**
 PLoS ONE (2015) 10:4 Article Number: e0123358. Date of Publication: 20 Apr 2015
**VOLUME**
 10
**ISSUE**
 4
**DATE OF PUBLICATION**
 20 Apr 2015
**DOI**
 10.1371/journal.pone.0123358

**RECORD 177**
**TITLE**
 **Guidelines for management of intra-abdominal infections**
**AUTHOR NAMES**
 Montravers P.; Dupont H.; Leone M.; Constantin J.-M.; Mertes P.-M.; Laterre P.-F.; Misset B.; Bru J.-P.; Gauzit R.; Sotto A.; Bru J.-P.; Hamy A.; Tuech J.-J.
**SOURCE**
 Anaesthesia Critical Care and Pain Medicine (2015) 34:2 (117-130). Date of Publication: 1 Apr 2015
**VOLUME**
 34
**ISSUE**
 2
**FIRST PAGE**
 117
**LAST PAGE**
 130
**DATE OF PUBLICATION**
 1 Apr 2015
**DOI**
 10.1016/j.accpm.2015.03.005

**RECORD 178**
**TITLE**
 **Potential use of procalcitonin as biomarker for bacterial sepsis inpatients with or without acute kidney injury**
**AUTHOR NAMES**
 Nakamura Y.; Murai A.; Mizunuma M.; Ohta D.; Kawano Y.; Matsumoto N.; Nishida T.; Ishikura H.
**SOURCE**
 Journal of Infection and Chemotherapy (2015) 21:4 (257-263). Date of Publication: 1 Apr 2015
**VOLUME**
 21
**ISSUE**
 4
**FIRST PAGE**
 257
**LAST PAGE**
 263
**DATE OF PUBLICATION**
 1 Apr 2015
**DOI**
 10.1016/j.jiac.2014.12.001

**RECORD 179**
**TITLE**
 **A case of acute fibrinous and organizing pneumonia during early postoperative period after lung transplantation**
**AUTHOR NAMES**
 Alici I.O.; Yekeler E.; Yazicioglu A.; Turan S.; Tezer-Tekce Y.; Demirag F.; Karaoglanoglu N.
**SOURCE**
 Transplantation Proceedings (2015) 47:3 (836-840). Date of Publication: 1 Apr 2015
**VOLUME**
 47
**ISSUE**
 3
**FIRST PAGE**
 836
**LAST PAGE**
 840
**DATE OF PUBLICATION**
 1 Apr 2015
**DOI**
 10.1016/j.transproceed.2015.02.002

**RECORD 180**
**TITLE**
 **Unusual foreign body in the sigmoid colon, chronic alcohol abuse, and Fournier gangrene: A case report**
**AUTHOR NAMES**
 Schulz D.; Mohor G.S.; Solovan C.
**SOURCE**
 Clinical Interventions in Aging (2015) 10 (673-677). Date of Publication: 31 Mar 2015
**VOLUME**
 10
**FIRST PAGE**
 673
**LAST PAGE**
 677
**DATE OF PUBLICATION**
 31 Mar 2015
**DOI**
 10.2147/CIA.S79609

**RECORD 181**
**TITLE**
 **Differential diagnosis of bacterial from candidal bloodstream infections in ICU patients: The role of procalcitonin**
**AUTHOR NAMES**
 Angelopoulos E.; Perivolioti E.; Kokkoris S.; Douka E.; Barbouti E.; Temperekidis P.; Vrettou C.; Psachoulia C.; Poulakou G.; Zakynthinos S.; Routsi C.
**SOURCE**
 Critical Care (2015) 19 SUPPL. 1 (S24). Date of Publication: 16 Mar 2015
**VOLUME**
 19
**FIRST PAGE**
 S24
**DATE OF PUBLICATION**
 16 Mar 2015
**DOI**
 10.1186/cc14148

**RECORD 182**
**TITLE**
 **Cutaneous mucormycosis in the ICU**
**AUTHOR NAMES**
 Herrero E.H.; Sánchez M.; Agrifoglio A.; Cachafeiro L.; Asensio M.J.; Galván B.; De Lorenzo A.G.
**SOURCE**
 Critical Care (2015) 19 SUPPL. 1 (S30-S31). Date of Publication: 16 Mar 2015
**VOLUME**
 19
**FIRST PAGE**
 S30
**LAST PAGE**
 S31
**DATE OF PUBLICATION**
 16 Mar 2015
**DOI**
 10.1186/cc14168

**RECORD 183**
**TITLE**
 **The value of lipopolysaccharide binding protein for diagnosis of late-onset neonatal sepsis in very low birth weight infants**
**AUTHOR NAMES**
 Leante-Castellanos J.L.; De Guadiana-Romualdo L.G.; Fuentes-Gutiérrez C.; Hernando-Holgado A.; García-González A.; Jiménez-Santos E.
**SOURCE**
 Journal of Perinatal Medicine (2015) 43:2 (253-257). Date of Publication: 1 Mar 2015
**VOLUME**
 43
**ISSUE**
 2
**FIRST PAGE**
 253
**LAST PAGE**
 257
**DATE OF PUBLICATION**
 1 Mar 2015
**DOI**
 10.1515/jpm-2014-0010

**RECORD 184**
**TITLE**
 **Development of eosinophilic granulomatosis with poliangiitis (Churg-Strauss syndrome) and brain tumor in a patient after more than 7 years of omalizumab use: A case report**
**AUTHOR NAMES**
 Borekci S.; Aydin O.; Hatemi G.; Gemicioglu B.
**SOURCE**
 International Journal of Immunopathology and Pharmacology (2015) 28:1 (134-137). Date of Publication: 1 Mar 2015
**VOLUME**
 28
**ISSUE**
 1
**FIRST PAGE**
 134
**LAST PAGE**
 137
**DATE OF PUBLICATION**
 1 Mar 2015
**DOI**
 10.1177/0394632015572567

**RECORD 185**
**TITLE**
 **Early- and late-onset severe pneumonia after renal transplantation**
**AUTHOR NAMES**
 Tu G.; Ju M.; Zheng Y.; Xu M.; Rong R.; Zhu D.; Zhu T.; Luo Z.
**SOURCE**
 International Journal of Clinical and Experimental Medicine (2015) 8:1 (1324-1332). Date of Publication: 30 Jan 2015
**VOLUME**
 8
**ISSUE**
 1
**FIRST PAGE**
 1324
**LAST PAGE**
 1332
**DATE OF PUBLICATION**
 30 Jan 2015

**RECORD 186**
**TITLE**
 **Rapid detection of health-care-associated bloodstream infection in critical care using Multipathogen real-time polymerase chain reaction technology: A diagnostic accuracy study and systematic review**
**AUTHOR NAMES**
 Warhurst G.; Dunn G.; Chadwick P.; Blackwood B.; McAuley D.; Perkins G.D.; McMullan R.; Gates S.; Bentley A.; Young D.; Carlson G.L.; Dark P.
**SOURCE**
 Health Technology Assessment (2015) 19:35 (1-141). Date of Publication: 1 May 2015
**VOLUME**
 19
**ISSUE**
 35
**FIRST PAGE**
 1
**LAST PAGE**
 141
**DATE OF PUBLICATION**
 1 May 2015
**DOI**
 10.3310/hta19350

**RECORD 187**
**TITLE**
 **Alterations of serum cytokine levels and their relation with inflammatory markers in candidemia**
**AUTHOR NAMES**
 Akin H.; Akalin H.; Budak F.; Ener B.; Ocakoğlu G.; Gürcüoğlu E.; Göral G.; Oral H.B.
**SOURCE**
 Medical Mycology (2015) 53:3 (258-268). Date of Publication: 2015
**VOLUME**
 53
**ISSUE**
 3
**FIRST PAGE**
 258
**LAST PAGE**
 268
**DATE OF PUBLICATION**
 2015
**DOI**
 10.1093/mmy/myu084

**RECORD 188**
**TITLE**
 **Fluoroquinolones: Another line in the long list of their collateral damage record**
**AUTHOR NAMES**
 Paiva J.A.; Pereira J.M.
**SOURCE**
 Critical Care Medicine (2015) 43:3 (708-710). Date of Publication: 4 Mar 2015
**VOLUME**
 43
**ISSUE**
 3
**FIRST PAGE**
 708
**LAST PAGE**
 710
**DATE OF PUBLICATION**
 4 Mar 2015
**DOI**
 10.1097/CCM.0000000000000802

**RECORD 189**
**TITLE**
 **Febrile neutropenia in children treated for malignancy**
**AUTHOR NAMES**
 Barton C.D.; Waugh L.K.; Nielsen M.J.; Paulus S.
**SOURCE**
 Journal of Infection (2015) 71:S1 (S27-S35). Date of Publication: 1 Jun 2015
**VOLUME**
 71
**ISSUE**
 S1
**FIRST PAGE**
 S27
**LAST PAGE**
 S35
**DATE OF PUBLICATION**
 1 Jun 2015
**DOI**
 10.1016/j.jinf.2015.04.026

**RECORD 190**
**TITLE**
 **Procalcitonin is a marker of gram-negative bacteremia in patients with sepsis**
**AUTHOR NAMES**
 Guo S.Y.; Zhou Y.; Hu Q.F.; Yao J.; Wang H.
**SOURCE**
 American Journal of the Medical Sciences (2015) 349:6 (499-504). Date of Publication: 6 Jun 2015
**VOLUME**
 349
**ISSUE**
 6
**FIRST PAGE**
 499
**LAST PAGE**
 504
**DATE OF PUBLICATION**
 6 Jun 2015
**DOI**
 10.1097/MAJ.0000000000000477

**RECORD 191**
**TITLE**
 **Assessment of bloodstream infections and risk factors in an intensive care unit**
**AUTHOR NAMES**
 Süner A.; Karaoğlan İ.; Mete A.Ö.; Namiduru M.; Boşnak V.; Baydar İ.
**SOURCE**
 Turkish Journal of Medical Sciences (2015) 45:6 (1243-1250). Date of Publication: 2015
**VOLUME**
 45
**ISSUE**
 6
**FIRST PAGE**
 1243
**LAST PAGE**
 1250
**DATE OF PUBLICATION**
 2015
**DOI**
 10.3906/sag-1303-41

**RECORD 192**
**TITLE**
 **Invasive Candida infections and the harm from antibacterial drugs in critically ill patients: Data from a randomized, controlled trial to determine the role of ciprofloxacin, piperacillin-tazobactam, meropenem, and cefuroxime**
**AUTHOR NAMES**
 Jensen J.-U.S.; Hein L.H.; Lundgren B.; Bestle M.H.; Mohr T.; Andersen M.H.; Løken J.; Tousi H.; Søe-Jensen P.; Lauritsen A.Ø.; Strange D.; Petersen J.A.; Thormar K.; Larsen K.M.; Drenck N.-E.; Helweg-Larsen J.; Johansen M.E.; Reinholdt K.; Møller J.K.; Olesen B.; Arendrup M.C.; Østergaard C.; Cozzi-Lepri A.; Grarup J.; Lundgren J.D.
**SOURCE**
 Critical Care Medicine (2015) 43:3 (594-602). Date of Publication: 4 Mar 2015
**VOLUME**
 43
**ISSUE**
 3
**FIRST PAGE**
 594
**LAST PAGE**
 602
**DATE OF PUBLICATION**
 4 Mar 2015
**DOI**
 10.1097/CCM.0000000000000746

**RECORD 193**
**TITLE**
 **Procalcitonin levels in gram-positive, gram-negative, and fungal bloodstream infections**
**AUTHOR NAMES**
 Leli C.; Ferranti M.; Moretti A.; Al Dhahab Z.S.; Cenci E.; Mencacci A.
**SOURCE**
 Disease Markers (2015) 2015 Article Number: 701480. Date of Publication: 2015
**VOLUME**
 2015
**DATE OF PUBLICATION**
 2015
**DOI**
 10.1155/2015/701480

**RECORD 194**
**TITLE**
 **Clinical features and risk factors for blood stream infections of Candida in neonates**
**AUTHOR NAMES**
 Liu M.; Huang S.; Guo L.; Li H.; Wang F.; Zhang Q.; Song G.
**SOURCE**
 Experimental and Therapeutic Medicine (2015) 10:3 (1139-1144). Date of Publication: 1 Sep 2015
**VOLUME**
 10
**ISSUE**
 3
**FIRST PAGE**
 1139
**LAST PAGE**
 1144
**DATE OF PUBLICATION**
 1 Sep 2015
**DOI**
 10.3892/etm.2015.2626

**RECORD 195**
**TITLE**
 **Clinical diagnosis of sepsis and the combined use of biomarkers and culture- and non-culture-based assays**
**AUTHOR NAMES**
 Bloos F.
**SOURCE**
 Methods in Molecular Biology (2015) 1237 (247-260). Date of Publication: 2015
**VOLUME**
 1237
**FIRST PAGE**
 247
**LAST PAGE**
 260
**DATE OF PUBLICATION**
 2015
**DOI**
 10.1007/978-1-4939-1776-1_19

**RECORD 196**
**TITLE**
 **Nosography of systemic inflammatory response syndrome, sepsis, severe sepsis, septic shock, and multiple organ dysfunction syndrome in internal medicine patients**
**AUTHOR NAMES**
 Spoto S.; Valeriani E.; Costantino S.
**SOURCE**
 Italian Journal of Medicine (2015) 9:3 (243-251). Date of Publication: 2015
**VOLUME**
 9
**ISSUE**
 3
**FIRST PAGE**
 243
**LAST PAGE**
 251
**DATE OF PUBLICATION**
 2015
**DOI**
 10.4081/itjm.2015.606

**RECORD 197**
**TITLE**
 **Role of biomarkers as predictors of infection and death in neutropenic febrile patients after hematopoietic stem cell transplantation**
**AUTHOR NAMES**
 Massaro K.; Costa S.F.
**SOURCE**
 Mediterranean Journal of Hematology and Infectious Diseases (2015) 7:1 Article Number: e2015059. Date of Publication: 2015
**VOLUME**
 7
**ISSUE**
 1
**DATE OF PUBLICATION**
 2015
**DOI**
 10.4084/MJHID.2015.059

**RECORD 198**
**TITLE**
 **The role of biomarkers for starting antifungals in the intensive care unit**
**AUTHOR NAMES**
 Pagani J.-L.; Revelly J.-P.; Que Y.-A.; Eggimann P.
**SOURCE**
 Clinical Pulmonary Medicine (2015) 22:6 (286-293). Date of Publication: 2015
**VOLUME**
 22
**ISSUE**
 6
**FIRST PAGE**
 286
**LAST PAGE**
 293
**DATE OF PUBLICATION**
 2015
**DOI**
 10.1097/CPM.0000000000000118

**RECORD 199**
**TITLE**
 **Disseminated Fusarium infection in autologous stem cell transplant recipient**
**AUTHOR NAMES**
 Avelino-Silva V.I.; Ramos J.F.; Leal F.E.; Testagrossa L.; Novis Y.S.
**SOURCE**
 Brazilian Journal of Infectious Diseases (2015) 19:1 (90-93). Date of Publication: 1 Jan 2015
**VOLUME**
 19
**ISSUE**
 1
**FIRST PAGE**
 90
**LAST PAGE**
 93
**DATE OF PUBLICATION**
 1 Jan 2015
**DOI**
 10.1016/j.bjid.2014.08.009

**RECORD 200**
**TITLE**
 **Impact of COPD in the outcome of ICU - Acquired pneumonia with and without previous intubation**
**AUTHOR NAMES**
 Rinaudo M.; Ferrer M.; Terraneo S.; De Rosa F.; Peralta R.; Fernández-Barat L.; Li Bassi G.; Torres A.
**SOURCE**
 Chest (2015) 147:6 (1530-1538). Date of Publication: 1 Jun 2015
**VOLUME**
 147
**ISSUE**
 6
**FIRST PAGE**
 1530
**LAST PAGE**
 1538
**DATE OF PUBLICATION**
 1 Jun 2015
**DOI**
 10.1378/chest.14-2005

**RECORD 201**
**TITLE**
 **Procalcitonin measurements in coccidioidomycosis**
**AUTHOR NAMES**
 Chahal R.; Nesbit L.; Strawter C.; Oren E.; Song K.; Knox K.
**SOURCE**
 Journal of Investigative Medicine (2015) 63:1 (95). Date of Publication: January 2015
**VOLUME**
 63
**ISSUE**
 1
**FIRST PAGE**
 95
**DATE OF PUBLICATION**
 January 2015
**DOI**
 10.1097/JIM.0000000000000133

**RECORD 202**
**TITLE**
 **Clinical evaluation of multiple inflammation biomarkers for diagnosis and prognosis for patients with systemic inflammatory response syndrome**
**AUTHOR NAMES**
 Reichsoellner M.; Raggam R.B.; Wagner J.; Krause R.; Hoenigl M.
**SOURCE**
 Journal of Clinical Microbiology (2014) 52:11 (4063-4066). Date of Publication: 1 Nov 2014
**VOLUME**
 52
**ISSUE**
 11
**FIRST PAGE**
 4063
**LAST PAGE**
 4066
**DATE OF PUBLICATION**
 1 Nov 2014
**DOI**
 10.1128/JCM.01954-14

**RECORD 203**
**TITLE**
 **Critically ill patient - a permanent challenge**
**AUTHOR NAMES**
 Iovănescu D.; Roşculet C.; Rogoz A.; Radu M.; Apostolescu C.; Zlotea R.; Manu B.; Tănase N.
**SOURCE**
 BMC Infectious Diseases (2014) 14 Supplement 7 Article Number: P91. Date of Publication: 15 Oct 2014
**VOLUME**
 14
**DATE OF PUBLICATION**
 15 Oct 2014
**DOI**
 10.1186/1471-2334-14-S7-P91

**RECORD 204**
**TITLE**
 **Procalcitonin fails to predict bacteremia in SIRS patients: A cohort study**
**AUTHOR NAMES**
 Hoenigl M.; Raggam R.B.; Wagner J.; Prueller F.; Grisold A.J.; Leitner E.; Seeber K.; Prattes J.; Valentin T.; Zollner-Schwetz I.; Schilcher G.; Krause R.
**SOURCE**
 International Journal of Clinical Practice (2014) 68:10 (1278-1281). Date of Publication: 1 Oct 2014
**VOLUME**
 68
**ISSUE**
 10
**FIRST PAGE**
 1278
**LAST PAGE**
 1281
**DATE OF PUBLICATION**
 1 Oct 2014
**DOI**
 10.1111/ijcp.12474

**RECORD 205**
**TITLE**
 **Methods of rapid diagnosis for the etiology of meningitis in adults**
**AUTHOR NAMES**
 Bahr N.C.; Boulware D.R.
**SOURCE**
 Biomarkers in Medicine (2014) 8:9 (1085-1103). Date of Publication: 1 Oct 2014
**VOLUME**
 8
**ISSUE**
 9
**FIRST PAGE**
 1085
**LAST PAGE**
 1103
**DATE OF PUBLICATION**
 1 Oct 2014
**DOI**
 10.2217/BMM.14.67

**RECORD 206**
**TITLE**
 **Characteristics of hospital-acquired and community-onset blood stream infections, South-East Austria**
**AUTHOR NAMES**
 Hoenigl M.; Wagner J.; Raggam R.B.; Prueller F.; Prattes J.; Eigl S.; Leitner E.; Hönigl K.; Valentin T.; Zollner-Schwetz I.; Grisold A.J.; Krause R.
**SOURCE**
 PLoS ONE (2014) 9:8 Article Number: e104702. Date of Publication: 8 Aug 2014
**VOLUME**
 9
**ISSUE**
 8
**DATE OF PUBLICATION**
 8 Aug 2014
**DOI**
 10.1371/journal.pone.0104702

**RECORD 207**
**TITLE**
 **Elevated procalcitonin predicts Gram-negative sepsis in burn patients**
**AUTHOR NAMES**
 Khira J.; Bousselmi K.; Lamia T.
**SOURCE**
 Clinical Chemistry and Laboratory Medicine (2014) 52 SUPPL. 1 (S961). Date of Publication: July 2014
**VOLUME**
 52
**FIRST PAGE**
 S961
**DATE OF PUBLICATION**
 July 2014
**DOI**
 10.1515/cclm-2014-4031

**RECORD 208**
**TITLE**
 **Neonatal sepsis and its complications**
**AUTHOR NAMES**
 Paterson A.
**SOURCE**
 Pediatric Radiology (2014) 44 SUPPL. 2 (S279-S280). Date of Publication: June 2014
**VOLUME**
 44
**FIRST PAGE**
 S279
**LAST PAGE**
 S280
**DATE OF PUBLICATION**
 June 2014
**DOI**
 10.1007/s00247-014-2968-2

**RECORD 209**
**TITLE**
 **Septic markers in hemato-oncology: Procalcitonin is more specific than c-reactive protein in the study of fever in patients with hematologic disorders**
**AUTHOR NAMES**
 Pereira M.; Marques G.; Silva N.; Cortesão E.; Espadana A.; Geraldes C.; Ribeiro L.; Ribeiro G.
**SOURCE**
 Haematologica (2014) 99 SUPPL. 1 (462-463). Date of Publication: 1 Jun 2014
**VOLUME**
 99
**FIRST PAGE**
 462
**LAST PAGE**
 463
**DATE OF PUBLICATION**
 1 Jun 2014

**RECORD 210**
**TITLE**
 **Invasive pulmonary aspergillosis in a patient presenting with idiopathic systemic capillary leak syndrome**
**AUTHOR NAMES**
 Hayama M.; Shime N.; Mio T.
**SOURCE**
 BMJ Case Reports (2014). Date of Publication: 23 May 2014
**DATE OF PUBLICATION**
 23 May 2014
**DOI**
 10.1136/bcr-2014-203764

**RECORD 211**
**TITLE**
 **The potential diagnostic role of procalcitonin for bacteremia in a large cohort of solid cancer patients**
**AUTHOR NAMES**
 Fioroni I.; Vincenzi B.; Zoccoli A.; Angeletti S.; De Florio L.; Dicuonzo G.; Picardi A.; Caricato M.; Santini D.; Tonini G.
**SOURCE**
 Journal of Clinical Oncology (2014) 32:15 SUPPL. 1. Date of Publication: 20 May 2014
**VOLUME**
 32
**ISSUE**
 15
**DATE OF PUBLICATION**
 20 May 2014

**RECORD 212**
**TITLE**
 **Use of an oscillatory PEP device to enhance bronchial hygiene in a patient of post-H1NI pneumonia and acute respiratory distress syndrome with pneumothorax**
**AUTHOR NAMES**
 Narula D.; Nangia V.
**SOURCE**
 BMJ Case Reports (2014). Date of Publication: 7 Mar 2014
**DATE OF PUBLICATION**
 7 Mar 2014
**DOI**
 10.1136/bcr-2013-202598

**RECORD 213**
**TITLE**
 **Procalcitonin as a marker of Candida species detection by blood culture and polymerase chain reaction in septic patients**
**AUTHOR NAMES**
 Cortegiani A.; Russotto V.; Montalto F.; Foresta G.; Accurso G.; Palmeri C.; Raineri S.M.; Giarratano A.
**SOURCE**
 BMC Anesthesiology (2014) 14 Article Number: 9. Date of Publication: 21 Feb 2014
**VOLUME**
 14
**DATE OF PUBLICATION**
 21 Feb 2014
**DOI**
 10.1186/1471-2253-14-9

**RECORD 214**
**TITLE**
 **A case of granulomatosis with polyangiitis complicated by cyclophosphamide toxicity and opportunistic infections: Choosing between Scylla and Charybdis**
**AUTHOR NAMES**
 Ernst E.; Girndt M.; Pliquett R.U.
**SOURCE**
 BMC Nephrology (2014) 15:1 Article Number: 28. Date of Publication: 4 Feb 2014
**VOLUME**
 15
**ISSUE**
 1
**DATE OF PUBLICATION**
 4 Feb 2014
**DOI**
 10.1186/1471-2369-15-28

**RECORD 215**
**TITLE**
 **Three cases of invasive tracheobronchial aspergillosis**
**AUTHOR NAMES**
 Liu X.D.; Han X.D.; Qu Y.; Wei D.; Ge Y.J.; Zhao W.Y.
**SOURCE**
 Chinese Medical Journal (2014) 127:1 (196). Date of Publication: 2014
**VOLUME**
 127
**ISSUE**
 1
**FIRST PAGE**
 196
**DATE OF PUBLICATION**
 2014
**DOI**
 10.3760/cma.j.issn.0366-6999.20132266

**RECORD 216**
**TITLE**
 **Fungemias following liver or kidney transplantation: A clinical analysis of 17 patients**
**AUTHOR NAMES**
 Nie X.; Wan Q.; Ye Q.; Zhou J.
**SOURCE**
 Journal of Pure and Applied Microbiology (2014) 8:SPEC. ISS. 1 (667-670). Date of Publication: May 2014
**VOLUME**
 8
**ISSUE**
 SPEC. ISS. 1
**FIRST PAGE**
 667
**LAST PAGE**
 670
**DATE OF PUBLICATION**
 May 2014

**RECORD 217**
**TITLE**
 **Culture-proven neonatal sepsis in preterm infants in a neonatal intensive care unit over a 7 year period: Coagulase-negative Staphylococcus as the predominant pathogen**
**AUTHOR NAMES**
 Ozkan H.; Cetinkaya M.; Koksal N.; Celebi S.; Hacimustafaoglu M.
**SOURCE**
 Pediatrics International (2014) 56:1 (60-66). Date of Publication: February 2014
**VOLUME**
 56
**ISSUE**
 1
**FIRST PAGE**
 60
**LAST PAGE**
 66
**DATE OF PUBLICATION**
 February 2014
**DOI**
 10.1111/ped.12218

**RECORD 218**
**TITLE**
 **Evaluation of multiplex real time polymerase chain reaction and procalcitonin in the diagnosis of sepsis**
**AUTHOR NAMES**
 Ozkaya-Parlakay A.; Cengiz A.B.; Ceyhan M.; Hascelik G.; Kara A.; Celik M.; Ozsurekci Y.; Karadag-Oncel E.
**SOURCE**
 Clinical Laboratory (2014) 60:7 (1075-1081). Date of Publication: 2014
**VOLUME**
 60
**ISSUE**
 7
**FIRST PAGE**
 1075
**LAST PAGE**
 1081
**DATE OF PUBLICATION**
 2014
**DOI**
 10.7754/Clin.Lab.2013.130732

**RECORD 219**
**TITLE**
 **Critically ill patient - A permanent challenge**
**AUTHOR NAMES**
 IovǍnescu D.; Roşculeţ C.; Rogoz A.; Radu M.; Apostolescu C.; Zlotea R.; Manu B.; TǍnase N.
**SOURCE**
 BMC Infectious Diseases (2014) 14:7. Date of Publication: 2014
**VOLUME**
 14
**ISSUE**
 7
**DATE OF PUBLICATION**
 2014

**RECORD 220**
**TITLE**
 **Clinical manifestations of lead-dependent infective endocarditis: Analysis of 414 cases**
**AUTHOR NAMES**
 Polewczyk A.; Janion M.; Podlaski R.; Kutarski A.
**SOURCE**
 European Journal of Clinical Microbiology and Infectious Diseases (2014) 33:9 (1601-1608). Date of Publication: September 2014
**VOLUME**
 33
**ISSUE**
 9
**FIRST PAGE**
 1601
**LAST PAGE**
 1608
**DATE OF PUBLICATION**
 September 2014
**DOI**
 10.1007/s10096-014-2117-8

**RECORD 221**
**TITLE**
 **Clinical value of procalcitonin for patients with suspected bloodstream infection**
**AUTHOR NAMES**
 Hattori T.; Nishiyama H.; Kato H.; Ikegami S.; Nagayama M.; Asami S.; Usami M.; Suzuki M.; Murakami I.; Minoshima M.; Yamagishi H.; Yuasa N.
**SOURCE**
 American Journal of Clinical Pathology (2014) 141:1 (43-51). Date of Publication: January 2014
**VOLUME**
 141
**ISSUE**
 1
**FIRST PAGE**
 43
**LAST PAGE**
 51
**DATE OF PUBLICATION**
 January 2014
**DOI**
 10.1309/AJCP4GV7ZFDTANGC

**RECORD 222**
**TITLE**
 **Serum procalcitonin and high sensitivity C-reactive protein in distinguishing ADHF and CAP**
**AUTHOR NAMES**
 Abdel Aziz M.A.H.; Mohammed H.H.; Abou Zaid A.A.E.; Assal H.H.; Rashad R.A.
**SOURCE**
 Egyptian Journal of Chest Diseases and Tuberculosis (2014) 63:2 (455-462). Date of Publication: 2014
**VOLUME**
 63
**ISSUE**
 2
**FIRST PAGE**
 455
**LAST PAGE**
 462
**DATE OF PUBLICATION**
 2014
**DOI**
 10.1016/j.ejcdt.2014.02.002

**RECORD 223**
**TITLE**
 **Serum procalcitonin and high sensitivity C-reactive protein in distinguishing ADHF and CAP**
**AUTHOR NAMES**
 Abdel Aziz M.A.H.; Mohammed H.H.; Abou Zaid A.A.E.; Assal H.H.; Rashad R.A.
**SOURCE**
 Egyptian Journal of Chest Diseases and Tuberculosis (2014) 63:2 (455-462). Date of Publication: April 2014
**VOLUME**
 63
**ISSUE**
 2
**FIRST PAGE**
 455
**LAST PAGE**
 462
**DATE OF PUBLICATION**
 April 2014
**DOI**
 10.1016/j.ejcdt.2014.02.002

**RECORD 224**
**TITLE**
 **Neonatal sepsis an old problem with new insights**
**AUTHOR NAMES**
 Shah B.A.; Padbury J.F.
**SOURCE**
 Virulence (2014) 5:1 (163-171). Date of Publication: 2014
**VOLUME**
 5
**ISSUE**
 1
**FIRST PAGE**
 163
**LAST PAGE**
 171
**DATE OF PUBLICATION**
 2014
**DOI**
 10.4161/viru.26906

**RECORD 225**
**TITLE**
 **Blood stream infections**
**AUTHOR NAMES**
 Bharadwaj R.; Bal A.; Kapila K.; Mave V.; Gupta A.
**SOURCE**
 BioMed Research International (2014) 2014 Article Number: 515273. Date of Publication: 2014
**VOLUME**
 2014
**DATE OF PUBLICATION**
 2014
**DOI**
 10.1155/2014/515273

**RECORD 226**
**TITLE**
 **Validation of nosocomial infection in neonatology: A new method for standardized surveillance**
**AUTHOR NAMES**
 Durand S.; Batista Novais A.R.; Mesnage R.; Combes C.; Didelot M.-N.; Lotthé A.; Filleron A.; Baleine J.; Cambonie G.
**SOURCE**
 American Journal of Infection Control (2014) 42:8 (861-864). Date of Publication: August 2014
**VOLUME**
 42
**ISSUE**
 8
**FIRST PAGE**
 861
**LAST PAGE**
 864
**DATE OF PUBLICATION**
 August 2014
**DOI**
 10.1016/j.ajic.2014.04.021

**RECORD 227**
**TITLE**
 **Infection in late preterm infants**
**AUTHOR NAMES**
 Picone S.; Aufieri R.; Paolillo P.
**SOURCE**
 Early Human Development (2014) 90:SUPPL.1 (S71-S74). Date of Publication: March 2014
**VOLUME**
 90
**ISSUE**
 SUPPL.1
**DATE OF PUBLICATION**
 March 2014
**DOI**
 10.1016/S0378-3782(14)70022-2

**RECORD 228**
**TITLE**
 **Scedosporium apiospermum fungaemia: The ramification of broad-spectrum antimicrobial treatments**
**AUTHOR NAMES**
 Ding C.H.; Muttaqillah N.A.S.; Rahman M.M.; Abidin N.Z.Z.; Biswas S.; Tzar M.N
**SOURCE**
 Bangladesh Journal of Medical Science (2014) 13:3 (326-328). Date of Publication: 2014
**VOLUME**
 13
**ISSUE**
 3
**FIRST PAGE**
 326
**LAST PAGE**
 328
**DATE OF PUBLICATION**
 2014
**DOI**
 10.3329/bjms.v13i3.19154

**RECORD 229**
**TITLE**
 **Saccharomyces Cerevisiae Fungemia, a Possible Consequence of the Treatment of Clostridium Difficile Colitis with a Probioticum**
**AUTHOR NAMES**
 Santino I.; Alari A.; Bono S.; Teti E.; Marangi M.; Bernardini A.; Magrini L.; Di Somma S.; Teggi A.
**SOURCE**
 International Journal of Immunopathology and Pharmacology (2014) 27:1 (143-146). Date of Publication: 2014
**VOLUME**
 27
**ISSUE**
 1
**FIRST PAGE**
 143
**LAST PAGE**
 146
**DATE OF PUBLICATION**
 2014
**DOI**
 10.1177/039463201402700120

**RECORD 230**
**TITLE**
 **Saccharomyces cerevisiae fungemia, a possible consequence of the treatment of Clostridium difficile colitis with a probioticum**
**AUTHOR NAMES**
 Santino I.; Alari A.; Bono S.; Teti E.; Marangi M.; Bernardini A.; Magrini L.; Di Somma S.; Teggi A.
**SOURCE**
 International Journal of Immunopathology and Pharmacology (2014) 27:1 (143-146). Date of Publication: January-March 2014
**VOLUME**
 27
**ISSUE**
 1
**FIRST PAGE**
 143
**LAST PAGE**
 146
**DATE OF PUBLICATION**
 January-March 2014

**RECORD 231**
**TITLE**
 **Acute respiratory distress caused by Neosartorya udagawae**
**AUTHOR NAMES**
 Farrell J.J.; Kasper D.J.; Taneja D.; Baman S.; Orourke L.M.; Lowery K.S.; Sampath R.; Bonomo R.A.; Peterson S.W.
**SOURCE**
 Medical Mycology Case Reports (2014) 6:1 (1-5). Date of Publication: October/December 2014
**VOLUME**
 6
**ISSUE**
 1
**FIRST PAGE**
 1
**LAST PAGE**
 5
**DATE OF PUBLICATION**
 October/December 2014
**DOI**
 10.1016/j.mmcr.2014.07.003

**RECORD 232**
**TITLE**
 **Mechanisms of infective endocarditis: Pathogen-host interaction and risk states**
**AUTHOR NAMES**
 Werdan K.; Dietz S.; Löffler B.; Niemann S.; Bushnaq H.; Silber R.-E.; Peters G.; Müller-Werdan U.
**SOURCE**
 Nature Reviews Cardiology (2014) 11:1 (35-50). Date of Publication: January 2014
**VOLUME**
 11
**ISSUE**
 1
**FIRST PAGE**
 35
**LAST PAGE**
 50
**DATE OF PUBLICATION**
 January 2014
**DOI**
 10.1038/nrcardio.2013.174

**RECORD 233**
**TITLE**
 **Serum and bal beta-d-glucan for the diagnosis of Pneumocystis pneumonia in HIV positive patients**
**AUTHOR NAMES**
 Salerno D.; Mushatt D.; Myers L.; Zhuang Y.; De La Rua N.; Calderon E.J.; Welsh D.A.
**SOURCE**
 Respiratory Medicine (2014) 108:11 (1688-1695). Date of Publication: 1 Nov 2014
**VOLUME**
 108
**ISSUE**
 11
**FIRST PAGE**
 1688
**LAST PAGE**
 1695
**DATE OF PUBLICATION**
 1 Nov 2014
**DOI**
 10.1016/j.rmed.2014.09.017

**RECORD 234**
**TITLE**
 **Is blood culture a reliable guide to diagnosing catheter-related bacteremia (CRB)? A case of CRB caused by fungi and seven types of bacteria in a hemodialysis patient**
**AUTHOR NAMES**
 Wang Z.; Yang J.; Tang X.; Pi X.; Li L.; Yu J.
**SOURCE**
 International Urology and Nephrology (2014) 46:12 (2421-2422). Date of Publication: 2014
**VOLUME**
 46
**ISSUE**
 12
**FIRST PAGE**
 2421
**LAST PAGE**
 2422
**DATE OF PUBLICATION**
 2014
**DOI**
 10.1007/s11255-014-0760-8

**RECORD 235**
**TITLE**
 **New diagnostic possibilities in systemic neonatal infections: Metabolomics**
**AUTHOR NAMES**
 Dessì A.; Corsello G.; Stronati M.; Gazzolo D.; Caboni P.; Carboni R.; Fanos V.
**SOURCE**
 Early Human Development (2014) 90:SUPPL.1 (S19-S21). Date of Publication: March 2014
**VOLUME**
 90
**ISSUE**
 SUPPL.1
**DATE OF PUBLICATION**
 March 2014
**DOI**
 10.1016/S0378-3782(14)70007-6

**RECORD 236**
**TITLE**
 **Update on infections and vaccinations in systemic lupus erythematosus and Sjögren's syndrome**
**AUTHOR NAMES**
 Pasoto S.G.; Ribeiro A.C.M.; Bonfa E.
**SOURCE**
 Current Opinion in Rheumatology (2014) 26:5 (528-537). Date of Publication: September 2014
**VOLUME**
 26
**ISSUE**
 5
**FIRST PAGE**
 528
**LAST PAGE**
 537
**DATE OF PUBLICATION**
 September 2014
**DOI**
 10.1097/BOR.0000000000000084

**RECORD 237**
**TITLE**
 **Pneumonia after kidney transplant: Incidence, risk factors, and mortality**
**AUTHOR NAMES**
 Dizdar O.S.; Ersoy A.; Akalin H.
**SOURCE**
 Experimental and Clinical Transplantation (2014) 12:3 (205-211). Date of Publication: June 2014
**VOLUME**
 12
**ISSUE**
 3
**FIRST PAGE**
 205
**LAST PAGE**
 211
**DATE OF PUBLICATION**
 June 2014
**DOI**
 10.6002/ect.2013.0093

**RECORD 238**
**TITLE**
 **Effects of procalcitonin on antibiotic use at an academic medical center**
**AUTHOR NAMES**
 Darling C.; Faulkner-Fennell C.
**SOURCE**
 Critical Care Medicine (2013) 41:12 SUPPL. 1 (A257-A258). Date of Publication: December 2013
**VOLUME**
 41
**ISSUE**
 12
**FIRST PAGE**
 A257
**LAST PAGE**
 A258
**DATE OF PUBLICATION**
 December 2013
**DOI**
 10.1097/01.ccm.0000440261.09268.2b

**RECORD 239**
**TITLE**
 **Endocarditis due to a co-infection of Candida albicans and Candida tropicalis in a drug abuser**
**AUTHOR NAMES**
 Fesharaki S.H.; Haghani I.; Mousavi B.; Kargar M.L.; Boroumand M.; Anvari M.S.; Abbasi K.; Meis J.F.; Badali H.
**SOURCE**
 Journal of Medical Microbiology (2013) 62:PART 11 (1763-1767). Date of Publication: November 2013
**VOLUME**
 62
**ISSUE**
 PART 11
**FIRST PAGE**
 1763
**LAST PAGE**
 1767
**DATE OF PUBLICATION**
 November 2013
**DOI**
 10.1099/jmm.0.060954-0

**RECORD 240**
**TITLE**
 **A complex study of invasive fungal infections in a Hungarian University Hospital**
**AUTHOR NAMES**
 Dóczi Csányi I.; Bereczki L.; Sóki J.; Venkei A.; Urbán E.; Molnár Z.; Hankovszky P.
**SOURCE**
 Mycoses (2013) 56 SUPPL. 3 (107). Date of Publication: October 2013
**VOLUME**
 56
**FIRST PAGE**
 107
**DATE OF PUBLICATION**
 October 2013
**DOI**
 10.1111/myc.12124

**RECORD 241**
**TITLE**
 **A chronic eosinophilic pneumonia case with pleural involvement**
**AUTHOR NAMES**
 Bekci T.; Bakdik S.; Yavsan M.
**SOURCE**
 Chest (2013) 144:4 MEETING ABSTRACT. Date of Publication: October 2013
**VOLUME**
 144
**ISSUE**
 4
**DATE OF PUBLICATION**
 October 2013
**DOI**
 10.1378/chest.1702121

**RECORD 242**
**TITLE**
 **Treatment of severe human metapneumovirus (hmpv) pneumonia in an immunocompromised child with oral ribavirin and ivig**
**AUTHOR NAMES**
 Kitanovski L.; Kopriva S.; Pokorn M.; Dolničar M.B.; Rajić V.; Stefanović M.; Jazbec J.
**SOURCE**
 Journal of Pediatric Hematology/Oncology (2013) 35:7 (e311-e313). Date of Publication: October 2013
**VOLUME**
 35
**ISSUE**
 7
**DATE OF PUBLICATION**
 October 2013
**DOI**
 10.1097/MPH.0b013e3182915d2d

**RECORD 243**
**TITLE**
 **Procalcitonin (PCT) and C-reactive protein (CRP) as markers of the differential diagnosis of severe community acquired pneumonia (sCAP)**
**AUTHOR NAMES**
 Bielosludtsev O.; Bielosludtseva K.; Nazarenko O.
**SOURCE**
 European Respiratory Journal (2013) 42 SUPPL. 57. Date of Publication: 1 Sep 2013
**VOLUME**
 42
**DATE OF PUBLICATION**
 1 Sep 2013

**RECORD 244**
**TITLE**
 **ICU-acquired pneumonia with or without etiologic diagnosis: A comparison of outcomes**
**AUTHOR NAMES**
 Giunta V.; Ferrer M.; Esperatti M.; Ranzani O.T.; Saucedo L.M.; Bassi G.L.; Blasi F.; Torres A.
**SOURCE**
 Critical Care Medicine (2013) 41:9 (2133-2143). Date of Publication: September 2013
**VOLUME**
 41
**ISSUE**
 9
**FIRST PAGE**
 2133
**LAST PAGE**
 2143
**DATE OF PUBLICATION**
 September 2013
**DOI**
 10.1097/CCM.0b013e31828a453b

**RECORD 245**
**TITLE**
 **The role of procalcitonin in the identification of invasive fungal infection-a systemic review and meta-analysis**
**AUTHOR NAMES**
 Dou Y.-H.; Du J.-K.; Liu H.-L.; Shong X.-D.
**SOURCE**
 Diagnostic Microbiology and Infectious Disease (2013) 76:4 (464-469). Date of Publication: August 2013
**VOLUME**
 76
**ISSUE**
 4
**FIRST PAGE**
 464
**LAST PAGE**
 469
**DATE OF PUBLICATION**
 August 2013
**DOI**
 10.1016/j.diagmicrobio.2013.04.023

**RECORD 246**
**TITLE**
 **Significantly higher procalcitonin levels could differentiate Gram-negative sepsis from Gram-positive and fungal sepsis**
**AUTHOR NAMES**
 Brodská H.; Malíčková K.; Adámková V.; Benáková H.; Šåastná M.M.; Zima T.
**SOURCE**
 Clinical and Experimental Medicine (2013) 13:3 (165-170). Date of Publication: August 2013
**VOLUME**
 13
**ISSUE**
 3
**FIRST PAGE**
 165
**LAST PAGE**
 170
**DATE OF PUBLICATION**
 August 2013
**DOI**
 10.1007/s10238-012-0191-8

**RECORD 247**
**TITLE**
 **Épico project. Development of educational recommendations using the DELPHI technique on invasive candidiasis in non-neutropenic critically ill adult patients**
**AUTHOR NAMES**
 Zaragoza R.; Llinares P.; Maseda E.; Ferrer R.; Rodríguez A.
**SOURCE**
 Revista Iberoamericana de Micologia (2013) 30:3 (135-149). Date of Publication: July 2013
**VOLUME**
 30
**ISSUE**
 3
**FIRST PAGE**
 135
**LAST PAGE**
 149
**DATE OF PUBLICATION**
 July 2013
**DOI**
 10.1016/j.riam.2013.05.006

**RECORD 248**
**TITLE**
 **Can an etiologic agent be identified in adults who are hospitalized for community-acquired pneumonia: Results of a one-year study**
**AUTHOR NAMES**
 Musher D.M.; Roig I.L.; Cazares G.; Stager C.E.; Logan N.; Safar H.
**SOURCE**
 Journal of Infection (2013) 67:1 (11-18). Date of Publication: July 2013
**VOLUME**
 67
**ISSUE**
 1
**FIRST PAGE**
 11
**LAST PAGE**
 18
**DATE OF PUBLICATION**
 July 2013
**DOI**
 10.1016/j.jinf.2013.03.003

**RECORD 249**
**TITLE**
 **A severe case of haemodynamic instability during anidulafungin administration**
**AUTHOR NAMES**
 Fink M.; Zerlauth U.; Kaulfersch C.; Rab A.; Alberer D.; Preiss P.; Sternad-Klobschauer K.; Habernig E.; Wandschneider W.; Grimm G.
**SOURCE**
 Journal of Clinical Pharmacy and Therapeutics (2013) 38:3 (241-242). Date of Publication: June 2013
**VOLUME**
 38
**ISSUE**
 3
**FIRST PAGE**
 241
**LAST PAGE**
 242
**DATE OF PUBLICATION**
 June 2013
**DOI**
 10.1111/jcpt.12046

**RECORD 250**
**TITLE**
 **Procalcitonin level associated with bacteriemia etiology in severe sepsis and septic shock**
**AUTHOR NAMES**
 Enguix A.; Garcia-De-La-Torre A.; De La Torre-Prados M.; Segovia M.J.; Escobar R.
**SOURCE**
 Biochimica Clinica (2013) 37 SUPPL. 1 (S136). Date of Publication: 2013
**VOLUME**
 37
**FIRST PAGE**
 S136
**DATE OF PUBLICATION**
 2013

**RECORD 251**
**TITLE**
 **Procalcitonin as a biomarker of infectious diseases**
**AUTHOR NAMES**
 Lee H.
**SOURCE**
 Korean Journal of Internal Medicine (2013) 28:3 (285-291). Date of Publication: May 2013
**VOLUME**
 28
**ISSUE**
 3
**FIRST PAGE**
 285
**LAST PAGE**
 291
**DATE OF PUBLICATION**
 May 2013
**DOI**
 10.3904/kjim.2013.28.3.285

**RECORD 252**
**TITLE**
 **Procalcitonin and mid-regional pro-adrenomedullin test combination in sepsis diagnosis**
**AUTHOR NAMES**
 Angeletti S.; Battistoni F.; Fioravanti M.; Bernardini S.; Dicuonzo G.
**SOURCE**
 Clinical Chemistry and Laboratory Medicine (2013) 51:5 (1059-1067). Date of Publication: May 2013
**VOLUME**
 51
**ISSUE**
 5
**FIRST PAGE**
 1059
**LAST PAGE**
 1067
**DATE OF PUBLICATION**
 May 2013
**DOI**
 10.1515/cclm-2012-0595

**RECORD 253**
**TITLE**
 **Epidemiology and clinical characteristics of lower respiratory tract infections among kidney transplant recipients**
**AUTHOR NAMES**
 Akalin H.; Dizdar O.; Ersoy A.
**SOURCE**
 Nephrology Dialysis Transplantation (2013) 28 SUPPL. 1 (i505). Date of Publication: May 2013
**VOLUME**
 28
**FIRST PAGE**
 i505
**DATE OF PUBLICATION**
 May 2013
**DOI**
 10.1093/ndt/gft155

**RECORD 254**
**TITLE**
 **Comparison of (1→3)-β-d-glucan, mannan/anti-mannan antibodies, and cand-tec candida antigen as serum biomarkers for candidemia**
**AUTHOR NAMES**
 Held J.; Kohlberger I.; Rappold E.; Grawitz A.B.; Häckera G.
**SOURCE**
 Journal of Clinical Microbiology (2013) 51:4 (1158-1164). Date of Publication: April 2013
**VOLUME**
 51
**ISSUE**
 4
**FIRST PAGE**
 1158
**LAST PAGE**
 1164
**DATE OF PUBLICATION**
 April 2013
**DOI**
 10.1128/JCM.02473-12

**RECORD 255**
**TITLE**
 **Sepsis in AIDS patients: Clinical, etiological and inflammatory characteristics**
**AUTHOR NAMES**
 Silva Jr. J.M.; De Sousa Dos Santos S.
**SOURCE**
 Journal of the International AIDS Society (2013) 16 Article Number: 17344. Date of Publication: 30 Jan 2013
**VOLUME**
 16
**DATE OF PUBLICATION**
 30 Jan 2013
**DOI**
 10.7448/IAS.16.1.17344

**RECORD 256**
**TITLE**
 **Epidemiology and new developments in the diagnosis of prosthetic joint infection**
**AUTHOR NAMES**
 Corvec S.; Portillo M.E.; Pasticci B.M.; Borens O.; Trampuz A.
**SOURCE**
 International Journal of Artificial Organs (2012) 35:10 (923-934). Date of Publication: 2012
**VOLUME**
 35
**ISSUE**
 10
**FIRST PAGE**
 923
**LAST PAGE**
 934
**DATE OF PUBLICATION**
 2012
**DOI**
 10.5301/ijao.5000168

**RECORD 257**
**TITLE**
 **Neonatal sepsis: A continuing disease burden**
**AUTHOR NAMES**
 Satar M.; Özlü F.
**SOURCE**
 Turkish Journal of Pediatrics (2012) 54:5 (449-457). Date of Publication: 2012
**VOLUME**
 54
**ISSUE**
 5
**FIRST PAGE**
 449
**LAST PAGE**
 457
**DATE OF PUBLICATION**
 2012

**RECORD 258**
**TITLE**
 **Biomarkers in infectious diseases**
**AUTHOR NAMES**
 Downes K.J.; Shah S.S.
**SOURCE**
 Journal of the Pediatric Infectious Diseases Society (2012) 1:4 (343-346) Article Number: pis099. Date of Publication: 2012
**VOLUME**
 1
**ISSUE**
 4
**FIRST PAGE**
 343
**LAST PAGE**
 346
**DATE OF PUBLICATION**
 2012
**DOI**
 10.1093/jpids/pis099

**RECORD 259**
**TITLE**
 **Atrioesophageal fistula after cryoballoon pulmonary vein isolation**
**AUTHOR NAMES**
 Stöckigt F.; Schrickel J.W.; Andrié R.; Lickfett L.
**SOURCE**
 Journal of Cardiovascular Electrophysiology (2012) 23:11 (1254-1257). Date of Publication: November 2012
**VOLUME**
 23
**ISSUE**
 11
**FIRST PAGE**
 1254
**LAST PAGE**
 1257
**DATE OF PUBLICATION**
 November 2012
**DOI**
 10.1111/j.1540-8167.2012.02324.x

**RECORD 260**
**TITLE**
 **Evaluation of procalcitonin levels in umbilical cord blood for diagnosis of chorioamnionitis and its correlation of neonatal sepsis**
**AUTHOR NAMES**
 Kaneko M.; Sameshima H.; Kodama Y.; Ikenoue T.
**SOURCE**
 International Journal of Gynecology and Obstetrics (2012) 119 SUPPL. 3 (S753). Date of Publication: October 2012
**VOLUME**
 119
**FIRST PAGE**
 S753
**DATE OF PUBLICATION**
 October 2012
**DOI**
 10.1016/S0020-7292(12)61872-4

**RECORD 261**
**TITLE**
 **Bacterial and invasive fungal infections and clostridium difficile diarrhea in a patient with multiple myeloma**
**AUTHOR NAMES**
 Molagic V.; Hristea A.; Popescu C.; Moroti R.; Niculescu I.; Rədulescu M.; Tiliscan C.; Nəstase R.; Dulama R.; Munteanu D.; Mihailescu R.; Ion M.; Gliga S.; Olaru I.; Petre R.; Tenita A.; Cristea D.; Chiotan M.; Borcan A.; Rafila A.; Dorobət¸f O.; Tələpan D.; Aramə V.
**SOURCE**
 Journal of Gastrointestinal and Liver Diseases (2012) 21 SUPPL. 4 (42). Date of Publication: October 2012
**VOLUME**
 21
**FIRST PAGE**
 42
**DATE OF PUBLICATION**
 October 2012

**RECORD 262**
**TITLE**
 **Disseminated associated with tsunami lung**
**AUTHOR NAMES**
 Kawakami Y.; Tagami T.; Kusakabe T.; Kido N.; Kawaguchi T.; Omura M.; Tosa R.
**SOURCE**
 Respiratory Care (2012) 57:10 (1674-1678). Date of Publication: October 2012
**VOLUME**
 57
**ISSUE**
 10
**FIRST PAGE**
 1674
**LAST PAGE**
 1678
**DATE OF PUBLICATION**
 October 2012
**DOI**
 10.4187/respcare.01701

**RECORD 263**
**TITLE**
 **Evaluation of a Polymerase Chain Reaction Assay for Pathogen Detection in Septic Patients under Routine Condition: An Observational Study**
**AUTHOR NAMES**
 Bloos F.; Sachse S.; Kortgen A.; Pletz M.W.; Lehmann M.; Straube E.; Riedemann N.C.; Reinhart K.; Bauer M.
**SOURCE**
 PLoS ONE (2012) 7:9 Article Number: e46003. Date of Publication: 27 Sep 2012
**VOLUME**
 7
**ISSUE**
 9
**DATE OF PUBLICATION**
 27 Sep 2012
**DOI**
 10.1371/journal.pone.0046003

**RECORD 264**
**TITLE**
 **Value of β-D-glucan and Candida albicans germ tube antibody for discriminating between Candida colonization and invasive candidiasis in patients with severe abdominal conditions**
**AUTHOR NAMES**
 León C.; Ruiz-Santana S.; Saavedra P.; Castro C.; Úbeda A.; Loza A.; Martín-Mazuelos E.; Blanco A.; Jerez V.; Ballús J.; Álvarez-Rocha L.; Utande-Vázquez A.; Fariñas O.
**SOURCE**
 Intensive Care Medicine (2012) 38:8 (1315-1325). Date of Publication: August 2012
**VOLUME**
 38
**ISSUE**
 8
**FIRST PAGE**
 1315
**LAST PAGE**
 1325
**DATE OF PUBLICATION**
 August 2012
**DOI**
 10.1007/s00134-012-2616-y

**RECORD 265**
**TITLE**
 **Fungaemia caused by Candida pulcherrima**
**AUTHOR NAMES**
 Bereczki L.; Bartha N.; Kocsubé S.; Sóki J.; Lengyel G.; Tálosi G.; Máder K.; Deák J.; Dóczi I.
**SOURCE**
 Medical Mycology (2012) 50:5 (522-524). Date of Publication: July 2012
**VOLUME**
 50
**ISSUE**
 5
**FIRST PAGE**
 522
**LAST PAGE**
 524
**DATE OF PUBLICATION**
 July 2012
**DOI**
 10.3109/13693786.2011.644590

**RECORD 266**
**TITLE**
 **Role of biomarkers in sepsis**
**AUTHOR NAMES**
 Tai D.Y.H.
**SOURCE**
 Critical Care and Shock (2012) 15:3 (60-62). Date of Publication: 2012
**VOLUME**
 15
**ISSUE**
 3
**FIRST PAGE**
 60
**LAST PAGE**
 62
**DATE OF PUBLICATION**
 2012

**RECORD 267**
**TITLE**
 **Low procalcitonin and high anti Candida IgG and IgM levels in non-neutropenic patients with candidemia**
**AUTHOR NAMES**
 Kappe R.; Gorges A.; Rimek D.
**SOURCE**
 Mycoses (2012) 55 SUPPL.4 (270). Date of Publication: June 2012
**VOLUME**
 55
**FIRST PAGE**
 270
**DATE OF PUBLICATION**
 June 2012
**DOI**
 10.1111/j.1439-0507.2012.02206.x

**RECORD 268**
**TITLE**
 **Diagnosis of infection in patients undergoing extracorporeal membrane oxygenation: A case-control study**
**AUTHOR NAMES**
 Pieri M.; Greco T.; De Bonis M.; Maj G.; Fumagalli L.; Zangrillo A.; Pappalardo F.
**SOURCE**
 Journal of Thoracic and Cardiovascular Surgery (2012) 143:6 (1411-1416). Date of Publication: June 2012
**VOLUME**
 143
**ISSUE**
 6
**FIRST PAGE**
 1411
**LAST PAGE**
 1416
**DATE OF PUBLICATION**
 June 2012
**DOI**
 10.1016/j.jtcvs.2012.01.005

**RECORD 269**
**TITLE**
 **Severe pneumonia in intensive care: Cause, diagnosis, treatment and management: A review of the literature**
**AUTHOR NAMES**
 De Pascale G.; Bello G.; Tumbarello M.; Antonelli M.
**SOURCE**
 Current Opinion in Pulmonary Medicine (2012) 18:3 (213-221+282-283). Date of Publication: May 2012
**VOLUME**
 18
**ISSUE**
 3
**DATE OF PUBLICATION**
 May 2012
**DOI**
 10.1097/MCP.0b013e328351f9bd

**RECORD 270**
**TITLE**
 **Evaluation and management of urinary tract infections in the neonate**
**AUTHOR NAMES**
 Beetz R.
**SOURCE**
 Current Opinion in Pediatrics (2012) 24:2 (205-211+282). Date of Publication: April 2012
**VOLUME**
 24
**ISSUE**
 2
**DATE OF PUBLICATION**
 April 2012
**DOI**
 10.1097/MOP.0b013e32834f0423

**RECORD 271**
**TITLE**
 **Serum procalcitonin increase in earthquake victims associated with sepsis**
**AUTHOR NAMES**
 Guo L.; Xie Y.; Xiong M.; Lv X.; Fan H.; Kang M.; Tao C.; Chen Z.
**SOURCE**
 Clinical Microbiology and Infection (2012) 18 SUPPL. 3 (783). Date of Publication: April 2012
**VOLUME**
 18
**FIRST PAGE**
 783
**DATE OF PUBLICATION**
 April 2012
**DOI**
 10.1111/j.1469-0691.2012.03802.x

**RECORD 272**
**TITLE**
 **Specimen collection for the diagnosis of pediatric pneumonia**
**AUTHOR NAMES**
 Hammitt L.L.; Murdoch D.R.; Scott J.A.G.; Driscoll A.; Karron R.A.; Levine O.S.; O'Brien K.L.
**SOURCE**
 Clinical Infectious Diseases (2012) 54:SUPPL. 2 (S132-S139). Date of Publication: 1 Apr 2012
**VOLUME**
 54
**ISSUE**
 SUPPL. 2
**DATE OF PUBLICATION**
 1 Apr 2012
**DOI**
 10.1093/cid/cir1068

**RECORD 273**
**TITLE**
 **Procalcitonin as a predictive marker for PCR test and blood culture results in suspected invasive candidemia**
**AUTHOR NAMES**
 Cortegiani A.; Raineri S.M.; Montalto F.; Strano M.T.; Giarratano A.
**SOURCE**
 Critical Care (2012) 16 SUPPL. 1 (S10-S11). Date of Publication: 20 Mar 2012
**VOLUME**
 16
**FIRST PAGE**
 S10
**LAST PAGE**
 S11
**DATE OF PUBLICATION**
 20 Mar 2012
**DOI**
 10.1186/cc10636

**RECORD 274**
**TITLE**
 **Procalcitonin has a poor prognosis value in critically ill patients with candidemia**
**AUTHOR NAMES**
 Charles P.E.; Bruyère R.; Roche H.; Quenot J.P.; Prin S.; Pavon A.; Dalle F.
**SOURCE**
 Critical Care (2012) 16 SUPPL. 1 (S11). Date of Publication: 20 Mar 2012
**VOLUME**
 16
**FIRST PAGE**
 S11
**DATE OF PUBLICATION**
 20 Mar 2012
**DOI**
 10.1186/cc10638

**RECORD 275**
**TITLE**
 **Role of serum biomarkers in the diagnosis of infection in patients undergoing extracorporeal membrane oxygenation**
**AUTHOR NAMES**
 Pieri M.; Greco T.; Scandroglio A.M.; De Bonis M.; Maj G.; Fumagalli L.; Zangrillo A.; Pappalardo F.
**SOURCE**
 Critical Care (2012) 16 SUPPL. 1 (S9-S10). Date of Publication: 20 Mar 2012
**VOLUME**
 16
**FIRST PAGE**
 S9
**LAST PAGE**
 S10
**DATE OF PUBLICATION**
 20 Mar 2012
**DOI**
 10.1186/cc10633

**RECORD 276**
**TITLE**
 **Prone and ECMO - A contradiction per se?**
**AUTHOR NAMES**
 Litmathe J.; Sucker C.; Easo J.; Wigger L.; Dapunt O.
**SOURCE**
 Perfusion (2012) 27:1 (78-82). Date of Publication: January 2012
**VOLUME**
 27
**ISSUE**
 1
**FIRST PAGE**
 78
**LAST PAGE**
 82
**DATE OF PUBLICATION**
 January 2012
**DOI**
 10.1177/0267659111424640

**RECORD 277**
**TITLE**
 **Soluble triggering receptor expressed on myeloid cells in severe acute pancreatitis: A biological marker of infected necrosis**
**AUTHOR NAMES**
 Lu Z.; Liu Y.; Dong Y.-H.; Zhan X.-B.; Du Y.-Q.; Gao J.; Gong Y.-F.; Li Z.-S.
**SOURCE**
 Intensive Care Medicine (2012) 38:1 (69-75). Date of Publication: January 2012
**VOLUME**
 38
**ISSUE**
 1
**FIRST PAGE**
 69
**LAST PAGE**
 75
**DATE OF PUBLICATION**
 January 2012
**DOI**
 10.1007/s00134-011-2369-z

**RECORD 278**
**TITLE**
 **Blastomycosis in China: A case report and literature review**
**AUTHOR NAMES**
 Zhao T.-M.; Gao J.; She D.-Y.; Chen L.-A.
**SOURCE**
 Chinese Medical Journal (2011) 124:24 (4368-4371). Date of Publication: 20111220
**VOLUME**
 124
**ISSUE**
 24
**FIRST PAGE**
 4368
**LAST PAGE**
 4371
**DATE OF PUBLICATION**
 20111220
**DOI**
 10.3760/cma.j.issn.0366-6999.2011.24.044

**RECORD 279**
**TITLE**
 **Advances in the management of sepsis and the understanding of key immunologic defects**
**AUTHOR NAMES**
 Skrupky L.P.; Kerby P.W.; Hotchkiss R.S.
**SOURCE**
 Anesthesiology (2011) 115:6 (1349-1362). Date of Publication: December 2011
**VOLUME**
 115
**ISSUE**
 6
**FIRST PAGE**
 1349
**LAST PAGE**
 1362
**DATE OF PUBLICATION**
 December 2011
**DOI**
 10.1097/ALN.0b013e31823422e8

**RECORD 280**
**TITLE**
 **One case of Penicillium marneffei infection after kidney transplantation**
**AUTHOR NAMES**
 Chen T.-Q.; Kong Y.-Z.; Luo Q.-W.; Lin M.-W.; Xu J.-W.
**SOURCE**
 Chinese Journal of Tissue Engineering Research (2011) 15:53 (10068-10070). Date of Publication: December 2011
**VOLUME**
 15
**ISSUE**
 53
**FIRST PAGE**
 10068
**LAST PAGE**
 10070
**DATE OF PUBLICATION**
 December 2011
**DOI**
 10.3969/j.issn.1673-8225.2011.53.046

**RECORD 281**
**TITLE**
 **Rhodotorula fungemia: Two cases and a brief review**
**AUTHOR NAMES**
 Duggal S.; Jain H.; Tyagi A.; Sharma A.; Chugh T.D.
**SOURCE**
 Medical Mycology (2011) 49:8 (879-882). Date of Publication: November 2011
**VOLUME**
 49
**ISSUE**
 8
**FIRST PAGE**
 879
**LAST PAGE**
 882
**DATE OF PUBLICATION**
 November 2011
**DOI**
 10.3109/13693786.2011.583694

**RECORD 282**
**TITLE**
 **Procalcitonin serum concentration in lung transplant recipients during mold colonization or infection**
**AUTHOR NAMES**
 Zegleń S.; Sioła M.; Woźniak-Grygiel E.; Łaszewska A.; Sindera P.; Wojarski J.; Ochman M.; Kucewicz E.; Karolak W.; Szewczyk M.; Zembala M.
**SOURCE**
 Transplantation Proceedings (2011) 43:8 (3089-3091). Date of Publication: October 2011
**VOLUME**
 43
**ISSUE**
 8
**FIRST PAGE**
 3089
**LAST PAGE**
 3091
**DATE OF PUBLICATION**
 October 2011
**DOI**
 10.1016/j.transproceed.2011.08.057

**RECORD 283**
**TITLE**
 **Serum β-d-glucan of critically ill patients with suspected ventilator-associated pneumonia: Preliminary observations**
**AUTHOR NAMES**
 Heyland D.; Jiang X.; Day A.G.; Laverdiere M.
**SOURCE**
 Journal of Critical Care (2011) 26:5 (536.e1-536.e9). Date of Publication: October 2011
**VOLUME**
 26
**ISSUE**
 5
**DATE OF PUBLICATION**
 October 2011
**DOI**
 10.1016/j.jcrc.2011.01.002

**RECORD 284**
**TITLE**
 **New era "soluble triggering receptor expressed on myeloid cells-I" as a marker for early detection of infection in trauma patients**
**AUTHOR NAMES**
 Soud D.E.M.; Amin O.A.I.; Amin A.A.I.
**SOURCE**
 Egyptian Journal of Anaesthesia (2011) 27:4 (267-272). Date of Publication: October 2011
**VOLUME**
 27
**ISSUE**
 4
**FIRST PAGE**
 267
**LAST PAGE**
 272
**DATE OF PUBLICATION**
 October 2011
**DOI**
 10.1016/j.egja.2011.07.002

**RECORD 285**
**TITLE**
 **Too much of a good thing is not necessarily better**
**AUTHOR NAMES**
 Nielsen H.
**SOURCE**
 Critical Care Medicine (2011) 39:9 (2182-2183). Date of Publication: September 2011
**VOLUME**
 39
**ISSUE**
 9
**FIRST PAGE**
 2182
**LAST PAGE**
 2183
**DATE OF PUBLICATION**
 September 2011
**DOI**
 10.1097/CCM.0b013e3182207c13

**RECORD 286**
**TITLE**
 **Procalcitonin level associated with bacteriemia etiology in severe sepsis and septic shock**
**AUTHOR NAMES**
 De La Torre-Prados M.-V.; García-De La Torre A.; Nieto-Gonzalez M.; Zambochi N.; Trujillano C.; Garcia-Alcántara A.
**SOURCE**
 Intensive Care Medicine (2011) 37 SUPPL. 1 (S22). Date of Publication: September 2011
**VOLUME**
 37
**FIRST PAGE**
 S22
**DATE OF PUBLICATION**
 September 2011

**RECORD 287**
**TITLE**
 **The late phase of sepsis is characterized by an increased microbiological burden and death rate**
**AUTHOR NAMES**
 Otto G.P.; Sossdorf M.; Claus R.A.; Rödel J.; Menge K.; Reinhart K.; Bauer M.; Riedemann N.C.
**SOURCE**
 Critical Care (2011) 15:4 Article Number: R183. Date of Publication: 28 Jul 2011
**VOLUME**
 15
**ISSUE**
 4
**DATE OF PUBLICATION**
 28 Jul 2011
**DOI**
 10.1186/cc10332

**RECORD 288**
**TITLE**
 **Addressing unmet clinical needs in the early diagnosis of sepsis**
**AUTHOR NAMES**
 Schaub N.; Frei R.; Mueller C.
**SOURCE**
 Swiss Medical Weekly (2011) 141:JULY. Date of Publication: July 2011
**VOLUME**
 141
**ISSUE**
 JULY
**DATE OF PUBLICATION**
 July 2011
**DOI**
 10.4414/smw.2011.13244

**RECORD 289**
**TITLE**
 **Septifast and blood culture for identification of bloodstream pathogens in patients with cystic fibrosis during febrile infective exacerbation**
**AUTHOR NAMES**
 Grosse-Onnebrink J.; Steinmann J.; Stehling F.; Tschiedel E.; Olivier M.; Rath P.M.; Mellies U.
**SOURCE**
 Paediatric Respiratory Reviews (2011) 12 Supplement 1 (S84). Date of Publication: 1 Jun 2011
**VOLUME**
 12
**FIRST PAGE**
 S84
**DATE OF PUBLICATION**
 1 Jun 2011

**RECORD 290**
**TITLE**
 **Early diagnostic value of plasma PCT and BG assay for CRBSI after OLT**
**AUTHOR NAMES**
 Chen J.; Wang Y.; Shen Z.; Zhu Z.; Song Y.; Han R.
**SOURCE**
 Transplantation Proceedings (2011) 43:5 (1777-1779). Date of Publication: June 2011
**VOLUME**
 43
**ISSUE**
 5
**FIRST PAGE**
 1777
**LAST PAGE**
 1779
**DATE OF PUBLICATION**
 June 2011
**DOI**
 10.1016/j.transproceed.2010.11.026

**RECORD 291**
**TITLE**
 **Single procalcitonin determination is not a reliable predictor of infection in critically ill cardiac surgery patients with low cardiac output syndrome**
**AUTHOR NAMES**
 Rossi A.; Barili F.; Degiovanni C.; Barzaghi N.
**SOURCE**
 Journal of Cardiothoracic and Vascular Anesthesia (2011) 25:3 SUPPL. 1 (S6). Date of Publication: June 2011
**VOLUME**
 25
**ISSUE**
 3
**FIRST PAGE**
 S6
**DATE OF PUBLICATION**
 June 2011
**DOI**
 10.1053/j.jvca.2011.03.026

**RECORD 292**
**TITLE**
 **Significantly higher procalcitonin levels could differentiate Gram negative bacteremia from Gram positive and fungaemia**
**AUTHOR NAMES**
 Brodska H.; Malickova K.; Benakova H.; Adamkova V.; Markova M.; Zima T.
**SOURCE**
 Clinical Chemistry and Laboratory Medicine (2011) 49 SUPPL. 1 (S352). Date of Publication: May 2011
**VOLUME**
 49
**FIRST PAGE**
 S352
**DATE OF PUBLICATION**
 May 2011
**DOI**
 10.1515/CCLM.2011.509

**RECORD 293**
**TITLE**
 **Similar Levels of inflammatory markers in non-neutropenic patientswith candidemia and Bacteremia**
**AUTHOR NAMES**
 Gorges A.; Rimek D.; Kappe R.
**SOURCE**
 Clinical Chemistry and Laboratory Medicine (2011) 49 SUPPL. 1 (S525). Date of Publication: May 2011
**VOLUME**
 49
**FIRST PAGE**
 S525
**DATE OF PUBLICATION**
 May 2011
**DOI**
 10.1515/CCLM.2011.516

**RECORD 294**
**TITLE**
 **Antibiotic therapy in patients with septic shock**
**AUTHOR NAMES**
 Textoris J.; Wiramus S.; Martin C.; Leone M.
**SOURCE**
 European Journal of Anaesthesiology (2011) 28:5 (318-324). Date of Publication: May 2011
**VOLUME**
 28
**ISSUE**
 5
**FIRST PAGE**
 318
**LAST PAGE**
 324
**DATE OF PUBLICATION**
 May 2011
**DOI**
 10.1097/EJA.0b013e328346c0de

**RECORD 295**
**TITLE**
 **Procalcitonin: A possible marker of invasive fungal infection in high risk patients?**
**AUTHOR NAMES**
 Montagna M.T.; Coretti C.; Caggiano G.
**SOURCE**
 Journal of Preventive Medicine and Hygiene (2011) 52:1 (38-39). Date of Publication: March 2011
**VOLUME**
 52
**ISSUE**
 1
**FIRST PAGE**
 38
**LAST PAGE**
 39
**DATE OF PUBLICATION**
 March 2011

**RECORD 296**
**TITLE**
 **The relationship between Candida species cultured from the respiratory tract and systemic inflammation in critically ill patients with ventilator-associated pneumonia**
**AUTHOR NAMES**
 Williamson D.R.; Albert M.; Perreault M.M.; Delisle M.-S.; Muscedere J.; Rotstein C.; Jiang X.; Heyland D.K.
**SOURCE**
 Canadian Journal of Anesthesia (2011) 58:3 (275-284). Date of Publication: March 2011
**VOLUME**
 58
**ISSUE**
 3
**FIRST PAGE**
 275
**LAST PAGE**
 284
**DATE OF PUBLICATION**
 March 2011
**DOI**
 10.1007/s12630-010-9439-5

**RECORD 297**
**TITLE**
 **Peritonitis due to Aspergillus nidulans and its effective treatment with voriconazole: The first case report**
**AUTHOR NAMES**
 Ulusoy Ş.; Özkan G.; Tosun I.; Kaynar K.; Köksal I.; Türkyilmaz S.; Vetem I.
**SOURCE**
 Peritoneal Dialysis International (2011) 31:2 (212-213). Date of Publication: March/April 2011
**VOLUME**
 31
**ISSUE**
 2
**FIRST PAGE**
 212
**LAST PAGE**
 213
**DATE OF PUBLICATION**
 March/April 2011
**DOI**
 10.3747/pdi.2010.00119

**RECORD 298**
**TITLE**
 **New molecular and surrogate biomarker-based tests in the diagnosis of bacterial and fungal infection in febrile neutropenic patients**
**AUTHOR NAMES**
 Chen S.C.-A.; Kontoyiannis D.P.
**SOURCE**
 Current Opinion in Infectious Diseases (2010) 23:6 (567-577). Date of Publication: December 2010
**VOLUME**
 23
**ISSUE**
 6
**FIRST PAGE**
 567
**LAST PAGE**
 577
**DATE OF PUBLICATION**
 December 2010
**DOI**
 10.1097/QCO.0b013e32833ef7d1

**RECORD 299**
**TITLE**
 **Candida score combined with procalcitonin to diagnose invasive candidiasis in surgical critically ill patients: A retrospective study**
**AUTHOR NAMES**
 Guan X.; Li X.; Wu R.; Chen J.
**SOURCE**
 Critical Care Medicine (2010) 38 SUPPL. 12 (A115). Date of Publication: December 2010
**VOLUME**
 38
**FIRST PAGE**
 A115
**DATE OF PUBLICATION**
 December 2010
**DOI**
 10.1097/01.ccm.0000390903.16849.8c

**RECORD 300**
**TITLE**
 **Isolation and characterization of an imported extremely-resistant Pseudomonas aeruginosa producing three different extended-spectrum β-lactamases and hyperproducing two multidrug-efflux pumps**
**AUTHOR NAMES**
 Ratkai C.; Nagy E.; Peixe L.; Bertalan V.; Hajdú E.
**SOURCE**
 Journal of Infection (2010) 61:6 (511-512). Date of Publication: December 2010
**VOLUME**
 61
**ISSUE**
 6
**FIRST PAGE**
 511
**LAST PAGE**
 512
**DATE OF PUBLICATION**
 December 2010
**DOI**
 10.1016/j.jinf.2010.10.006

**RECORD 301**
**TITLE**
 **The antimicrobial peptides derived from chromogranin/secretogranin family, new actors of innate immunity**
**AUTHOR NAMES**
 Shooshtarizadeh P.; Zhang D.; Chich J.-F.; Gasnier C.; Schneider F.; Haïkel Y.; Aunis D.; Metz-Boutigue M.-H.
**SOURCE**
 Regulatory Peptides (2010) 165:1 (102-110). Date of Publication: 30 Nov 2010
**VOLUME**
 165
**ISSUE**
 1
**FIRST PAGE**
 102
**LAST PAGE**
 110
**DATE OF PUBLICATION**
 30 Nov 2010
**DOI**
 10.1016/j.regpep.2009.11.014

**RECORD 302**
**TITLE**
 **Primary cutaneous cryptococcosis in a renal transplant recipient: Case report**
**AUTHOR NAMES**
 Zorman J.V.; Zupanc T.L.; Parac Z.; Cucek I.
**SOURCE**
 Mycoses (2010) 53:6 (535-537). Date of Publication: November 2010
**VOLUME**
 53
**ISSUE**
 6
**FIRST PAGE**
 535
**LAST PAGE**
 537
**DATE OF PUBLICATION**
 November 2010
**DOI**
 10.1111/j.1439-0507.2009.01737.x

**RECORD 303**
**TITLE**
 **The clinical application of serum procalcitonin in infectious disease**
**AUTHOR NAMES**
 Guo L.; Xiong M.; Fan H.
**SOURCE**
 Clinical Chemistry (2010) 56:6 SUPPL. 1 (A45). Date of Publication: October 2010
**VOLUME**
 56
**ISSUE**
 6
**FIRST PAGE**
 A45
**DATE OF PUBLICATION**
 October 2010

**RECORD 304**
**TITLE**
 **Procalcitonin as a predictor of blood culture results in critically ill patients**
**AUTHOR NAMES**
 Ng S.; Faix J.D.; Park P.W.
**SOURCE**
 Clinical Chemistry (2010) 56:6 SUPPL. 1 (A48). Date of Publication: October 2010
**VOLUME**
 56
**ISSUE**
 6
**FIRST PAGE**
 A48
**DATE OF PUBLICATION**
 October 2010

**RECORD 305**
**TITLE**
 **Assessment of candida score together with serum procalcitonin in ICU patients: Predictor of invasive candidiasis?**
**AUTHOR NAMES**
 Li X.; Wu R.; Chen J.; Guan X.; Huang S.
**SOURCE**
 Intensive Care Medicine (2010) 36 SUPPL. 2 (S251). Date of Publication: September 2010
**VOLUME**
 36
**FIRST PAGE**
 S251
**DATE OF PUBLICATION**
 September 2010
**DOI**
 10.1007/s00134-010-2000-8

**RECORD 306**
**TITLE**
 **Molecular biology-based methods for microorganism detection in ICU patients**
**AUTHOR NAMES**
 Prucha M.; Zazula R.; Pekova S.; Stastny P.; Muller M.; Tyll T.
**SOURCE**
 Intensive Care Medicine (2010) 36 SUPPL. 2 (S200). Date of Publication: September 2010
**VOLUME**
 36
**FIRST PAGE**
 S200
**DATE OF PUBLICATION**
 September 2010
**DOI**
 10.1007/s00134-010-1999-x

**RECORD 307**
**TITLE**
 **Procalcitonin in the initial evaluation of septic patients admitted to the ICU**
**AUTHOR NAMES**
 Lozano-Sáez R.; Díez-de-los-Rios-Carrasco M.-J.; Seller-Pérez G.; Arrebola-Ramírez M.; Herrera-Gutierrez M.E.; Álvarez-Montero L.; Quesada-García G.
**SOURCE**
 Intensive Care Medicine (2010) 36 SUPPL. 2 (S134). Date of Publication: September 2010
**VOLUME**
 36
**FIRST PAGE**
 S134
**DATE OF PUBLICATION**
 September 2010
**DOI**
 10.1007/s00134-010-1999-x

**RECORD 308**
**TITLE**
 **Case report 3: Colorectal cancer patient**
**AUTHOR NAMES**
 Borges Sa M.
**SOURCE**
 Mycoses (2010) 53:SUPPL. 2 (8-9). Date of Publication: July 2010
**VOLUME**
 53
**ISSUE**
 SUPPL. 2
**FIRST PAGE**
 8
**LAST PAGE**
 9
**DATE OF PUBLICATION**
 July 2010
**DOI**
 10.1111/j.1439-0507.2010.01908.x

**RECORD 309**
**TITLE**
 **Procalcitonin levels in surgical patients at risk of candidemia**
**AUTHOR NAMES**
 Martini A.; Gottin L.; Menestrina N.; Schweiger V.; Simion D.; Vincent J.-L.
**SOURCE**
 Journal of Infection (2010) 60:6 (425-430). Date of Publication: June 2010
**VOLUME**
 60
**ISSUE**
 6
**FIRST PAGE**
 425
**LAST PAGE**
 430
**DATE OF PUBLICATION**
 June 2010
**DOI**
 10.1016/j.jinf.2010.03.003

**RECORD 310**
**TITLE**
 **Diagnostic value of procalcitonin, interleukin 8, interleukin 6, and c-reactive protein for detecting bacteremia and fungemia in cancer patients**
**ORIGINAL (NON-ENGLISH) TITLE**
 **Valor diagnóstico de la procalcitonina, la interleucina 8, la interleucina 6 y la proteína C reactiva en la deteccin de bacteriemia y fungemia en pacientes con cáncer**
**AUTHOR NAMES**
 Aznar-Oroval E.; Sánchez-Yepes M.; Lorente-Alegre P.; San Juan-Gadea M.C.; Ortiz-Muñoz B.; Prez-Ballestero P.; Picn-Roig I.; Maquez-Richart J.
**SOURCE**
 Enfermedades Infecciosas y Microbiologia Clinica (2010) 28:5 (273-277). Date of Publication: May 2010
**VOLUME**
 28
**ISSUE**
 5
**FIRST PAGE**
 273
**LAST PAGE**
 277
**DATE OF PUBLICATION**
 May 2010
**DOI**
 10.1016/j.eimc.2009.08.001

**RECORD 311**
**TITLE**
 **Evaluation of neutrophilic CD64, interleukin 10 and procalcitonin as diagnostic markers of early- and late-onset neonatal sepsis**
**AUTHOR NAMES**
 Zeitoun A.A.H.; Gad S.S.; Attia F.M.; Abu Maziad A.S.; Bell E.F.
**SOURCE**
 Scandinavian Journal of Infectious Diseases (2010) 42:4 (299-305). Date of Publication: 2010
**VOLUME**
 42
**ISSUE**
 4
**FIRST PAGE**
 299
**LAST PAGE**
 305
**DATE OF PUBLICATION**
 2010
**DOI**
 10.3109/00365540903449832

**RECORD 312**
**TITLE**
 **Community acquired pneumonia and tuberculosis a cliniciańs dilemma**
**AUTHOR NAMES**
 Lalloo U.G.; Nyamande K.
**SOURCE**
 Clinical Pulmonary Medicine (2010) 17:2 (61-65). Date of Publication: March 2010
**VOLUME**
 17
**ISSUE**
 2
**FIRST PAGE**
 61
**LAST PAGE**
 65
**DATE OF PUBLICATION**
 March 2010
**DOI**
 10.1097/CPM.0b013e3181d266d6

**RECORD 313**
**TITLE**
 **Use of plasma procalcitonin levels as an adjunct to clinical microbiology**
**AUTHOR NAMES**
 Gilbert D.N.
**SOURCE**
 Journal of Clinical Microbiology (2010) 48:7 (2325-2329). Date of Publication: July 2010
**VOLUME**
 48
**ISSUE**
 7
**FIRST PAGE**
 2325
**LAST PAGE**
 2329
**DATE OF PUBLICATION**
 July 2010
**DOI**
 10.1128/JCM.00655-10

**RECORD 314**
**TITLE**
 **Recommendations in the empiric anti-infective agents of intra-abdominal infection**
**ORIGINAL (NON-ENGLISH) TITLE**
 **Recomendaciones en el tratamiento antibiótico empírico de la infección intraabdominal**
**AUTHOR NAMES**
 Guirao X.; Arias J.; Badía J.Ma.; García-Rodríguez J.A.; Mensa J.; Álvarez-Lerma F.; Borges M.; Barberán J.; Maseda E.; Salavert M.; Llinares P.; Gobernado M.; Rey Garcia C.
**SOURCE**
 Revista Espanola de Quimioterapia (2009) 22:3 (151-172). Date of Publication: 2009
**VOLUME**
 22
**ISSUE**
 3
**FIRST PAGE**
 151
**LAST PAGE**
 172
**DATE OF PUBLICATION**
 2009

**RECORD 315**
**TITLE**
 **Serum procalcitonin levels in critically ill patients colonized with Candida spp: New clues for the early recognition of invasive candidiasis?**
**AUTHOR NAMES**
 Charles P.E.; Castro C.; Ruiz-Santana S.; León C.; Saavedra P.; Martín E.
**SOURCE**
 Intensive Care Medicine (2009) 35:12 (2146-2150). Date of Publication: December 2009
**VOLUME**
 35
**ISSUE**
 12
**FIRST PAGE**
 2146
**LAST PAGE**
 2150
**DATE OF PUBLICATION**
 December 2009
**DOI**
 10.1007/s00134-009-1623-0

**RECORD 316**
**TITLE**
 **Severe form of Legionnaires' disease in an immunocompetent patient**
**ORIGINAL (NON-ENGLISH) TITLE**
 **Teška forma legionarske bolesti kod imunokompetentnog bolesnika**
**AUTHOR NAMES**
 Andrijević I.; Matijašević J.; Považan D.; Kojičić M.; Batranović U.
**SOURCE**
 Vojnosanitetski Pregled (2009) 66:12 (1010-1014). Date of Publication: December 2009
**VOLUME**
 66
**ISSUE**
 12
**FIRST PAGE**
 1010
**LAST PAGE**
 1014
**DATE OF PUBLICATION**
 December 2009

**RECORD 317**
**TITLE**
 **Diagnosis of hospital-acquired pneumonia and methods of testing for pathogens**
**SOURCE**
 Respirology (2009) 14:SUPPL. 2 (S10-S22). Date of Publication: November 2009
**VOLUME**
 14
**ISSUE**
 SUPPL. 2
**DATE OF PUBLICATION**
 November 2009
**DOI**
 10.1111/j.1440-1843.2009.01572.x

**RECORD 318**
**TITLE**
 **Updates in community-acquired pneumopathy**
**ORIGINAL (NON-ENGLISH) TITLE**
 **Actualités dans la pneumopathie communautaire**
**AUTHOR NAMES**
 Catherinot E.
**SOURCE**
 Revue des Maladies Respiratoires Actualites (2009) 1:5 (545-547). Date of Publication: November 2009
**VOLUME**
 1
**ISSUE**
 5
**FIRST PAGE**
 545
**LAST PAGE**
 547
**DATE OF PUBLICATION**
 November 2009
**DOI**
 10.1016/S1877-1203(09)72537-6

**RECORD 319**
**TITLE**
 **Severe hospital-acquired pneumonia: A review for clinicians**
**AUTHOR NAMES**
 Dallas J.; Kollef M.
**SOURCE**
 Current Infectious Disease Reports (2009) 11:5 (349-356). Date of Publication: 2009
**VOLUME**
 11
**ISSUE**
 5
**FIRST PAGE**
 349
**LAST PAGE**
 356
**DATE OF PUBLICATION**
 2009
**DOI**
 10.1007/s11908-009-0050-2

**RECORD 320**
**TITLE**
 **Procalcitonin Serum Concentration During Pneumocystis Jiroveci Colonization or Pseudomonas Aeruginosa Infection/Colonization in Lung Transplant Recipients**
**AUTHOR NAMES**
 Zeglen S.; Wojarski J.; Wozniak-Grygiel E.; Siola M.; Szewczyk M.; Kucewicz-Czech E.; Nozynski J.; Zembala M.
**SOURCE**
 Transplantation Proceedings (2009) 41:8 (3225-3227). Date of Publication: October 2009
**VOLUME**
 41
**ISSUE**
 8
**FIRST PAGE**
 3225
**LAST PAGE**
 3227
**DATE OF PUBLICATION**
 October 2009
**DOI**
 10.1016/j.transproceed.2009.08.007

**RECORD 321**
**TITLE**
 **Early introduction of peritoneal dialysis may improve survival in severe sepsis**
**AUTHOR NAMES**
 Szakszon K.; Csízy I.; Szabó T.
**SOURCE**
 Pediatric Emergency Care (2009) 25:9 (599-602). Date of Publication: September 2009
**VOLUME**
 25
**ISSUE**
 9
**FIRST PAGE**
 599
**LAST PAGE**
 602
**DATE OF PUBLICATION**
 September 2009
**DOI**
 10.1097/PEC.0b013e3181b922ba

**RECORD 322**
**TITLE**
 **Utility of a commercially available multiplex real-time PCR assay to detect bacterial and fungal pathogens in febrile neutropenia**
**AUTHOR NAMES**
 Von Lilienfeld-Toal M.; Lehmann L.E.; Raadts A.D.; Hahn-Ast C.; Orlopp K.S.; Marklein G.; Purr I.; Cook G.; Hoeft A.; Glasmacher A.; Stüber F.
**SOURCE**
 Journal of Clinical Microbiology (2009) 47:8 (2405-2410). Date of Publication: August 2009
**VOLUME**
 47
**ISSUE**
 8
**FIRST PAGE**
 2405
**LAST PAGE**
 2410
**DATE OF PUBLICATION**
 August 2009
**DOI**
 10.1128/JCM.00491-09

**RECORD 323**
**TITLE**
 **Effectiveness of posaconazole in antifungal profilaxis in the ANLL and allo BMT treatment**
**AUTHOR NAMES**
 Pavone V.; De Francesco R.; Del Casale C.; Greco G.; Leo L.; Lobreglio G.; Mele A.; Messa A.; Pasanisi G.; Rana A.; Rossini B.; Sibilla S.
**SOURCE**
 Haematologica (2009) 94 SUPPL. 2 (685). Date of Publication: June 2009
**VOLUME**
 94
**FIRST PAGE**
 685
**DATE OF PUBLICATION**
 June 2009

**RECORD 324**
**TITLE**
 **Efficacy of procalcitonin in the early diagnosis of bacterial infections in a critical care unit**
**AUTHOR NAMES**
 Nakamura A.; Wada H.; Ikejiri M.; Hatada T.; Sakurai H.; Matsushima Y.; Nishioka J.; Maruyama K.; Isaji S.; Takeda T.; Nobori T.
**SOURCE**
 Shock (2009) 31:6 (586-591). Date of Publication: June 2009
**VOLUME**
 31
**ISSUE**
 6
**FIRST PAGE**
 586
**LAST PAGE**
 591
**DATE OF PUBLICATION**
 June 2009
**DOI**
 10.1097/SHK.0b013e31819716fa

**RECORD 325**
**TITLE**
 **Neonatal sepsis and septic shock: Current trends in epidemiology and management**
**AUTHOR NAMES**
 Sherlock R.
**SOURCE**
 Journal of Pediatric Infectious Diseases (2009) 4:2 (153-159). Date of Publication: 2009
 Sepsis and Septic Shock: Global Perspectives, Book Series Title:
**VOLUME**
 4
**ISSUE**
 2
**FIRST PAGE**
 153
**LAST PAGE**
 159
**DATE OF PUBLICATION**
 2009
**DOI**
 10.3233/JPI-2009-0161

**RECORD 326**
**TITLE**
 **Respiratory Infections Research: a Perspective From the Tuberculosis and Respiratory Infections Area (TIR)**
**ORIGINAL (NON-ENGLISH) TITLE**
 **Investigación en infecciones respiratorias: una perspectiva desde el Área Tuberculosis e Infecciones Respiratorias (TIR)**
**AUTHOR NAMES**
 Jesús Cremades M.; Luiza de Souza-Galvão M.; García J.M.; Menéndez R.
**SOURCE**
 Archivos de Bronconeumologia (2009) 45:SUPPL. 1 (11-15). Date of Publication: 2009
**VOLUME**
 45
**ISSUE**
 SUPPL. 1
**FIRST PAGE**
 11
**LAST PAGE**
 15
**DATE OF PUBLICATION**
 2009
**DOI**
 10.1016/S0300-2896(09)70265-0

**RECORD 327**
**TITLE**
 **The serum concentration of procalcitonin (PCT) in various infections in HIV positive patients**
**AUTHOR NAMES**
 Mikuła T.; Lipowski D.; Stańczak W.
**SOURCE**
 HIV and AIDS Review (2008) 7:2 (5-9). Date of Publication: 2008
**VOLUME**
 7
**ISSUE**
 2
**FIRST PAGE**
 5
**LAST PAGE**
 9
**DATE OF PUBLICATION**
 2008
**DOI**
 10.1016/S1730-1270(10)60002-1

**RECORD 328**
**TITLE**
 **Fungal infection in children with oncohematologic disorders - Is early diagnostics possible?**
**ORIGINAL (NON-ENGLISH) TITLE**
 **Zakażenia grzybicze u dzieci z chorobami rozrostowymi układu krwiotwórczego - Czy wczesna diagnostyka jest możliwa?**
**AUTHOR NAMES**
 Irga N.; Grabiec-Wiśniewska A.; Kosiak W.; Komarnicka J.
**SOURCE**
 Onkologia Polska (2008) 11:2 (82-86). Date of Publication: 2008
**VOLUME**
 11
**ISSUE**
 2
**FIRST PAGE**
 82
**LAST PAGE**
 86
**DATE OF PUBLICATION**
 2008

**RECORD 329**
**TITLE**
 **Progress in medical management of intra-abdominal infection**
**AUTHOR NAMES**
 Laterre P.-F.
**SOURCE**
 Current Opinion in Infectious Diseases (2008) 21:4 (393-398). Date of Publication: August 2008
**VOLUME**
 21
**ISSUE**
 4
**FIRST PAGE**
 393
**LAST PAGE**
 398
**DATE OF PUBLICATION**
 August 2008
**DOI**
 10.1097/QCO.0b013e328306ef2a

**RECORD 330**
**TITLE**
 **Report from the 25th International Congress of Chemotherapy and the 17th European Congress of Clinical Microbiology and Infectious Diseases**
**ORIGINAL (NON-ENGLISH) TITLE**
 **Zpráva z kongresu 25th International Congress of Chemotherapy a 17th European Congress of Clinical Microbiology and Infectious Diseases**
**AUTHOR NAMES**
 Holub M.; Beran O.
**SOURCE**
 Klinicka Mikrobiologie a Infekcni Lekarstvi (2007) 13:4 (165-167). Date of Publication: 2007
**VOLUME**
 13
**ISSUE**
 4
**FIRST PAGE**
 165
**LAST PAGE**
 167
**DATE OF PUBLICATION**
 2007

**RECORD 331**
**TITLE**
 **Procalcitonin (PCT) and C-reactive Protein (CRP) as severe systemic infection markers in febrile neutropenic adults**
**AUTHOR NAMES**
 Massaro K.S.R.; Costa S.F.; Leone C.; Chamone D.A.F.
**SOURCE**
 BMC Infectious Diseases (2007) 7 Article Number: 137. Date of Publication: 22 Nov 2007
**VOLUME**
 7
**DATE OF PUBLICATION**
 22 Nov 2007
**DOI**
 10.1186/1471-2334-7-137

**RECORD 332**
**TITLE**
 **Evaluation of serum procalcitonin concentration in the ICU following severe burn**
**AUTHOR NAMES**
 Bargues L.; Chancerelle Y.; Catineau J.; Jault P.; Carsin H.
**SOURCE**
 Burns (2007) 33:7 (860-864). Date of Publication: November 2007
**VOLUME**
 33
**ISSUE**
 7
**FIRST PAGE**
 860
**LAST PAGE**
 864
**DATE OF PUBLICATION**
 November 2007
**DOI**
 10.1016/j.burns.2006.10.401

**RECORD 333**
**TITLE**
 **Response to Letter to the Editor**
**AUTHOR NAMES**
 Lavrentia A.; Kontakiotis T.
**SOURCE**
 Burns (2007) 33:7 (941-942). Date of Publication: November 2007
**VOLUME**
 33
**ISSUE**
 7
**FIRST PAGE**
 941
**LAST PAGE**
 942
**DATE OF PUBLICATION**
 November 2007
**DOI**
 10.1016/j.burns.2007.02.004

**RECORD 334**
**TITLE**
 **Aseptic meningitis**
**AUTHOR NAMES**
 Lee B.E.; Davies H.D.
**SOURCE**
 Current Opinion in Infectious Diseases (2007) 20:3 (272-277). Date of Publication: June 2007
**VOLUME**
 20
**ISSUE**
 3
**FIRST PAGE**
 272
**LAST PAGE**
 277
**DATE OF PUBLICATION**
 June 2007
**DOI**
 10.1097/QCO.0b013e3280ad4672

**RECORD 335**
**TITLE**
 **Effective treatment strategies for paediatric community-acquired pneumonia**
**AUTHOR NAMES**
 Atkinson M.; Yanney M.; Stephenson T.; Smyth A.
**SOURCE**
 Expert Opinion on Pharmacotherapy (2007) 8:8 (1091-1101). Date of Publication: June 2007
**VOLUME**
 8
**ISSUE**
 8
**FIRST PAGE**
 1091
**LAST PAGE**
 1101
**DATE OF PUBLICATION**
 June 2007
**DOI**
 10.1517/14656566.8.8.1091

**RECORD 336**
**TITLE**
 **Bile duct infections as a late complication after endoscopic sphincterotomy**
**ORIGINAL (NON-ENGLISH) TITLE**
 **Zakażenia dróg żółciowych jako późne powikłanie sfinkterotomii endoskopowej**
**AUTHOR NAMES**
 Mandryka Y.; Klimczak J.; Duszewski M.; Kondras M.; Modzelewski B.
**SOURCE**
 Polski Merkuriusz Lekarski (2006) 21:126 (525-527). Date of Publication: 2006
**VOLUME**
 21
**ISSUE**
 126
**FIRST PAGE**
 525
**LAST PAGE**
 527
**DATE OF PUBLICATION**
 2006

**RECORD 337**
**TITLE**
 **Markers of acute inflammation in assessing and managing lower respiratory tract infections: Focus on procalcitonin**
**AUTHOR NAMES**
 Müller B.; Prat C.
**SOURCE**
 Clinical Microbiology and Infection (2006) 12:SUPPL. 9 (8-16). Date of Publication: December 2006
**VOLUME**
 12
**ISSUE**
 SUPPL. 9
**FIRST PAGE**
 8
**LAST PAGE**
 16
**DATE OF PUBLICATION**
 December 2006
**DOI**
 10.1111/j.1469-0691.2006.01654.x

**RECORD 338**
**TITLE**
 **Vascular catheter-related infection and sepsis**
**AUTHOR NAMES**
 Donelli G.
**SOURCE**
 Surgical Infections (2006) 7:SUPPL. 2 (S-25-S-27). Date of Publication: 2006
**VOLUME**
 7
**ISSUE**
 SUPPL. 2
**DATE OF PUBLICATION**
 2006

**RECORD 339**
**TITLE**
 **Nosocomial pneumonia**
**AUTHOR NAMES**
 Ostendorf U.; Ewig S.; Torres A.
**SOURCE**
 Current Opinion in Infectious Diseases (2006) 19:4 (327-338). Date of Publication: August 2006
**VOLUME**
 19
**ISSUE**
 4
**FIRST PAGE**
 327
**LAST PAGE**
 338
**DATE OF PUBLICATION**
 August 2006
**DOI**
 10.1097/01.qco.0000235158.40184.28

**RECORD 340**
**TITLE**
 **Prospective evaluation of procalcitonin in adults with non-neutropenic fever after allogeneic hematopoietic stem cell transplantation**
**AUTHOR NAMES**
 Ortega M.; Rovira M.; Filella X.; Martínez J.A.; Almela M.; Puig J.; Carreras E.; Mensa J.
**SOURCE**
 Bone Marrow Transplantation (2006) 37:5 (499-502). Date of Publication: March 2006
**VOLUME**
 37
**ISSUE**
 5
**FIRST PAGE**
 499
**LAST PAGE**
 502
**DATE OF PUBLICATION**
 March 2006
**DOI**
 10.1038/sj.bmt.1705262

**RECORD 341**
**TITLE**
 **Vasculitis and infection: Effects of immunosuppressive therapy**
**AUTHOR NAMES**
 Lode H.M.; Schmidt-Ioanas M.
**SOURCE**
 Clinical Nephrology (2005) 64:6 (475-479). Date of Publication: December 2005
**VOLUME**
 64
**ISSUE**
 6
**FIRST PAGE**
 475
**LAST PAGE**
 479
**DATE OF PUBLICATION**
 December 2005

**RECORD 342**
**TITLE**
 **Are vaginal fluid procalcitonin levels useful for the prediction of subclinial infection in patients with preterm premature rupture of membranes?**
**AUTHOR NAMES**
 Torbé A.; Czajka R.
**SOURCE**
 Journal of Obstetrics and Gynaecology Research (2005) 31:5 (464-470). Date of Publication: October 2005
**VOLUME**
 31
**ISSUE**
 5
**FIRST PAGE**
 464
**LAST PAGE**
 470
**DATE OF PUBLICATION**
 October 2005
**DOI**
 10.1111/j.1447-0756.2005.00321.x

**RECORD 343**
**TITLE**
 **The role of HIV infection in acute respiratory infections among children in sub-Saharan Africa**
**AUTHOR NAMES**
 Jeena P.
**SOURCE**
 International Journal of Tuberculosis and Lung Disease (2005) 9:7 (708-715). Date of Publication: July 2005
**VOLUME**
 9
**ISSUE**
 7
**FIRST PAGE**
 708
**LAST PAGE**
 715
**DATE OF PUBLICATION**
 July 2005

**RECORD 344**
**TITLE**
 **Procalcitonin - A marker of invasive fungal infection?**
**AUTHOR NAMES**
 Dornbusch H.J.; Strenger V.; Kerbl R.; Lackner H.; Schwinger W.; Sovinz P.; Urban C.
**SOURCE**
 Supportive Care in Cancer (2005) 13:5 (343-346). Date of Publication: May 2005
**VOLUME**
 13
**ISSUE**
 5
**FIRST PAGE**
 343
**LAST PAGE**
 346
**DATE OF PUBLICATION**
 May 2005
**DOI**
 10.1007/s00520-004-0721-3

**RECORD 345**
**TITLE**
 **Value of measuring serum procalcitonin, C-reactive protein, and mannan antigens to distinguish fungal from bacterial infections**
**AUTHOR NAMES**
 Petrikkos G.L.; Christofilopoulou S.A.; Tentolouris N.K.; Charvalos E.A.; Kosmidis C.J.; Daikos G.L.
**SOURCE**
 European Journal of Clinical Microbiology and Infectious Diseases (2005) 24:4 (272-275). Date of Publication: April 2005
**VOLUME**
 24
**ISSUE**
 4
**FIRST PAGE**
 272
**LAST PAGE**
 275
**DATE OF PUBLICATION**
 April 2005
**DOI**
 10.1007/s10096-005-1312-z

**RECORD 346**
**TITLE**
 **Nosocomial enterococcal infection in neonates [1]**
**AUTHOR NAMES**
 Bilikova E.; Koprnova J.; Hafed B.M.; Svetlansky I.; Chovancova D.; Drobna M.; Huttova M.; Krcmery V.
**SOURCE**
 International Journal of Infectious Diseases (2004) 8:2 (127-129). Date of Publication: March 2004
**VOLUME**
 8
**ISSUE**
 2
**FIRST PAGE**
 127
**LAST PAGE**
 129
**DATE OF PUBLICATION**
 March 2004
**DOI**
 10.1016/j.ijid.2003.12.001

**RECORD 347**
**TITLE**
 **Procalcitonin serum levels in perinatal bacterial and fungal infection of preterm infants**
**AUTHOR NAMES**
 Distefano G.; Curreri R.; Betta P.; Romeo M.G.; Amato M.
**SOURCE**
 Acta Paediatrica, International Journal of Paediatrics (2004) 93:2 (216-219). Date of Publication: February 2004
**VOLUME**
 93
**ISSUE**
 2
**FIRST PAGE**
 216
**LAST PAGE**
 219
**DATE OF PUBLICATION**
 February 2004
**DOI**
 10.1080/08035250310021082

**RECORD 348**
**TITLE**
 **Sepsis: A frequent, life-threatening syndrome**
**AUTHOR NAMES**
 Jacobi J.
**SOURCE**
 Pharmacotherapy (2002) 22:12 II (169S-181S). Date of Publication: 1 Dec 2002
**VOLUME**
 22
**ISSUE**
 12 II
**DATE OF PUBLICATION**
 1 Dec 2002

**RECORD 349**
**TITLE**
 **Infections in the immunocompromised rheumatologic patient**
**AUTHOR NAMES**
 Greenberg S.B.
**SOURCE**
 Critical Care Clinics (2002) 18:4 (931-956). Date of Publication: November 2002
**VOLUME**
 18
**ISSUE**
 4
**FIRST PAGE**
 931
**LAST PAGE**
 956
**DATE OF PUBLICATION**
 November 2002
**DOI**
 10.1016/S0749-0704(02)00022-2

**RECORD 350**
**TITLE**
 **Evaluation of parallel operated small-scale bubble columns for microbial process development using Staphylococcus carnosus**
**AUTHOR NAMES**
 Dilsen S.; Paul W.; Herforth D.; Sandgathe A.; Altenbach-Rehm J.; Freudl R.; Wandrey C.; Weuster-Botz D.
**SOURCE**
 Journal of Biotechnology (2001) 88:1 (77-84). Date of Publication: 1 Jun 2001
**VOLUME**
 88
**ISSUE**
 1
**FIRST PAGE**
 77
**LAST PAGE**
 84
**DATE OF PUBLICATION**
 1 Jun 2001
**DOI**
 10.1016/S0168-1656(01)00265-6

**RECORD 351**
**TITLE**
 **Procalcitonin as a marker of nosocomial infections in the neonatal intensive care unit**
**AUTHOR NAMES**
 Chiesa C.; Pacifico L.; Rossi N.; Panero A.; Matrunola M.; Mancuso G.
**SOURCE**
 Intensive Care Medicine, Supplement (2000) 26:2 (S175-S177). Date of Publication: 2000
**VOLUME**
 26
**ISSUE**
 2
**DATE OF PUBLICATION**
 2000

**RECORD 352**
**TITLE**
 **Procalcitonin: A new marker for diagnosis of acute rejection and bacterial infection in patients after heart and lung transplantation**
**AUTHOR NAMES**
 Hammer S.; Meisner F.; Dirschedl P.; Höbel G.; Fraunberger P.; Meiser B.; Reichardt B.; Hammer C.
**SOURCE**
 Transplant Immunology (1998) 6:4 (235-241). Date of Publication: December 1998
**VOLUME**
 6
**ISSUE**
 4
**FIRST PAGE**
 235
**LAST PAGE**
 241
**DATE OF PUBLICATION**
 December 1998
**DOI**
 10.1016/S0966-3274(98)80013-0

**RECORD 353**
**TITLE**
 **Failure of PCT to indicate severe fungal infection in two immunodeficient patients [1]**
**AUTHOR NAMES**
 Huber W.; Schweigart U.; Bottermann P.
**SOURCE**
 Infection (1997) 25:6 (377-378). Date of Publication: 1997
**VOLUME**
 25
**ISSUE**
 6
**FIRST PAGE**
 377
**LAST PAGE**
 378
**DATE OF PUBLICATION**
 1997
